# Bi-Catalyzed Trifluoromethylation of C(sp^2^)–H Bonds under Light

**DOI:** 10.1021/jacs.3c10333

**Published:** 2023-11-14

**Authors:** Takuya Tsuruta, Davide Spinnato, Hye Won Moon, Markus Leutzsch, Josep Cornella

**Affiliations:** Max-Planck-Institut für Kohlenforschung, Kaiser-Wilhelm-Platz 1, Mülheim an der Ruhr, 45470, Germany

## Abstract

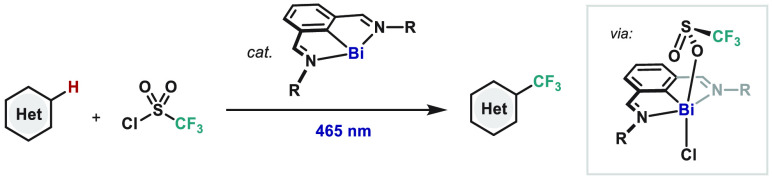

We disclose a Bi-catalyzed C–H trifluoromethylation of (hetero)arenes using CF_3_SO_2_Cl under light irradiation. The catalytic method permits the direct functionalization of various heterocycles bearing distinct functional groups. The structural and computational studies suggest that the process occurs through an open-shell redox manifold at bismuth, comprising three unusual elementary steps for a main group element. The catalytic cycle starts with rapid oxidative addition of CF_3_SO_2_Cl to a low-valent Bi(I) catalyst, followed by a light-induced homolysis of Bi(III)–O bond to generate a trifluoromethyl radical upon extrusion of SO_2_, and is closed with a hydrogen-atom transfer to a Bi(II) radical intermediate.

Direct trifluoromethylation of heteroarenes has become a powerful synthetic tool^1,2^ that enables facile modification of active pharmaceutical ingredient-like molecules (API) with peripheral fluorinated methyl moieties.^3^ In this regard, many successful catalytic strategies for the generation of the trifluoromethyl radical have appeared in the literature,^4,5^ based on photochemical^6,7^ or thermal approaches.^1,2,8^ From the reactivity standpoint, however, the majority display a common aspect: the generation of the CF_3_ radical generally occurs through outer-sphere single electron transfer (SET) processes. Albeit minor, catalytic examples employing transition-metal–CF_3_ species and derivatives have been reported, which release the CF_3_ radical upon external stimuli.^8d,9^ We set out to expand this latter concept to bismuth redox catalysis, a group 15 element,^10−12^ given the demonstrated ability to homolyze Bi(III)–X bonds (X = C, N, O) under thermal or photochemical conditions.^13^ On the basis of recent discoveries on oxidative addition to *N,C,N*-pincer Bi(I) complexes,^12e,12h,14^ we expected that a direct C–H trifluoromethylation reaction would be within reach. In this communication, we disclose a Bi-catalyzed trifluoromethylation of heteroarenes using CF_3_SO_2_Cl under light irradiation (Figure 1). The protocol features open-shell Bi redox catalysis, comprising three elementary steps that have few precedents in main-group catalysis, namely (1) oxidative addition (OA) of CF_3_SO_2_Cl to Bi(I); (2) ligand-to-ligand charge transfer (LLCT) leading to facile scission of the Bi(III)–O bond,^13j,13k^ generation of CF_3_ radical upon release of SO_2_; and (3) hydrogen atom transfer (HAT) by the transient Bi(II) radical intermediate that allows rearomatization and formation of the Ar–CF_3_ product. We gathered experimental evidence of these steps through stoichiometric reactivity of Bi(III) intermediates as well as structural, spectroscopic, and computational analysis.

**Figure 1:**
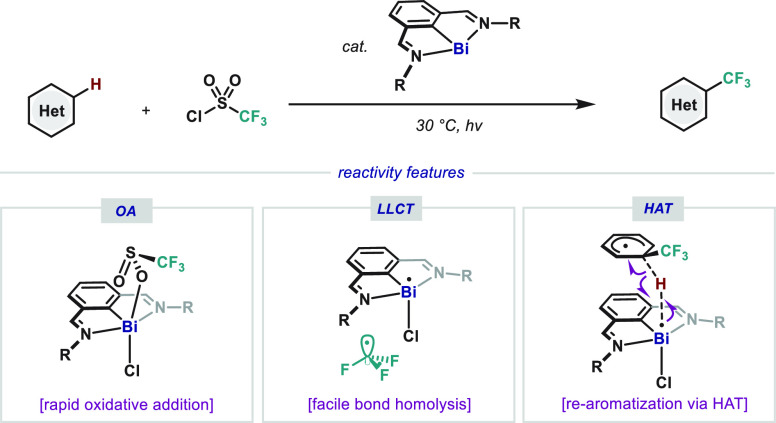
Bi(I)-catalyzed radical trifluoromethylation of (hetero)arenes mediated by light.

After evaluation of the reaction parameters (see Supporting Information (SI) for details), we found that 1,3,5-trimethoxybenzene (**2a**) undergoes smooth trifluoromethylation with CF_3_SO_2_Cl (**3**) to afford **4a** in high yields in the presence of 10 mol % of Dostál-type complex **1a** or **1b** under light irradiation (Table 1, entries 1 and 2).^12c,15^ Replacement of the ^*t*^Bu by mesityl groups (**1c**) significantly decreases the yield of **4a** (entry 3). Chlorinated solvents proved to be suitable for this transformation; among them, CHCl_3_ afforded **4a** in the highest yield (entries 1, 4, and 5). The use of other polar solvents was not beneficial (entries 6–8). Although addition of these bases did not have a substantial effect on highly reactive **2a** (entries 9–10), it proved beneficial for other less reactive substrates (*vide infra*). The reaction did not deliver any trifluoromethylated product when performed in the dark (entry 11), thus pointing to the crucial role of light irradiation.

**Table 1:**
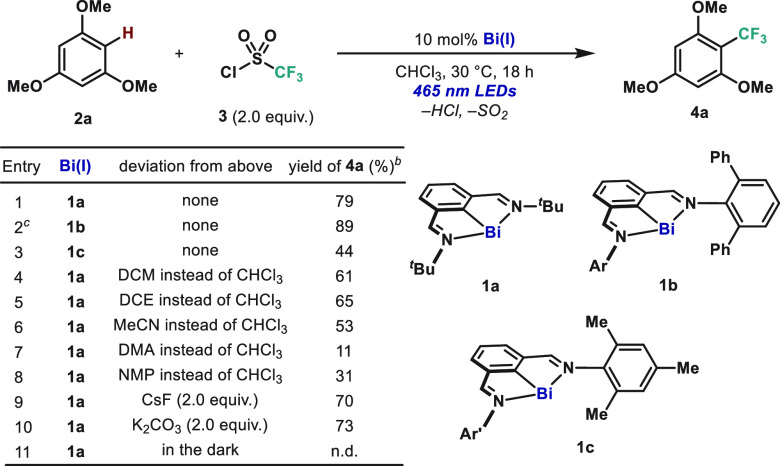
Optimization of the Bi-Catalyzed Trifluoromethylation of (Hetero)arenes^a^

a Reaction conditions: **2a** (1.0 equiv, 0.05 mmol) and **3** (2.0 equiv) in the presence of bismuthinidene **1** (10 mol %) under 465 nm LEDs irradiation at 30 °C for 18 h under Ar atmosphere.

b Yields were determined by ^19^F NMR with benzotrifluoride as the internal standard.

c White LEDs were used.

Having established the catalytic protocol, we investigated the substrate scope of our Bi(I)-catalyzed direct C–H trifluoromethylation; as shown in Table 2, this protocol is applicable to a variety of electron-rich and electron-deficient (hetero)arenes. Electron-rich arenes bearing aldehyde and ketone groups were tolerated, attaining trifluoromethylated products (**4b**, **4c**, and **4d**) in moderate yields. Trimethoxybenzene bearing a bromo group (**2e**) was also amenable to the reaction conditions. Mesitylene (**2f**) was successfully trifluoromethylated, giving **4f** in 50% yield. A 2,4,6-trimethoxypyrimidine delivered trifluoromethylated product **4g** in high yield. The reaction of 2,6-dimethoxypyridine proceeded to afford **4h** in a moderate yield. Remarkably, electron-deficient pyrazine-based heteroarene performed well, affording product **4i** in 56% yield. Pyrone was also successfully trifluoromethylated (**4j**). Both *N*-methyl and *N*-Boc protected pyrrole were tolerated (**4k** and **4l**). Also, the reaction using thiophene furnished the desired product **4m** in a good yield. The trifluoromethylation of *N*-benzyl-3-methylindole occurred at the C2 position in a good yield (**4n**). More complex biomolecules can also be trifluoromethylated, including (+)-mentofuran (**2o**), caffeine (**2p**), an uracil derivative (**2q**), brucin (**2r**), griseofulvin (**2s**), and sulfadoxine (**2t**). As observed in related radical trifluoromethylation reactions,^16^**4r** is formed via alkene isomerization followed by trifluoromethylation at the allylic position. In the case of **2s**, α-C–H bond trifluoromethylation occurred instead. This catalytic protocol is also applicable to C–H perfluoroalkylation using the corresponding sulfonyl chloride (product **4a′**). As shown in Table 2, CsF and K_2_CO_3_ were beneficial in the majority of cases in order to avoid the decomposition of the Bi catalyst (*vide infra* and SI). Unfortunately, trifluoromethylation of more challenging substrates such as toluene or pyridine were beyond the scope of this protocol.

**Table 2:**
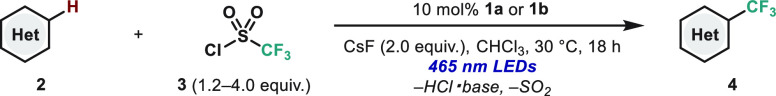

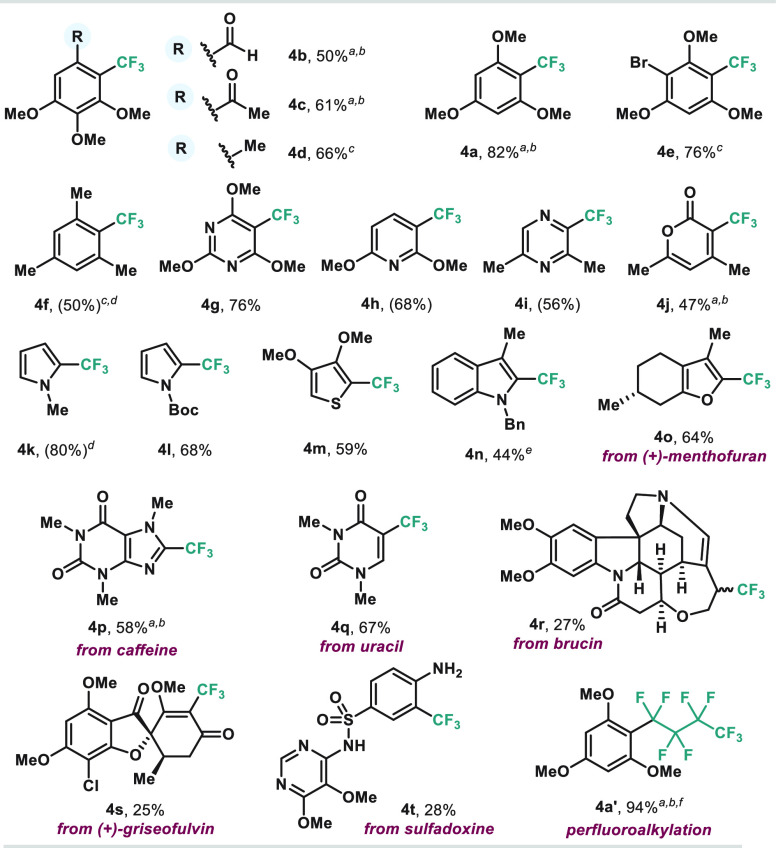
Scope of the Bi-Catalyzed C–H Trifluoromethylation of (Hetero)arenes

Isolated yields reported on 0.20 mmol scale unless otherwise noted. ^19^F NMR yields are shown for volatile products in parentheses using benzotrifluoride as the internal standard.

a White LEDs was used instead of blue LEDs.

b Without CsF.

c K_2_CO_3_ was used instead of CsF.

d A 2-fold excess of **2** was used against **3**.

e A 4-fold excess of **2** was used against **3**.

f C_4_F_9_SO_2_Cl was used instead of **3**.

On the basis of our previous work on oxidative addition to Bi(I) pincer complexes (either via radical or polar mechanisms),^12e^ we speculated that CF_3_SO_2_Cl (**3**, *E*_1/2_ = −0.18 vs SCE) is primed for reactivity with **1a** (*E*_1/2_ = −0.45 vs SCE).^5d^ Indeed, in the absence of light, treatment of **1a** with **3** in THF at 25 °C resulted in instantaneous formation of neutral Bi(III) complex **5a** (Figure 2). This complex was characterized by single-crystal X-ray diffraction (SC-XRD), thus confirming the *trans* disposition of the Cl and SO_2_CF_3_ anions in the solid state. The SO_2_CF_3_^–^ ligand is coordinated through the oxygen atom to the Bi-center (see SI for details).^17,18^ The Bi–O bond distances are 2.561(7) and 3.225(8) Å, respectively, indicating that the SO_2_CF_3_ group coordinates in an η^1^-fashion. When complex **5a** was irradiated under blue light in the presence of **2a** (5.0 equiv), **4a** was obtained in a 71% yield. This suggests that the Bi(III)–O bond in **5a** undergoes homolysis upon irradiation leading to the CF_3_SO_2_ radical. Subsequent extrusion of SO_2_ leads to a CF_3_ radical, which engages in C–C bond formation with the corresponding arene. Additionally, the use of **3** among trifluoromethylating reagents avoids α-fluorine elimination leading to CF_2_, commonly observed in the few reports on Bi(III)–CF_3_ complexes.^19,20^ In order to assess the formation of CF_3_ radicals, complex **5a** was treated with TEMPO under blue light. After 12 h, trifluoromethyl-TEMPO adduct **6** was observed by ^19^F NMR and HRMS, indicating radical generation upon irradiation. Noticeably, **6** was not detected in the absence of light. Therefore, light irradiation is necessary to homolyze the Bi–O bond and generate the CF_3_ radical.

**Figure 2:**
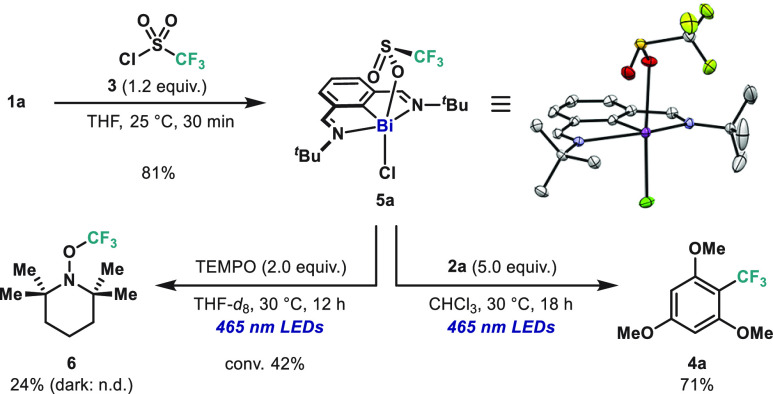
Synthesis of Bi(III) complex **5a** and SC-XRD structure (hydrogen atoms omitted for clarity) through oxidative addition of **1a** to **3**, and its reactivity toward generating the CF_3_ radical.

UV–vis spectroscopy revealed an absorption band centered at λ_max_ = 421 nm for complex **5a**. For the assignment of the observed absorption, time-dependent density functional theory (TD-DFT) calculations were conducted. As shown in Figure 3A, TD-DFT calculations of the optimized structure of **5a** indicate that transitions at 440 and 450 nm are dominated by HOMO to LUMO and HOMO to LUMO+1 transitions. The HOMO is essentially the nonbonding molecular orbital of the axial hypervalent bonding, localized on anionic SO_2_CF_3_ and Cl ligand (see SI for details). On the other hand, the LUMO and LUMO+1 can be described as π* orbitals delocalized throughout the *N,C,N*-pincer ligand. Therefore, the absorptions in the operating blue light regime are associated with LLCT processes that ultimately reduce the electron density of the hypervalent bonding orbitals and induce Bi–O bond homolysis.^21^

**Figure 3:**
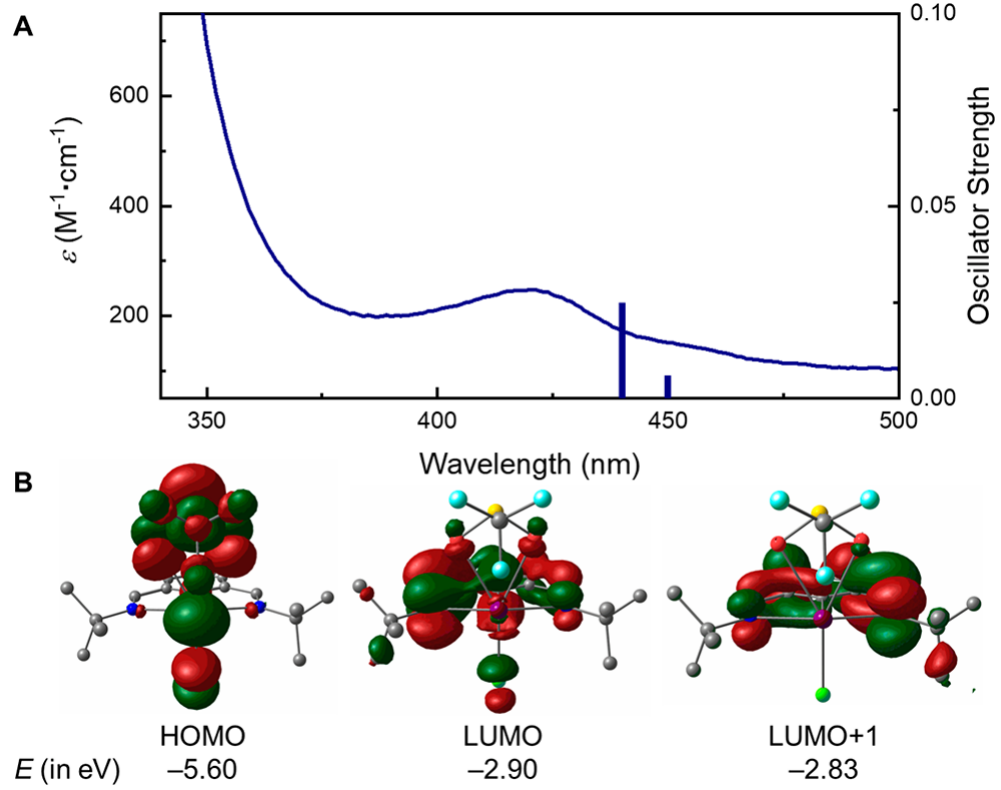
(A) Experimental UV–vis spectrum for **5a** (solid blue line) superimposed with the corresponding TD-DFT excited transitions (blue bars). (B) Molecular orbital isosurfaces and energies for **5a**. Hydrogen atoms have been omitted for clarity.

Mechanistically, after the addition of the CF_3_ radical to (hetero)arenes to afford intermediate **2u′** (Figure 4A), various pathways can be envisaged. Based on recent precedents,^12e^ an inner sphere radical–radical recombination followed by a polar E2-type elimination could occur (Figure 4A, *path a*). Additionally, two alternative radical pathways are also operative. On one hand, oxidation of **2u′** by the **Int-Ia** would lead to a cationic **2u′**^**+**^ (Figure 4A, *path b*). Based on recent Co-mediated trifluoromethylations, a direct HAT from **Int-Ia** is also plausible to proceed (Figure 4A, *path c*).^5g,22^ In order to discriminate between them experimentally, we designed an experiment employing redox-active ester **7** (Figure 4B). According to our previous work, after radical oxidative addition of **7** to **1a**, intermediate **8** would ensue (observed by NMR, see SI). If *path a* is operative, then E2-type elimination should proceed in the absence of light. Yet, no anthracene (**9**) was observed in the dark after prolonged reaction times, even in the presence of a base (see SI for details). The same experiment provided information about *path b* and *c*: treatment of catalytic amounts of complex **1a** with **7** under blue light irradiation resulted in the formation of anthracene (**9**) in moderate yields. This is consistent with the generation of an in-cage radical pair upon irradiation, thus leaving a weak hydrogen atom [BDE_C–H_(9,10-dihydro-9-anthryl) = 42.5 kcal mol^–1^]. Differentiation between *path b* and *c* is experimentally challenging; hence, we recurred to theoretical analysis to discriminate between both. Direct oxidation of **2u′** followed by deprotonation—commonly proposed in radical C–H trifluoromethylation of arenes—is highly endergonic in this system, according to the calculated redox potential values [**2u′**, *E*_1/2_ (**2u′**^+^/**2u′**) = −0.1 vs SCE; **Int I**, *E*_cal_ (**Int-Ia/1a**+**Cl**^**–**^) = −0.6 vs SCE].^5d^ Yet, modeling the HAT between **2u′** and **Int-Ia** reveals that the HAT proceeds through **TS2a** with a low kinetic barrier (**TS2a**, Δ*G*^‡^ = +2.9 kcal mol^–1^). On thermodynamic grounds, the C–H abstraction in **2u′**[BDE_C–H_(**2u′**) = 25.2 kcal mol^–1^] en route to the Bi–H bond [BDE_Bi–H_(**Int-IIa**) = 44.6 kcal mol^–1^] is favorable,^12d,13d,15,23−25^ thus favoring *path c* over *path b*. It is important to mention that when monitoring the trifluoromethylation of **2a** by ^1^H and ^19^F NMR spectroscopy, **5a** is the dominant Bi species observed at the beginning of the reaction. Yet, in the absence of a base, gradual decomposition of **5a** leads to protodemetalated ligand and unidentifiable Bi/OSOCF_3_ species. Monitoring the same reaction in the presence of a base (2,6-lutidine) led to constant concentration of **5a**, indicating the important role of the base in preventing catalyst decomposition through C–Bi protonolysis (see SI for details). Additionally, we measured a quantum yield (**Φ**) of 0.19 using dimethyluracil (**2q**), thus pointing to the absence of a radical chain mechanism.^26^

**Figure 4:**
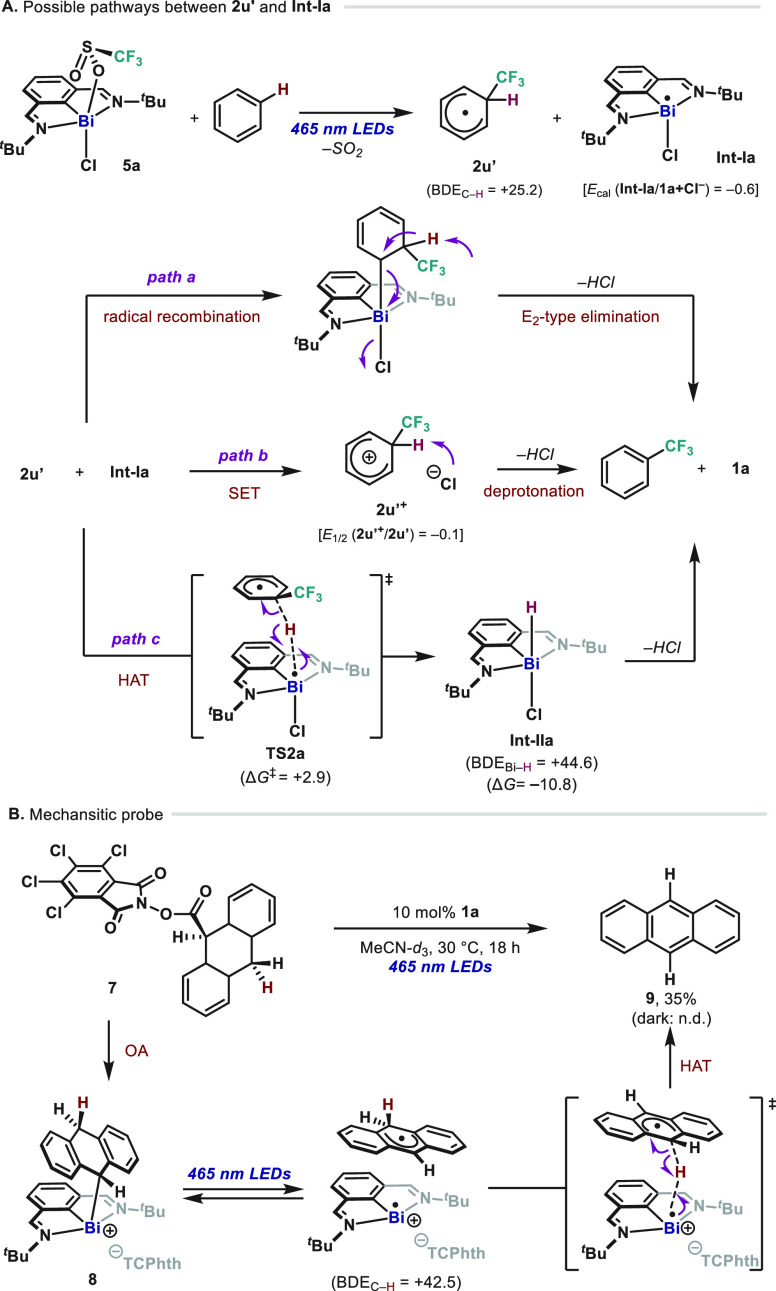
(A) Three possible pathways between **2u′** and **Int-Ia**. TCPhth = tetrachlorophthalimide. BDE = bond dissociation energy (kcal mol^–1^), *E* (V vs SCE) (B) Probing the catalytic rearomatization through HAT.

On the foregoing mechanistic studies, a proposed catalytic cycle is depicted in Figure 5. The cycle begins with the radical oxidative addition of CF_3_SO_2_Cl (**3**) to Bi(I) (**1**) to afford Bi(III) intermediate **5**. The resultant Bi(III) complex **5** undergoes a light-mediated Bi–O bond homolysis through an LLCT process to release the CF_3_ radical and SO_2_. At this point, addition of the CF_3_ radical to the (hetero)arene generates **2u′** and **Int-I**.^13g,13i,27^ Finally, the Bi(II) intermediate **Int-I** abstracts the hydrogen atom from radical intermediate **2u′** to rearomatize and release hydrogen chloride through ligand coupling or deprotonation facilitated by base to close the cycle.

**Figure 5:**
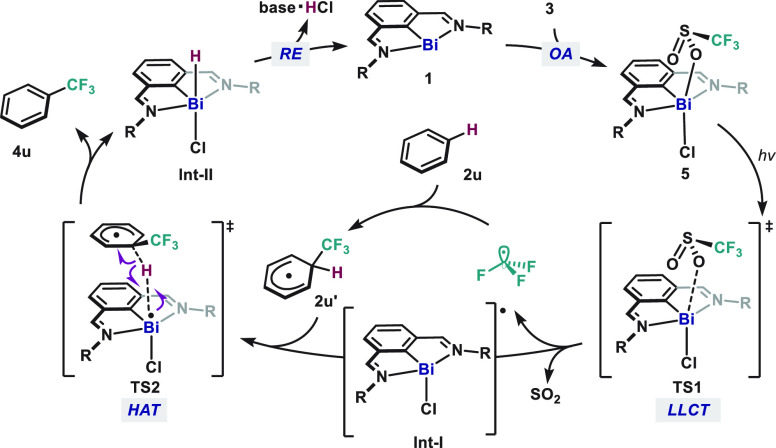
Proposed catalytic cycle of direct C–H radical trifluoromethylation of (hetero)arenes via open-shell bismuth redox cycle.

In summary, engaging photoinduced bond homolysis with Bi complexes unlocks direct C–H radical trifluoromethylation of (hetero)arenes through distinct elementary steps for main group compounds. Spectroscopic and computational experiments support a light-induced homolysis of the Bi–O bond, eventually leading to a CF_3_ radical. Moreover, a combined experimental and computational analysis revealed that the Bi(II) radical can abstract the hydrogen atom from the relatively weak C–H bond to rearomatize and deliver the trifluoromethylated product. These findings support a distinct low-valent Bi radical redox cycle and present further opportunities for Bi-catalyzed reactions involving open-shell Bi(II) radical species.

## Supporting Information Available

Experimental procedures and analytical data (^1^H, ^13^C and ^19^F NMR, HRMS, X-ray crystallographic details, and Cartesian coordinates). (PDF)
