# Supplementary material for: Bi-Catalyzed Trifluoromethylation of C(sp2)–H Bonds under Light
Source: J Am Chem Soc. 2023 Nov 14;145(47):25538–44. doi: 10.1021/jacs.3c10333 (PMC10690797; doi:10.1021/jacs.3c10333)

Supporting Information

Bi-catalyzed Trifluoromethylation of $C(sp^2)$–H Bonds under Light

Takuya Tsuruta, Davide Spinnato, Hye Won Moon, Markus Leutzsch and Josep Cornella*

Max-Planck-Institut für Kohlenforschung, Kaiser-Wilhelm-Platz 1, 45470, Mülheim an der Ruhr, Germany.

cornella@kofo.mpg.de

Table of Contents

1. General Information 4

2. General Experimental Procedure..5

2.1. Procedure for the direct C–H trifluoromethylation of (hetero)arenes 5

3. Substrate Preparation Procedure 6

3.1. Preparation of oxidative addition complex 6

3.2. Preparation of **1c** 7

3.2.1. Preparation of **S1** 7

3.2.2. Preparation of Bi(III) complex **S2** 7

3.2.3. Preparation of Bi(I) complex **1c** 8

3.3. Preparation of *tert*-butyl 1H-pyrrole-1-carboxylate (**2l**) 9

3.4. Preparation of 1-benzyl-3-methyl-1H-indole (**2n**) 9

3.5. Preparation of 4,5,6,7-tetrachloro-1,3-dioxoisoindolin-2-yl 9,10-dihydroanthracene-9-carboxylate **7** 10

4. Optimization of the trifluoromethylation using **5a**...11

4.1. Solvent screening 11

4.2. Additive screening...12

4.3. Optimization of the catalytic trifluoromethylation...13

4.4. Reaction conditions screening for electron-deficient substrates 14

5. Product Characterization 15

5.1. Characterization of direct C–H trifluoromethylation products **4** 15

5.2. Characterization of direct C–H trifluoromethylation of natural products. 25

6. X-ray data 30

6.1. Single crystal structure analysis of **5a** 30

7. Experimental Mechanistic Studies 36

7.1. Proposed Mechanism 36

7.2. CF_3 radical trapping with TEMPO..37

7.3. The reactivity of **5a** toward C–H trifluoromethylation of **2a** 38

7.4. Quantum yield measurement...39

7.5. Aromatization through HAT 40

7.7. Kinetic Analysis 43
7.7.1. Kinetic Analysis without base..43
7.7.2. Kinetic Analysis with bases 49
7.7.3. Light On/Off experiment...51
7.8. Potential Reactivity of Low-valent Bismuth(I) (**1a**) toward Chloroform 52
7.8.1. The reaction of **1a** with chloroform 52
7.8.2. The reaction of **1a** with mixture of **3** and chloroform....................................53
8. Computational Mechanistic Studies..55
8.1. Computational details...55
8.2. Time-dependent density-functional theory (TD-DFT) calculations 56
8.3. Computational Study of Re-aromatization Process via HAT 58
8.4. XYZ coordinates of DFT optimized structures..59
9. References 69
10. NMR Spectrum...72

1. General Information

Unless otherwise stated, all manipulations were performed under argon using standard Schlenk line techniques or in an MBraun argon-filled glove box.

Instruments: Flash chromatography: Merck silica gel 60 (40-63 μm). ESI-MS: ESQ 3000 (Bruker). Accurate mass determinations: Bruker APEX III FT-MS (7 T magnet) or MAT 95 (Finnigan). Melting points were measured with an EZ-Melt Automated Melting Point Apparatus from Standford Research Systems.

NMR data were recorded on a Bruker AVIII HD 300 MHz, Bruker AVIII HD 400 MHz, Bruker AVIII 500 MHz or Bruker AVNeo 600 MHz NMR spectrometer. ^{1}H and ^{13}C chemical shifts are reported relative to the solvent residual peaks as an internal reference. For ^{1}H NMR the following residual proton peaks of the deuterated solvents were used: $CDCl_3$, δ 7.260; THF-d_8, δ 3.580, CD_3CN, δ 1.940. For ^{13}C NMR: $CDCl_3$, δ 77.16; THF-d_8, δ 67.57; CD_3CN, δ 1.32. ^{13}C spectra were acquired with broadband ^{1}H decoupling unless mentioned otherwise. ^{19}F NMR spectra measured at 282 MHz were generally acquired with broadband ^{1}H decoupling. ^{19}F NMR data measured at 585 MHz was generally measured without ^{1}H decoupling. UV-Vis absorption spectra were recorded on a Cary 6000i UV-Vis-NIR Spectrophotometer.

Chemicals: $CHCl_3$, $CDCl_3$, THF, THF-d_8, *n*-pentane, CD_3CN and other solvents were distilled from the proper drying agents and stored over 3 Å or 4 Å molecular sieves under argon prior to use. 3 Å or 4 Å molecular sieves were activated at 200 °C under high vacuum (1 ×10−4bar) for 3 days. $CHCl_3$ and $CDCl_3$ were degassed through freeze-pump-thaw cycle. Anhydrous $BiCl_3$ (99.9%, trace metal basis) was purchased from Alfa Aesar. Cobaltocene (min. 98%) was purchased from Strem Chemicals and stored in the freezer of the glove box prior to use. *N*,*C*,*N*-pincer Bi(I) complexes **1a** and **1b** were prepared by the reported method.[1–3] Unless otherwise noted, all reagents were obtained from commercial suppliers and used without further purification.

2. General Experimental Procedure

2.1. Procedure for the direct C–H trifluoromethylation of (hetero)arenes.

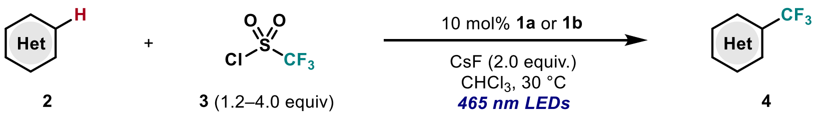

In an argon-filled glovebox, **1a** or **1b** (10 mol%), **2** (0.20 mmol), and cesium fluoride (0.40 mmol, 2.0 equiv) were placed in an oven-dried reaction vial, and the reaction vial was sealed with a screw cap containing a Teflon^{TM}-coated rubber septum. The vial was taken out of the glovebox and $CHCl_3$ (0.1 mL, 1.0 M, and degassed and dried) was added, and then **3** (0.24–0.80 mmol, 1.2–4.0 equiv) was added by syringe immediately. The vial was sealed with parafilm and placed it into a 465 nm blue LEDs (LEDXON® 24 V LED band) or white LEDs (LUMOtech® LED light 24 V) reactor with a cooling fun to keep the temperature 30 °C. After the reaction was completed, the reaction mixture was concentrated under reduced pressure by a rotary evaporator. The crude material was purified by flash column chromatographu using Merck silica gel 60 (40–63 µm) or by preparative TLC (Silica gel 60 F254, 1 mm, 20x20 cm, Sigma-Aldrich) to give the corresponding product.

Note: In the case that the substrate is liquid, it was added to the reaction vial by syringe after adding **3**.

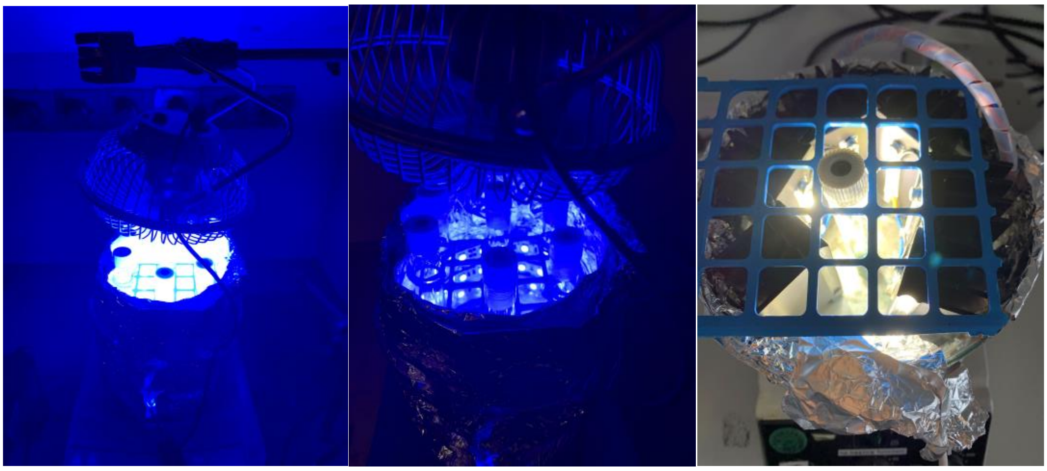

Figure S1. Light Set-up: blue LEDs (left and middle) and white LED (right)

3. Substrate Preparation Procedure

3.1. Preparation of oxidative addition complex **5a**

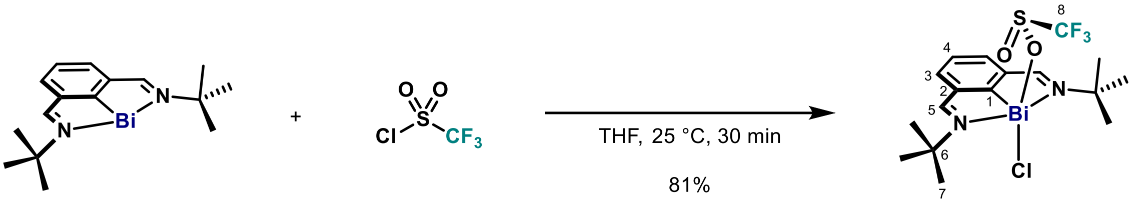

1a, 0.20 mmol + **3**, 1.2 equiv → **5a**

In an argon-filled glove box, **1a** (90.5 mg, 0.200 mmol) was placed in an oven-dried 100 mL Schlenk flask. The schlenk flask was taken out of the glovebox and THF (anhydrous) was added. **3** (26 µL, 0.24 mmol, 1.2 equiv) was added dropwise to this suspension under stirring at 25 °C. The resulting orange mixture was stirred for 30 minutes. The reaction mixture was concentrated under reduced pressure. The obtained yellow solid was washed with pentane for five times and dried to afford the product in 81% yield (10 mg, 0.16 mmol) as a pale yellow solid. Single crystals of **5a** suitable for X-ray crystallographic analysis were obtained by slow diffusion of *n*-hexane into a THF of **5a** at 25 °C.

^{1}H NMR (300 MHz, THF-*d*$_8$) δ 9.83 (s, 2H, H-5), 8.26 (d, *J* = 7.5 Hz, 2H, H-3), 7.86 (t, *J* = 7.5 Hz, 1H, H-4), 1.55 (s, 18H, H-7).

^{13}C{^{1}H} NMR (151 MHz, THF-*d*$_8$) δ 212.6 (C-1), 168.9 (C-5), 150.9 (C-2), 136.4 (C-3), 129.9 (C-4), 125.9 (q, *J* = 351.6 Hz, C-8), 61.9 (C-6), 30.9 (C-7). (NOTE: The peak intensity of C-1 was too low because of the line broadening effects arising from neighbored from ^{209}Bi.)

^{19}F NMR (282 MHz, THF-*d*$_8$) δ –87.9.

HRMS (ESI): calc'd for $C_{17}H_{23}BiF_3N_2O_2S$ [M-Cl]$^+$: 585.12194, found: 585.12244.

calc'd for $C_{16}H_{23}BiN_2Cl$ [M-SO_2CF_3]$^+$: 487.13483, found: 487.13497.

UV-Vis measurement

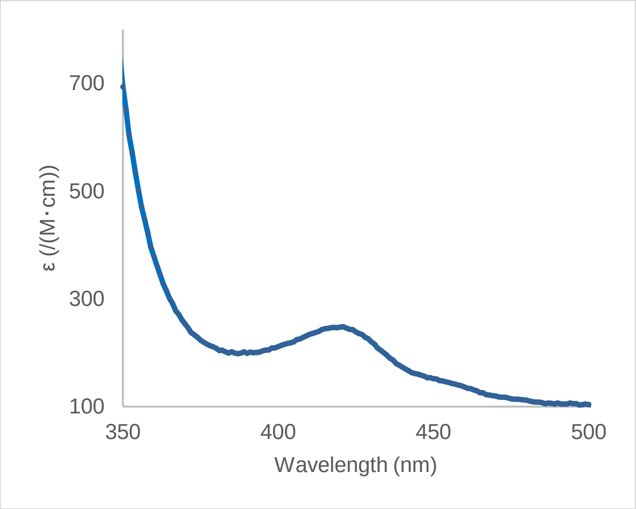

Figure S2. UV-Vis absorption spectrum of **5a** in $CHCl_3$. The sample was diluted with $CHCl_3$ to 0.20 mM and sealed in a cuvette (0.20 cm). **5a** displayed an absorption band centered at λ_{max} = 421 nm.

3.2. Preparation of **1c**

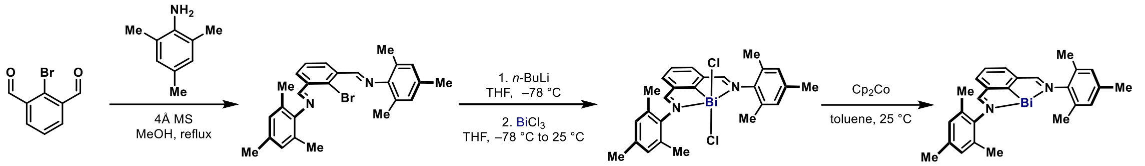

S1 S2 1c

3.2.1. Preparation of **S1**

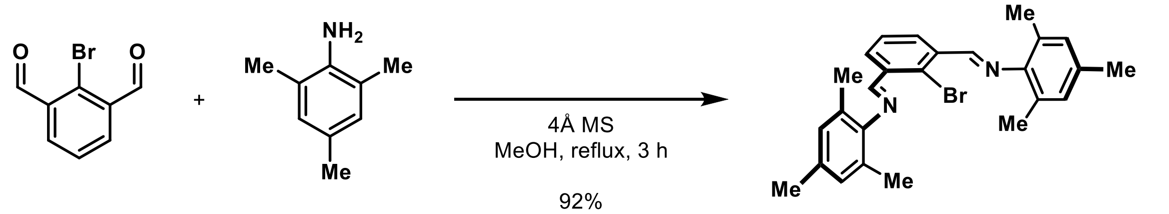

30 mmol 2.2 equiv **S1**

In a vacuum dried 100 mL round-bottomed flask with a reflux condenser, 2,4,6-trimethylaniline (8.9 g, 66 mmol, 2.2 equiv) was added dropwise to a solution of 2-bromoisophthalaldehyde (6.4 g, 30 mmol) and 4 Å MS in MeOH (60 mL) at 25 °C under argon atmosphere. The reaction mixture was allowed up to warm to at 70 °C (reflux) and stirred for 3 h. After cooling down, the reaction mixture was filtrated and filtrate was concentrated under reduce pressure to afford product **S1** in 92% yield (12.4 g, 3.75 mmol) as a pale-yellow solid.

^{1}H NMR (300 MHz, $CDCl_3$) δ 8.73 (s, 2H), 8.38 (d, *J* = 7.7 Hz, 2H), 7.55 (t, *J* = 7.7 Hz, 1H), 6.92 (s, 4H), 2.30 (s, 6H), 2.17 (s, 12H).

^{13}C{^{1}H} NMR (151 MHz, $CDCl_3$) δ 162.0, 148.5, 135.8, 133.7, 131.5, 129.0, 128.5, 128.0, 127.2, 20.9, 18.5.

HRMS (ESI): calc'd for $C_{26}H_{28}N_2Br$ $[M+H]^+$: 447.14304, found: 447.14304.

The spectral data matched with those reported in the literature.[4]

3.2.2. Preparation of Bi(III) complex **S2**

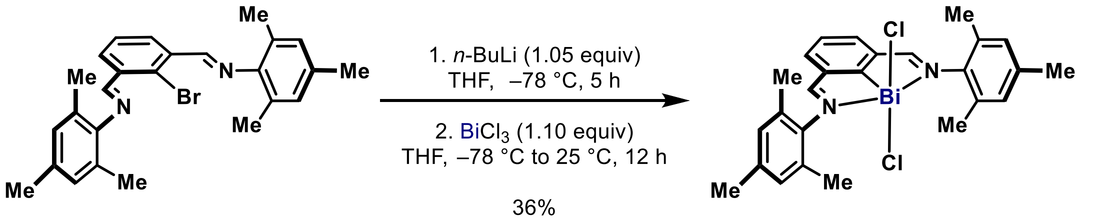

S1
20 mmol

S2

S2 was prepared according to literature procedure.[1] In a vacuum dried 250 mL round-bottomed flask, *n*-BuLi (2.50 M in hexane, 8.40 mL, 21.0 mmol, 1.05 equiv) was added dropwise to a dry ice/acetone-cooled solution of **S1** (6.4 g, 20 mmol) in dry THF under an argon atmosphere. The resulting dark brown solution was stirred at this temperature for 5 h. Inside an argon-filled glovebox, $BiCl_3$ (6.9 g, 22 mmol, 1.1 equiv) was placed in an oven-dried 100 mL Schlenk flask. The schlenk was taken out of the glovebox and THF (anhydrous, 20 mL) was added. The resulting mixture was cooled to –78 °C using a dry ice/acetone-cooled bath. The solution of **S1** was transferred via a cannula under argon atmosphere to a pre-cooled suspension of $BiCl_3$ and the resulting solution was stirred for 12 h at 25 °C. The work-up was carried out without the protection of argon.

The reaction mixture was evaporated to dryness, and washed with MTBE to afford the product in 36% yield (4.77 g, 7.19 mmol) as a pale-brown solid.

^{1}H NMR (300 MHz, $CDCl_3$) δ 9.59 (s, 2H), 8.42 (d, *J* = 7.6 Hz, 2H), 7.98 (t, *J* = 7.5 Hz, 1H), 6.95 (s, 4H), 2.41 (s, 12H), 2.30 (s, 6H).

^{13}C{^{1}H} NMR (151 MHz, $CDCl_3$) δ 218.5, 176.0, 146.6, 144.6, 138.1, 137.0, 130.6, 129.77, 129.76, 21.0, 19.9.

HRMS (ESI): calc'd for $C_{26}H_{27}BiN_2Cl$ $[M–Cl]^+$: 611.16613, found: 611.16584.

3.2.3. Preparation of Bi(I) complex **1c**

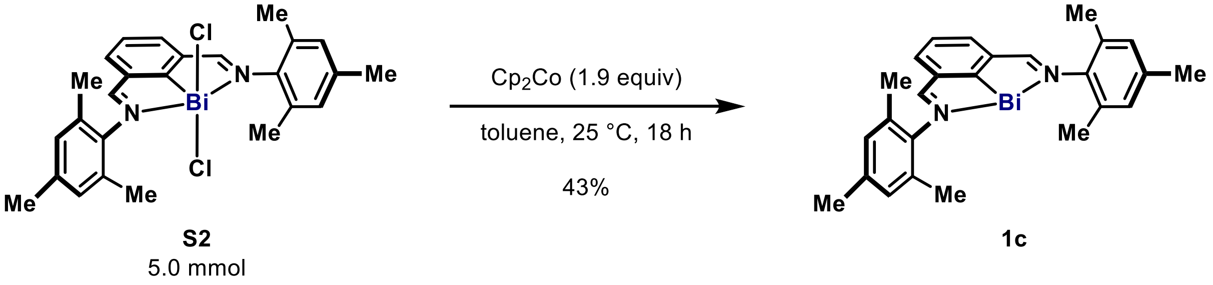

In an argon-filled glove box, **S2** (3.1 g, 5.0 mmol) and toluene (anhydrous and degassed, 50 mL) were added to a 100 mL Schlenk flask. At room temperature, cobaltocene (1.8 g, 9.5 mmol, 1.9 equiv.) was added portionwise to the suspension under rapid stirring. The resulting dark green mixture was stirred for an additional 18 h and the Schlenk was taken out of the glove box. Cobaltocenium chloride was filtered off under argon atmosphere. The filtrate was dried to afford bismuthinidene (**1c**) in 43% yield (1.25 g, 2.17 mmol) as a dark green solid.

^{1}H NMR (300 MHz, THF-d_8) δ 9.63 (s, 2H), 8.23 (d, *J* = 7.4 Hz, 2H), 7.18 (t, *J* = 7.4 Hz, 1H), 6.95– 6.94(m, 4H), 2.31 (s, 6H), 2.11 (s, 12H).

^{13}C{^{1}H} NMR (75 MHz, THF-d_8) δ 209.1, 171.8, 149.1, 146.8, 136.5, 135.2, 130.0, 129.6, 123.6, 21.0, 19.3

HRMS (EI): calc'd for $C_{26}H_{27}BiN_2$ $[M]^+$: 576.19727, found: 576.19769.

Notes: If a sticky solid is obtained after removing toluene solvent, dissolve the solid in pentane (anhydrous and degassed) to obtain a powder.

3.3. Preparation of *tert*-butyl 1H-pyrrole-1-carboxylate (**2l**)

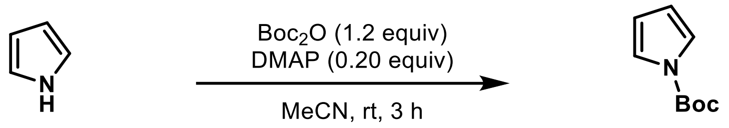

In a vacuum dried 100 mL round-bottomed flask, Boc_2O (2.6 g, 12 mmol, 1.2 equiv) and DMAP (244 mg, 0.200 equiv) were added to a well-stirred solution of pyrrole (671 mg, 694 µL, 10.0 mmol) in MeCN (10 mL) at 25 °C under argon atmosphere. After being stirred for 3 h, the reaction mixture was concentrated under reduced pressure. The obtained crude material was purified by column chromatography (SiO_2, Et_2O/pentane, 0:100→10:90) to afford the product in 98% yield (1.64 g, 9.80 mmol) as a colorless oil.

^{1}H NMR (300 MHz, $CDCl_3$) δ 7.26–7.23 (m, 2H), 6.24–6.21 (m, 2H), 1.61 (s, 9H).

^{13}C{^{1}H} NMR (75 MHz, $CDCl_3$) δ 149.0, 120.1, 111.9, 83.6, 28.1.

HRMS (EI): calc'd for $C_9H_{13}NO_2$ $[M]^+$: 167.094079, found: 167.094160.

The spectral data matched with those reported in the literature.[5]

3.4. Preparation of 1-benzyl-3-methyl-1H-indole (**2n**)

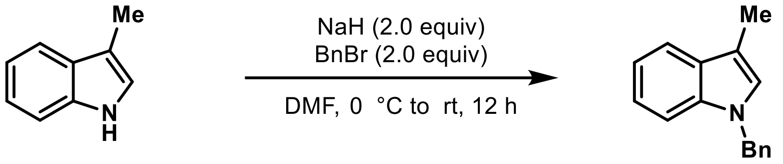

In a vacuum dried 100 mL round-bottomed flask, 3-methylindole (656 mg, 5.00 mmol) was added to a well-stirred solution of NaH (0.24g, 10 mmol, 2.0 equiv) in DMF (10 mL) at 0 °C under argon atmosphere. The reaction mixture was allowed up to warm to at 25 °C and stirred for 1 h. Then benzyl bromide (BnBr, 1.2 mL, 10 mmol, 2.0 equiv) was added dropwise. After being stirred for 12 h, the reaction mixture was carefully poured into water to quench and the aqueous phase was extracted with EtOAc three times. The combined organic layer was washed with brine, and dried over $MgSO_4$. After filtration, the solution was concentrated under reduce pressure. The obtained crude material was purified by column chromatography (SiO_2, EtOAc/hexane, 0:100→10:90) to afford the product in 75% yield (829 mg, 3.75 mmol) as a white solid.

^{1}H NMR (300 MHz, $CDCl_3$) δ 7.59 (ddd, J = 7.5, 1.4, 0.8 Hz, 1H), 7.33–7.23 (m, 4H), 7.21–7.08 (m, 4H), 6.90 (d, J = 1.1 Hz, 1H), 5.27 (s, 2H), 2.35 (d, J = 1.1 Hz, 3H).

^{13}C{^{1}H} NMR (151 MHz, $CDCl_3$) δ 138.0, 136.8, 129.0, 128.8, 127.6, 126.9, 126.0, 121.7, 119.2, 118.9, 111.0, 109.6, 49.9, 9.8.

HRMS (EI): calc'd for $C_{16}H_{15}N$ $[M]^+$: 221.119899, found: 221.120150

The spectral data matched with those reported in the literature.[6]

3.5. Preparation of 4,5,6,7-tetrachloro-1,3-dioxoisoindolin-2-yl 9,10-dihydroanthracene-9-carboxylate **7**

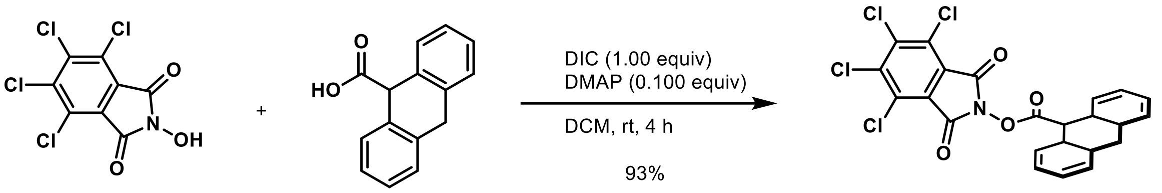

1.00 mmol 1.00 equiv **7**

7 was prepared according to literature procedures.[7] In a vacuum dried 100 mL round-bottomed flask, *N*,*N'*-diisopropylcarbodiimide (DIC, 126 mg, 155 μL) was added to well-stirred solution of *N*-hydroxytetrachlorophtalimide (301 mg, 1.00 mmol), 9,10-Dihydroanthracen-9-carboxylic acid (224 mg, 1.00 equiv) and DMAP (12.2 mg, 0.100 equiv) in anhydrous DCM (10 mL) at 25 °C under argon atmosphere. After being stirred for 4 h, the reaction mixture was concentrated under reduced pressure. The obtained crude material was washed with hexane and DCM for three times respectively and resulting crude material was purified by column chromatography (SiO_2, DCM) to afford the product in 93% yield (507 mg, 0.927 mmol) as a pale yellow solid.

^{1}H NMR (600 MHz, $CDCl_3$) δ 7.55–7.52 (m, 2H), 7.40–7.36 (m, 2H), 7.34 (td, *J* = 7.3, 2.0 Hz, 2H), 7.32 (tdd, *J* = 7.3, 2.1, 0.8 Hz, 2H), 5.35 (s, 1H), 4.31 (d, *J* = 18.4 Hz, 1H), 3.98 (d, *J* = 18.5 Hz, 1H).

^{13}C{^{1}H} NMR (151 MHz, $CDCl_3$) δ 167.6, 157.4, 141.2, 136.9, 131.5, 130.6, 128.6, 128.5, 128.4, 127.0, 124.8, 49.6, 35.5.

HRMS (ESI): calc'd for $C_{23}H_{11}NO_4Cl_4Na$ $[M+Na]^+$: 527.93344, found: 527.93377.

4. Optimization of the trifluoromethylation using 5a.

4.1. Solvent screening

Procedure: In a reaction vial, 1,3,5-trimethoxybenzene (**2a**, 0.050 mmol, 8.4 mg, 5.0 equiv) and **5a** (0.010 mmol, 6.2 mg) were placed. The vial was sealed with a screw cap containing a Teflon™-coated rubber septum and degassed by quickly alternating vacuum evacuation and argon backfill for three times. Then, degassed and dry solvent (0.50 mL, 0.020 M) was added to the vial. Then the vial was sealed with parafilm, and it was placed into the blue LED reactor. After the reaction mixture stirred for 18 h, it was passed through a short silica gel column eluting with DCM and the resulting solution was concentrated under reduced pressure by a rotary evaporator. Subsequently, 4-fluorotoluene (29.2 mg, 24.5 μL, 0.200 mmol, 3.00 equiv.) was added as an internal standard. The reaction mixture was diluted with $CDCl_3$, and the yield was determined by quantitative ^{19}F NMR.

Table S1. Solvent screening.

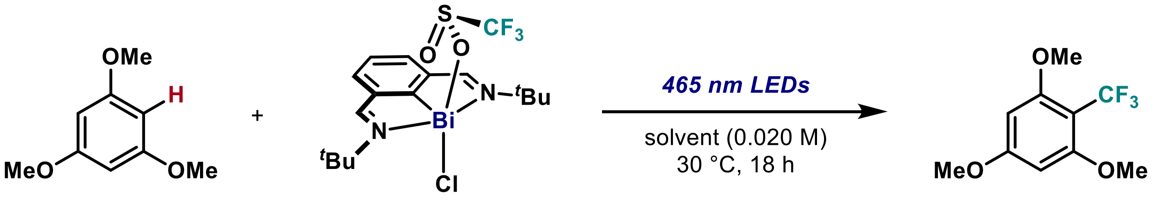

2a 5a 4a

Entry	solvent	yield of **4a** (%)[a]
1	THF	2
2	MeCN	8
3	DMF	12
4	DMA	27
5	DMSO	12
6	NMP	14
7	DCM	17
8	DCE	9
9	CCl_4	15
10	$CHCl_3$	53
11[b]	$CHCl_3$	n.d.
12	$CHCl_3$ (0.10 M)	71
13	$CHCl_3$ (0.0050 M)	23
14[c]	$CHCl_3$ (0.10 M)	81

[a]Yield was determined by quantitative ^{19}F NMR with 4-fluorotoluene as an internal standard. [b]The reaction was performed under dark condition. [c]White LED was used.

4.2. Additive screening

Procedure: Inside an argon-filled glovebox, 1,3,5-trimethoxybenzene (**2a**, 0.050 mmol, 8.4 mg, 5.0 equiv), **5a** (0.010 mmol, 6.2 mg), and additives (0.020 mmol, 2.0 equiv) were placed in an oven-dried vial, and it was sealed with a screw cap containing a Teflon™-coated rubber septum. The vial was taken out of the glovebox and degassed dried chloroform (0.50 mL, 0.020 M) was added. Then the vial was sealed with parafilm, and it was placed into the blue LED irradiation set up. After 18 h, it was passed through a short silica gel column eluting with DCM and the resulting solution was concentrated under reduced pressure by a rotary evaporator. Subsequently, 4-fluorotoluene (29.2 mg, 24.5 μL, 0.200 mmol, 3.00 equiv.) was added as an internal standard. The reaction mixture was diluted with $CDCl_3$, and the yield was determined by quantitative ^{19}F NMR.

Table S2. Additive screening.

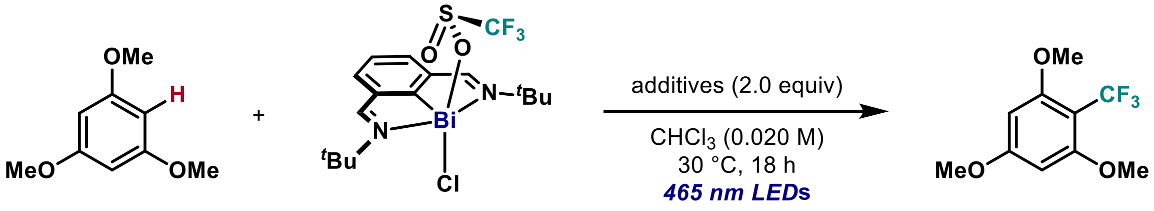

2a **5a** **4a**

Entry	additive	yield of **4a** (%)^{a}
1	—	53
2	KF	49
3	K_3PO_4	42
4	K_2HPO_4	41
5	K_2CO_3	45
6	KO*ᵗ*Bu	26
7	LiOTf	42
8	N^iPr_2Et	25
9	2,6-lutidine	50
10	4Å Molecular sieves	21

^{a}Yield was determined by quantitative ^{19}F NMR with 4-fluorotoluene as an internal standard.

4.3. Optimization of the catalytic trifluoromethylation

Procedure: Inside an argon-filled glovebox, 1,3,5-trimethoxybenzen (**2a**, 0.050 mmol, 8.4 mg), and *N*,*C*,*N*-pincer bismuth complex **1** (10 mol%, 0.0050 mmol) were placed in an oven-dried reaction vial, and the reaction vial was sealed with a screw cap containing a Teflon[TM]-coated rubber septum. The vial was taken out of the glovebox and solvent (0.050 mL, 0.10 M) was added. Then, **3** (11 µL, 0.10 mmol, 2.0 equiv) was added immediately and the vial was placed in the irradiation set-up. After the reaction mixture stirred for 18 h, the reaction mixture was diluted with $CDCl_3$, and the yield was determined by ^{19}F NMR using benzotrifluoride (6.2 µL, 0.050 mmol) as an internal standard.

Table S3. Reaction condition screening.

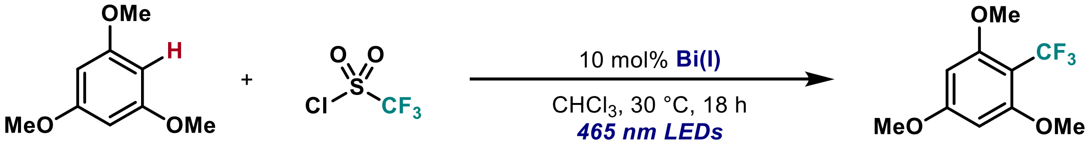

2a **3** (2.0 equiv.) **4a**

Entry	Bi(I)	deviation from above	yield of **4a** (%)
1	**1a**	none	79
2[a]	**1b**	none	89
3	**1c**	none	44
4	**1a**	DCM instead of $CHCl_3$	61
5	**1a**	DCE instead of $CHCl_3$	65
6	**1a**	MeCN instead of $CHCl_3$	53
7	**1a**	DMA instead of $CHCl_3$	11
8	**1a**	NMP instead of $CHCl_3$	31
9	**1a**	CsF (2.0 equiv.)	70
10	**1a**	K_2CO_3 (2.0 equiv.)	73
11	**1a**	in the dark	n.d.

[a]White LED was used.

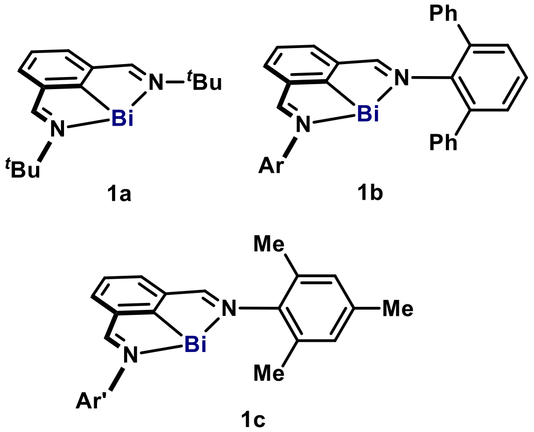

1a **1b** **1c**

4.4. Reaction conditions screening for electron-deficient substrates

Procedure: Inside an argon-filled glovebox, 2,6-dimethylpyrazine (**2i**, 0.050 mmol, 5.4 mg) and *N*,*C*,*N*-pincer bismuth complex **1** (10 mol%, 0.0050 mmol) were placed in an oven-dried reaction vial, and the reaction vial was sealed with a screw cap containing a Teflon^{TM}-coated rubber septum. The vial was taken out of the glovebox and $CHCl_3$ (0.050 mL, 1.0 M) was added. Then, **3** (11 µL, 0.10 mmol, 2.0 equiv) was added immediately and the vial is placed in the irradiation set-up. After the reaction mixture stirred for 18 h, the reaction mixture was diluted with $CDCl_3$, and the yield was determined by ^{1}H NMR using trichloroethylene (9.0 µL, 0.10 mmol) or ^{19}F NMR using benzotrifluoride (6.2 µL, 0.050 mmol) as an internal standard.

Table S4. Reaction screening for electron-deficient substrate.

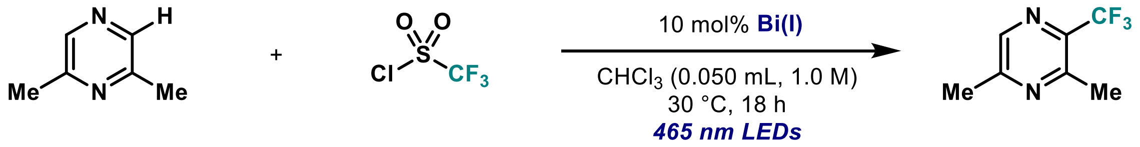

2i + **3**, 2.0 equiv → **4i**

Entry	Bi(I)	deviations from initial conditions	yield of **4i** (%)
1	**1b**	—	16
2	**1a**	—	18
3	**1a**	addition of CsF (2.0 equiv)	30
4	**1a**	addition of NaF (2.0 equiv)	34
5	**1a**	addition of NaI (2.0 equiv)	n.d.
6	**1a**	addition of MgF_2 (2.0 equiv)	30
7	**1a**	addition of CsF (2.0 equiv), $CHCl_3$ (0.40 M)	33
8	**1a**	addition of CsF (2.0 equiv), $CHCl_3$ (0.40 M) 4.0 equiv of **3**	43
9	**1a**	addition of CsF (2.0 equiv), THF instead of $CHCl_3$	<5
10	**1a**	addition of CsF (2.0 equiv), MeCN instead of $CHCl_3$	<5
11	**1a**	addition of CsF (2.0 equiv), DCM instead of $CHCl_3$	<5
12	**1a**	addition of KO^{t}Bu (2.0 equiv), 4.0 equiv of **3**	18
13	**1a**	addition of KHMDS (2.0 equiv), 4.0 equiv of **3**	11
14	**1a**	addition of K_2CO_3 (2.0 equiv), 4.0 equiv of **3**	58
15	**1a**	addition of K_2CO_3 (2.0 equiv), 4.0 equiv of **3** MeCN instead of $CHCl_3$	27

5. Product Characterization

5.1. Characterization of direct C–H trifluoromethylation products **4**

1,3,5-trimethoxy-2-(trifluoromethyl)benzene (4a)

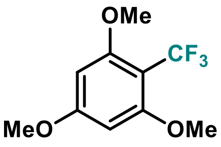

Following the general procedure, the reaction was performed with 1,3,5-trimethoxybenzene (**2a**, 34 mg, 0.20 mmol), **3** (43 µL, 0.4 mmol, 2.0 equiv) and **1b** (16 mg, 0.010 mmol, 10 mol%) for 3 h under white light irradiation. The crude mixture was purified by flash column chromatography on silica gel (hexane/EtOAc = 90:10) to afford the product in 82% (38.6 mg, 0.160 mmol) as a white solid.

^{1}H NMR (600 MHz, $CDCl_3$) δ 6.13 (s, 2H), 3.84 (s, 9H).

^{13}C{^{1}H} NMR (151 MHz, $CDCl_3$) δ 163.6, 160.6 (q, *J* = 1.4 Hz), 124.5 (q, *J* = 273.4 Hz), 100.6 (q, *J* = 30.0 Hz), 91.4, 56.4, 55.5.

^{19}F NMR (282 MHz, $CDCl_3$) δ –54.2.

HRMS (EI): calc'd for $C_{10}H_{11}O_3F_3$ $[M]^+$: 236.065480, found: 236.065620.

The spectral data matched with those reported in the literature.[8]

3,4,5-trimethoxy-2-(trifluoromethyl)benzaldehyde (4b)

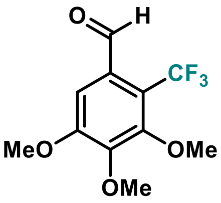

Following the general procedure, the reaction was performed with 3,4,5-trimethoxybenzaldehyde (**2b**, 39 mg, 0.20 mmol), **3** (43 µL, 0.40 mmol, 2.0 equiv) and **1b** (16 mg, 0.020 mmol, 10 mol%) for 18 h under white light irradiation. The crude mixture was purified by preparative TLC (hexane/EtOAc = 80:20) to afford the product in 50% (26.3 mg, 0.100 mmol) as a yellow oil.

^{1}H NMR (300 MHz, $CDCl_3$) δ 10.33 (q, *J* = 2.5 Hz, 1H), 7.34 (s, 1H), 3.95 (s, 6H), 3.94 (s, 3H).

^{13}C{^{1}H} NMR (151 MHz, $CDCl_3$) δ 189.3 (q, *J* = 6.0 Hz), 155.9, 152.9 (q, *J* = 2.2 Hz), 147.5, 131.0, 124.3 (q, *J* = 275.1 Hz), 118.1 (q, *J* = 31.2 Hz), 107.4, 62.2, 61.2, 56.4.

^{19}F NMR (282 MHz, $CDCl_3$) δ –51.1.

HRMS (EI): calc'd for $C_{11}H_{11}O_4F_3$ $[M]^+$: 264.060395, found: 264.060560.

The spectral data matched with those reported in the literature.[9]

1-(3,4,5-trimethoxy-2-(trifluoromethyl)phenyl)ethan-1-one (4c)

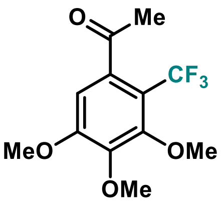

Following the general procedure, the reaction was performed with 3′,4′,5′-trimethoxyacetophenone (**2c**, 42 mg, 0.20 mmol), **3** (64 µL, 0.60 mmol, 3.0 equiv) and **1b** (16 mg, 0.020 mmol, 10 mol%) for 18 h under white light irradiation. The crude mixture was purified by preparative TLC (hexane/EtOAc/DCM = 60:30:10) to afford the product in 61% (34 mg, 0.12 mmol) as a yellow oil.

^{1}H NMR (300 MHz, $CDCl_3$) δ δ 6.49 (s, 1H), 3.96 (s, 3H), 3.90 (s, 3H), 3.89 (s, 3H), 2.49 (q, *J* = 1.2 Hz, 3H).

^{13}C{^{1}H} NMR (151 MHz, $CDCl_3$) δ 202.6, 156.4, 153.0 (q, *J* = 2.0 Hz), 143.6, 137.9 (q, *J* = 2.5 Hz), 123.6 (q, *J* = 273.2 Hz), 113.1 (q, *J* = 31.0 Hz), 104.6, 62.0, 61.0, 56.4, 31.6 (q, *J* = 3.1 Hz).

^{19}F NMR (282 MHz, $CDCl_3$) δ –54.9.

HRMS (EI): calc'd for $C_{12}H_{13}O_4F_3$ $[M]^+$: 278.075910, found: 278.076045.

The spectral data matched with those reported in the literature.[8]

1,2,3-trimethoxy-5-methyl-4-(trifluoromethyl)benzene (4d)

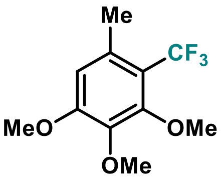

Following the general procedure, the reaction was performed with 3,4,5-trimethoxytoluene (**2d**, 36 mg, 0.20 mmol), **3** (43 µL, 0.40 mmol, 2.0 equiv), K_2CO_3 (56 mg, 0.40 mmol, 0.20 equiv), and **1b** (15.9 mg, 0.020 mmol, 10 mol%) for 18 h under blue light irradiation. The crude mixture was purified by preparative TLC (100% toluene) to afford the product in 66% (33.2 mg, 0.130 mmol) as a colorless oil.

^{1}H NMR (300 MHz, $CDCl_3$) δ 6.49 (s, 1H), 3.91 (s, 3H), 3.88 (s, 3H), 3.85 (s, 3H), 2.42 (qd, *J* = 3.5, 0.6 Hz, 3H).

^{13}C{^{1}H} NMR (151 MHz, $CDCl_3$) δ 155.3, 153.5 (q, *J* = 1.9 Hz), 141.1, 133.3 (q, *J* = 1.4 Hz), 125.0 (q, *J* = 274.2 Hz), 115.6 (q, *J* = 28.9 Hz), 110.8, 61.9, 61.0, 56.1, 21.7 (q, *J* = 4.2 Hz).

^{19}F NMR (282 MHz, $CDCl_3$) δ –54.3.

HRMS (EI): calc'd for $C_{11}H_{13}O_3F_3$ $[M]^+$: 250.081131, found: 250.081420.

The spectral data matched with those reported in the literature.[9]

2-bromo-1,3,5-trimethoxy-4-(trifluoromethyl)benzene (4e)

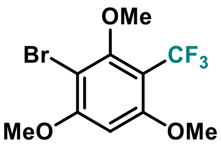

Following the general procedure, the reaction was performed with 1-bromo-2,4,6-trimethoxybenzene (**2e**, 49 mg, 0.20 mmol), **3** (85 μL, 0.80 mmol, 4.0 equiv), K_2CO_3 (56 mg, 0.40 mmol, 2.0 equiv), and **1a** (9.0 mg, 0.020 mmol, 10 mol%) for 6 h. The crude mixture was purified by preparative TLC hexane/EtOAc = 80:20) to afford the product in 76% (48.2 mg, 0.150 mmol) as a white solid.

^{1}H NMR (300 MHz, $CDCl_3$) δ δ 6.35 (s, 1H), 3.95 (s, 3H), 3.90 (s, 3H), 3.86 (s, 3H).

^{13}C{^{1}H} NMR (151 MHz, $CDCl_3$) δ 160.0, 159.3, 157.8, 123.6 (q, J = 273.9 Hz), 107.0 (q, J = 30.0 Hz), 99.9, 93.2, 62.5, 56.7, 56.6.

^{19}F NMR (282 MHz, $CDCl_3$) δ –55.6.

HRMS (EI): calc'd for $C_{10}H_{10}O_3F_3Br$ $[M]^+$: 313.976006, found: 313.976320.

The spectral data matched with those reported in the literature.[8]

1,3,5-trimethyl-2-(trifluoromethyl)benzene (4f)

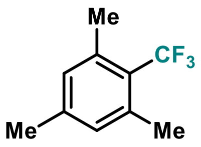

Following the general procedure, the reaction was performed with mesitylene (**2f**, 56 µL, 0.40 mmol, 2.0 equiv), **3** (21 µL, 0.20 mmol), K_2CO_3 (55 mg, 0.40 mmol, 2.0 equiv), and **1b** (15.9 mg, 0.020 mmol, 10 mol%) for 18 h under blue light irradiation. The reaction mixture was diluted with $CDCl_3$, and the yield was determined by ^{19}F NMR (50% yield) using benzotrifluoride (25 µL, 0.20 mmol) as an internal standard.

^{19}F NMR (282 MHz, $CDCl_3$) δ –53.7.

HRMS (EI): calc'd for $C_{10}H_{11}F_3$ $[M]^+$: 188.080735, found: 188.080970.

The spectral data matched with those reported in the literature.[10]

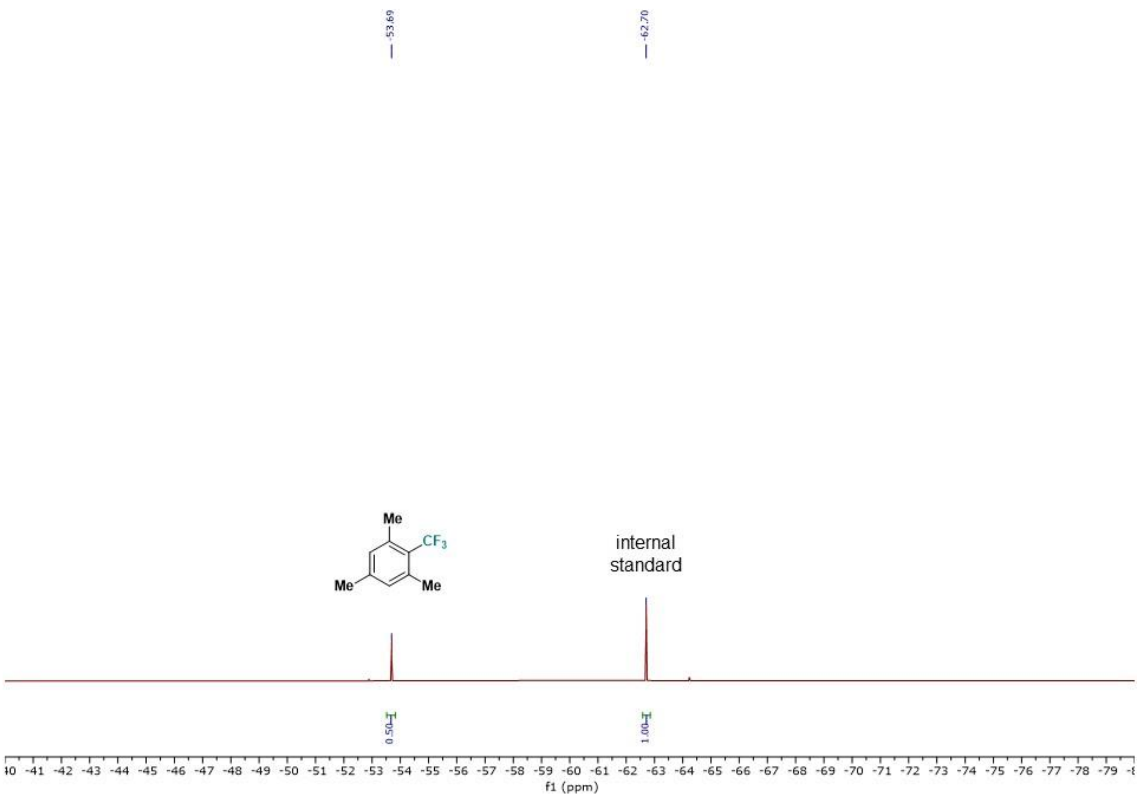

Figure S3. Crude NMR yield of **4f** using benzotrifluoride as internal standard.

2,4,6-trimethoxy-5-(trifluoromethyl)pyrimidine (4g)

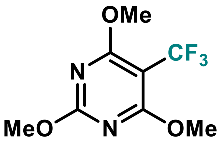

Following the general procedure, the reaction was performed with 2,4,6-trimethoxypyrimidine (**2g**, 34 mg, 0.20 mmol), **3** (85 µL, 0.80 mmol, 4.0 equiv), CsF (61 mg, 0.20 mmol, 2.0 equiv) and **1b** (16 mg, 0.020 mmol, 10 mol%) for 18 h under blue light irradiation. The crude mixture was purified by preparative TLC (toluene = 100%) to afford the product in 76% (36.2 mg, 0.150 mmol) as white solid.

^{1}H NMR (300 MHz, $CDCl_3$) δ 4.02 (s, 6H), 4.00 (s, 3H).

^{13}C{^{1}H} NMR (151 MHz, $CDCl_3$) δ 169.9, 165.1, 123.6 (q, J = 271.3 Hz), 89.5 (q, J = 34.2 Hz), 55.3, 55.0.

^{19}F NMR (282 MHz, $CDCl_3$) δ –56.0.

HRMS (EI): calc'd for $C_8H_9N_2O_3F_3$ $[M]^+$: 238.055978, found: 238.056040.

The spectral data matched with those reported in the literature.[8]

2,6-dimethoxy-3-(trifluoromethyl)pyridine (4h)

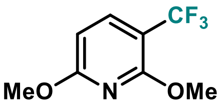

Following the general procedure, the reaction was performed with 2,6-dimethoxypyridine (**2h**, 19 µL, 0.20 mmol), **3** (85 µL, 0.80 mmol, 4.0 equiv), CsF (61 mg, 0.20 mmol, 2.0 equiv) and **1b** (16 mg, 0.020 mmol, 10 mol%) for 18 h under blue light irradiation. The reaction mixture was diluted with $CDCl_3$, and the yield was determined by ^{19}F NMR (68% yield) using benzotrifluoride (25 µL, 0.20 mmol) as an internal standard. Although double trifluoromethylated by-product (23%) was observed in 23%, it could be separated from **4h** by column chromatography. The crude mixture was purified by preparative TLC (pentene/Et_2O = 70:30) to afford the product as pale yellow solid.

^{1}H NMR (300 MHz, $CDCl_3$) δ 7.72 (dd, *J* = 8.3, 0.7 Hz, 1H), 6.33 (dd, *J* = 8.3, 0.7 Hz, 1H), 4.02 (s, 3H), 3.96 (s, 3H).

^{13}C{^{1}H} NMR (151 MHz, $CDCl_3$) δ 165.3, 160.6 (q, *J* = 1.8 Hz), 139.0 (q, *J* = 4.5 Hz), 123.9 (q, *J* = 270.1 Hz), 104.6 (q, *J* = 33.4 Hz), 100.9, 54.03, 53.99.

^{19}F NMR (282 MHz, $CDCl_3$) δ –61.9.

HRMS (EI): calc'd for $C_8H_8N_1O_2F_3$ $[M]^+$: 207.050164, found: 207.050400.

The spectral data matched with those reported in the literature.[9]

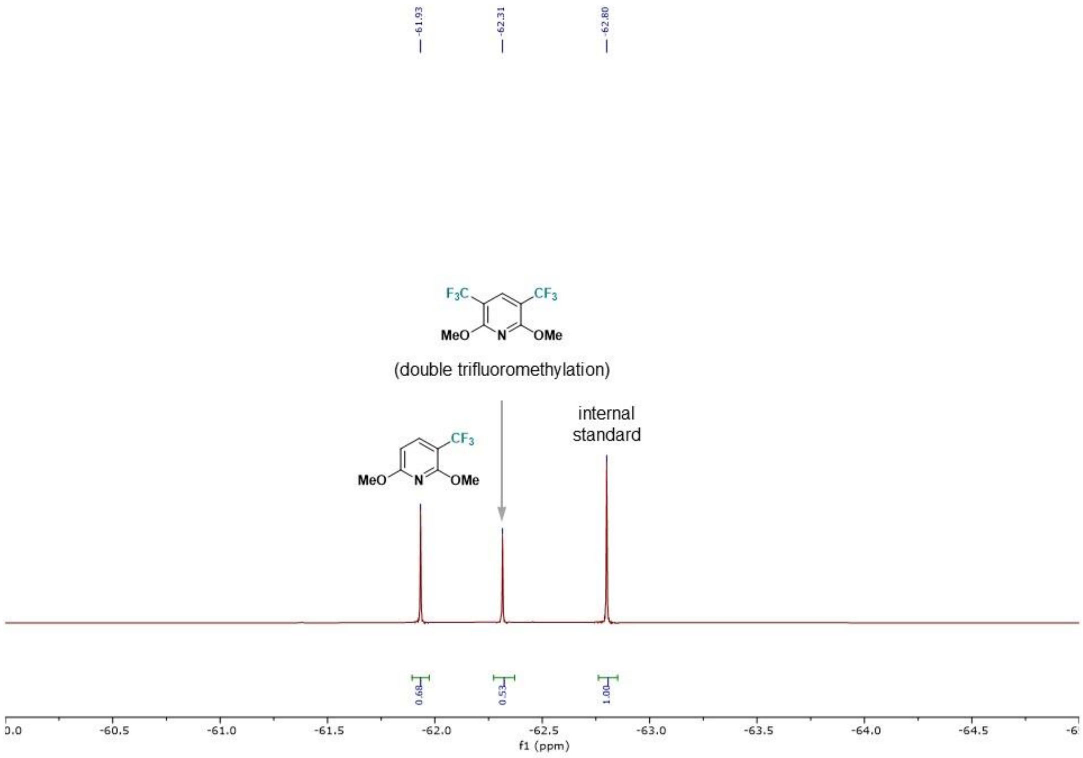

Figure S4. Crude NMR yield of **4h** using benzotrifluoride as internal standard.

3,5-dimethyl-2-(trifluoromethyl)pyrazine (4i)

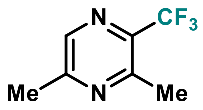

Following the general procedure, the reaction was performed with 2,6-dimethylpyrazine (**2i**, 22 mg, 0.20 mmol), **3** (85 µL, 0.80 mmol, 4.0 equiv), K_2CO_3 (55 mg, 0.40 mmol, 2.0 equiv) and **1a** (9.0 mg, 0.020 mmol, 10 mol%) for 18 h under blue light irradiation. The reaction mixture was diluted with $CDCl_3$, and the yield was determined by ^{19}F NMR (56% yield) using benzotrifluoride (25 µL, 0.20 mmol) as an internal standard. Double trifluoromethylated by-product was observed in 8%.

^{19}F NMR (282 MHz, $CDCl_3$) δ –65.5.

HRMS (EI): calc'd for $C_7H_7N_2F_3$ $[M]^+$: 176.055583, found: 176.055730.

The spectral data matched with those reported in the literature.[11]

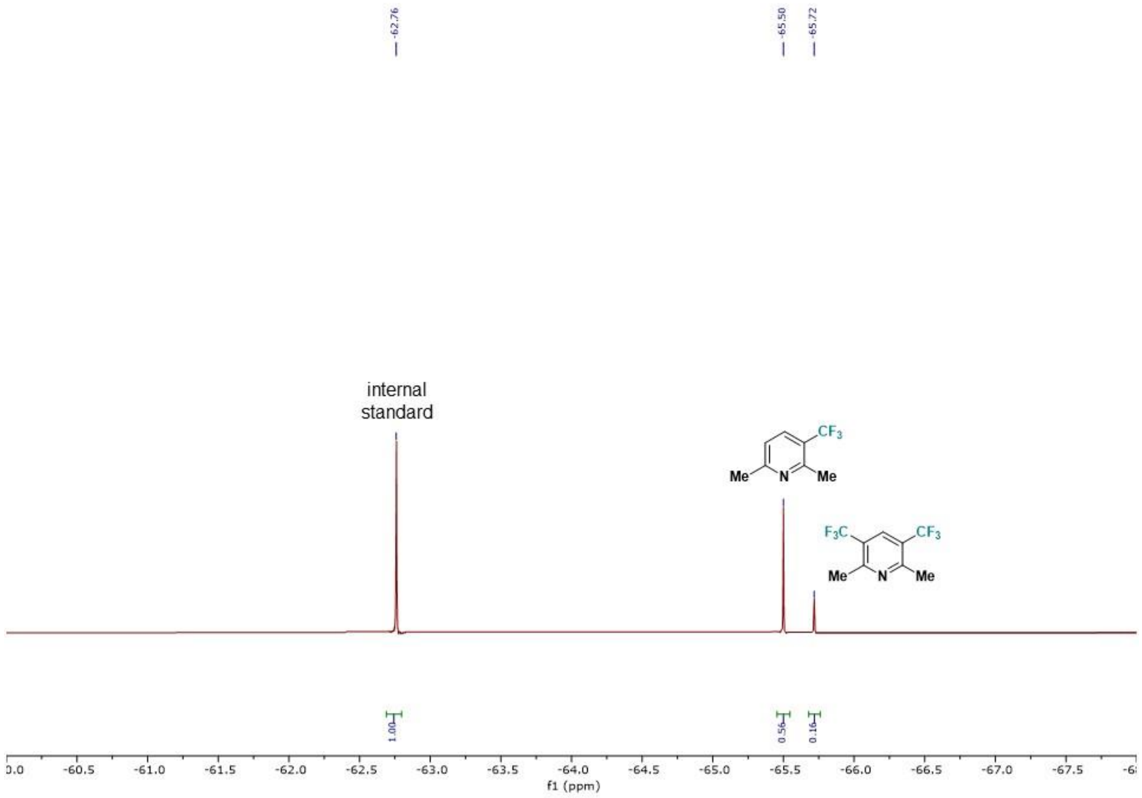

Figure S5 Crude NMR yield of **4i** using benzotrifluoride as internal standard.

4,6-dimethyl-5-(trifluoromethyl)-2H-pyran-2-one (4j)

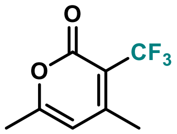

Following the general procedure, the reaction was performed with 4,6-dimethyl-*α*-pyrone (**2j**, 25 mg, 0.20 mmol), **3** (43 µL, 0.40 mmol, 2.0 equiv), and **1b** (16 mg, 0.020 mmol, 10 mol%) for 18 h under white light irradiation. The crude mixture was purified by preparative TLC (Pentane/DCM = 60:40) to afford the product in 47% (18 mg, 0.094 mmol) as an off-white solid.

^{1}H NMR (300 MHz, $CDCl_3$) δ 5.91 (s, 1H), 2.34 (q, J = 2.8 Hz, 3H), 2.26 (t, J = 0.9 Hz, 3H).

^{13}C{^{1}H} NMR (151 MHz, $CDCl_3$) δ 164.1, 158.3 (q, J = 1.6 Hz), 158.2 (q, J = 1.6 Hz), 123.3 (q, J = 273.9 Hz), 111.3 (q, J = 31.1 Hz), 108.4, 20.8 (q, J = 3.8 Hz), 20.0.

^{19}F NMR (282 MHz, $CDCl_3$) δ –58.1.

HRMS (EI): calc'd for $C_8H_7O_2F_3$ $[M]^+$: 192.039440, found: 192.039265.

1-methyl-2-(trifluoromethyl)-1*H*-pyrrole (4k)

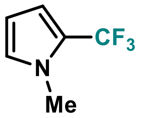

Following the general procedure, the reaction was performed with *N*-methyl pyrrole (**2k**, 36 µL, 0.40 mmol, 2.0 equiv), **3** (21 µL, 0.20 mmol), CsF (61 mg, 0.20 mmol, 2.0 equiv) and **1a** (9.0 mg, 0.020 mmol, 10 mol%) in $CHCl_3$ (0.50 mL, 0.40 M) for 4 h under blue light irradiation. The reaction mixture was diluted with $CDCl_3$, and the yield was determined by ^{19}F NMR (80% yield) using benzotrifluoride (25 µL, 0.20 mmol) as an internal standard.

^{19}F NMR (282 MHz, $CDCl_3$) δ –58.8.

HRMS (EI): calc'd for $C_6H_6NF_3$ $[M]^+$: 149.044684, found: 149.044870.

The spectral data matched with those reported in the literature.[11]

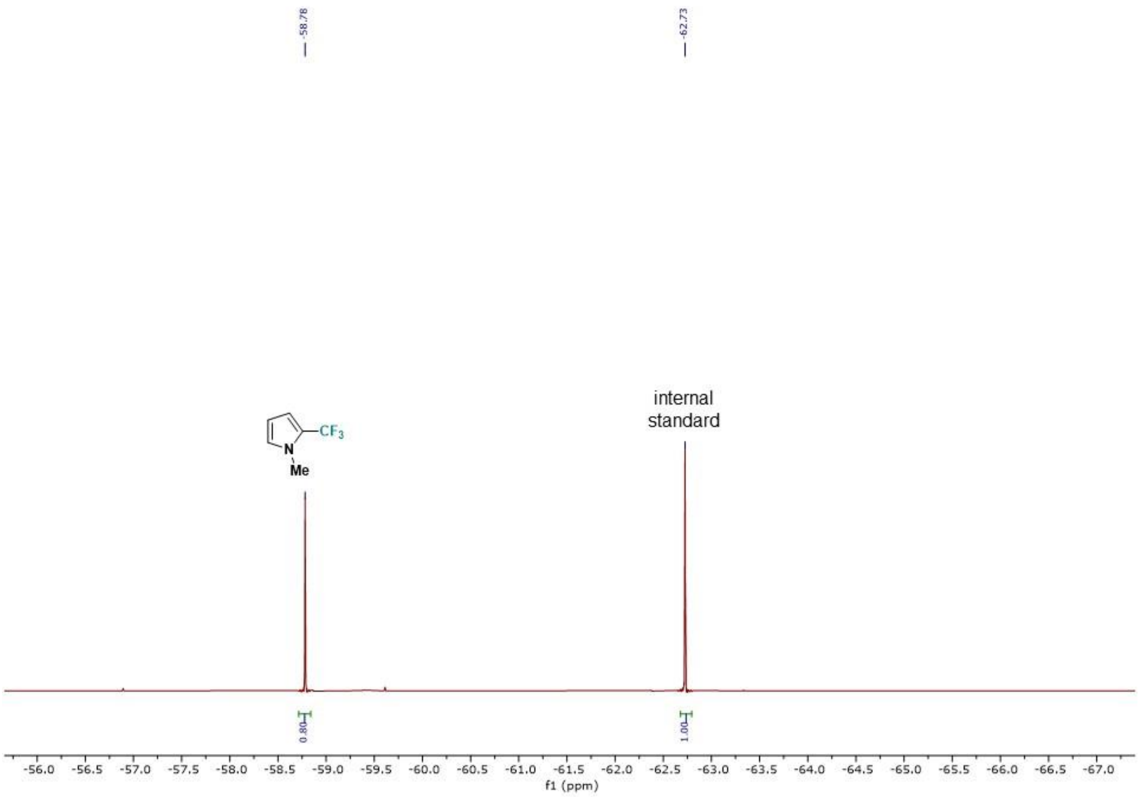

Figure S6. Crude NMR yield of **4k** using benzotrifluoride as internal standard.

***tert*-butyl 2-(trifluoromethyl)-1*H*-pyrrole-1-carboxylate (4l)**

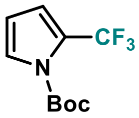

Following the general procedure, the reaction was performed with *N*-methyl pyrrole (**2l**, 33 mg, 0.20 mmol), **3** (43 µL, 0.40 mmol, 2.0 equiv), CsF (61 mg, 0.20 mmol, 2.0 equiv) and **1a** (9.0 mg, 0.020 mmol, 10 mol%) in $CHCl_3$ (0.50 mL, 0.40 M) for 48 h under blue light irradiation. The crude mixture was purified by preparative TLC (pentene/Et_2O = 95:5) to afford the product in 68% (32.0 mg, 0.137 mmol) as a colorless oil.

^{1}H NMR (600 MHz, $CDCl_3$) δ 7.44 (dd, *J* = 3.3, 1.9 Hz, 1H), 6.76–6.70 (m, 1H), 6.19 (t, *J* = 3.5 Hz, 1H), 1.61 (s, 9H).

^{13}C{^{1}H} NMR (151 MHz, $CDCl_3$) δ 147.6, 125.9 (q, *J* = 2.1 Hz), 121.9 (q, *J* = 40.1 Hz), 120.7 (q, *J* = 266.2 Hz), 117.9 (q, *J* = 4.6 Hz), 109.8, 85.8, 27.9.

^{19}F NMR (565 MHz, $CDCl_3$) δ –58.3.

HRMS (ESI): calc'd for $C_{10}H_{12}NO_2F_3Na$ $[M+Na]^+$: 258.07123, found: 258.07115.

The spectral data matched with those reported in the literature.[11]

3,4-dimethoxy-2-(trifluoromethyl)thiophene (4m)

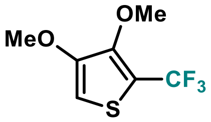

Following the general procedure, the reaction was performed with 3,4-dimethoxy thiophene (**2m**, 29 mg, 0.20 mmol), **3** (85 μL, 0.8 mmol, 4 equiv), CsF (61 mg, 0.20 mmol, 2.0 equiv) and **1a** (9.0 mg, 0.020 mmol, 10 mol%) in $CHCl_3$ (0.50 mL, 0.40 M) for 18 h under blue light irradiation. The crude mixture was purified by preparative TLC (pentene/Et_2O = 90:10) to afford the product in 59% (25.0 mg, 0.118 mmol) as yellow liquid

^{1}H NMR (600 MHz, $CDCl_3$) δ 6.33 (s, 1H), 3.94 (s, 3H), 3.85 (s, 3H).

^{13}C{^{1}H} NMR (151 MHz, $CDCl_3$) δ 150.7, 147.5 (q, *J* = 3.3 Hz), 122.3 (q, *J* = 268.5 Hz), 113.1 (q, *J* = 37.9 Hz), 98.5 (q, *J* = 1.8 Hz), 61.2, 57.7.

^{19}F NMR (565 MHz, $CDCl_3$) δ –55.6.

HRMS (ESI): calc'd for $C_7H_7F_3O_2S$ $[M+H]^+$: 213.01927, found: 213.01916.

The spectral data matched with those reported in the literature.[12]

1-benzyl-3-methyl-2-(trifluoromethyl)-1*H*-indole (4n)

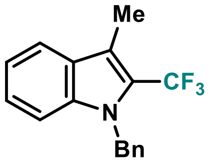

Following the general procedure, the reaction was performed with 1-benzyl-3-methyl-1*H*-indole (**2n**, 177 mg, 0.800 mmol, 4.00 equiv), **3** (21 μL, 0.20 mmol), CsF (61 mg, 0.20 mmol, 2.0 equiv) and **1a** (9.0 mg, 0.020 mmol, 10 mol%) in $CHCl_3$ (0.50 mL, 0.40 M) for 18 h under blue light irradiation. The crude mixture was purified by preparative TLC (pentene/Et_2O = 95:5) to afford the product in 44% (25.7 mg, 0.0890 mmol) as a colorless oil.

^{1}H NMR (300 MHz, $CDCl_3$) δ 7.68 (d, *J* = 7.6 Hz, 1H), 7.33–7.15 (m, 6H), 6.99 (d, *J* = 5.9 Hz, 2H), 5.46 (s, 2H), 2.52 (q, *J* = 2.6 Hz, 3H).

^{13}C{^{1}H} NMR (151 MHz, $CDCl_3$) δ 137.7, 137.5, 128.8, 127.5, 127.4, 125.9, 125.0, 122.9 (q, *J* = 35.2 Hz), 122.8 (q, *J* = 269.7 Hz), 120.4, 120.3, 115.1 (q, *J* = 3.0 Hz), 110.6, 48.3 (q, *J* = 2.4 Hz), 9.2 (q, *J* = 1.9 Hz).

^{19}F NMR (282 MHz, $CDCl_3$) δ –55.2.

HRMS (EI): calc'd for $C_{17}H_{14}N_1F_3$ $[M]^+$: 289.107284, found: 289.107550.

The spectral data matched with those reported in the literature.[13]

5.2. Characterization of direct C–H trifluoromethylation of natural products.

(*R*)-3,6-dimethyl-2-(trifluoromethyl)-4,5,6,7-tetrahydrobenzofuran (4o)

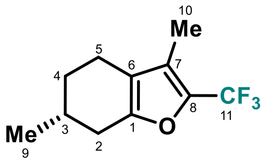

Following the general procedure, the reaction was performed with (+)-menthofuran (**2o**, 30 mg, 0.20 mmol), **3** (85 µL, 0.8 mmol, 4 equiv), CsF (61 mg, 0.20 mmol, 2.0 equiv) and **1a** (9.0 mg, 0.020 mmol, 10 mol%) in $CHCl_3$ (0.50 mL, 0.40 M) for 18 h. under blue light irradiation The crude mixture was purified by preparative TLC (hexane) to afford the product in 64% (28 mg, 0.13 mmol) as a colorless oil.

^{1}H NMR (600 MHz, $CDCl_3$) δ 2.67 (ddq, *J* = 16.5, 5.5, 1.3 Hz, 1H, H-2a), 2.41–2.34 (m, 1H, H-5a), 2.34–2.26 (m, 1H, H-5b), 2.18 (ddq, *J* = 16.2, 9.4, 2.1 Hz, 1H, H-2b), 2.04 (q, *J* = 2.0 Hz, 3H, H-10), 1.93 (ddddd, *J* = 10.8, 9.4, 6.7, 5.4, 2.8 Hz, 1H, H-3), 1.85 (ddddd, *J* = 13.0, 5.5, 3.6, 2.9, 1.0 Hz, 1H, H-4a), 1.36 (dddd, *J* = 13.3, 10.8, 10.0, 5.7 Hz, 1H, H-4b), 1.08 (d, *J* = 6.7 Hz, 3H, H-9).

^{13}C{^{1}H} NMR (151 MHz, $CDCl_3$) δ 152.6 (q, *J* = 1.6 Hz, C-1), 135.1 (q, *J* = 39.9 Hz, C-8), 122.6 (q, *J* = 2.5 Hz, C-7), 120.9 (q, J = 266.9 Hz, C-11), 119.1 (C-6), 31.2 (C-2), 31.0 (C-4), 29.5 (C-3), 21.4 (C-9), 19.7 (C-5), 8.09–7.95 (m, C-10).

^{19}F NMR (565 MHz, $CDCl_3$) δ –61.2– –61.3 (m).

HRMS (EI): calc'd for $C_{11}H_{13}OF_3$ $[M]^+$: 218.091301, found: 218.091520.

1,3,7-trimethyl-8-(trifluoromethyl)-3,7-dihydro-1H-purine-2,6-dione (4p)

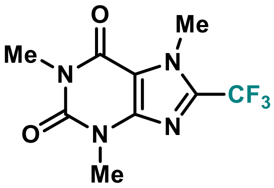

Following the general procedure, the reaction was performed with caffeine (**2p**, 39 mg, 0.20 mmol), **3** (43 µL, 0.40 mmol, 2.0 equiv) and **1b** (16 mg, 0.020 mmol, 10 mol%) for 18 h under white light irradiation. The crude mixture was purified by preparative TLC (hexane/EtOAc = 70:30) to afford the product in 58% (30.4 mg, 0.116 mmol) as a white solid.

^{1}H NMR (300 MHz, $CDCl_3$) δ 4.15 (q, *J* = 1.1 Hz, 3H), 3.59 (s, 3H), 3.42 (s, 3H).

^{13}C{^{1}H} NMR (151 MHz, $CDCl_3$) δ 155.6, 151.5, 146.7, 139.1 (q, *J* = 39.9 Hz), 118.3 (q, *J* = 271.3 Hz), 109.8, 33.3 (q, *J* = 1.9 Hz), 30.1, 28.4.

^{19}F NMR (282 MHz, $CDCl_3$) δ –62.4.

HRMS (EI): calc'd for $C_9H_9N_4O_2F_3$ $[M]^+$: 262.067210, found: 262.067450.

The spectral data matched with those reported in the literature.[8]

1,3-dimethyl-5-(trifluoromethyl)pyrimidine-2,4(1H,3H)-dione (4q)

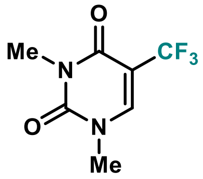

Following the general procedure, the reaction was performed with 1,3-dimethyluracil (**2q**, 22 mg, 0.20 mmol), **3** (85 µL, 0.8 mmol, 4 equiv), CsF (61 mg, 0.20 mmol, 2.0 equiv) and **1a** (9.0 mg, 0.020 mmol, 10 mol%) in $CHCl_3$ (0.50 mL, 0.40 M) for 18 h under blue light irradiation. The crude mixture was purified by preparative TLC (DCM/MeOH = 99:1) to afford the product in 67% (28.1 mg, 0.135 mmol) as an off-white solid.

^{1}H NMR (600 MHz, $CDCl_3$) δ δ 7.66 (q, *J* = 1.2 Hz, 1H), 3.49 (s, 3H), 3.37 (s, 3H).

^{13}C{^{1}H} NMR (151 MHz, $CDCl_3$) δ 158.8, 151.1, 143.6 (q, *J* = 5.8 Hz), 122.1 (q, *J* = 269.8 Hz), 104.3 (q, *J* = 33.0 Hz), 37.9, 28.2.

^{19}F NMR (565 MHz, $CDCl_3$) δ –63.8.

HRMS (EI): calc'd for $C_7H_7N_2O_2F_3$ $[M]^+$: 208.045470, found: 208.045413.

The spectral data matched with those reported in the literature.[14]

Brucin-CF_3 (4r)

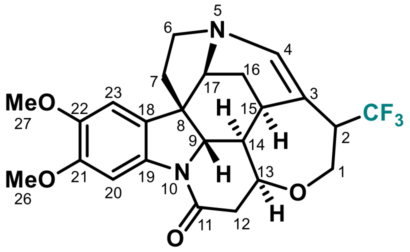

Following the general procedure, the reaction was performed with brucin (**4r**, 62 mg, 0.20 mmol), **3** (85 µL, 0.80 mmol, 4.0 equiv), CsF (61 mg, 0.20 mmol, 2.0 equiv) and **1a** (9.0 mg, 0.020 mmol, 10 mol%) in $CHCl_3$ (0.50 mL, 0.40 M) for 72 h under blue light irradiation. The crude mixture was purified by preparative TLC (DCM/MeOH = 95:5) to afford the product in 27% (25.3 mg, 0.0550 mmol) as a white solid.

^{1}H NMR (600 MHz, $CDCl_3$) δ 7.76 (s, 1H, H-20), 6.71 (s, 1H, H-23), 5.97 (s, 1H, H-4), 4.28 (dd, *J* = 12.8, 6.8, 1H, H-1a), 4.00 (dt, *J* = 8.6, 4.4, 1H, H-13), 3.90 (s, 3H, H-26), 3.89 (s, 3H, H-27), 3.78 (d, *J* = 10.1, 1H, H-9), 3.69 (dd, *J* = 12.8, 11.6, 1H, H-1b), 3.49 (ddd, *J* = 11.0, 10.5, 6.5, 1H, H-6a), 3.25 (ddd, *J* = 12.2, 9.3, 4.2, 1H, H-6b), 3.14 (m, 1H, H-2), 3.04 (dd, *J* = 16.5, 8.5, 1H, H-12a), 2.72 (m, 1H, H-15), 2.60 (dd, *J* = 16.6, 4.5, 1H, H-12b), 2.35 (dt, *J* = 13.5, 3.6, 1H, H-16a), 2.31 (ddd, *J* = 13.7, 9.4, 7.3, 1H, H-7a), 1.93 (ddd, *J* = 13.7, 9.4, 7.3, 1H, H-7b), 1.83 (dt, *J* = 13.7, 2.7, 1H, H-16b), 1.45 (dt, *J* = 10.1, 3.9, 1H, H-14a)

^{13}C{^{1}H} NMR (151 MHz, $CDCl_3$) δ 169.8 (C-11), 149.5 (C-21), 146.7 (C-22), 140.6 (C-4), 135.5 (C-19), 126.1 (q, C-24), 123.8 (C-18), 105.2 (C-23), 100.9 (C-20), 66.3 (C-1), 64.5 (C-9), 58.9 (C-17), 56.7 (C-27), 56.4 (C-26), 53.8 (C-8), 53.5 (C-14), 52.1 (C-6), 48.8 (q, *J* = 9.55, C-2), 45.3 (C-7), 41.2 (C-12), 27.2 (C-15), 27.0 (C-16). (Note: C-3 was not found because of the peak broadening.)

^{19}F NMR (282 MHz, $CDCl_3$) δ –70.0.

HRMS (ESI): calc'd for $C_{24}H_{25}N_2F_3O_4$ $[M+H]^+$: 463.18392, found: 463.18384.

NOTE: Although the double bond of brucin is originally at C2-C3 position, it can be migrated to C3-C4 position, which was observed in related radical trifluoromethylation.

The spectral data matched with those reported in the literature.[15]

Griseofulvin-CF_3 (4s)

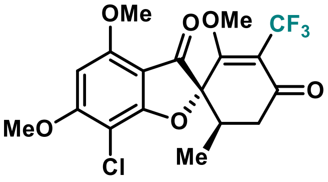

Following the general procedure, the reaction was performed with (+)-griseofulvin (**2s**, 71 mg, 0.20 mmol), **3** (85 µL, 0.80 mmol, 4.0 equiv), CsF (61 mg, 0.20 mmol, 2.0 equiv) and **1a** (9.0 mg, 0.020 mmol, 10 mol%) in $CHCl_3$ (0.50 mL, 0.40 M) for 18 h under blue light irradiation. The crude mixture was purified by preparative TLC (Pentane/DCM/AcOEt = 50:20:30) to afford the product in 25% (21 mg, 0.050 mmol) as a yellowish solid.

^{1}H NMR (600 MHz, $CDCl_3$) δ 6.16 (s, 1H), 4.05 (s, 3H), 3.99 (s, 3H), 3.86 (q, J = 0.7 Hz, 3H), 3.13 (dd, J = 16.9, 13.9 Hz, 1H), 2.83 (dqd, J = 13.5, 6.7, 4.9 Hz, 1H), 2.53 (dd, J = 16.9, 4.9 Hz, 1H), 0.95 (d, J = 6.7 Hz, 3H).

^{13}C{^{1}H} NMR (151 MHz, $CDCl_3$) δ 192.0, 191.0, 173.0 (q, J = 1.9 Hz), 169.2, 165.1, 158.1, 121.7 (q, J = 276.1 Hz), 118.2 (q, J = 28.2 Hz), 105.1, 97.6, 92.3, 90.1, 64.6 (q, J = 3.1 Hz), 57.3, 56.6, 40.2, 36.0, 14.3.

^{19}F NMR (565 MHz, $CDCl_3$) δ –58.5.

HRMS (ESI): calc'd for $C_{18}H_{16}Cl_1F_3Na_1O_6$ $[M+Na]^+$: 443.04819, found: 443.04797.

Surfadoxine-CF_3 (4t)

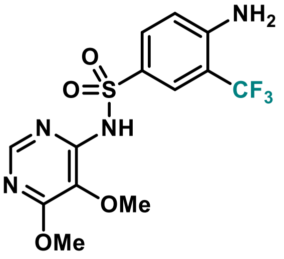

Following the general procedure, the reaction was performed with sulfadoxine (**2t**, 62 mg, 0.20 mmol), **3** (85 µL, 0.80 mmol, 4.0 equiv), CsF (61 mg, 0.20 mmol, 2.0 equiv) and **1a** (9.0 mg, 0.020 mmol, 10 mol%) in $CHCl_3$ (0.50 mL, 0.40 M) for 48 h under blue light irradiation. The crude mixture was purified by preparative TLC (DCM/MeOH = 98:2) to afford the product in 28% (21 mg, 0.056 mmol) as an off-white solid.

^{1}H NMR (600 MHz, $CDCl_3$) δ 8.27 (dd, *J* = 2.2, 0.7 Hz, 1H), 8.17 (s, 1H), 8.05 (ddd, *J* = 8.8, 2.3, 0.5 Hz, 1H), 6.75 (dd, *J* = 8.8, 0.7 Hz, 1H), 3.99 (s, 3H), 3.86 (s, 3H).

^{13}C{^{1}H} NMR (151 MHz, $CDCl_3$) δ 161.0, 151.1, 149.8, 148.7, 133.6, 129.0 (q, *J* = 5.3 Hz), 127.3, 126.5 124.1 (q, *J* = 270 Hz), 116.3, 112.7 (q, *J* = 31.4 Hz), 60.7, 54.3.

^{19}F NMR (565 MHz, $CDCl_3$) δ –63.3.

HRMS (ESI): calc'd for $C_{13}H_{13}F_3N_4NaO_4S$ $[M+Na]^+$: 401.05013, found: 401.05018.

5.3. Characterization of nonafluorobutylation product

1,3,5-trimethoxy-2-(4,4,4,4,4,4,4,4,4-nonafluoro-4l12-buta-1,3-diyn-1-yl)benzene (4a')

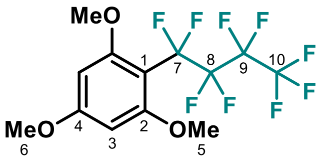

Following the general procedure, the reaction was performed with 1,3,5-trimethoxybenzene (**2a**, 34 mg, 0.20 mmol), nonafluoro-1-butanesulfonyl chloride (42 µL, 0.24 mmol, 1.2 equiv) and **1b** (16 mg, 0.020 mmol, 10 mol%) for 18 h under white light irradiation. The crude mixture was purified by preparative TLC (hexane/Et_2O = 80:20) to afford the product in 94% (72.3 mg, 0.187 mmol) as a white solid.

^{1}H NMR (300 MHz, $CDCl_3$) δ 6.15 (s, 2H), 3.84 (s, 3H), 3.80 (s, 6H).

^{13}C{^{1}H} NMR (151 MHz, $CDCl_3$) δ 164.0 (C-4), 161.9 (t, *J* = 2.26 Hz, C-2), 117.9 (qt, *J* = 288.4, 33.0 Hz, C-10), 117.3 (tt, *J* = 259.1, 33.7 Hz, C-7), 111.2 (tp, *J* = 267.0, 37.0 Hz, C-8), 109.3 (th, *J* = 267.0, 37.0 Hz, C-9), 98.7 (t, *J* = 21.8 Hz, C-1), 91.9 (C-3), 56.5 (C-5), 55.5 (C-6).

^{19}F NMR (282 MHz, $CDCl_3$) δ –80.9 (tt, *J* = 10.2, 2.9 Hz, F-10), –102.8 (dddd, *J* = 16.8, 11.3, 5.8, 2.9 Hz, F-7), –120.8 – –124.4 (m, F-8), –124.4 – –129.7 (m, F-9).

HRMS (EI): calc'd for $C_{13}H_{11}O_3F_9$ $[M]^+$: 386.055901, found: 386.055980.

The spectral data matched with those reported in the literature.[16]

6. X-ray data

6.1. Single crystal structure analysis of **5a**

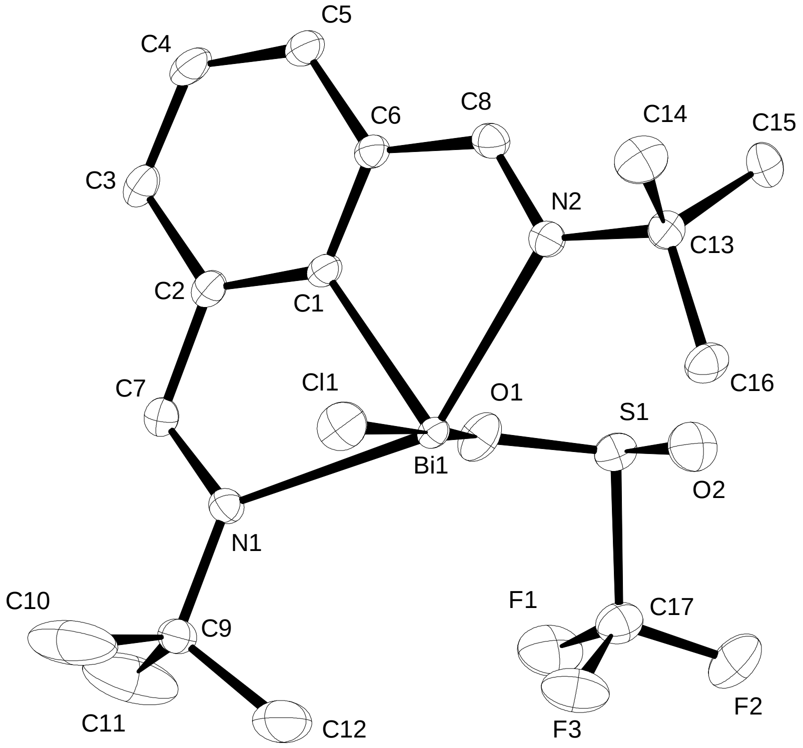

Figure S7. The molecular structure of **5a**. H atoms have been removed for clarity. Complete .cif-data of the compound are available under the CCDC number **CCDC-2288158.**

Table S5. Crystal data and structure refinement of **5a**.

Identification code	14625	
Empirical formula	$C_{17} H_{23} Bi Cl F_3 N_2 O_2 S$	
Color	colourless	
Formula weight	620.86 g · mol^{-1}	
Temperature	100(2) K	
Wavelength	0.71073 Å	
Crystal system	MONOCLINIC	
Space group	**$P2_1/n$, (no. 14)**	
Unit cell dimensions	a = 12.5744(6) Å	α = 90°.
	b = 9.3258(5) Å	β = 106.339(3)°.
	c = 18.6891(10) Å	γ = 90°.
Volume	2103.09(19) $Å^3$	
Z	4	
Density (calculated)	1.961 Mg · m^{-3}	
Absorption coefficient	8.650 mm^{-1}	
F(000)	1192 e	
Crystal size	0.191 x 0.105 x 0.041 mm^3	
θ range for data collection	2.271 to 30.508°.	
Index ranges	$-17 \le h \le 17$, $-13 \le k \le 13$, $-26 \le l \le 26$	
Reflections collected	260312	
Independent reflections	6424 [R_{int} = 0.0451]	
Reflections with I>2σ(I)	6055	
Completeness to θ = 25.242°	100.0 %	
Absorption correction	Semi-empirical from equivalents	
Max. and min. transmission	0.72 and 0.33	
Refinement method	Full-matrix least-squares on F^2	
Data / restraints / parameters	6424 / 0 / 250	
Goodness-of-fit on F^2	1.149	
Final R indices [I>2σ (I)]	R_1 = 0.0197	wR^2 = 0.0450
R indices (all data)	R_1 = 0.0214	wR^2 = 0.0456
Largest diff. peak and hole	2.3 and -1.7 e · $Å^{-3}$	

Table S6. Bond lengths [Å] and angles [°] of **5a**.

Bi(1)-Cl(1)	2.5646(7)
Bi(1)-O(1)	2.5618(19)
Bi(1)-N(1)	2.495(2)
Bi(1)-N(2)	2.498(2)
Bi(1)-C(1)	2.199(2)
S(1)-O(1)	1.509(2)
S(1)-O(2)	1.483(2)
S(1)-C(17)	1.869(3)
F(1)-C(17)	1.339(3)
F(2)-C(17)	1.327(3)
F(3)-C(17)	1.337(3)
N(1)-C(7)	1.277(3)
N(1)-C(9)	1.495(3)
N(2)-C(8)	1.276(3)
N(2)-C(13)	1.494(3)
C(1)-C(2)	1.387(3)
C(1)-C(6)	1.386(3)
C(2)-C(3)	1.401(3)
C(2)-C(7)	1.471(3)
C(3)-C(4)	1.391(4)
C(4)-C(5)	1.389(4)
C(5)-C(6)	1.400(3)
C(6)-C(8)	1.469(3)
C(9)-C(10)	1.506(5)
C(9)-C(11)	1.515(4)
C(9)-C(12)	1.509(5)
C(13)-C(14)	1.530(4)
C(13)-C(15)	1.530(4)
C(13)-C(16)	1.522(4)
O(1)-Bi(1)-Cl(1)	168.70(5)
N(1)-Bi(1)-Cl(1)	88.29(5)
N(1)-Bi(1)-O(1)	81.17(7)
N(1)-Bi(1)-N(2)	141.47(7)
N(2)-Bi(1)-Cl(1)	85.85(5)
N(2)-Bi(1)-O(1)	99.84(7)

C(1)-Bi(1)-Cl(1)	92.68(6)
C(1)-Bi(1)-O(1)	80.12(7)
C(1)-Bi(1)-N(1)	71.29(8)
C(1)-Bi(1)-N(2)	70.99(8)
O(1)-S(1)-C(17)	99.70(13)
O(2)-S(1)-O(1)	109.96(12)
O(2)-S(1)-C(17)	100.30(13)
S(1)-O(1)-Bi(1)	114.57(10)
C(7)-N(1)-Bi(1)	111.95(16)
C(7)-N(1)-C(9)	121.1(2)
C(9)-N(1)-Bi(1)	126.92(15)
C(8)-N(2)-Bi(1)	112.39(17)
C(8)-N(2)-C(13)	120.5(2)
C(13)-N(2)-Bi(1)	127.15(15)
F(1)-C(17)-S(1)	110.7(2)
F(2)-C(17)-S(1)	110.8(2)
F(2)-C(17)-F(1)	107.7(2)
F(2)-C(17)-F(3)	107.6(2)
F(3)-C(17)-S(1)	112.53(19)
F(3)-C(17)-F(1)	107.3(2)
C(2)-C(1)-Bi(1)	119.26(17)
C(6)-C(1)-Bi(1)	119.60(17)
C(6)-C(1)-C(2)	121.1(2)
C(1)-C(2)-C(3)	119.3(2)
C(1)-C(2)-C(7)	117.3(2)
C(3)-C(2)-C(7)	123.3(2)
C(4)-C(3)-C(2)	119.5(2
C(5)-C(4)-C(3)	121.1(2)
C(4)-C(5)-C(6)	119.2(2)
C(1)-C(6)-C(5)	119.7(2)
C(1)-C(6)-C(8)	117.4(2)
C(5)-C(6)-C(8)	122.9(2)
N(1)-C(7)-C(2)	119.8(2)
N(2)-C(8)-C(6)	119.5(2)
N(1)-C(9)-C(10)	108.8(2)
N(1)-C(9)-C(11)	112.0(2)
N(1)-C(9)-C(12)	108.1(2)
C(10)-C(9)-C(11)	109.7(4)

C(10)-C(9)-C(12)	110.0(4)
C(12)-C(9)-C(11)	108.2(3)
N(2)-C(13)-C(14)	108.5(2)
N(2)-C(13)-C(15)	109.5(2)
N(2)-C(13)-C(16)	106.6(2)
C(14)-C(13)-C(15)	112.0(2)
C(16)-C(13)-C(14)	110.1(2)
C(16)-C(13)-C(15)	110.0(2)

The complex **5a** was structurally characterized by single crystal X-ray diffraction, thus confirming the common *trans* disposition of the Cl and SO_2CF_3 anions in its solid state, with directional intermolecular non-covalent interaction, including hydrogen bonds, where with the SO_2CF_3 ligand is coordinated through the oxygen atom to the Bi-center due to its high oxophilicity. The Bi–O bond distances are 2.561(7) Å and 3.225(8) Å, respectively, indicating that the SO_2CF_3 group coordinates in a η^1-fashion because there are some intermolecular hydrogen bonds; 1) between O1 and H_{imine}, which is from an imine group of an adjacent molecule; 2) between S and $H_{t\text{-}Bu}$, which is from ^{t}Bu group of an adjacent molecule; 3) between F and H_{meta}, which is *meta*-hydrogen atom at *meta* position of ligand backbone of an adjacent molecule. Therefore, the complex **5a** is unsymmetrical in the solid state.

The distance of Bi–O2 bond (2.561(7) Å) is much longer than the closely related Bi–TEMPO complex (2.178(3) Å),[17] bismuth(III) sulfinate complex (2.242(2) Å),[18] and the sum of ion radii (2.20 Å). Moreover, the bond length of both S–O1 and S–O2 bond are almost same (1.483(3) Å and 1.509(0) Å, respectively). This configuration revealed that SO_2CF_3 group is rather counter anion than connects to Bi center. Additionally, the Bi–Cl bond (2.564(6) Å) is shorter than that of closely related Bi dichloride (2.662(2) Å and 2.6890(18) Å, respectively).[19] The shorter Bi–Cl bond distance is ascribed to the more cationic character of Bi center. Altogether these structural peculiarities strongly suggest that Bi–O2 bond is strongly polarized and SO_2CF_3 group has a strong interaction with Bi center.

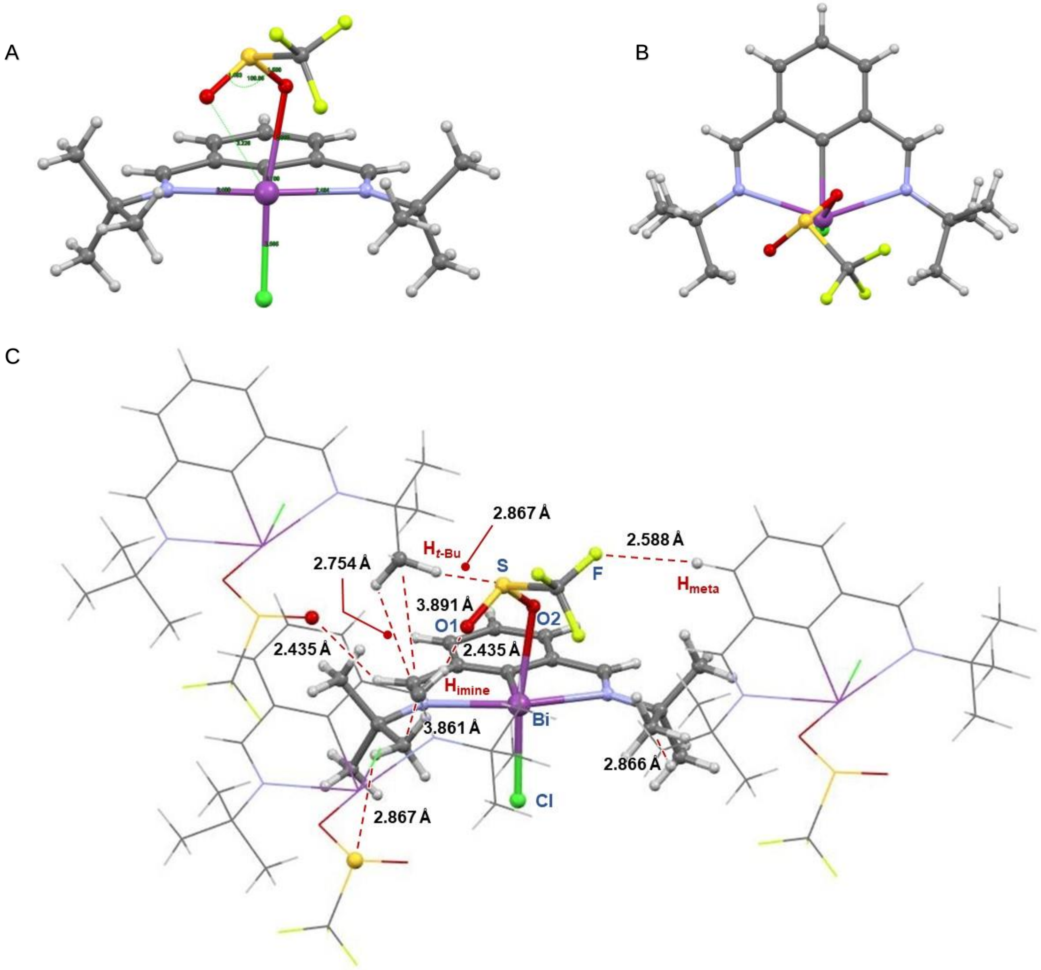

Figure S8. (A) Front view of the complex **5a** (B) Top view of the complex **5a** (C) Molecular packing structure of crystal **5a**. Intermolecular interactions, where the distances of two atom are within the sum of van der Waals (vdW) radii, are shown in red dotted line.

7. Experimental Mechanistic Studies

7.1. Proposed Mechanism

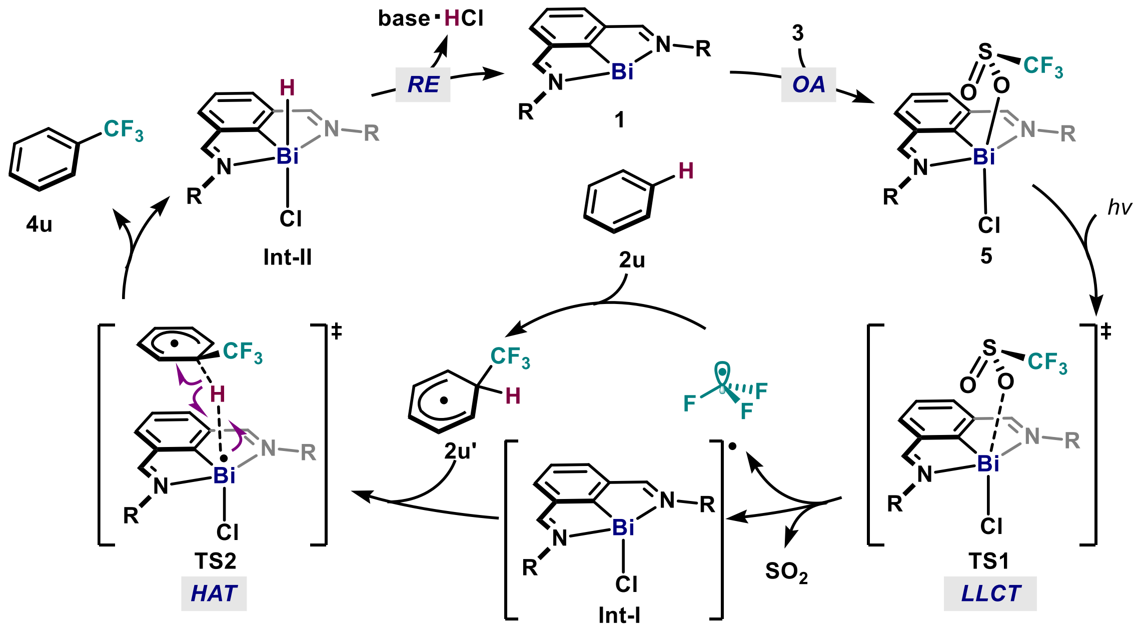

Figure S9. Proposed mechanism of direct C–H radical trifluoromethylation of (hetero)arene via open-shell bismuth redox cycle.

7.2. CF_3 radical trapping with TEMPO

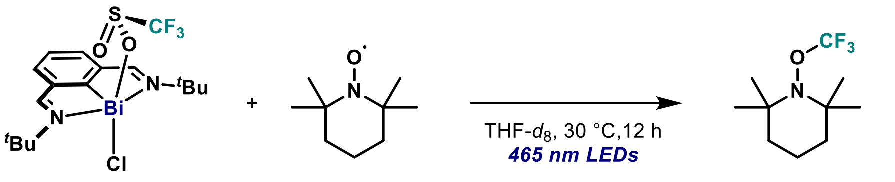

In an argon-filled glove box, **5a** (6.2 mg, 0.010 mmol), TEMPO (3.1 mg, 0.020 mmol, 2.0 equiv), benzotrifluoride (1.5 mg, 0.010 mmol, 1.0 equiv) and THF-d_8 (0.40 mL) were placed in normal NMR tube and it was sealed with parafilm. The NMR tube was taken out of the glovebox and placed it into a 465 nm blue LEDs. After 12 h, the reaction mixture was analyzed by ^{19}F NMR and **6** was detected in 24% yield.

^{19}F NMR (282 MHz, THF-d_8) δ –56.3.

HRMS (ESI): calc'd for $C_{10}H_{19}N_1O_1F_3$ $[M+H]^+$: 226.14133, found: 226.14122.

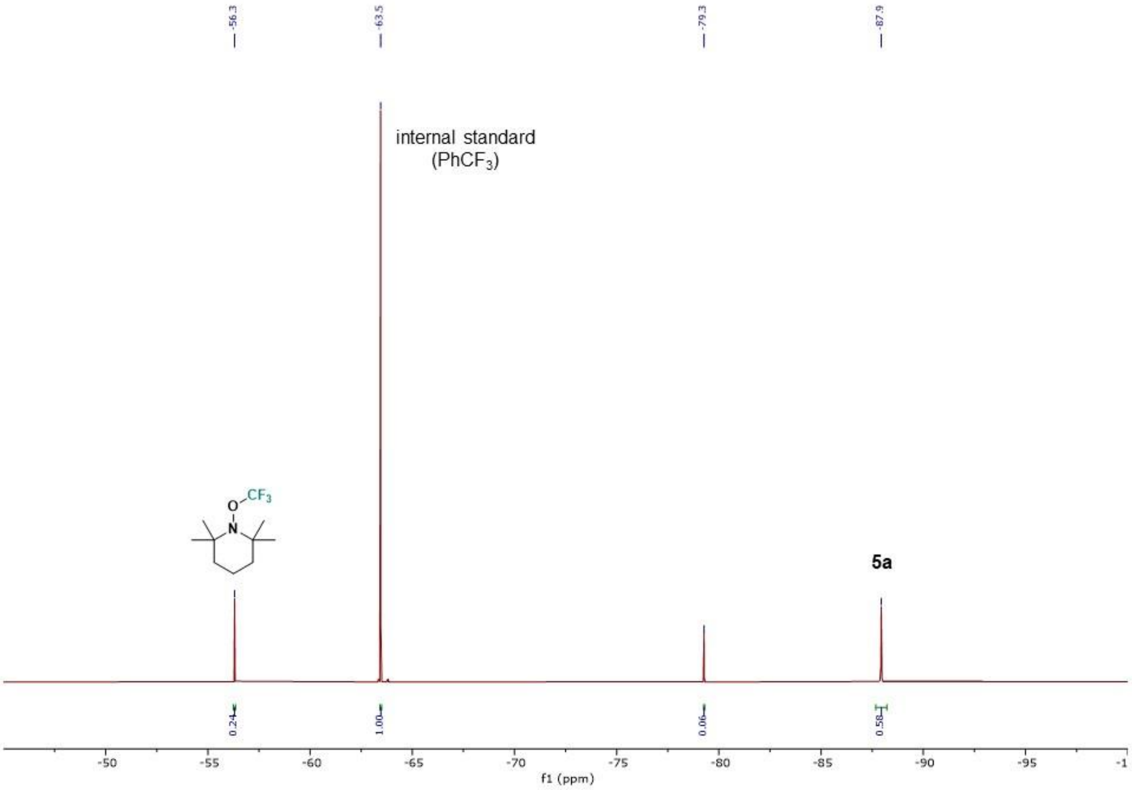

Figure S10. Crude NMR yield of **6** with **5a**.

7.3. The reactivity of **5a** toward C–H trifluoromethylation of **2a**

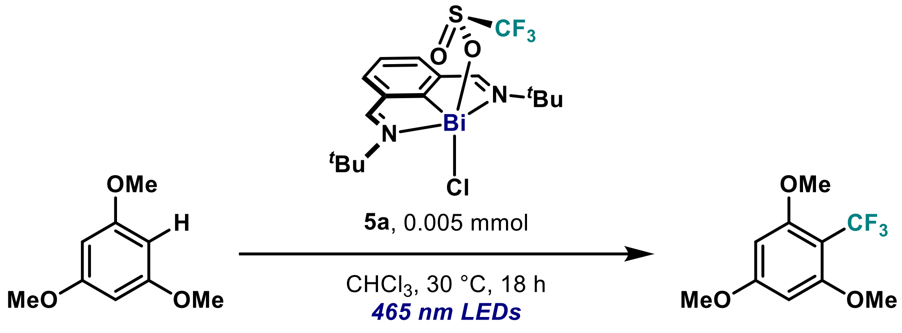

2a, 5.0 equiv **4a**, 71%

In an argon-filled glove box, **2a** (4.2 mg, 0.025 mmol, 5.0 equiv), and **5a** (3.1 mg, 0.0050 mmol) were placed in an oven-dried reaction vial, and the reaction vial was sealed with a screw cap containing a Teflon^{TM}-coated rubber septum. The vial was taken out of the glovebox and chloroform (0.10 mL, 0.050 M, degassed and dried) was added. Then it was placed into the blue LED reactor. After the reaction mixture stirred for 18 h, 4-fluorotoluene (1.7 mg, 1.7 µL, 0.015 mmol, 3.0 equiv) was added as an internal standard. The reaction mixture was diluted with $CDCl_3$, and analyzed by quantitative ^{19}F NMR spectroscopy at 565 MHz without broadband ^{1}H decoupling. **4a** was detected in 71% yield based on **5a**.

^{19}F NMR (282 MHz, THF-d_8) δ –54.2.

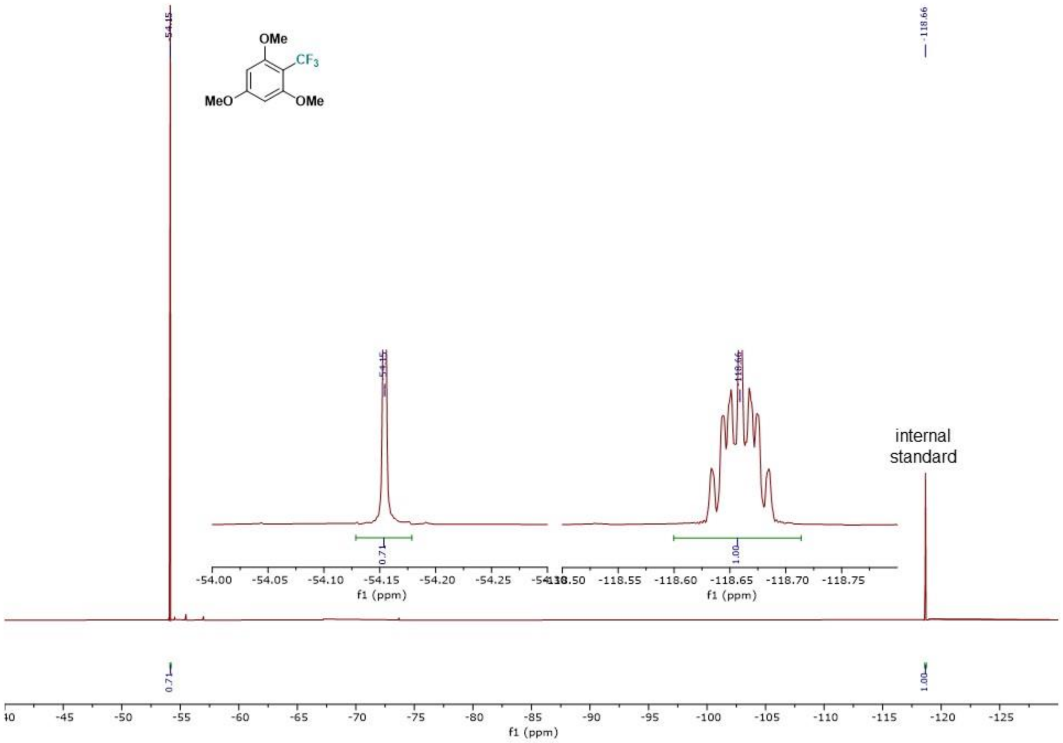

Figure S11. Crude NMR yield of C–H trifluoromethylation with **5a**.

7.4. Quantum yield measurement

Photon flux determination*:* The quantum yield was calculated a modified version of reported procedure.[20,21] The ferrioxalate actinometer solution measures the decomposition of ferric ions to ferrous ions, which are complexed by 1,10-phenanthroline and monitored by UV/Vis absorbance at 510 nm. The moles of iron-phenanthroline complex formed are related to moles of photons absorbed The photon flux was determined by standard ferrioxalate actinometry. A 0.15 M solution of ferrioxalate was prepared by dissolving 0.73 g of potassium ferrioxalate hydrate in 10 mL of H_2SO_4 (0.05 M). A buffered solution of 1,10-phenanthroline was prepared by dissolving 17 mg of 1,10-phenanthroline and 3.75 g of sodium acetate in 17 mL of H_2SO_4 (0.5 M). Both solutions were stored in the dark. To determine the photon flux, 1.0 mL of the ferrioxalate solution was added in a test tube and it was placed in the irradiation set-up where it was irradiated for 10.0 seconds at λ = 456 nm. After irradiation, 0.175 mL of the 1,10-phenanthroline solution was added. The solution was then allowed to rest for 1 h to allow the ferrous ions to completely coordinate to the 1,10-phenanthroline. The absorbance of the solution was measured at 510 nm. A non-irradiated sample was also prepared and the absorbance at 510 nm measured. Conversion was calculated using Eq. 1:

$$\text{Moles of } Fe^{2+} = (V\Delta A)/(l\cdot\varepsilon) \quad \text{(Eq. 1)}$$

Where "V" is the total volume (1.175×10^{-3} L) of the solution after addition of 1,10-phenanthroline, "ΔA" is the difference in absorbance at 510 nm between the irradiated and non-irradiated solutions, "l" is the path length (0.2 cm), and "ε" is the molar absorptivity at 510 nm (11100 L mol^{-1} cm^{-1}).
The photon flux can be calculated using Eq. 2:

$$\text{photon flux} = (\text{Moles of } Fe^{2+})/(\Phi\cdot t\cdot f) \quad \text{(Eq. 2)}$$

Where "Φ" is the quantum yield for the ferrioxalate actinometer (1.1 for a 0.15 M solution at λ = 456 nm), "t" is the time (10.0 s), and "f" is the fraction of light absorbed at λ = 456 nm (0.450459). The photon flux was calculated to be 6.67×10^{-10} einstein s^{-1}.
Sample calculation:

$$\text{Moles of } Fe^{2+} = (1.175\times10^{-3}\ [L]\times0.493655)/(0.2\ [cm]\times11100\ [L\ mol^{-1}\ cm^{-1}]) = 2.61\times10^{-7}\ [mol]$$

$$\text{photon flux} = 2.61\times10^{-7}\ [mol]/(1.11\times10\ [s]\times0.450459) = 5.22\times10^{-7}\ [einstein\ s^{-1}]$$

Quantum yield measurement: As a result of the minimal double trifluoromethylation byproduct observed, the quantum yield was measured on 1,3-dimethyluracil (**2q**). Inside an argon-filled glovebox, **1a** (10 mol%, 0.080 mmol), dimethyluracil (0.80 mmol, 1.0 equiv), and CsF (243 mg, 1.60 mmol, 2.00 equiv) were placed in an oven-dried reaction vial, and the reaction vial was sealed with a screw cap containing a Teflon™-coated rubber septum. The vial was taken out of the glovebox and degassed and dried $CDCl_3$ (2.0 mL) were added. Finally, **3** (340 µL, 3.20 mmol, 4.00 equiv) was added and the vial is placed in the irradiation set-up. After 1 hour, the reaction mixture was diluted with $CDCl_3$, and the yield was determined by ^{19}F NMR spectroscopy using benzotrifluoride as an internal standard (10%).

$$\Phi = 8.0\times10^{-5}\,[\text{mol}]/(5.22\times10^{-7}\,[\text{einstein s}^{-1}]\times3600\,[\text{s}]\times0.22) = 0.19$$

The quantum yield was determined to be 19%

7.5. Aromatization through HAT

According to our proposed mechanism, the trifluoromethylation product is obtained through *hydrogen atom transfer (HAT)* from Bi(II) radical and cyclohexadienyl radical. To test the feasibility of this process, we have evaluated the ability of Bi(II) radical (**Int-Ia**) to abstract hydrogen atom of cyclohexadienyl radical (**2u'**). We employed **7** as a model substrate as it can be converted to anthracene, namely aromatic compound, through HAT.

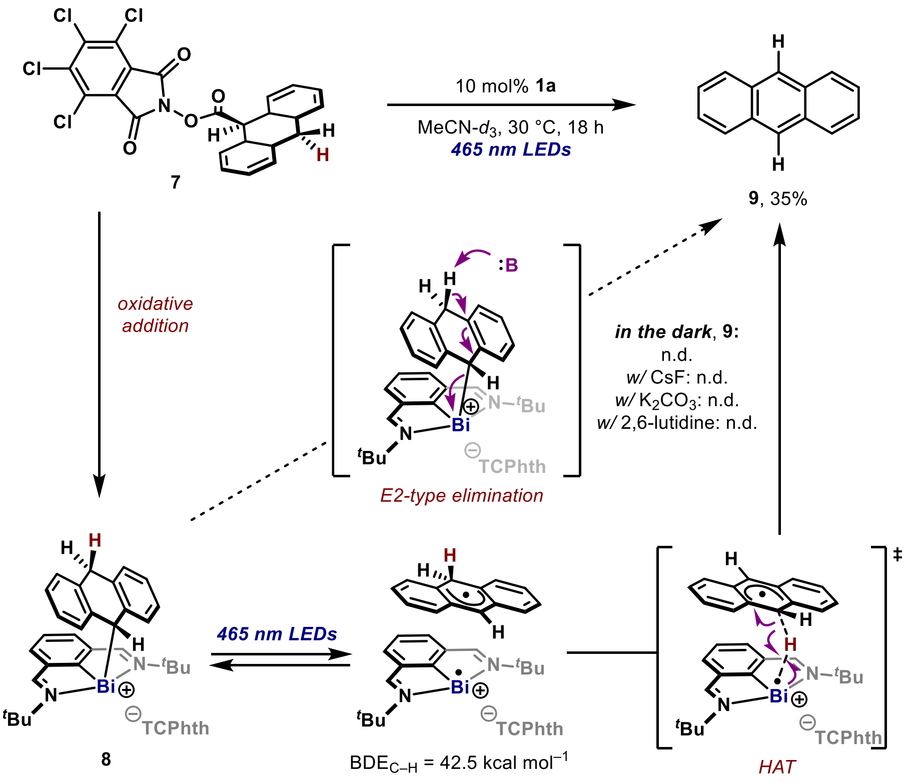

Figure S12. Probing the catalytic re-aromatization through HAT. TCPhth = tetrachlorophtalimide.

In an argon-filled glove box, **1a** (0.9 mg, 0.002 mmol), **7** (10.1 mg, 0.0200 mmol), and MeCN-d_3 (0.5 mL) were placed in normal NMR tube and it was sealed with parafilm. The NMR tube was taken out of the glovebox and analyzed by ^{1}H NMR (Figure S13). After 465 nm LED irradiation for 18 h, the NMR tube was taken into the glovebox again and 1,3,5-trimethoxybenzene (3.4 mg, 0.020 mmol) was added as an internal standard. The reaction mixture was analyzed by ^{1}H NMR and the anthracene was detected in 35% yield (Figure S14). In contrast, when the reaction was performed in the dark, **9** was not observed, even in the presence of 2.0 equiv. of bases (CsF, K_2CO_3, 2,6-lutidine), indicating that light irradiation is necessary to facilitate Bi–C bond homolysis, rather than a E2-type elimination pathway in complex **8**.

9

^{1}H NMR (300 MHz, MeCN-*d*$_3$) δ 8.50 (s, 2H, H_a), 8.06 (m, 4H, H_b), 7.50 (m, 4H, H_c).

HRMS (APPI): calc'd for $C_{14}H_{10}$ [M]$^+$: 178.07770, found: 178.07786.

The spectral data matched with those reported in the literature.[22]

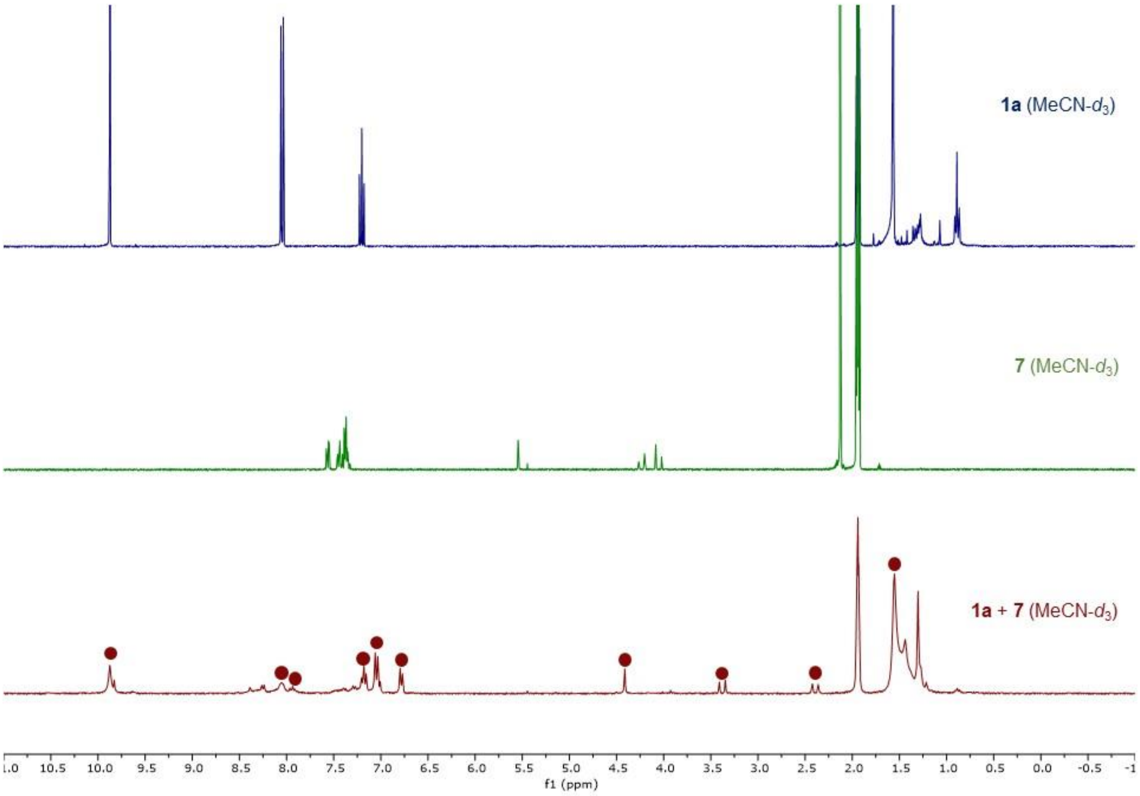

Figure S13. Crude NMR of **1a**, **7**, and **1a**+**7** in MeCN-*d*$_3$.

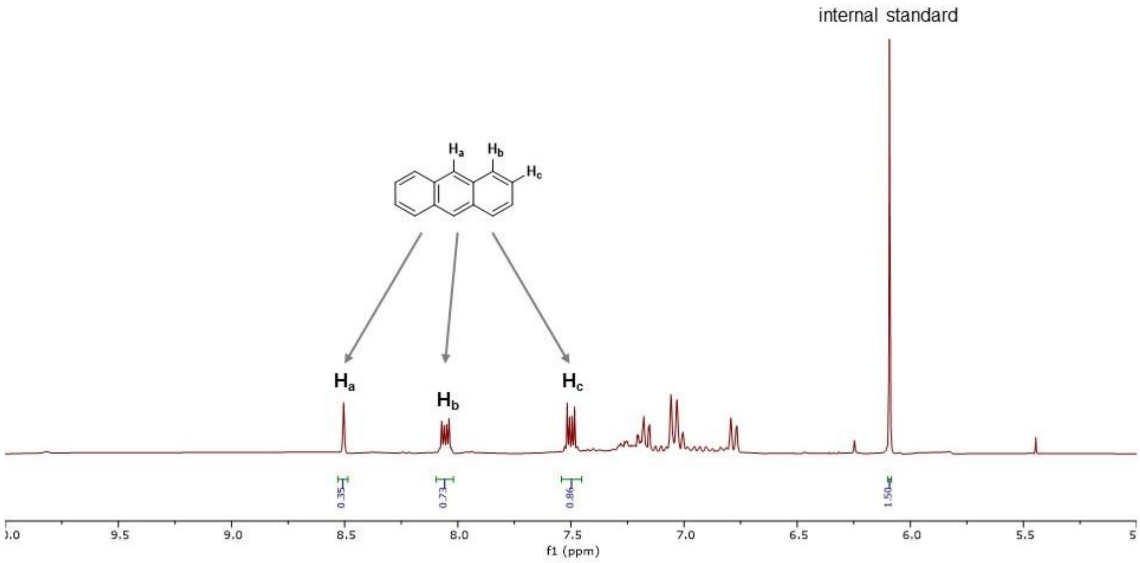

Figure S14. Crude NMR yield of **9** using 1,3,5-trimethoxybenzene as internal standard.

7.7. Kinetic Analysis

7.7.1. Kinetic Analysis without base

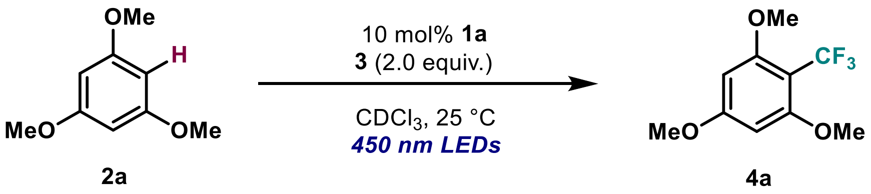

Reaction monitoring under blue light was performed using a fiber-coupled LED NMR setup in analogy to Lenherr and Ji (**Figure S16**).[24] As a light source, a fiber-coupled ultra high power LED from Prizmatix Ltd. was used (LED head: UHP-T-450 SR, peak wavelength λ = 450 nm). Reactions were monitored every 1–3 minutes with single scan ^{1}H and ^{19}F NMR spectra for an appropriate time after the light was turned on full intensity. The raw NMR data was imported into MNOVA 14.3.1 with the reaction monitoring plugin and processed therein. The relative concentration profiles were referenced to the first acquired ^{1}H or ^{19}F NMR spectrum of each reaction.

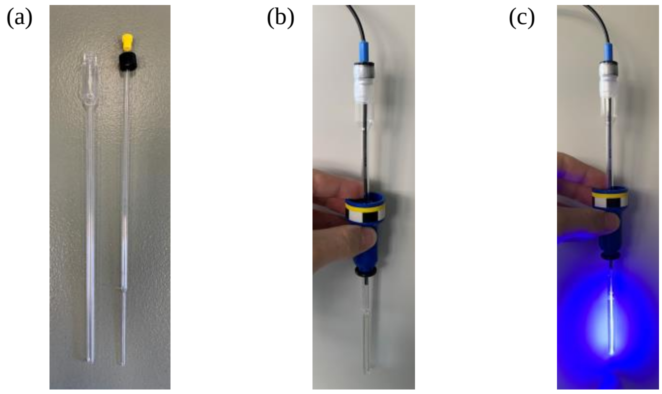

Figure S15. The set-up to perform NMR experiments while the sample was irradiated; (a) NMR tube for the samples (left) and the glass tube which we can insert an optical fiber into it (right); (b) the assembled sample before irradiation. The black tube is an optical fiber; (c) the NMR sample under blue light irradiation.

In an argon-filled glove box, **1a** (2.3 mg, 0.0050 mmol, 10 mol%), **2a** (8.4 mg, 0.050 mmol), **3** (11 µL, 0.10 mmol, 2.0 equiv), and $CDCl_3$ (0.40 mL, dried and degassed) were placed in an oven-dried NMR tube which can be irradiated via an optical fiber during measurement. The NMR tube was closed with parafilm. The NMR tube was brought to the NMR spectrometer while avoiding ambient light with aluminum foil. ^{1}H and ^{19}F NMR spectra were acquired every 1 minute under blue light irradiation.

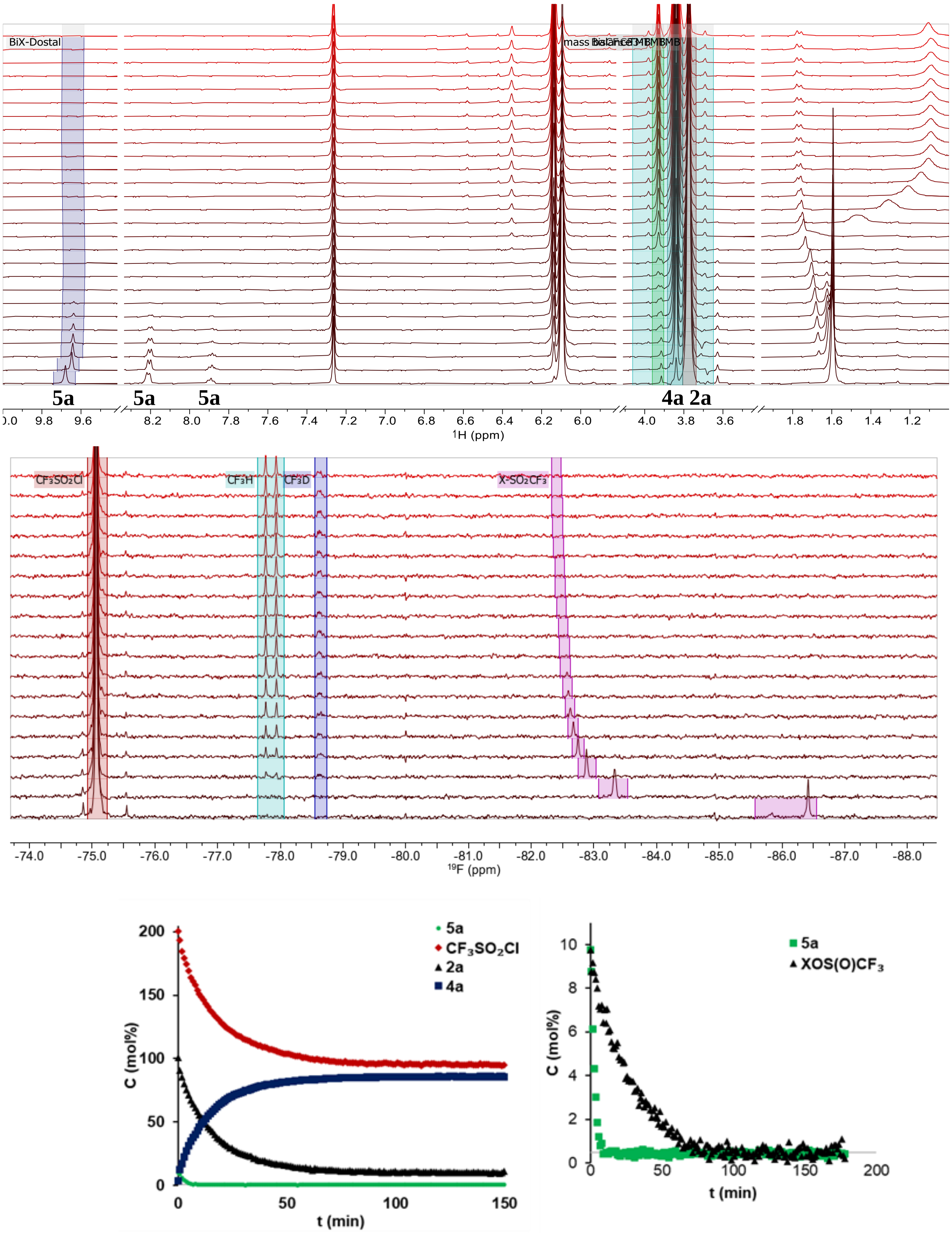

Figure S16. Kinetic profile of the Bi(I)-catalyzed trifluoromethylation in $CDCl_3$ (0.125 M).

In the case of 1,3,5-trimethoxybenzene, the reaction proceeds smoothly without base. However, monitoring the reaction by NMR, we could clearly observe that Bi(III) intermediate **5a** decomposed as the reaction progressed. When **5a** is completely consumed, the reaction still proceeds to certain extent (additional ca 20-25%). After removal of the NMR tube from the spectrometer, a white precipitate accumulated at the bottom of the tube. Based on the reactivity reported by Power in 2000 on $(2,6\text{-}Mes_2C_6H_3)_2BiH$,[25] a possible decomposition pathway based on a competing C–H reductive elimination at the carbon sp^2 on the ligand was speculated. This would lead to demetalated "free" ligand and insoluble bismuth salts. The insoluble white solid could be isolated via filtration and re-dissolved in MeCN-*d*$_3$. NMR analysis revealed the presence of a dicationic diamine (the ^{14}N coupling of proton on imine moieties was visible in ^1H NMR), originating from double protonation of the demetalated ligand (Figure S18). We speculate that due to the absence of base in the system, formation of HCl leads to protodemetalation and the eventually, protonation of the Schiff bases. Interestingly, a CF_3-based peak could also be observed by ^{19}F NMR spectrum (around δ = –83 ppm) after the decomposition of **5a**. This suggests that the unknown Bi salts contain SO_2CF_3 anions which can still be photochemically active and provide CF_3 radical (albeit in lower efficiency).

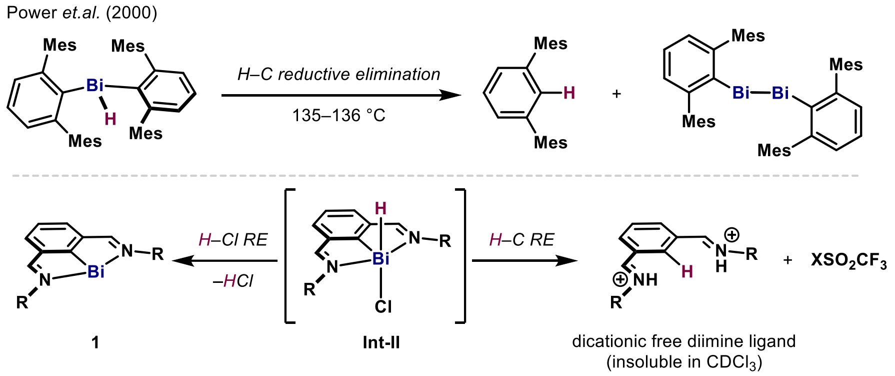

Figure S17. Two distinctive pathways from **Int-II** via H–Cl and H–C reductive elimination

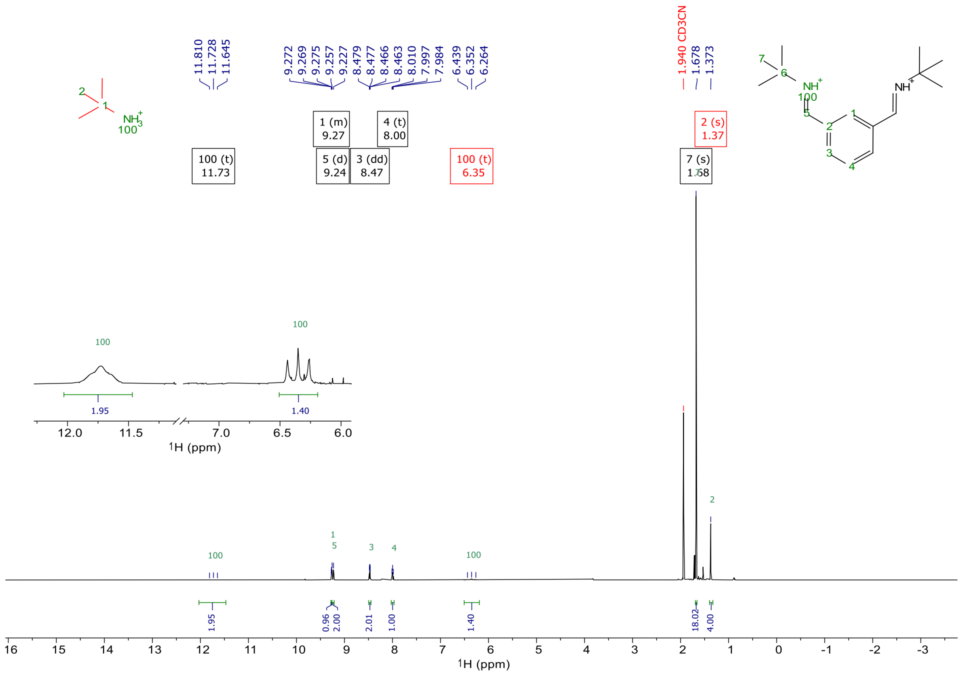

Figure S18. ^1H NMR of insoluble solid in MeCN-*d*$_3$.

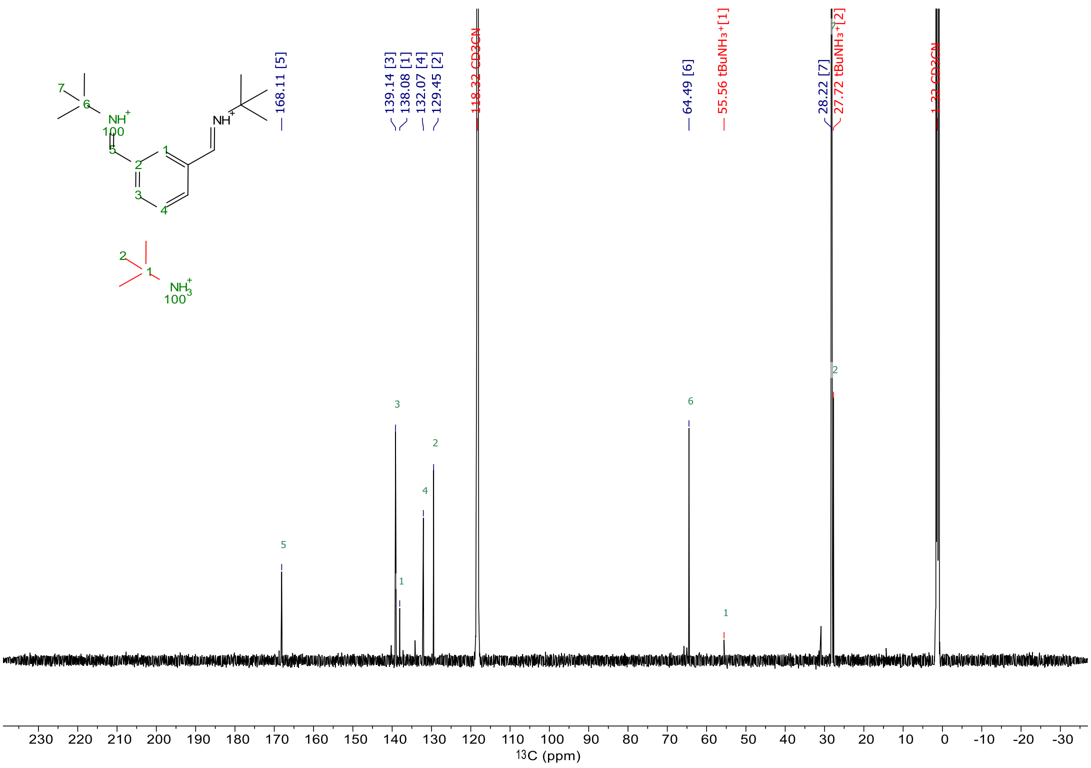

Figure S19. $^{13}C\{^1H\}$ NMR of insoluble solid in MeCN-d_3.

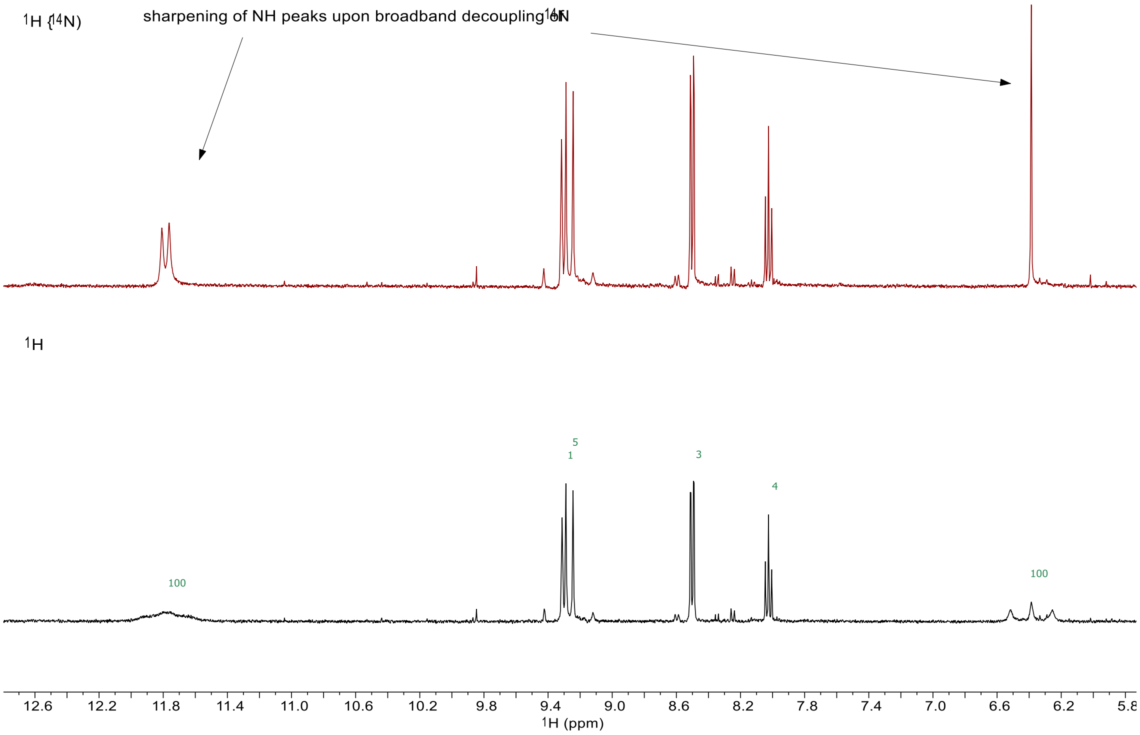

Figure S20. $^1H\{^{14}N\}$ NMR of insoluble solid in MeCN-d_3.

Accordingly, we isolated the corresponding solid and performed the kinetic experiments again. We prepared the white precipitate with 0.020 mmol of **1a**. After confirming the disappearance of peaks derived from *N*,*C*,*N*-pincer ligand by ^{1}H NMR and seeing the white solid crashing out, it was collected and washed with dry $CDCl_3$ under argon atmosphere (8.4 mg). As the reaction was conducted at 0.0050 mmol of catalyst (0.05 mmol scale×10 mol%), we have added 2.1 mg of the solid for kinetic experiment [8.4×(0.0050/0.020)]. As a result, the product **4a** was obtained only in ca 5% in 3 h. Thus, it is unlikely that the solid can catalyze the trifluoromethylation reaction after the decomposition of **5a**.

Based on these results, we cannot discard that an alternative pathway based on Bi species containing SO_2CF_3 anions is also operative and compete with the main pathway *in the absence of base*.

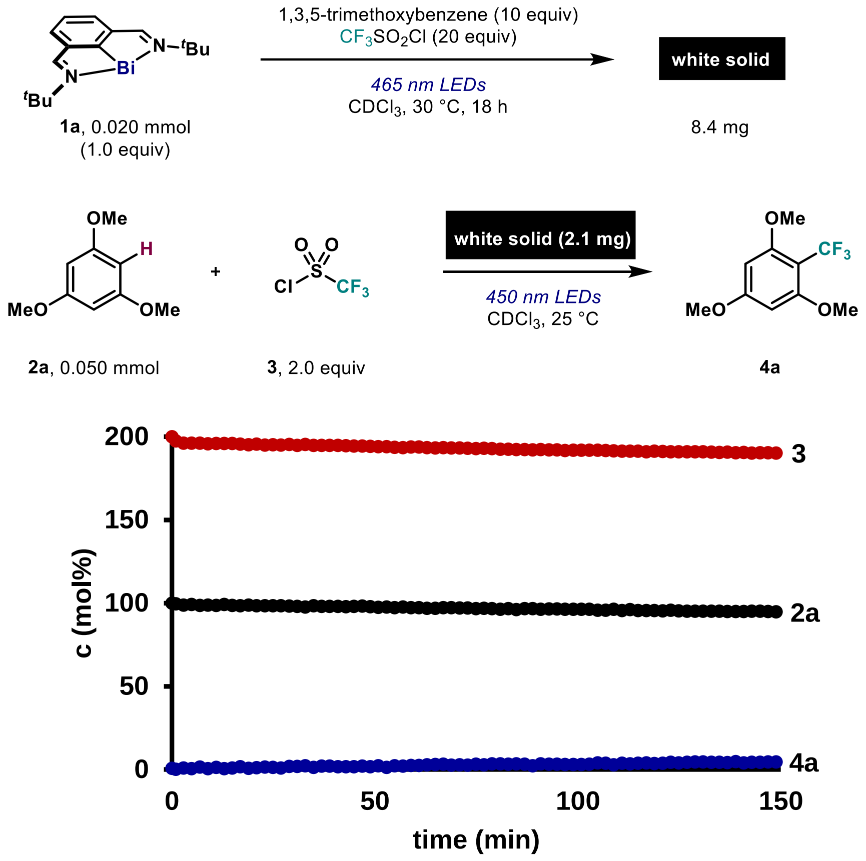

Figure S21. Kinetic profile of trifluoromethylation using the isolated white solid.

To further shed some light into the mechanism, the use of 10 mol% of $BiCl_3$ or $BiCl_3/NaSO_2CF_3$ instead of *N*,*C*,*N*-bismuthinidene afforded **4a** in 8% and 24% yields respectively. Taken this information into consideration, in the absence of base, alternative unidentified $BiX_y(SO_2CF_3)_{3-y}$ species in different oxidation states are able to mediate and/or catalyze the trifluoromethylation reaction after the decomposition of **5a**, thus adding a potential alternative mechanism to the reaction.

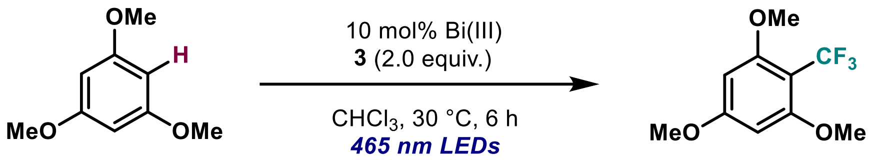

2a

4a
$BiCl_3$; 8%
$BiCl_3/NaSO_2CF_3$; 24%

Figure S22. The reaction using $BiCl_3$ or $BiCl_3/NaSO_2CF_3$ as a catalyst.

7.7.2. Kinetic Analysis with bases

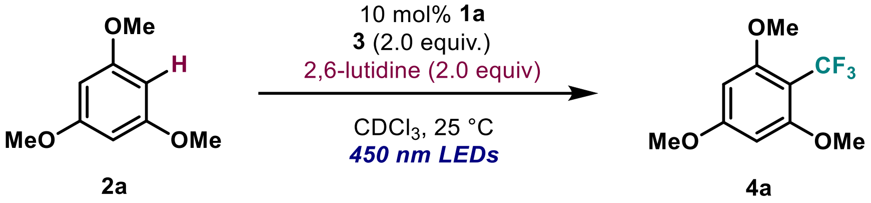

In order to elucidate the role of the base required in certain examples, the catalytic model reaction in the presence of 2,6-lutidine (a soluble organic base) was monitored by ^{1}H NMR. In an argon-filled glove box, **1a** (2.3 mg, 0.0050 mmol, 10 mol%), **2a** (8.4 mg, 0.050 mmol), **3** (11 µL, 0.10 mmol, 2.0 equiv), 2,6-lutidine (12 µL 0.10 mmol, 2.0 equiv), and $CDCl_3$ (0.40 mL, dried and degassed) were placed in an oven-dried NMR tube which can be irradiated via an optical fiber during measurement. The NMR tube was brought to the NMR spectrometer while avoiding ambient light with aluminum foil. ^{1}H and ^{19}F NMR spectra were acquired every 2 minutes under blue light irradiation.

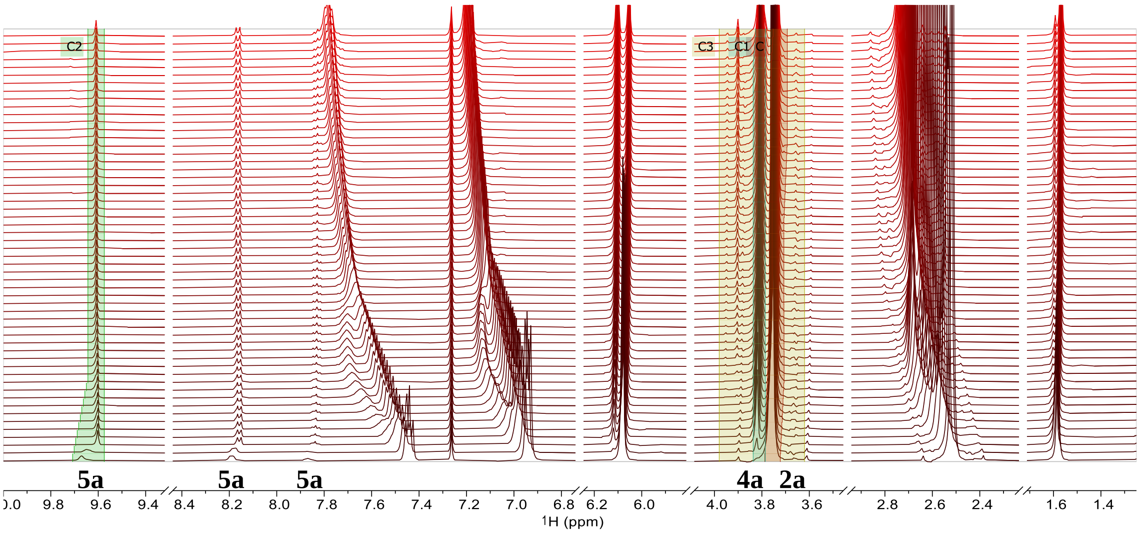

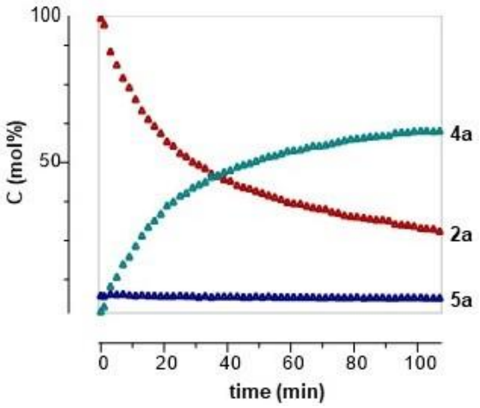

Figure S23. Kinetic profile of the Bi(I)-catalyzed trifluoromethylation with 2,6-lutidine as a base.

Differently, than in the absence of base, **decomposition of the Bi catalyst was not observed** and the concentration of **5a** remained constant during the course of the reaction. Moreover, any other Bi species derived from *N*,*C*,*N*-pincer ligand scaffold was not detected in ^{1}H NMR, thus pointing to the crucial role of lutidine in avoiding decomposition of the pincer Bi complex by quenching the HCl. It is important to

mention that as shown in Figure S23, the reaction rate in the presence of base is lower than in the absence of base. We speculate that the presence of base can interact reversibly with both the oxidative addition complex **5a** and **3**, thus slowing down the catalytic process.

In addition, we performed the same kinetic experiments in the presence of CsF and K_2CO_3, which were employed as bases in the substrate scope. Although **5a** was decomposed completely in 3 h, it was still present in the reaction mixture even after 30 min.

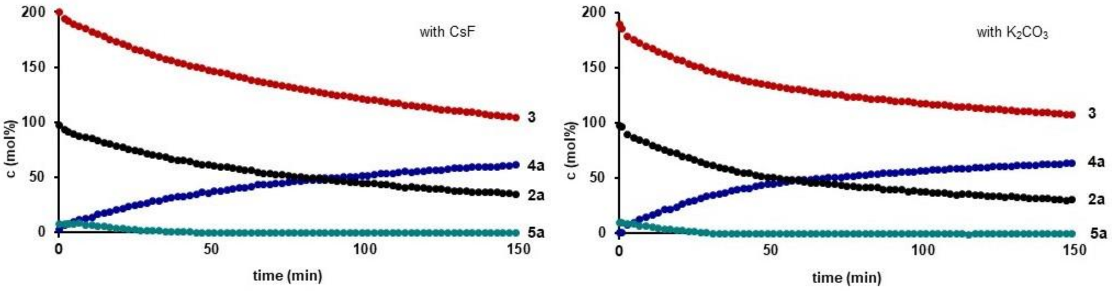

Figure S24. Kinetic profiles of the Bi(I)-catalyzed trifluoromethylation with CsF and K_2CO_3.

At this point, we can certainly state that the additives affect catalyst decomposition. At this moment, we cannot rule out the possibility that catalytically active "Bi-OSOCF$_3$" species or cluster are formed, largely influenced by these bases, engaging with the anions forming unknown bismuth salts. To test a homogeneity of the reaction, we added Hg(0) to our reaction system. The mercury test is often used for discriminating between homogeneous and heterogeneous catalytic system. As a result, the mercury drop slightly inhibited the reactivity, indicating that some sort of Bi cluster might mediate or catalyze the trifluoromethylation reaction after the decomposition of **5a**. However, we cannot exclude the possibility of other inhibitive effect of mercury such as blocking the light penetration and interacting to *N*,*C*,*N*-pincer ligand-ligated Bi center. It is also important to state that if such species exist, and are responsible for some catalytic turnover, they should be active under the irradiation conditions of 450 nm, since on/off experiments stated the need of light. This is unusual for simple bismuth(III) salts without chromophores, which are white powders and require UV-A light to trigger homolysis. Another potential explanation for this behavior is a shift in mechanism, where species of Bi(II) bearing CF_3SO_2 as counterions remain as the resting state of the reaction, prior to complete degradation. These cationic species if monomeric, could be silent by ^{1}H NMR but present a sharp peak on the ^{19}F NMR.[41]

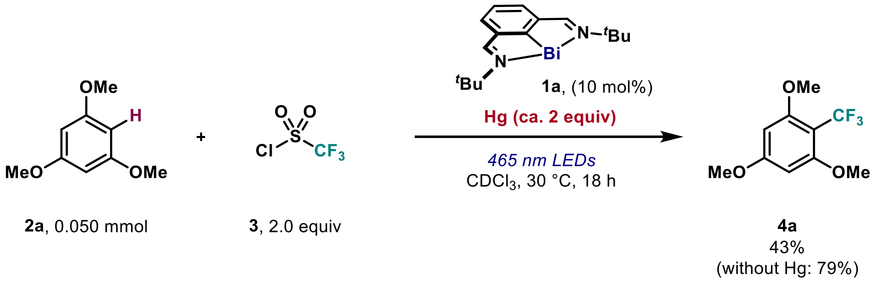

Figure S25. Mercury drop test.

7.7.3. Light On/Off experiment

Additionally, we conducted a light On/Off experiment with the same procedure above. It was found that although the generation of **4a** was occurred in the presence of light, it was stopped when we turned off the light. It suggests that the reaction doesn't proceed through radical-chain mechanism considering the quantum yield together.

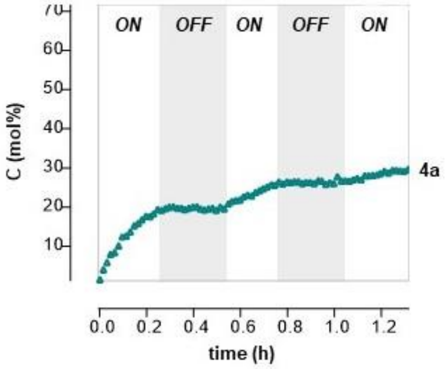

Figure S26. Light-ON/OFF experiment.

7.8. Potential Reactivity of Low-valent Bismuth(I) (**1a**) toward Chloroform

7.8.1. The reaction of **1a** with chloroform

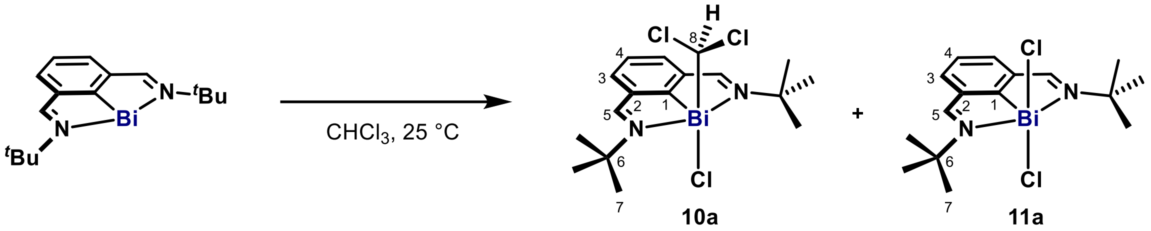

In an argon-filled glovebox, **1a** was placed in an oven-dried vial, and it was sealed with a screw cap containing a Teflon™-coated rubber septum. The vial was taken out of the glovebox and degassed dry $CHCl_3$ was added. After the dark green color of **1a** faded out, the solvent was removed and then the solid was dissolved in degassed dry $CDCl_3$. In ^1H NMR analysis, the peaks of **1a** were disappeared and the two distinct *N*,*C*,*N*-pincer ligand-supported Bi(III) species **10a** and **11a** were newly observed. Therefore, low-valent Bi(I) has a potential to react with chloroform which was used as a reaction mixture for our trifluoromethylation reaction here.

10a

^{1}H NMR (600 MHz, $CDCl_3$) δ 9.75 (s, 2H), 8.10 (d, *J* = 7.5 Hz, 2H), 7.83 (t, *J* = 7.5 Hz, 1H), 6.24 (s, 1H, H-8), 1.61 (s, 18H).

^{13}C{^{1}H} NMR (151 MHz, $CDCl_3$) δ 199.0 (C-1), 167.4 (C-5), 148.0 (C-2), 136.2 (C-3), 129. 5 (C-4), 108.4 (C-8), 61.4 (C-6), 31.3 (C-7)

HRMS (ESI): calc'd for $C_{17}H_{24}N_2Cl_1Bi$ $[M–Cl]^+$: 535.11150, found: 535.1128.

11a

^{1}H NMR (600 MHz, $CDCl_3$) δ 9.61 (s, 2H), 8.17 (d, *J* = 7.5 Hz, 2H), 7.86 (m, 1H), 1.60 (s, 18H).

^{13}C{^{1}H} NMR (151 MHz, $CDCl_3$) δ 209.0 (C-1), 167.0 (C-5), 148.5 (C-2), 136.3 (C-3), 129.7 (C-4), 62.0 (C-6), 30.9 (C-7).

HRMS (ESI): calc'd for $C_{16}H_{23}N_2ClBi$ $[M–Cl]^+$: 487.13483, found: 487.13443.

The spectral data matched with those reported in the literature.[19]

7.8.2. The reaction of **1a** with mixture of **3** and chloroform

Next, we investigated the relative rates of the oxidative addition of **3** and chloroform to **1a**. **3** (2 µL, 0.02 mmol, 2 equiv) and $CDCl_3$ (0.500 mL, 6.23 mmol, 623 equiv. degassed and anhydrous) were added to an oven-dried reaction vial with a screw cap containing a Teflon™-coated rubber septum under argon atmosphere, and the reaction mixture was stirred for 30 minutes. Meanwhile inside an argon-filled glovebox, **1a** (4.5 mg, 0.010 mmol) was placed in an oven-dried normal NMR tube and sealed with the septum cap. Then, the NMR tube was taken out of the glovebox. The prepared mixture of **3** and $CDCl_3$ was added to the NMR tube in which **1a** was. The dark green color of **1a** faded away within 5 seconds and turned to yellow. In ^1H spectrum, **5a** was observed as a major species, not **10a** (**Figure S27**, top, **Figure S28**). This is consistent with the tendency of the redox potential values [$E_{1/2}$(**1a**$^+$/**1a**) = –0.45 vs. SCE, $E_{1/2}$(**3**) = –0.18 vs. SCE, $E_{1/2}$($CHCl_3$)= –1.0 vs. SCE].[7,26,27] Moreover, on thermodynamic ground, the generation of **5a** is favorable [ΔG (ΔH) = –45.7 (–58.7) kcal mol^{-1}] (**Figure S27**, bottom). Additionally, in an actual catalytic reaction, the ratio of **3** to **1** is much higher than that of chloroform (the catalytic reaction: **1**/**3**/$CHCl_3$ = 1 : 20:62, the control experiment: **1**/**3**/$CHCl_3$ = 1 : 2:653) so that the oxidative addition of **3** to **1** is more feasible.

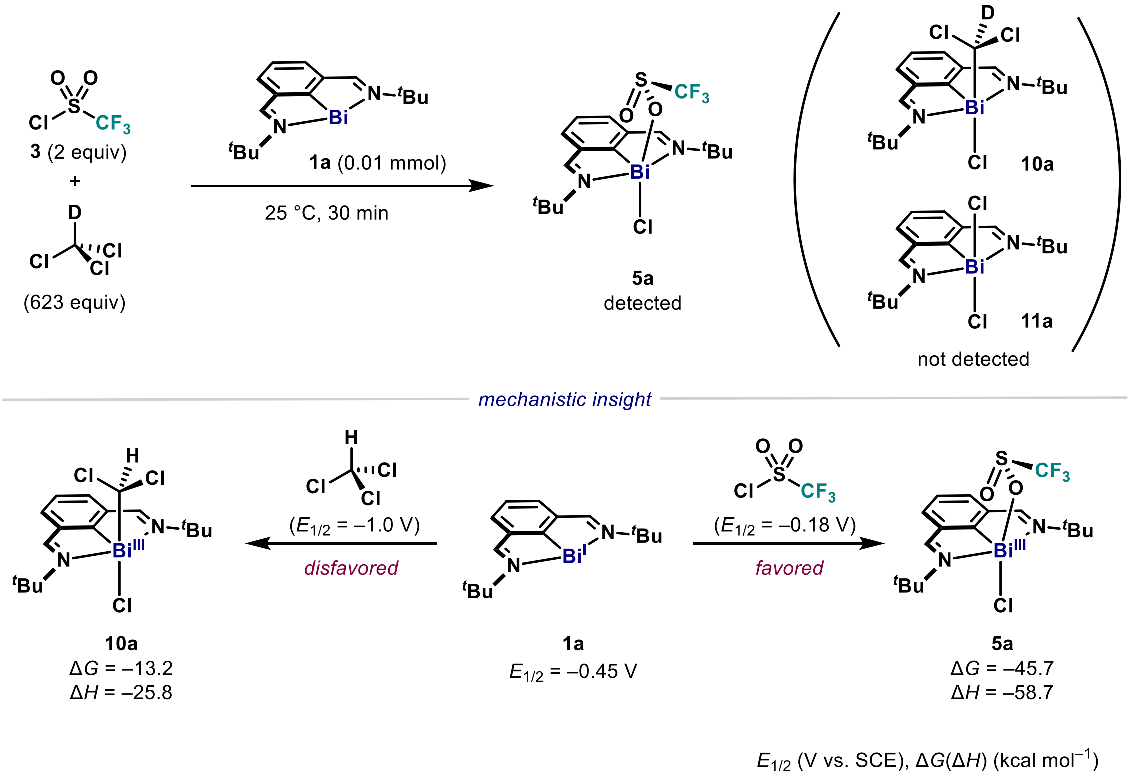

Figure S27. The reaction of **1a** with mixture of **3** and chloroform (top) and mechanistic insight about the feasibility of the oxidative addition of **1a** to **3** or chloroform in terms of the redox potentials and Gibbs free energy (bottom).

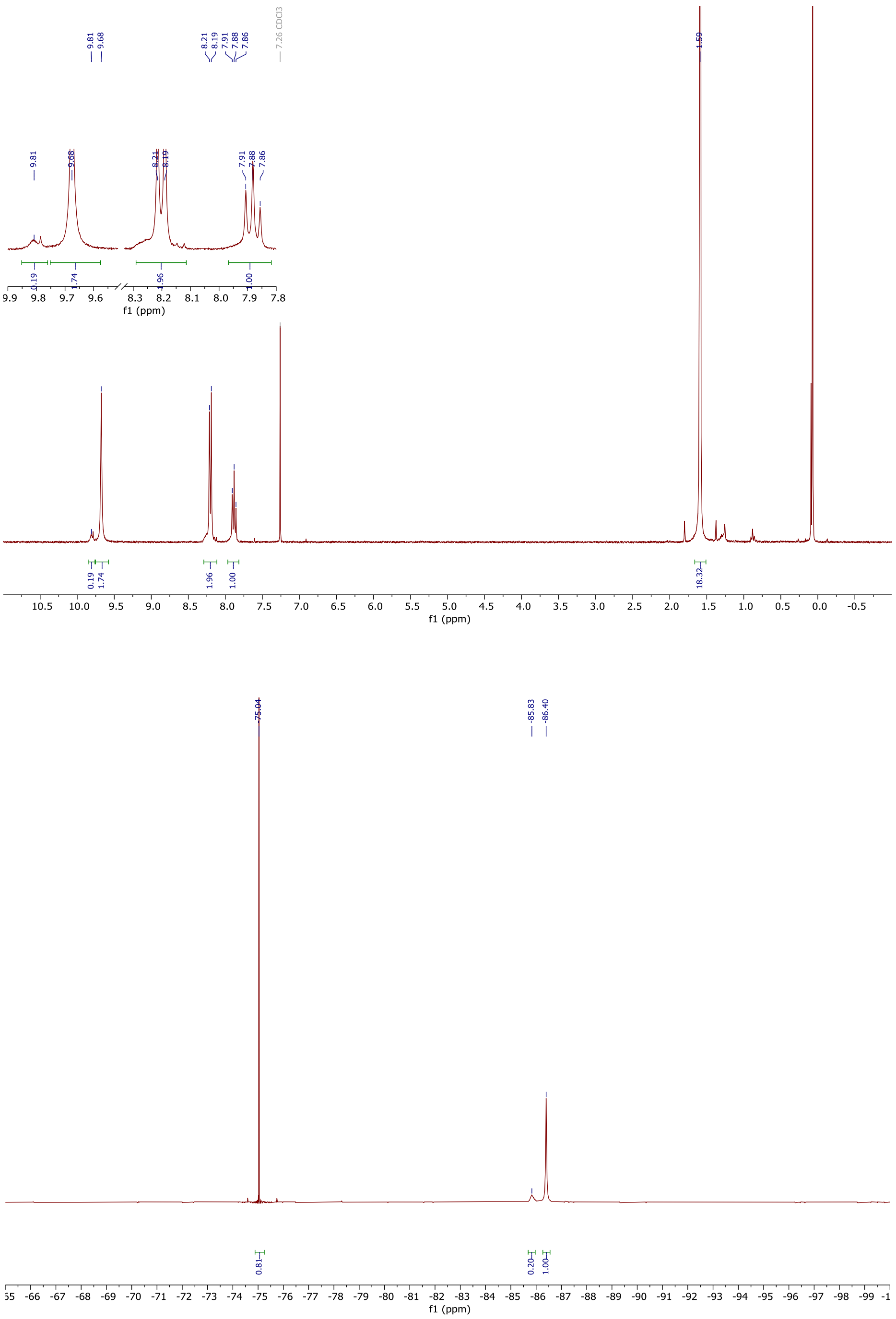

Figure S28. ^{1}H and ^{19}F spectrum of the reaction of **1a** with mixture of **3** and chloroform.

As a side note, we observed another species both in ^{1}H (δ = 9.8, 8.3–8.5 ppm) and ^{19}F (δ = –85.8 ppm) spectrum. Assuming that these peaks correspond to the *N*,*C*,*N*-pincer ligand supported Bi intermediate including two SO_2CF_3 moieties, the integral both in ^{1}H and ^{19}F spectrum (ca. 10 mol% against **5a**) would match very well. Also these species were disappeared as soon as tuning on the blue light. Attempts to detect such species are currently ongoing.

8. Computational Mechanistic Studies

8.1. Computational details

Quantum chemical calculations: All calculations were performed using the development version of ORCA 5.0.1 program suite employing the scalar relativistic zero order regular approximation (ZORA).[28,29] Geometry optimizations were carried out using the BP86 density functional[30] conjunction with the ZORA-Def2-TZVP basis set for H, C, N, O, F, S, and Cl atoms,[31] as well as the SARC-ZORA-TZVP basis for Bi atom, which features a contraction optimized for the ZORA Hamiltonian at 298.15 K.[32] The RI approximation with SARC/J fitting basis set was employed to accelerate the calculations.[32–35] Furthermore, the atom-pairwise D3 dispersion correction with Becke-Johnson (D3BJ) damping was considered.[36,37] Subsequent frequency calculations revealed that all optimized geometries were local minima with no imaginary frequencies, while transition state geometries were verified by only one imaginary frequency. Intrinsic reaction coordinate (IRC) calculations were carried out to confirm the transition states connecting the correct reactants and products on the potential energy surface.[38] Single point energy calculations were carried out at the same level of theory. The Gibbs free energy was calculated by the sum of total electronic energy in the single-point energy calculation and the thermal correction energy in the frequency calculation. The % contributions are calculated by Mulliken population analysis. The Chemcraft 1.8, and Mercury 2020.3.0 software were used to display molecular geometries.

Summary of the level of theory:(U)BP86-D3(BJ)/SARC-ZORA-TZVP(for Bi), Def2-TZVP(for the others)// (U)BP86-D3(BJ)/SARC-ZORA-TZVP(for Bi), Def2-TZVP(for the others).

BDE: The bond dissociation energy (BDE) was calculated by the following fomula.

$$\mathrm{BDE} = \Delta H^0_{\mathrm{Radical}} + \Delta H^0_{\mathrm{H}} - \Delta H^0_{\mathrm{molecule}}$$

Redox potential:The redox potential values of **2u’** and **Int-1** were calculated using BP86 functionals based on ferrocene ($E_{1/2}$ = +0.42 V vs SCE). At the end of the calculations, +0.42 was added to the calculated values to convert it to versus SCE.[39]

8.2. Time-dependent density-functional theory (TD-DFT) calculations

TD-DFT calculations was performed using ORCA 5.1.0 program based on the DFT optimized geometries using the same functional and the basis set (vide supra) with a solvation effect of chloroform modeled by SMD method with 100 roots in total.[40] Although the crystal structure of **5a** is unsymmetrical due to crystal packing (vide infra), the NMR peaks were symmetrical. It is assumed that SO_2CF_3 moiety could coordinate to Bi center through an η^3–coordination mode in solution state. Furthermore, the geometry optimization of **5a** starting from XRD structure converged to the structure in an η^3-fashion. For this reason, the optimized structure of **5a** with η^3-coordination of SO_2CF_3 moiety was used for this calculation.

(A)

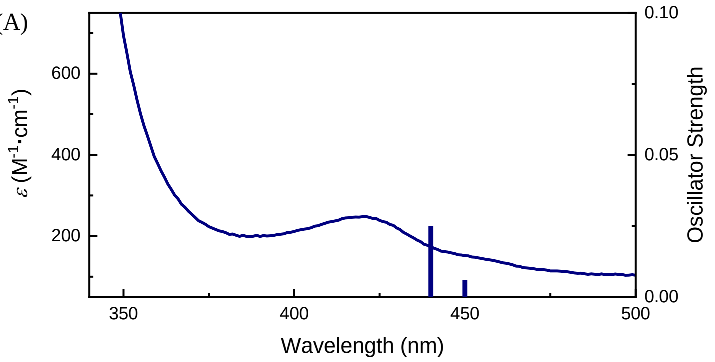

(B)

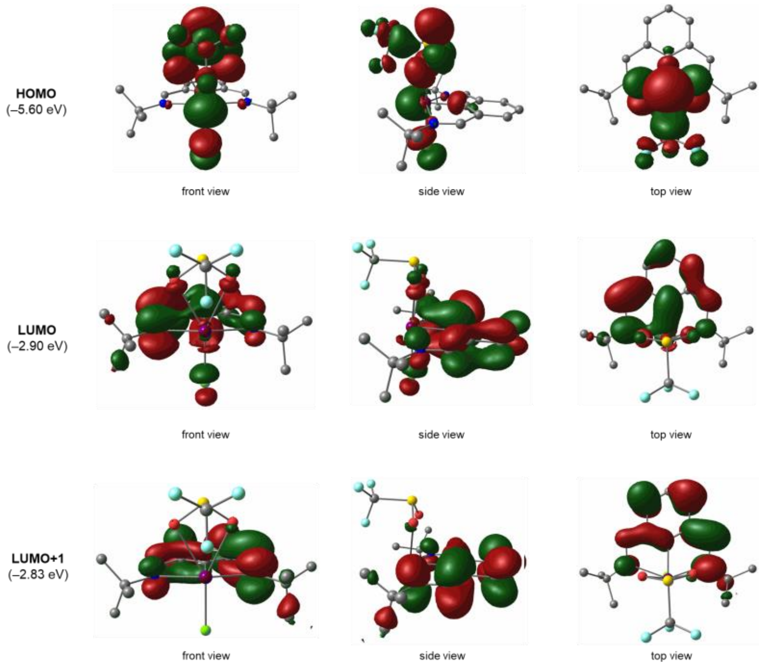

Figure S29. (A) Experimental UV-vis spectrum for **5a** (solid blue line) super-imposed with corresponding TD-DFT excited transitions (blue bars) at the BP86/SARC-ZORA-TZVP (for Bi), ZORA-def2-TZVP (for the others) level of theory. (B) Selected molecular orbital isosurfaces, orbital energies, and the sum of the square of the orbital coefficients for **5a**. Hydrogen atoms are omitted for clarity.

Table S7. The % contribution of each atom in HOMO, LUMO, LUMO+1 of **5a**.

HOMO		LUMO		LUMO+1	
atom	% populations	atom	% populations	atom	% populations
0Bi	9.0	0Bi	7.5	0Bi	1.4
1Cl	12.5	8N	16.7	8N	4.3
2S	17.5	9N	3.1	9N	17.7
6O	20.4	11C	17.9	11C	2.9
7O	20.1	12C	6.1	12C	2.7
10C	10.1	13C	0.9	13C	19.0
		15C	4.7	15C	1.7
		16C	5.7	16C	13.6
		18C	1.3	18C	7.9
		19C	21.0	21C	18.0

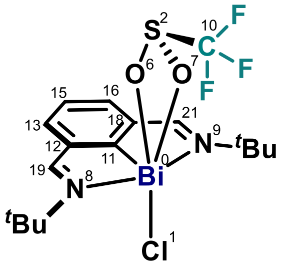

The computed UV/vis absorption spectra showed good agreement with the measured excitation spectra. The computed absorption maximums around 440 and 450 nm are close to the experimental absorption maximums of **5a** at 421 nm, which were assigned as transitions from HOMO to LUMO and HOMO to LUMO+1.

In addition, we carried out Mulliken population analysis to assign % contributions of each atom in MOs (Table S7). Although there is 9% Bi contribution in HOMO, we found that this is mainly a mixture of Rydberg orbitals. This points that the HOMO is essentially the nonbonding orbital of the axial hypervalent bonding of **5a** localized on axial ligands [20.4% O(6); 20.1% O(7); 12.5% Cl(1)]. By contrast, the LUMO and LUMO+1 can be described as π* orbitals delocalized throughout the *N*,*C*,*N*-pincer ligand. Thus, the transitions around 450 nm can be described as a *ligand-to-ligand charge transfer (LLCT)* which reduces the electron density of the hypervalent bonding orbitals and induces Bi–O bond homolysis.

Although LLCT would deliver the electron in the π* system, placing the electron on the Bi center is the most energetically favorable configuration.[41] However, we cannot discard that electron transfer occurs through the delocalized system, akin TM-based open-shell species.

8.3. Computational Study of Re-aromatization Process via HAT

To probe the feasibility of HAT process to obtain a trifluoromethylated product, we conducted a series of DFT calculations. The formation of singlet-state diradical **Int-Ia'** is exergonic (ΔG = –8.3 kcal mol^{-1}, ΔH = –23.5 kcal mol^{-1}). Subsequently, there is transition state **TS2a** with a low activation energy barrier, in which a hydrogen atom of **2u'** is transferred to the Bi(II) radical to form a Bi–H intermediate **Int-IIa** through re-aromatization ($\Delta G^{\ddagger}$ = +2.9 kcal mol^{-1}, $\Delta H^{\ddagger}$ = +2.3 kcal mol^{-1}). This pathway would be feasible because of the acquisition of aromatic stabilization and the transformation to stable Bi(III) intermediate. Moreover, in terms of bond dissociation enthalpy (BDE), the C–H abstraction in **2u'** [$BDE_{C–H}$(**2u'**) = +25.2 kcal mol^{-1}] en route to Bi–H bond [$BDE_{Bi–H}$(**Int-IIa**) = +44.6 kcal mol^{-1}] is favorable.

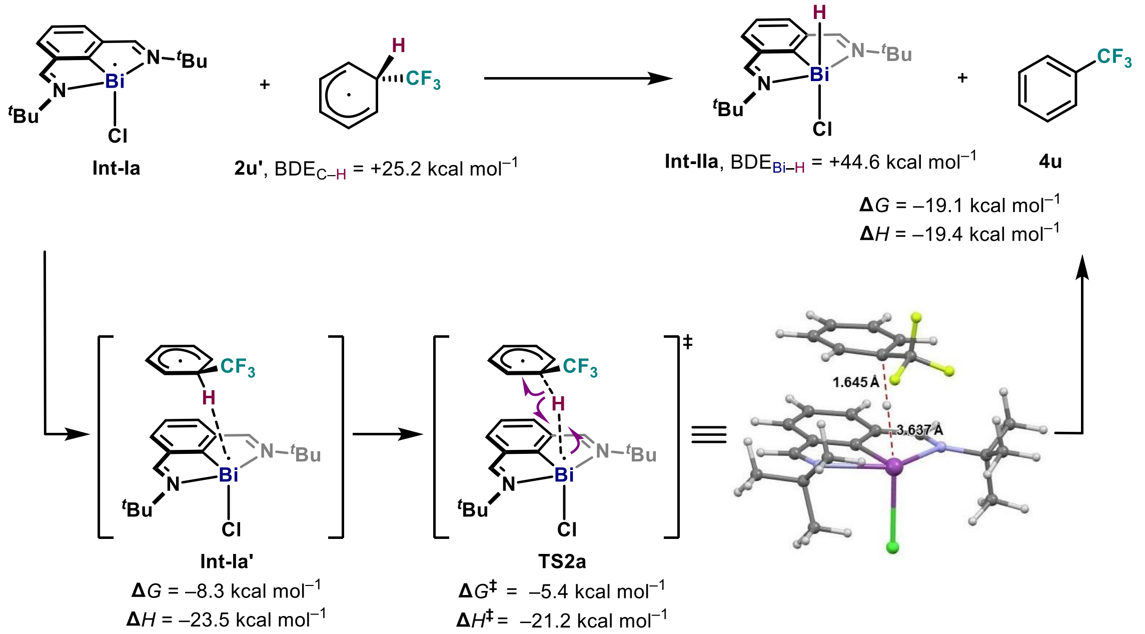

Figure S30. Postulated pathway for the CF_3 radical addition to arenes, followed by a re-aromatizing HAT process.

8.4. XYZ coordinates of DFT optimized structures

1a

Bi	-0.160087000	0.104737000	-0.566431000
N	-2.529495000	-0.338091000	-0.001543000
N	2.077923000	0.486383000	0.421175000
C	-0.340569000	0.014072000	1.578712000
C	-1.603752000	-0.228018000	2.175039000
C	-1.709027000	-0.283257000	3.574612000
H	-2.681662000	-0.469404000	4.038091000
C	-0.576840000	-0.101359000	4.377364000
H	-0.668462000	-0.146009000	5.462907000
C	0.672455000	0.137711000	3.793049000
H	1.552280000	0.279097000	4.426598000
C	0.803385000	0.198031000	2.395779000
C	-2.722509000	-0.405394000	1.289007000
H	-3.717438000	-0.594849000	1.715194000
C	2.052884000	0.442106000	1.726810000
H	2.961591000	0.586882000	2.327386000
C	-3.667942000	-0.516845000	-0.934780000
C	-4.293071000	-1.907977000	-0.735089000
H	-3.530884000	-2.688857000	-0.865195000
H	-5.094952000	-2.075156000	-1.468399000
H	-4.725342000	-2.012388000	0.269656000
C	-3.121239000	-0.404990000	-2.362270000
H	-2.664165000	0.581949000	-2.525704000
H	-3.928452000	-0.535681000	-3.096262000
H	-2.359360000	-1.176424000	-2.546146000
C	-4.710536000	0.589470000	-0.699512000
H	-5.144625000	0.523833000	0.307919000
H	-5.528638000	0.503813000	-1.428928000
H	-4.244903000	1.578727000	-0.809630000
C	3.355180000	0.731181000	-0.291661000
C	3.955725000	2.075684000	0.152191000
H	3.234317000	2.887443000	-0.016032000
H	4.868730000	2.294865000	-0.419838000
H	4.219716000	2.065626000	1.218667000
C	4.330646000	-0.424067000	-0.008381000
H	4.588661000	-0.475670000	1.058616000
H	5.260882000	-0.288540000	-0.578516000
H	3.877411000	-1.382009000	-0.299133000
C	3.047085000	0.782349000	-1.792106000
H	2.603793000	-0.166868000	-2.127074000
H	3.965015000	0.955786000	-2.370771000
H	2.339743000	1.594441000	-2.014338000

CF_3SO_2Cl

S	1.301257000	-0.067792000	-0.659984000
O	1.693319000	-1.452166000	-0.740347000
O	1.810924000	0.954350000	-1.538672000
Cl	1.539829000	0.569994000	1.298453000
C	-0.605098000	-0.006754000	-0.755229000
F	-1.031926000	1.237559000	-0.531734000
F	-0.939100000	-0.390937000	-1.993304000
F	-1.130139000	-0.844058000	0.141222000

CH_3Cl

C	1.413902000	5.207957000	1.704411000
H	0.581575000	4.512798000	1.583405000
Cl	0.845346000	6.564257000	2.709551000
Cl	1.893968000	5.766151000	0.082354000
Cl	2.742479000	4.329169000	2.501700000

5a

Bi	4.618111000	5.264583000	3.151597000
Cl	4.382854000	6.042602000	0.680032000
S	5.119243000	4.170344000	6.215715000
F	5.878367000	6.265090000	7.792860000
F	3.727052000	5.848417000	7.868618000
F	4.585772000	6.862101000	6.121396000
O	6.228457000	4.554568000	5.245972000
O	3.821196000	4.019253000	5.435121000
N	7.007681000	4.856194000	2.520308000
N	2.327742000	4.177004000	2.926604000
C	4.806761000	5.894179000	7.054159000
C	4.854164000	3.146377000	2.495629000
C	6.125174000	2.672604000	2.175201000
C	6.285747000	1.328456000	1.793959000
H	7.276806000	0.942183000	1.545036000
C	5.169544000	0.489653000	1.729749000
C	3.894360000	0.979155000	2.028761000
H	3.025819000	0.319930000	1.961008000
C	3.733418000	2.323522000	2.408024000
C	7.233027000	3.628379000	2.230477000
H	8.246660000	3.262249000	2.010108000
C	2.424977000	2.921464000	2.688505000
H	1.544551000	2.264235000	2.673380000
C	8.083347000	5.875591000	2.575761000
C	8.478133000	6.211799000	1.128134000
H	7.592765000	6.523958000	0.557785000
H	9.214309000	7.027747000	1.121080000
H	8.923747000	5.338972000	0.629768000
C	9.278631000	5.359127000	3.389464000
H	9.778135000	4.511838000	2.898787000
H	10.023393000	6.159038000	3.501079000

H	8.945531000	5.044515000	4.387794000
C	7.491194000	7.114698000	3.257977000
H	7.145571000	6.865846000	4.271068000
H	8.248195000	7.906702000	3.335383000
H	6.650542000	7.511566000	2.669104000
C	1.049282000	4.869173000	3.182710000
C	0.729716000	5.664420000	1.903751000
H	0.529316000	4.982830000	1.065394000
H	1.575672000	6.304303000	1.619347000
C	-0.098502000	3.912936000	3.525205000
H	0.143972000	3.301569000	4.406445000
H	-0.998711000	4.498197000	3.754341000
H	-0.348890000	3.246845000	2.686946000
C	1.292105000	5.831409000	4.356498000
H	2.050281000	6.583488000	4.092369000
H	0.364308000	6.367297000	4.598517000
H	1.642838000	5.288986000	5.243231000
H	5.292896000	-0.552984000	1.436021000
H	-0.156850000	6.292657000	2.068781000

10a

Bi	3.598084000	7.206187000	6.476925000
N	1.624428000	8.442845000	7.416139000
N	4.271502000	4.982079000	5.538072000
C	1.855686000	5.842386000	6.539700000
C	0.654001000	6.292313000	7.084409000
C	0.924541000	3.632808000	6.233350000
H	1.029693000	2.595229000	5.907982000
C	2.008097000	4.521411000	6.121162000
C	0.591389000	7.686927000	7.517361000
H	-0.357804000	8.060079000	7.924543000
C	1.642637000	9.870238000	7.788430000
C	2.954344000	10.106040000	8.553805000
H	3.002066000	9.474228000	9.450802000
H	3.033462000	11.159763000	8.853466000
H	3.821382000	9.873879000	7.917168000
C	0.452711000	10.280263000	8.663573000
H	-0.503307000	10.189886000	8.128523000
H	0.564266000	11.333218000	8.954329000
H	0.406402000	9.675362000	9.580748000
C	1.643894000	10.665006000	6.470448000
H	2.449893000	10.329150000	5.803960000
H	1.772951000	11.736210000	6.681072000
H	0.696778000	10.519592000	5.933458000
C	3.311123000	4.130952000	5.586655000
H	3.437649000	3.098880000	5.233483000
C	5.607857000	4.691996000	4.984523000
C	5.691205000	5.430297000	3.637166000
H	4.986127000	4.996089000	2.915554000

H	6.709772000	5.353314000	3.231027000
H	5.427054000	6.490671000	3.748872000
C	5.870534000	3.193521000	4.797541000
H	5.756727000	2.649331000	5.746332000
H	6.899679000	3.047966000	4.443364000
H	5.201430000	2.747688000	4.047892000
C	6.627105000	5.276078000	5.975888000
H	6.488488000	6.362774000	6.080134000
H	7.649255000	5.104177000	5.612417000
H	6.516298000	4.814791000	6.966296000
C	-0.428661000	5.402700000	7.196423000
H	-1.376545000	5.742765000	7.619888000
C	-0.286980000	4.079484000	6.768588000
H	-1.126186000	3.389464000	6.857581000
Cl	2.794144000	7.944572000	3.985741000
C	3.936314000	6.175108000	8.807344000
Cl	2.639664000	6.577390000	10.000765000
Cl	4.231737000	4.392635000	8.830256000
H	4.848719000	6.610742000	9.234355000

Int-Ia

Bi	3.449821000	6.229714000	7.029191000
N	1.443877000	7.679237000	7.605913000
N	4.136093000	4.289492000	5.738920000
C	1.665696000	5.396084000	6.092512000
C	0.415608000	6.027889000	6.242462000
C	0.673490000	3.745394000	4.612213000
H	0.768554000	2.868665000	3.966547000
C	1.809997000	4.264630000	5.258632000
C	0.359911000	7.233356000	7.046919000
H	-0.602750000	7.749396000	7.152818000
C	1.495601000	8.933432000	8.381748000
C	2.316217000	8.635783000	9.647490000
H	1.829534000	7.858836000	10.253696000
H	2.422720000	9.542857000	10.258616000
H	3.324362000	8.286307000	9.381808000
C	0.113496000	9.468091000	8.779473000
H	-0.479891000	9.764526000	7.903006000
H	0.238176000	10.360736000	9.407014000
H	-0.455614000	8.724081000	9.356063000
C	2.231799000	9.969604000	7.513268000
H	3.210017000	9.590841000	7.188146000
H	2.379308000	10.900211000	8.080154000
H	1.650724000	10.196467000	6.608832000
C	3.138455000	3.734053000	5.093619000
H	3.299237000	2.890535000	4.410921000
C	5.536384000	3.879854000	5.475710000
C	5.917640000	4.350140000	4.060174000
H	5.315123000	3.832390000	3.300331000
H	6.978505000	4.140796000	3.860981000

H	5.741529000	5.430359000	3.965703000
C	5.697767000	2.357982000	5.615755000
H	5.388548000	2.026427000	6.617301000
H	6.749856000	2.077448000	5.466657000
H	5.101258000	1.813162000	4.871370000
C	6.429356000	4.586409000	6.502355000
H	6.362285000	5.678790000	6.387547000
H	7.479790000	4.299619000	6.356190000
H	6.139824000	4.313263000	7.528044000
C	-0.705022000	5.495489000	5.589024000
H	-1.679830000	5.977288000	5.698435000
C	-0.573189000	4.353545000	4.784344000
H	-1.447984000	3.946719000	4.276500000
Cl	4.382332000	7.852432000	5.142534000

2u’

C	-23.005216000	10.501920000	3.962985000
C	-24.216458000	10.462381000	4.696280000
C	-21.788433000	10.510630000	4.585688000
H	-25.173077000	10.451047000	4.175609000
H	-20.864723000	10.541272000	4.008150000
C	-24.174163000	10.439214000	6.111949000
C	-21.667138000	10.480789000	6.083579000
H	-25.110609000	10.419022000	6.672658000
C	-22.990463000	10.445483000	6.795397000
H	-22.973617000	10.428131000	7.884978000
H	-23.043526000	10.529293000	2.872333000
C	-20.763396000	9.315389000	6.539385000
F	-20.578440000	9.333286000	7.888988000
F	-21.277815000	8.104832000	6.217551000
F	-19.528891000	9.398142000	5.968746000
H	-21.091838000	11.371095000	6.423182000

Int-Ia’

Bi	0.857900000	6.805294000	8.387688000
N	0.490947000	4.236547000	8.501534000
N	2.086514000	8.478057000	7.215122000
C	1.851844000	5.823498000	6.697430000
C	1.801176000	4.433134000	6.526017000
C	3.321302000	6.012705000	4.770141000
H	3.939644000	6.619182000	4.105707000
C	2.640159000	6.622467000	5.839959000
C	1.051710000	3.650629000	7.500937000
H	0.992628000	2.563081000	7.353752000
C	-0.286624000	3.534161000	9.536539000
C	0.403975000	3.837171000	10.877993000
H	1.425337000	3.433069000	10.890597000
H	-0.162234000	3.390090000	11.706882000
H	0.464873000	4.921001000	11.049767000
C	-0.374165000	2.018593000	9.324991000

H	-0.865397000	1.769379000	8.373224000
H	-0.969282000	1.573260000	10.133282000
H	0.618994000	1.546077000	9.342901000
C	-1.695537000	4.151355000	9.518400000
H	-1.653397000	5.237593000	9.677027000
H	-2.311981000	3.707171000	10.312495000
H	-2.184894000	3.980047000	8.550414000
C	2.742590000	8.016390000	6.174255000
H	3.321087000	8.685371000	5.527396000
C	2.034138000	9.933369000	7.501779000
C	1.218807000	10.610741000	6.386340000
H	1.725996000	10.516464000	5.415652000
H	1.087902000	11.680389000	6.603660000
H	0.229205000	10.140279000	6.307052000
C	3.448380000	10.527025000	7.594604000
H	4.017268000	10.041562000	8.399109000
H	3.387928000	11.601719000	7.815526000
H	4.003323000	10.412114000	6.653560000
C	1.315677000	10.121732000	8.842550000
H	0.287602000	9.731849000	8.791705000
H	1.253165000	11.189361000	9.093153000
H	1.857397000	9.614248000	9.653883000
C	2.488274000	3.839925000	5.457623000
H	2.448179000	2.757053000	5.318997000
C	3.237236000	4.634742000	4.577748000
H	3.771847000	4.170701000	3.749266000
Cl	-1.420306000	7.409285000	7.262643000
H	3.270381000	6.197903000	8.276253000
C	4.430521000	6.108175000	8.460209000
C	4.490699000	6.506735000	9.934619000
F	5.737589000	6.432226000	10.454906000
F	4.052234000	7.788805000	10.129036000
F	3.687696000	5.706762000	10.701071000
C	4.785561000	4.684415000	8.231058000
H	4.357381000	3.940547000	8.902713000
C	5.560375000	4.305634000	7.171662000
H	5.747112000	3.242277000	7.008835000
C	6.141994000	5.252536000	6.291528000
H	6.775122000	4.922415000	5.469261000
C	5.945440000	6.613302000	6.539172000
H	6.436590000	7.353808000	5.903941000
C	5.132080000	7.058306000	7.566105000
H	5.047911000	8.121014000	7.780553000

TS2a

Bi	0.963379000	6.781156000	8.342063000
N	0.739288000	4.217867000	8.393764000
N	2.071300000	8.565692000	7.072959000
C	1.835078000	5.892946000	6.507335000

C	1.826319000	4.507327000	6.300623000
C	3.130395000	6.196391000	4.478191000
H	3.655114000	6.850377000	3.777816000
C	2.512886000	6.738379000	5.617532000
C	1.188444000	3.678241000	7.313877000
H	1.111761000	2.597910000	7.127389000
C	0.040704000	3.471155000	9.455016000
C	0.797929000	3.759731000	10.761355000
H	1.821195000	3.363009000	10.714680000
H	0.280218000	3.295692000	11.612097000
H	0.861864000	4.840891000	10.944124000
C	-0.026902000	1.959870000	9.209906000
H	-0.582415000	1.719163000	8.291964000
H	-0.548816000	1.479710000	10.048230000
H	0.976403000	1.513935000	9.142044000
C	-1.380874000	4.056996000	9.530445000
H	-1.350323000	5.138120000	9.724137000
H	-1.945161000	3.576217000	10.341753000
H	-1.917265000	3.902762000	8.584663000
C	2.574617000	8.148035000	5.960979000
H	3.053645000	8.840384000	5.253650000
C	2.044894000	10.012403000	7.416810000
C	1.203349000	10.750555000	6.362134000
H	1.685244000	10.723266000	5.374180000
H	1.081954000	11.803894000	6.651695000
H	0.213891000	10.278597000	6.287708000
C	3.479910000	10.556645000	7.478973000
H	4.062913000	10.020321000	8.240037000
H	3.462677000	11.622349000	7.745641000
H	3.992387000	10.460913000	6.511394000
C	1.373799000	10.145445000	8.787934000
H	0.337989000	9.778498000	8.739793000
H	1.348835000	11.199173000	9.097322000
H	1.930666000	9.582501000	9.550959000
C	2.446180000	3.972170000	5.160340000
H	2.435399000	2.893027000	4.989250000
C	3.091716000	4.818184000	4.253307000
H	3.571399000	4.399395000	3.368535000
Cl	-1.218985000	7.873107000	7.028358000
H	2.898889000	6.379066000	8.597953000
C	4.488539000	6.065041000	8.879141000
C	4.323969000	6.693969000	10.248369000
F	5.491148000	6.651439000	10.954730000
F	3.965435000	8.009194000	10.186177000
F	3.391774000	6.060351000	11.014420000
C	4.536350000	4.643940000	8.798139000
H	4.111728000	4.051211000	9.607618000
C	5.038914000	4.029308000	7.662574000
H	5.014953000	2.940745000	7.590047000

C	5.594655000	4.785549000	6.621088000
H	5.990479000	4.293740000	5.733445000
C	5.670319000	6.176140000	6.753150000
H	6.140986000	6.771201000	5.968923000
C	5.167329000	6.814621000	7.878237000
H	5.239829000	7.896568000	7.978379000

Int-IIa

Bi	3.498898000	6.938705000	6.759811000
N	1.508547000	8.197806000	7.865344000
N	4.079678000	4.718719000	5.847773000
C	1.667994000	5.663424000	6.779170000
C	0.454889000	6.145350000	7.274813000
C	0.621625000	3.544773000	6.238617000
H	0.685942000	2.532541000	5.832430000
C	1.762331000	4.366741000	6.266174000
C	0.426201000	7.509783000	7.798755000
H	-0.543387000	7.917256000	8.119887000
C	1.564730000	9.600089000	8.312708000
C	2.687237000	9.675255000	9.360235000
H	2.450549000	9.050960000	10.232590000
H	2.826566000	10.712437000	9.694901000
H	3.637052000	9.320404000	8.935488000
C	0.249659000	10.113234000	8.909948000
H	-0.562438000	10.113515000	8.168735000
H	0.384627000	11.148246000	9.250831000
H	-0.063058000	9.511725000	9.776212000
C	1.941676000	10.433014000	7.073075000
H	2.882344000	10.078241000	6.630126000
H	2.065088000	11.488628000	7.352831000
H	1.161971000	10.358665000	6.303266000
C	3.060263000	3.941269000	5.756818000
H	3.130385000	2.958333000	5.268709000
C	5.395017000	4.349765000	5.260362000
C	5.214456000	3.981564000	3.778974000
H	4.623126000	3.062888000	3.658275000
H	6.196955000	3.809416000	3.317700000
H	4.706282000	4.805106000	3.257851000
C	5.983031000	3.182599000	6.069267000
H	6.068211000	3.452568000	7.131047000
H	6.983596000	2.932400000	5.689886000
H	5.353173000	2.285179000	5.988891000
C	6.302085000	5.580156000	5.369505000
H	5.873696000	6.415559000	4.796402000
H	7.298128000	5.354472000	4.964887000
H	6.426794000	5.878748000	6.422022000
C	-0.685623000	5.322358000	7.242736000
H	-1.640864000	5.696931000	7.618690000
C	-0.595879000	4.026899000	6.726672000

H	-1.480735000	3.390648000	6.701288000
Cl	3.164394000	7.182953000	3.945412000
H	3.854507000	6.015121000	8.417784000

4u

C	-29.285082000	19.780213000	-3.366574000
C	-30.440569000	19.423875000	-2.666478000
C	-28.121822000	20.115935000	-2.670093000
H	-31.349243000	19.158560000	-3.207898000
H	-27.217774000	20.391278000	-3.214244000
C	-30.435693000	19.403135000	-1.271980000
C	-28.110320000	20.097614000	-1.274956000
H	-31.332372000	19.121319000	-0.720563000
H	-27.204552000	20.354153000	-0.726527000
C	-29.268224000	19.738762000	-0.577912000
H	-29.290861000	19.793967000	-4.457251000
C	-29.280957000	19.765714000	0.928265000
F	-29.723984000	20.968558000	1.404935000
F	-30.102821000	18.815159000	1.452675000
F	-28.045124000	19.567179000	1.461323000

9

C	-1.224096000	0.724037000	-0.000055000
C	-1.224108000	-0.724016000	-0.000057000
C	1.224092000	0.724015000	-0.000068000
C	1.224079000	-0.724037000	-0.000070000
C	0.000004000	1.405871000	-0.000061000
C	-0.000020000	-1.405872000	-0.000064000
C	-2.477052000	1.407665000	-0.000048000
C	-2.477076000	-1.407622000	-0.000051000
C	2.477059000	1.407623000	-0.000074000
C	2.477035000	-1.407665000	-0.000077000
C	-3.661095000	0.712082000	-0.000042000
C	-3.661107000	-0.712019000	-0.000044000
C	3.661090000	0.712020000	-0.000081000
C	3.661078000	-0.712082000	-0.000083000
H	0.000013000	2.498891000	-0.000060000
H	-0.000030000	-2.498892000	-0.000066000
H	-2.474417000	2.499661000	-0.000046000
H	-2.474459000	-2.499618000	-0.000053000
H	2.474443000	2.499618000	-0.000073000
H	2.474400000	-2.499661000	-0.000079000
H	-4.610943000	1.248628000	-0.000036000
H	-4.610964000	-1.248549000	-0.000039000
H	4.610948000	1.248549000	-0.000085000
H	4.610927000	-1.248627000	-0.000088000

9,10-dihydro-9-anthryl

C	-1.245780000	0.253244000	1.275233000

C	-1.278574000	-0.822253000	0.340796000
C	1.245779000	0.253235000	1.275209000
C	1.278548000	-0.822262000	0.340772000
C	0.000005000	0.797919000	1.690100000
C	-0.000021000	-1.316945000	-0.289116000
C	-2.473874000	0.738784000	1.794766000
C	-2.505479000	-1.378411000	-0.017533000
C	2.473886000	0.738766000	1.794719000
C	2.505442000	-1.378428000	-0.017581000
C	-3.683820000	0.176697000	1.417398000
C	-3.705760000	-0.892669000	0.510018000
C	3.683820000	0.176671000	1.417328000
C	3.705736000	-0.892695000	0.509948000
H	0.000015000	1.626231000	2.401894000
H	-0.000025000	-2.419646000	-0.318458000
H	-2.449165000	1.564589000	2.508909000
H	-2.525647000	-2.207895000	-0.728987000
H	2.449195000	1.564572000	2.508863000
H	2.525590000	-2.207912000	-0.729036000
H	-4.617208000	0.562664000	1.829749000
H	-4.653686000	-1.344296000	0.215470000
H	4.617220000	0.562631000	1.829661000
H	4.653653000	-1.344329000	0.215381000
H	-0.000031000	-1.008087000	-1.353709000

9. References

(1) Vránová, I.; Alonso, M.; Lo, R.; Sedlák, R.; Jambor, R.; Růžička, A.; De Proft, F.; Hobza, P.; Dostál, L. From Dibismuthenes to Three- and Two-Coordinated Bismuthinidenes by Fine Ligand Tuning: Evidence for Aromatic BiC_3N Rings through a Combined Experimental and Theoretical Study. *Eur. J. Org. Chem.* **2015**, *21*, 16917–16928.

(2) Wang, F.; Planas, O.; Cornella, J. Bi(I)-Catalyzed Transfer-Hydrogenation with Ammonia-Borane. *J. Am. Chem. Soc.* **2019**, *141*, 4235–4240.

(3) Pang, Y.; Leutzsch, M.; Nöthling, N.; Cornella, J. Catalytic Activation of N_2O at a Low-Valent Bismuth Redox Platform. *J. Am. Chem. Soc.* **2020**, *142*, 19473–19479.

(4) Jia, T.; Xu, S.-Y.; Huang, L.-C.; Gao, W. Scandium and Gadolinium Complexes with Aryldiimine NCN Pincer Ligands: Synthesis, Characterization, and Catalysis on Isoprene and 1,5-Hexadiene Polymerization. *Polyhedron* **2018**, *145*, 182–190.

(5) Salman, H.; Abraham, Y.; Tal, S.; Meltzman, S.; Kapon, M.; Tessler, N.; Speiser, S.; Eichen, Y. 1,3-Di(2-Pyrrolyl)Azulene: An Efficient Luminescent Probe for Fluoride. *Eur. J. Org. Chem.* **2005**, 2207–2212.

(6) Shao, C.; Shi, G.; Zhang, Y.; Pan, S.; Guan, X. Palladium-Catalyzed C–H Ethoxycarbonyldifluoromethylation of Electron-Rich Heteroarenes. *Org. Lett.* **2015**, *17*, 2652–2655.

(7) Mato, M.; Spinnato, D.; Leutzsch, M.; Moon, H. W.; Reijerse, E. J.; Cornella, J. Bismuth Radical Catalysis in the Activation and Coupling of Redox-Active Electrophiles. *Nat. Chem.* **2023**, *15*, 1138–1145.

(8) Ghosh, I.; Khamrai, J.; Savateev, A.; Shlapakov, N.; Antonietti, M.; König, B. Organic Semiconductor Photocatalyst Can Bifunctionalize Arenes and Heteroarenes. *Science* **2019**, *365*, 360–366.

(9) Li, L.; Mu, X.; Liu, W.; Wang, Y.; Mi, Z.; Li, C.-J. Simple and Clean Photoinduced Aromatic Trifluoromethylation Reaction. *J. Am. Chem. Soc.* **2016**, *138*, 5809–5812.

(10) Ye, F.; Berger, F.; Jia, H.; Ford, J.; Wortman, A.; Börgel, J.; Genicot, C.; Ritter, T. Aryl Sulfonium Salts for Site-Selective Late-Stage Trifluoromethylation. *Angew. Chem., Int. Ed.* **2019**, *58*, 14615–14619.

(11) Muralirajan, K.; Kancherla, R.; Bau, J. A.; Taksande, M. R.; Qureshi, M.; Takanabe, K.; Rueping, M. Exploring the Structure and Performance of Cd–Chalcogenide Photocatalysts in Selective Trifluoromethylation. *ACS Catal.* **2021**, *11*, 14772–14780.

(12) Wang, D.; Deng, G.-J.; Chen, S.; Gong, H. Catalyst-Free Direct C–H Trifluoromethylation of Arenes in Water–Acetonitrile. *Green Chem.* **2016**, *18*, 5967–5970.

(13) Pang, Y.; Lee, J. W.; Kubota, K.; Ito, H. Solid-State Radical C–H Trifluoromethylation Reactions Using Ball Milling and Piezoelectric Materials. *Angew. Chem. Int. Ed.* **2020**, *59*, 22570–22576.

(14) Li, X.; Zhao, J.; Zhang, L.; Hu, M.; Wang, L.; Hu, J. Copper-Mediated Trifluoromethylation Using Phenyl Trifluoromethyl Sulfoxide. *Org. Lett.* **2015**, *17*, 298–301.

(15) Transformation of Brucine into Trifluoromethyl Neobrucine Using the Homoleptic Nickel Catalyst $[Ni(CF_3)_4]^{2-}$. *Tetrahedron Lett.* **2022**, *97*, 153795.

(16) Wang, Y.; Wang, J.; Li, G.-X.; He, G.; Chen, G. Halogen-Bond-Promoted Photoactivation of Perfluoroalkyl Iodides: A Photochemical Protocol for Perfluoroalkylation Reactions. *Org. Lett.* **2017**, *19*, 1442–1445.

(17) Yang, X.; Reijerse, E. J.; Bhattacharyya, K.; Leutzsch, M.; Kochius, M.; Nöthling, N.; Busch, J.; Schnegg, A.; Auer, A. A.; Cornella, J. Radical Activation of N–H and O–H Bonds at Bismuth(II). *J. Am. Chem. Soc.* **2022**.

(18) Magre, M.; Cornella, J. Redox-Neutral Organometallic Elementary Steps at Bismuth: Catalytic Synthesis of Aryl Sulfonyl Fluorides. *J. Am. Chem. Soc.* **2021**, *143*, 21497–21502.

(19) Vránová, I.; Jambor, R.; Růžička, A.; Jirásko, R.; Dostál, L. Reactivity of N,C,N-Chelated Antimony(III) and Bismuth(III) Chlorides with Lithium Reagents: Addition vs Substitution. *Organometallics* **2015**, *34*, 534–541.

(20) Hatchard, C. G.; Parker, C. A.; Bowen, E. J. A New Sensitive Chemical Actinometer - II. Potassium Ferrioxalate as a Standard Chemical Actinometer. *Proc. R. Soc. Lond. A Math. Phys. Sci.* **1997**, *235*, 518–536.

(21) Montalti, M.; Credi, A.; Prodi, L.; Teresa Gandolfi, M. *Handbook of Photochemistry*; CRC Press, 2006.

(22) Hoshi, T.; Nakazawa, T.; Saitoh, I.; Mori, A.; Suzuki, T.; Sakai, J.-I.; Hagiwara, H. Biphenylene-Substituted Ruthenocenylphosphine for Suzuki-Miyaura Coupling of Aryl Chlorides. *Org. Lett.* **2008**, *10*, 2063–2066.

(23) Tania; Poynder, T. B.; Kaur, A.; Barwise, L.; Houston, S. D.; Nair, A. J.; Clegg, J. K.; Wilson, D. J. D.; Dutton, J. L. PhICl2 Is Activated by Chloride Ions. *Dalton Trans.* **2021**.

(24) Lehnherr, D.; Ji, Y.; Neel, A. J.; Cohen, R. D.; Brunskill, A. P. J.; Yang, J.; Reibarkh, M. Discovery of a Photoinduced Dark Catalytic Cycle Using in Situ LED-NMR Spectroscopy. *J. Am. Chem. Soc.* **2018**, *140*, 13843–13853.

(25) Hardman, N. J.; Twamley, B.; Power, P. P. (2,6-$Mes_2H_3C_6)_2BiH$, a Stable, Molecular Hydride of a Main Group Element of the Sixth Period, and Its Conversion to the Dibismuthene (2,6-$Mes_2H_3C_6$)BiBi(2,6-$Mes_2C_6H_3$). *Angew. Chem. Int. Ed.* **2000**, *39*, 2771–2773.

(26) Brudzisz, A. M.; Brzózka, A.; Sulka, G. D. Effect of the Supporting Electrolyte on Chloroform Reduction at a Silver Electrode in Aqueous Solutions. *Molecules* **2021**, *26*.

(27) Nagib, D. A.; MacMillan, D. W. C. Trifluoromethylation of Arenes and Heteroarenes by Means of Photoredox Catalysis. *Nature* **2011**, *480*, 224–228.

(28) Neese, F. The ORCA Program System. *Wiley Interdiscip. Rev. Comput. Mol. Sci.* **2012**, *2*, 73–78.

(29) Neese, F. Software Update: The ORCA Program System—Version 5.0. *Wiley Interdiscip. Rev. Comput. Mol. Sci.* **2022**, *12*.

(30) Becke, A. D. Density-Functional Exchange-Energy Approximation with Correct Asymptotic Behavior. *Phys. Rev. A Gen. Phys.* **1988**, *38*, 3098–3100.

(31) Weigend, F.; Ahlrichs, R. Balanced Basis Sets of Split Valence, Triple Zeta Valence and Quadruple Zeta Valence Quality for H to Rn: Design and Assessment of Accuracy. *Phys. Chem. Chem. Phys.* **2005**, *7*, 3297–3305.

(32) Pantazis, D. A.; Neese, F. All-Electron Scalar Relativistic Basis Sets for the 6p Elements. *Theor. Chem. Acc.* **2012**, *131*, 1292.

(33) Pantazis, D. A.; Chen, X.-Y.; Landis, C. R.; Neese, F. All-Electron Scalar Relativistic Basis Sets for Third-Row Transition Metal Atoms. *J. Chem. Theory Comput.* **2008**, *4*, 908–919.

(34) Pantazis, D. A.; Neese, F. All-Electron Scalar Relativistic Basis Sets for the Lanthanides. *J. Chem. Theory Comput.* **2009**, *5*, 2229–2238.

(35) Pantazis, D. A.; Neese, F. All-Electron Scalar Relativistic Basis Sets for the Actinides. *J. Chem. Theory Comput.* **2011**, *7*, 677–684.

(36) Grimme, S.; Ehrlich, S.; Goerigk, L. Effect of the Damping Function in Dispersion Corrected Density Functional Theory. *J. Comput. Chem.* **2011**, *32,* 1456–1465.

(37) Grimme, S.; Antony, J.; Ehrlich, S.; Krieg, H. A Consistent and Accurate Ab Initio Parametrization of Density Functional Dispersion Correction (DFT-D) for the 94 Elements H-Pu. *J. Chem. Phys.* **2010**, *132*, 154104.

(38) Fukui, K. The Path of Chemical Reactions - the IRC Approach. *Acc. Chem. Res.* **1981**, *14,* 363–368.

(39) Pavlishchuk, V. V.; Addison, A. W. Conversion Constants for Redox Potentials Measured versus Different Reference Electrodes in Acetonitrile Solutions at 25 °C. *Inorganica Chim. Acta* **2000**, *298*, 97–102.

(40) Marenich, A. V.; Cramer, C. J.; Truhlar, D. G. Universal Solvation Model Based on Solute Electron Density and on a Continuum Model of the Solvent Defined by the Bulk Dielectric Constant and Atomic Surface Tensions. *J. Phys. Chem. B* **2009**, *113*, 6378–6396.

(41) (a) Yang, X.; Reijerse, E. J.; Bhattacharyya, K.; Leutzsch, M.; Kochi-us, M.; Nöthling, N.; Busch, J.; Schnegg, A.; Auer, A. A.; Cornella, J. Radical Activation of N–H and O–H Bonds at Bismuth(II). *J. Am. Chem. Soc.* **2022**, *144*, 16535–16544. (b) Yang, X.; Reijerse, E. J.; Nöthling, N.; SantaLucia, D. J.; Leutzsch, M.; Schnegg, A.; Cornella, J. Synthesis, Isolation, and Characterization of Two Cationic Organo-bismuth(II) Pincer Complexes Relevant in Radical Redox Chemistry. *J. Am. Chem. Soc.* **2023**, *145*, 5618–5623.

10. NMR Spectrum

Oxidative addition complex (5a)

^{1}H NMR (300 MHz, THF-d_8)

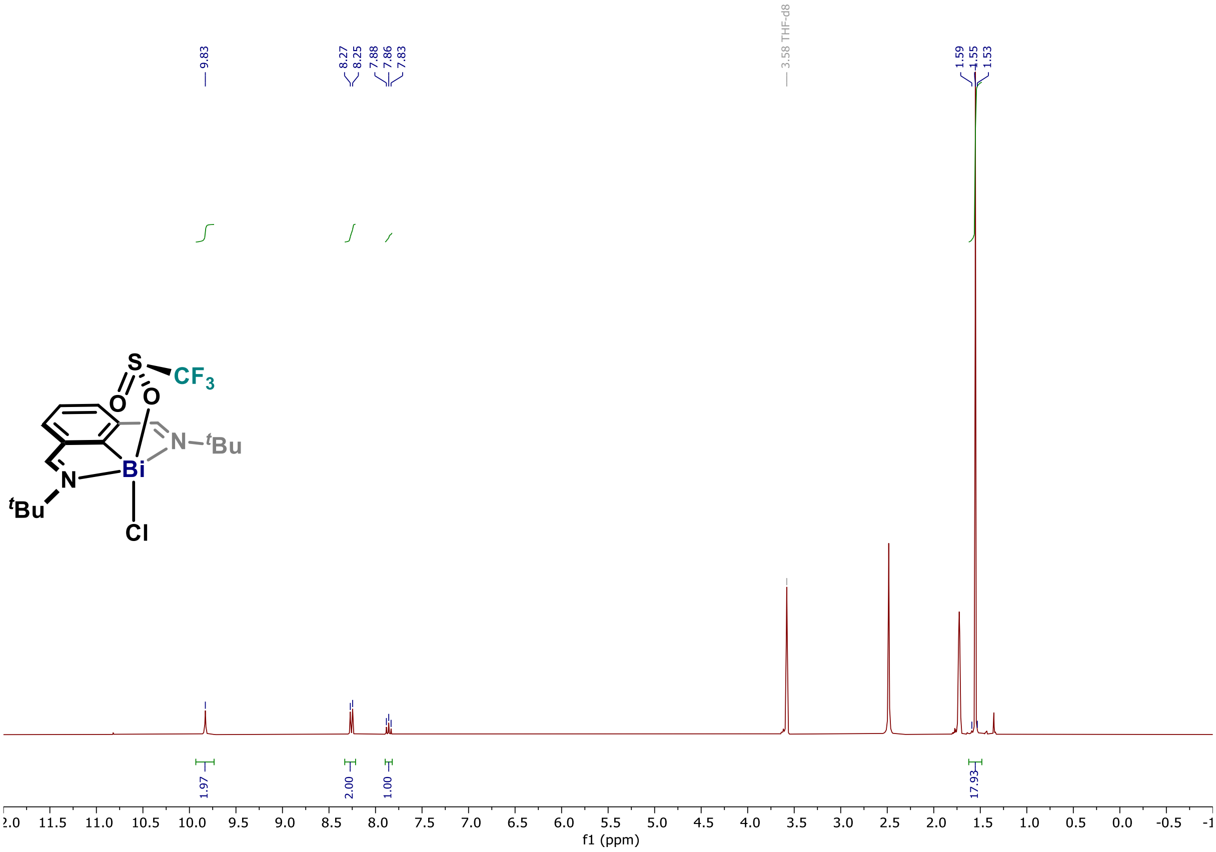

^{13}C{^{1}H} NMR (151 MHz, THF-d_8)

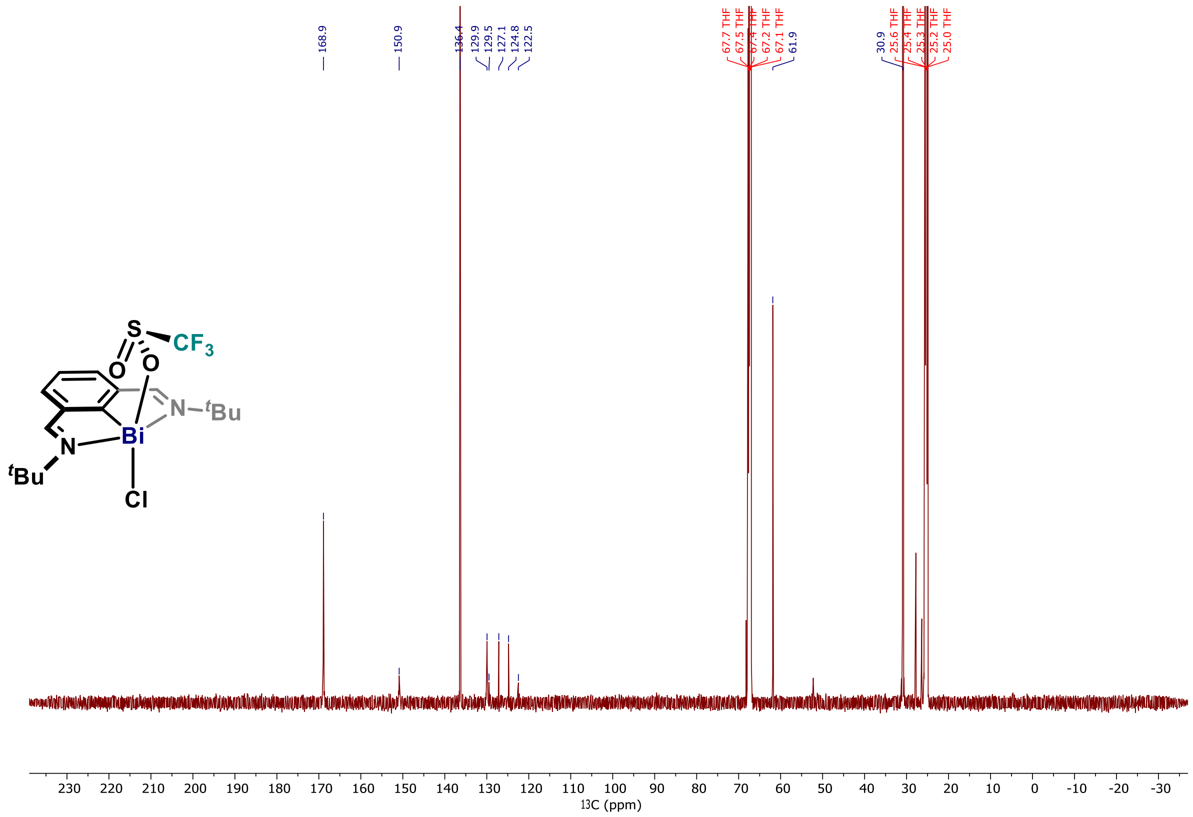

^{19}F NMR (282 MHz, THF-d_8)

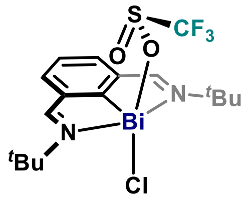

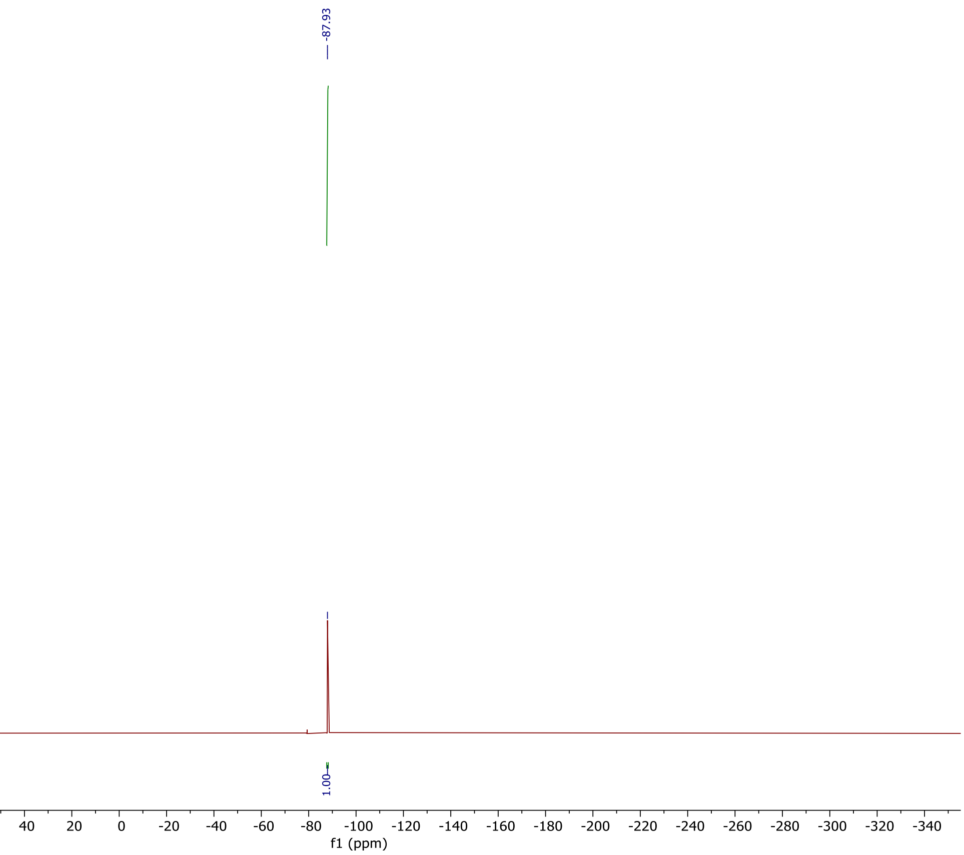

^{1}H-^{13}C HSQC

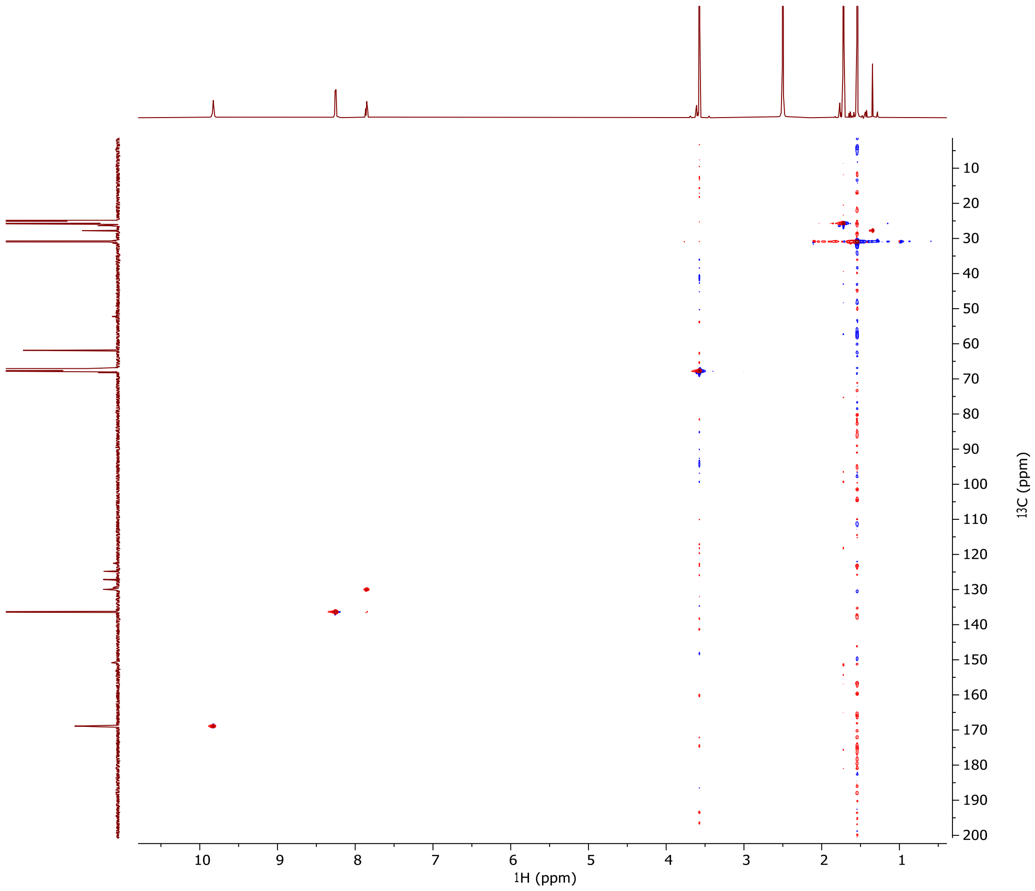

^{1}H-^{13}C HMBC

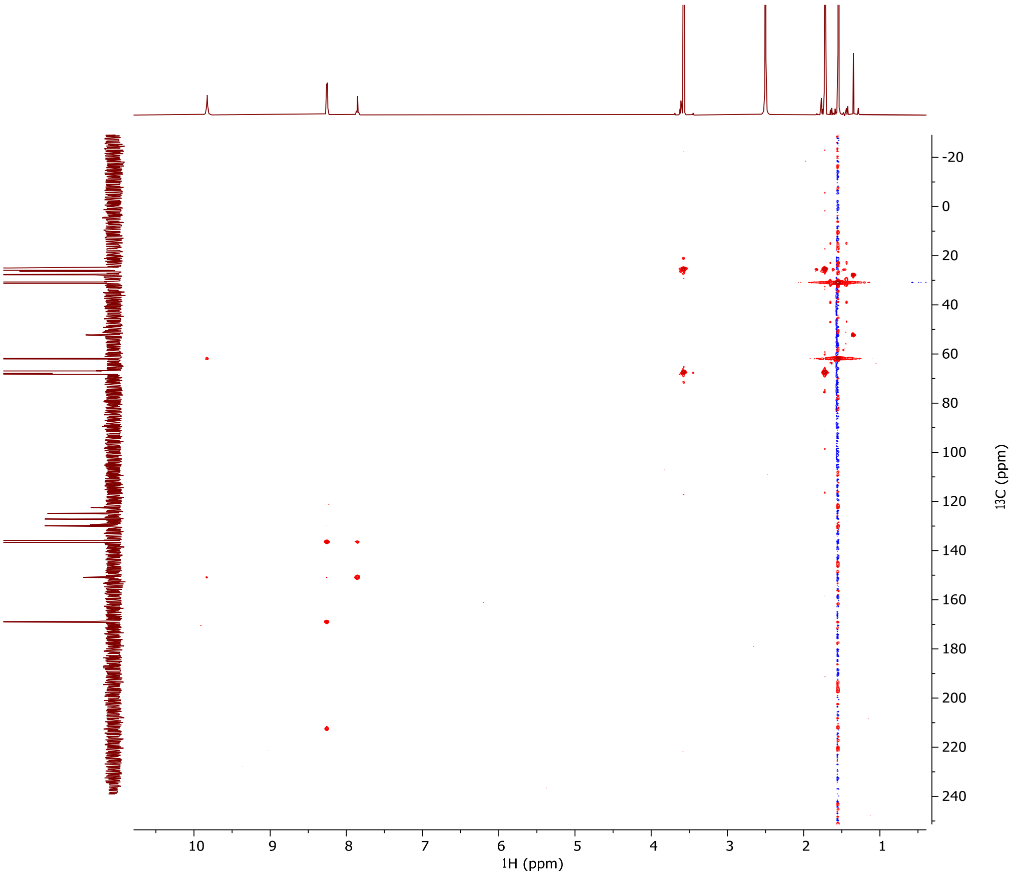

^{1}H-^{1}H COSY

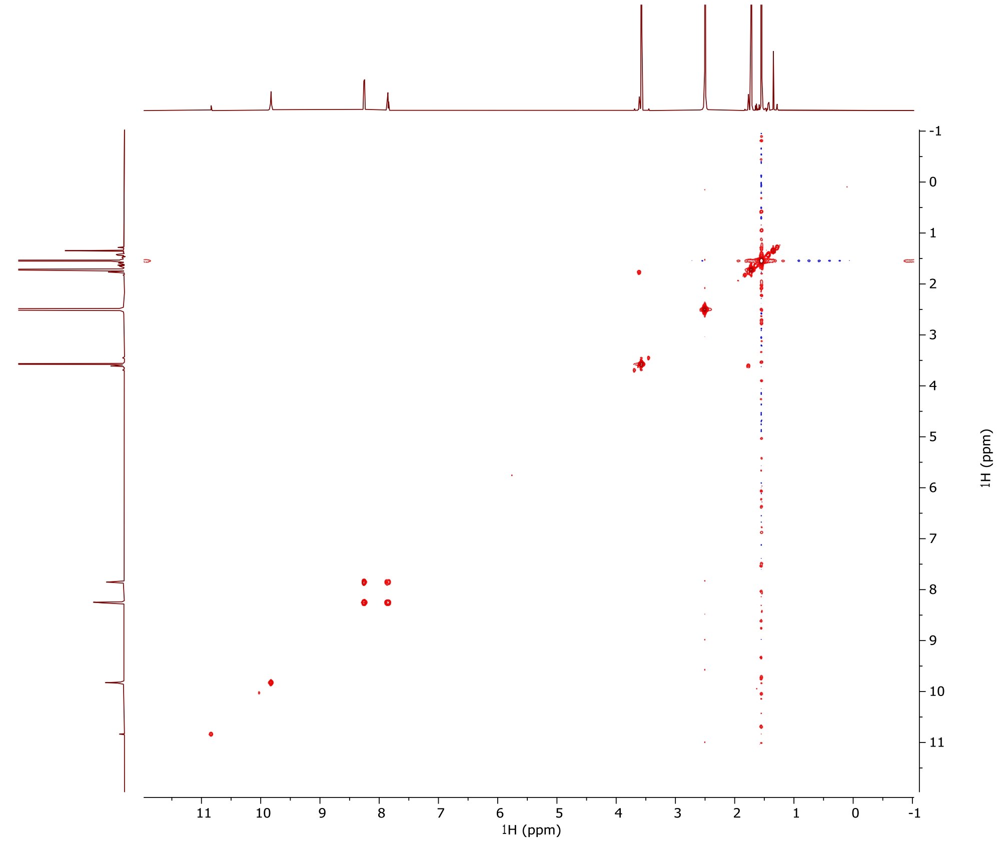

^{1}H-^{1}H NOESY

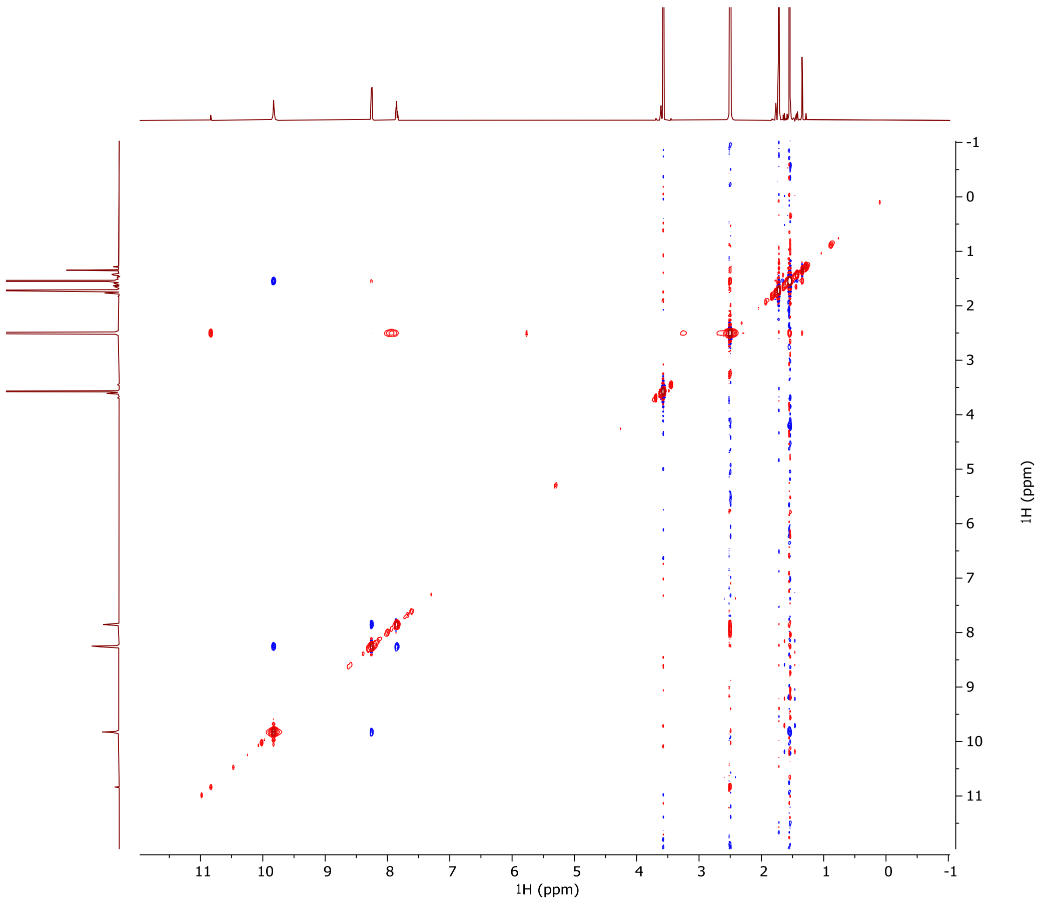

S1

^{1}H NMR (300 MHz, $CDCl_3$)

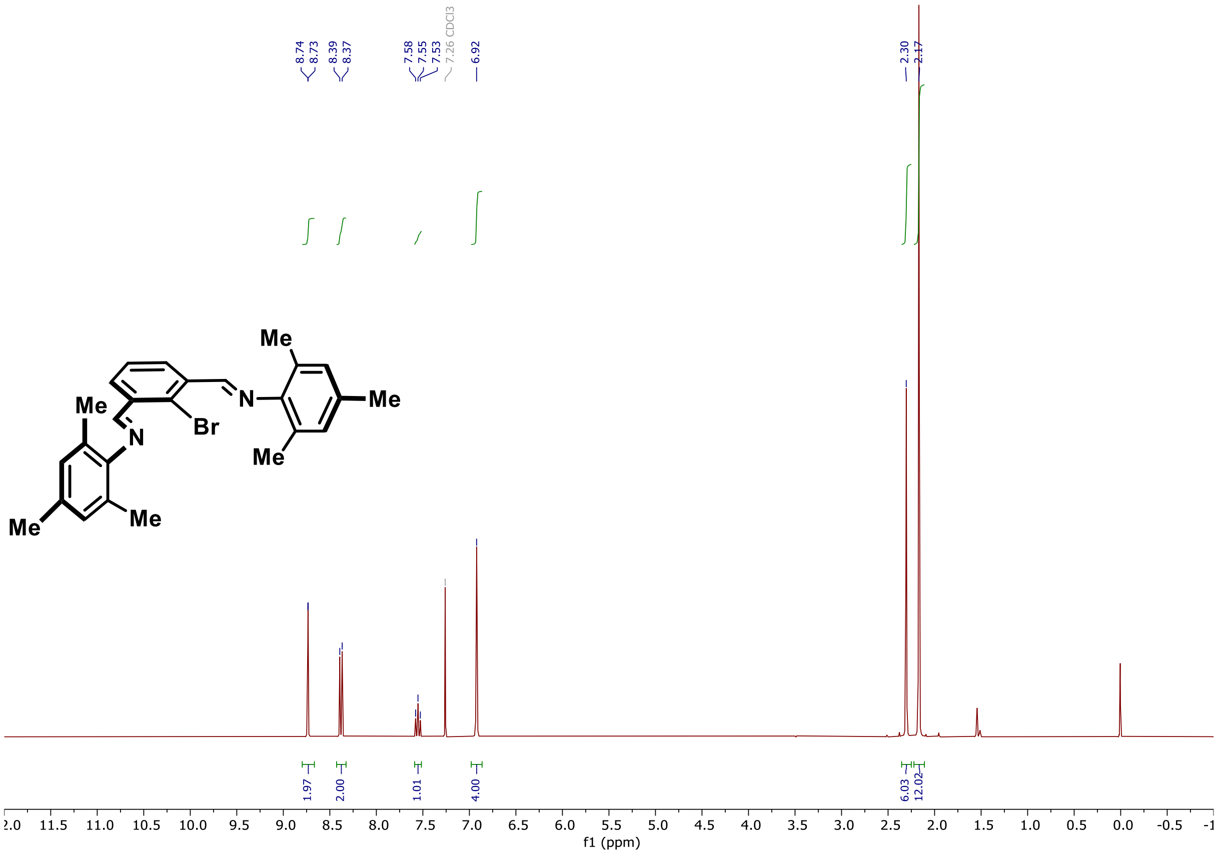

^{13}C{^{1}H} NMR (151 MHz, $CDCl_3$)

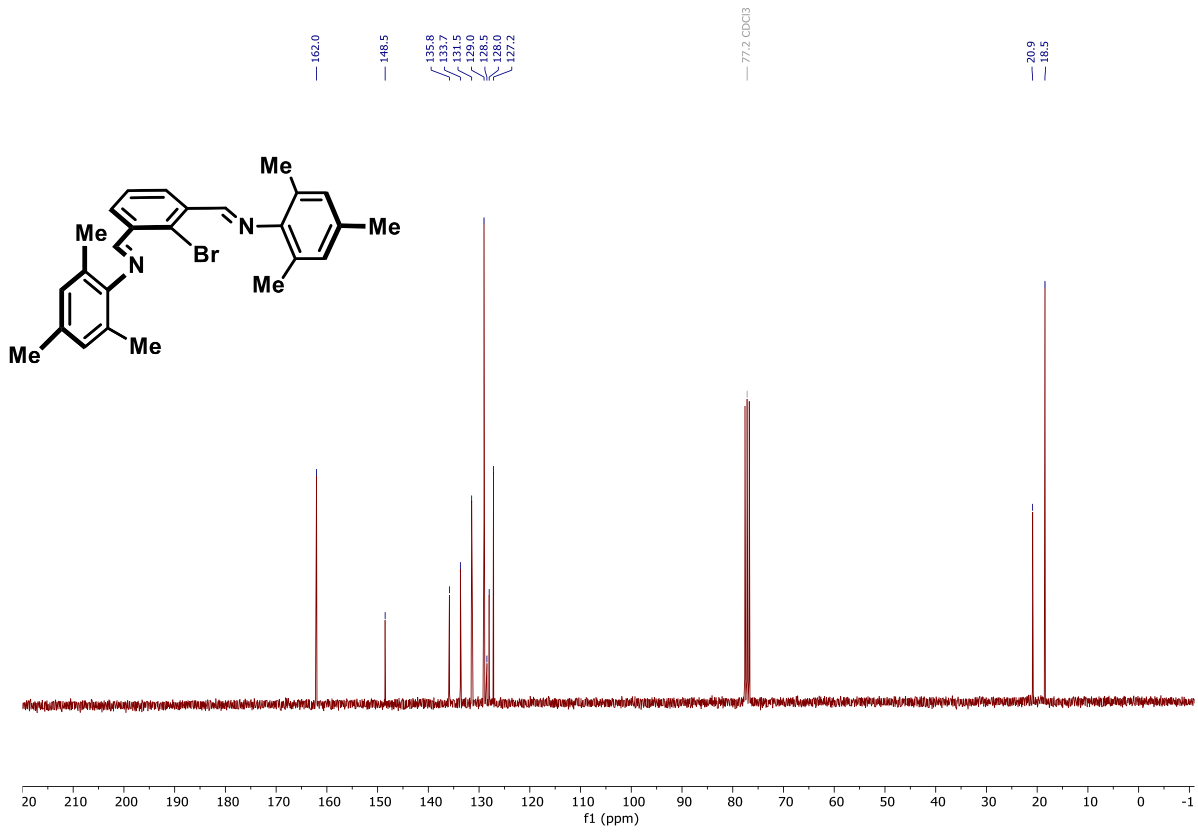

S2

^{1}H NMR (300 MHz, $CDCl_3$)

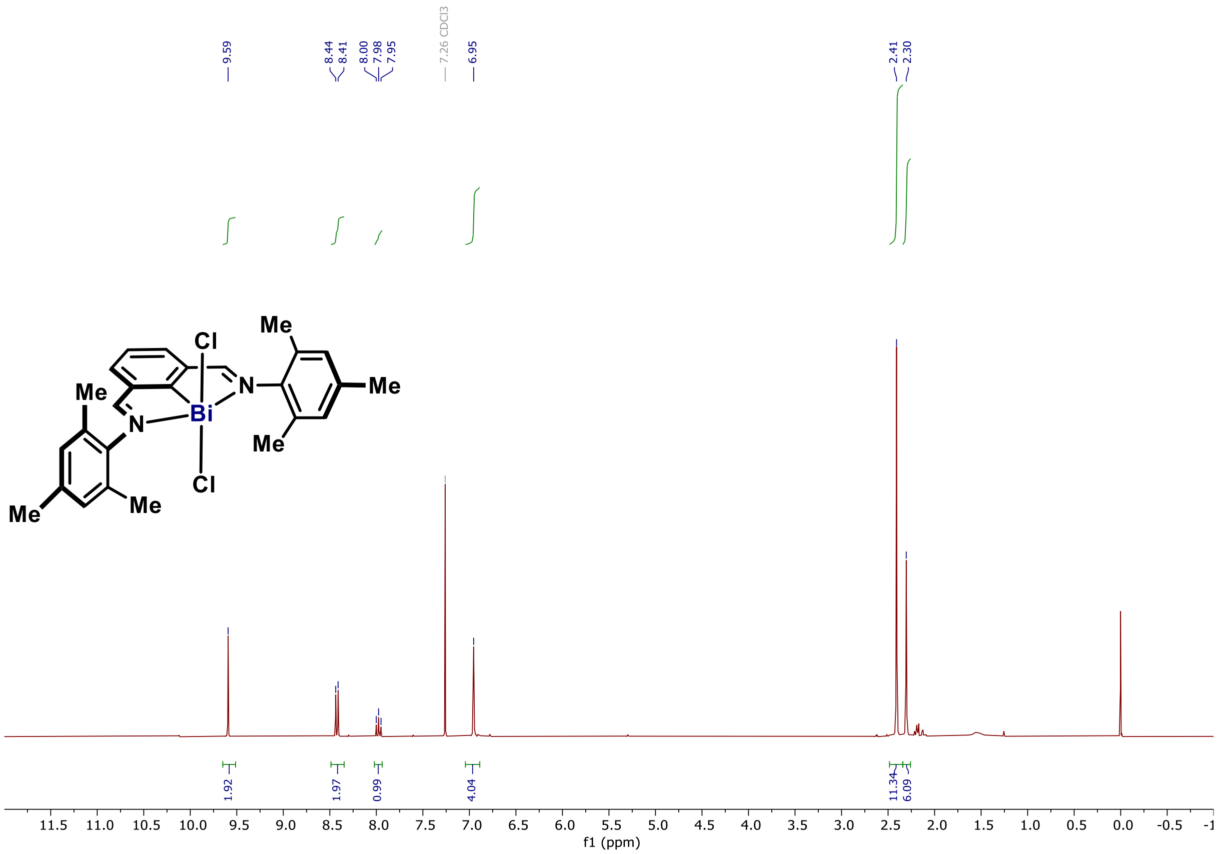

^{13}C{^{1}H} NMR (151 MHz, $CDCl_3$)

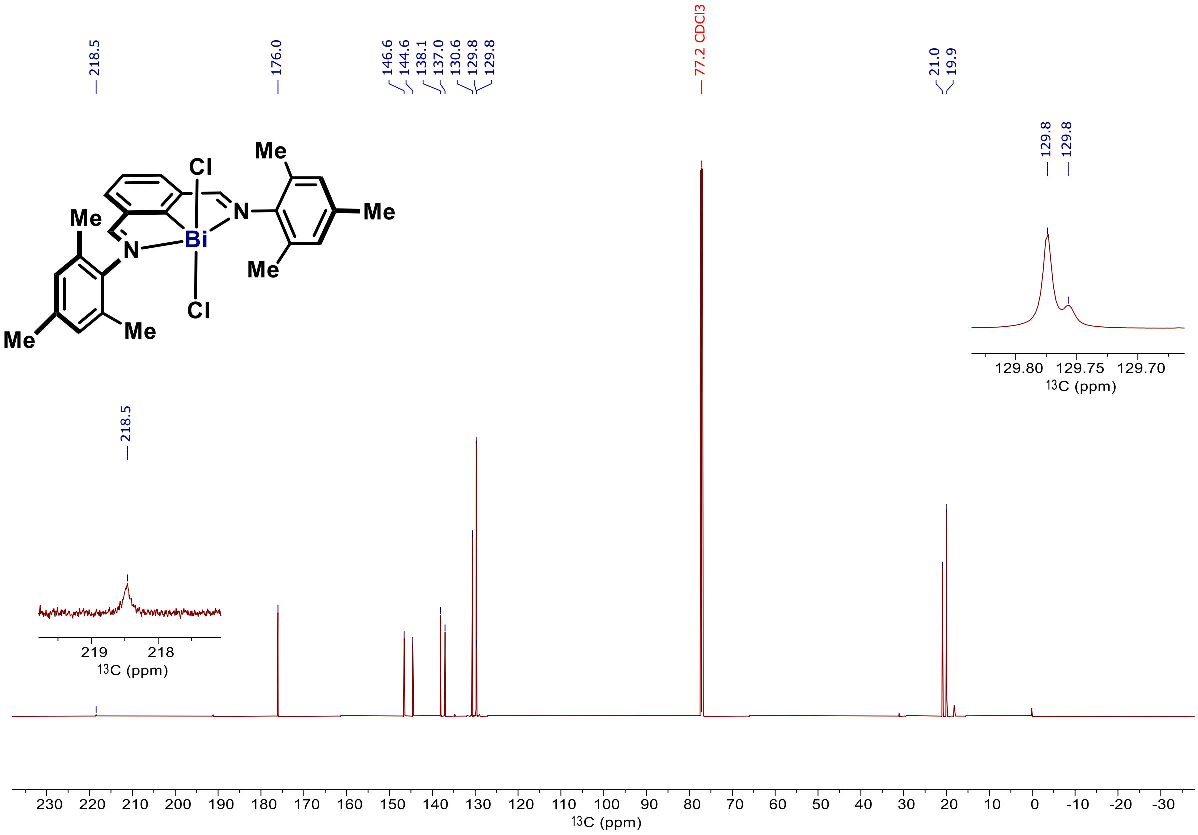

1c

^{1}H NMR (300 MHz, $CDCl_3$)

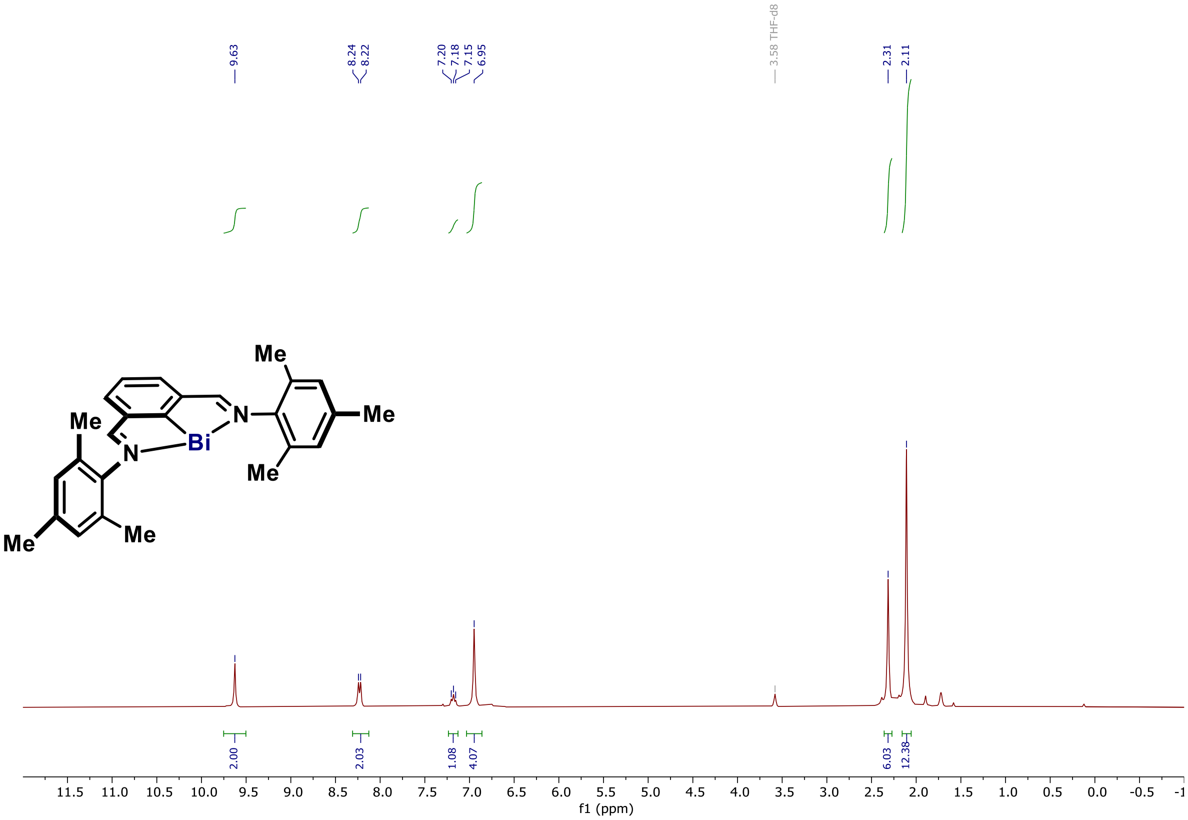

$^{13}C\{^{1}H\}$ NMR (75 MHz, $CDCl_3$)

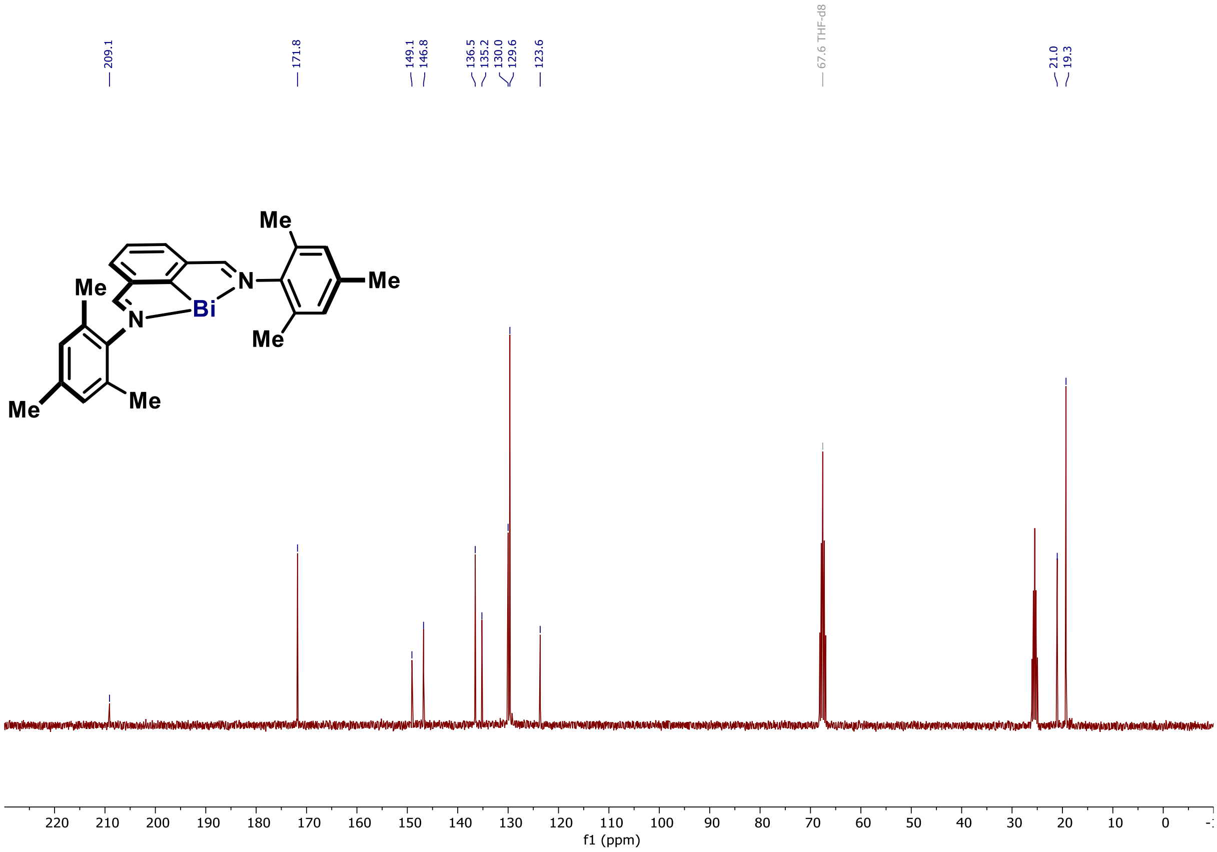

***tert*-butyl 1H-pyrrole-1-carboxylate (2l)**

^{1}H NMR (300 MHz, $CDCl_3$)

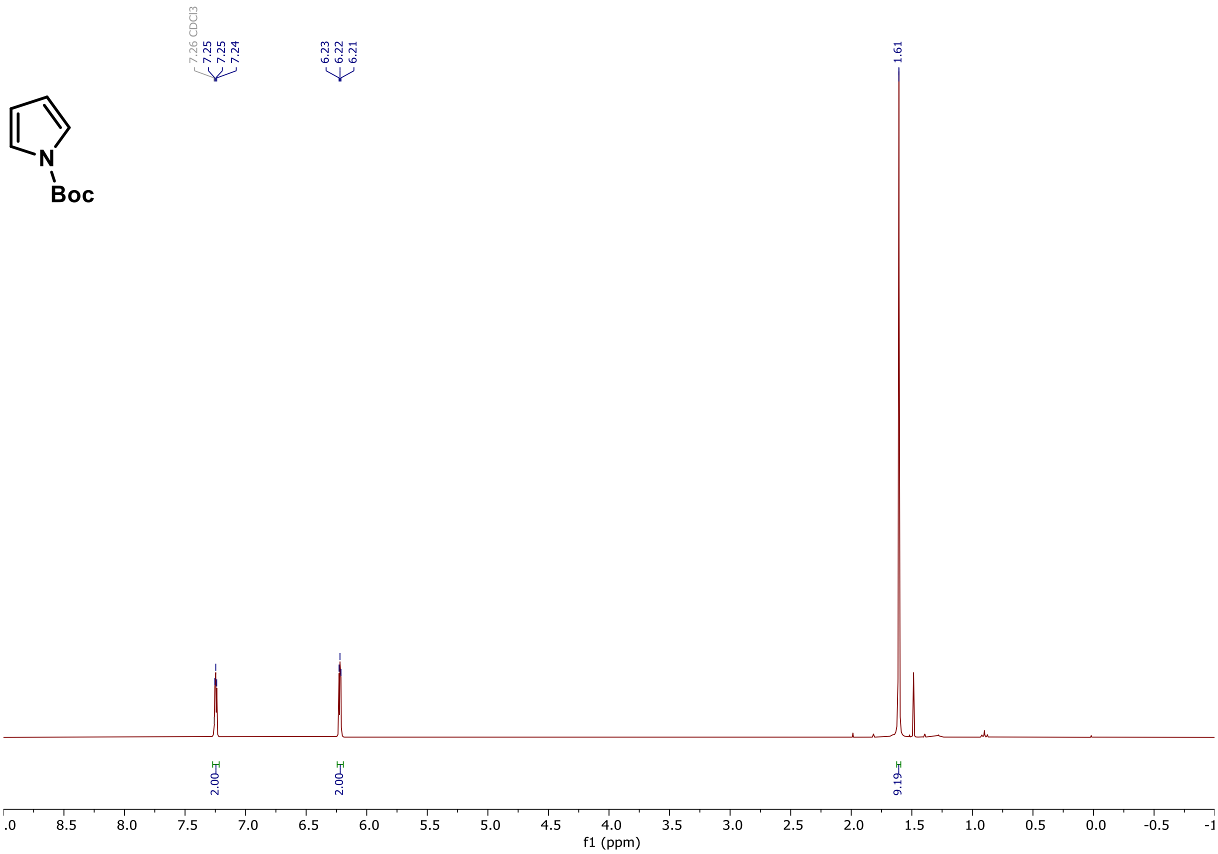

$^{13}C\{^1H\}$ NMR (75 MHz, $CDCl_3$)

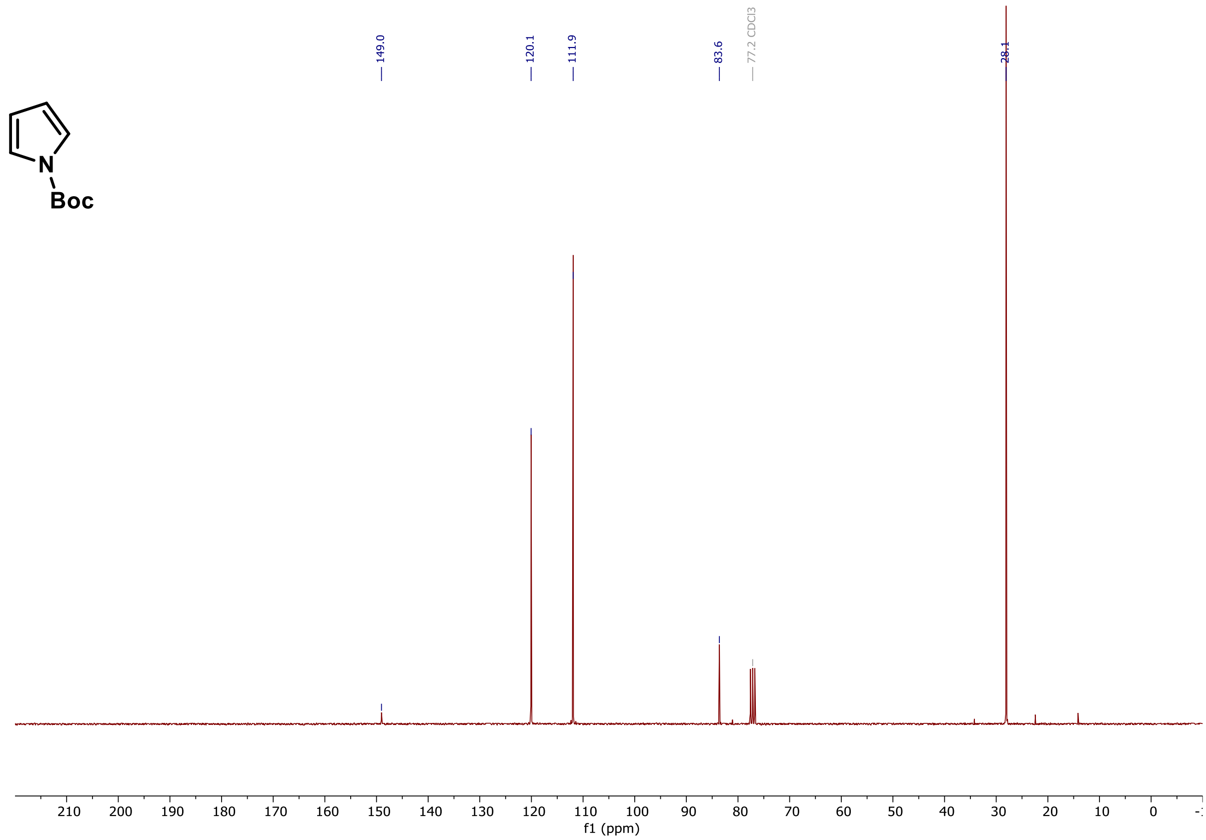

1-benzyl-3-methyl-1H-indole (2n)

^{1}H NMR (300 MHz, $CDCl_3$)

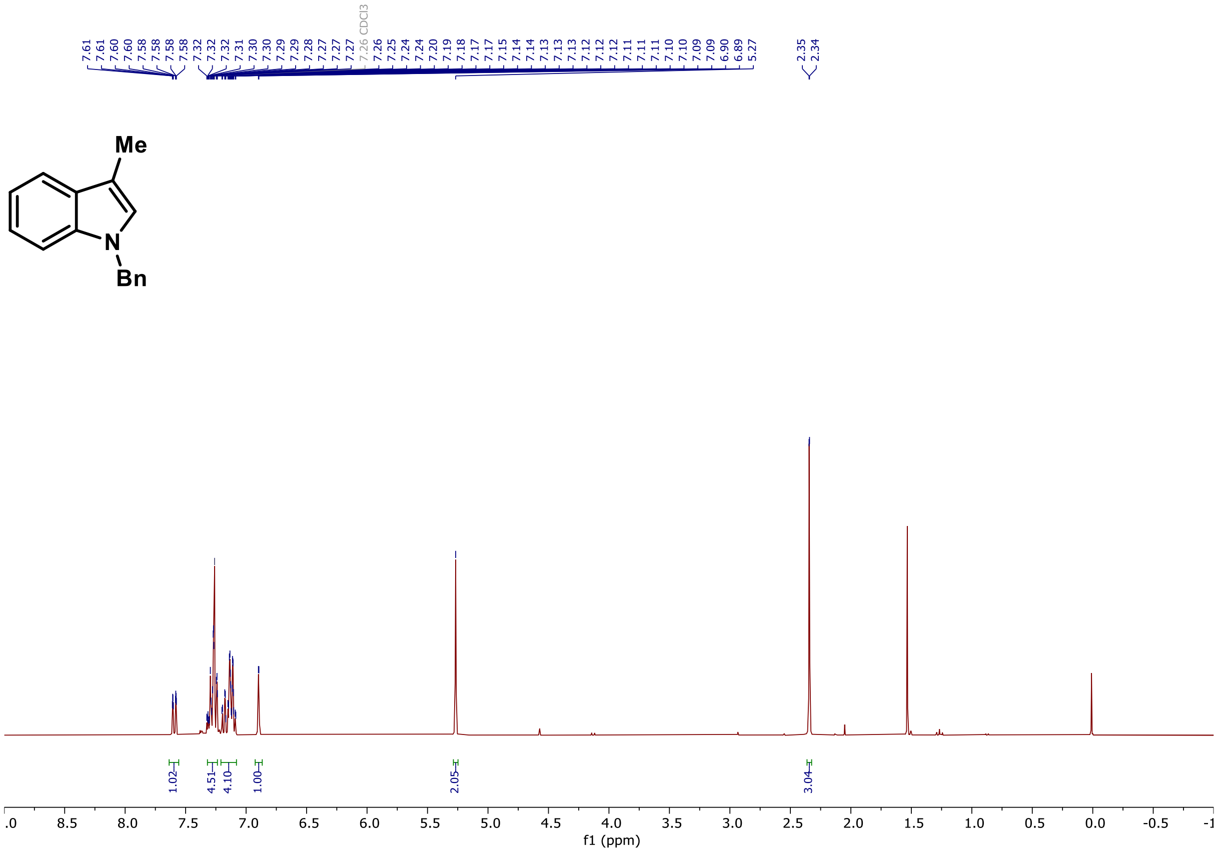

$^{13}C\{^{1}H\}$ NMR (151 MHz, $CDCl_3$)

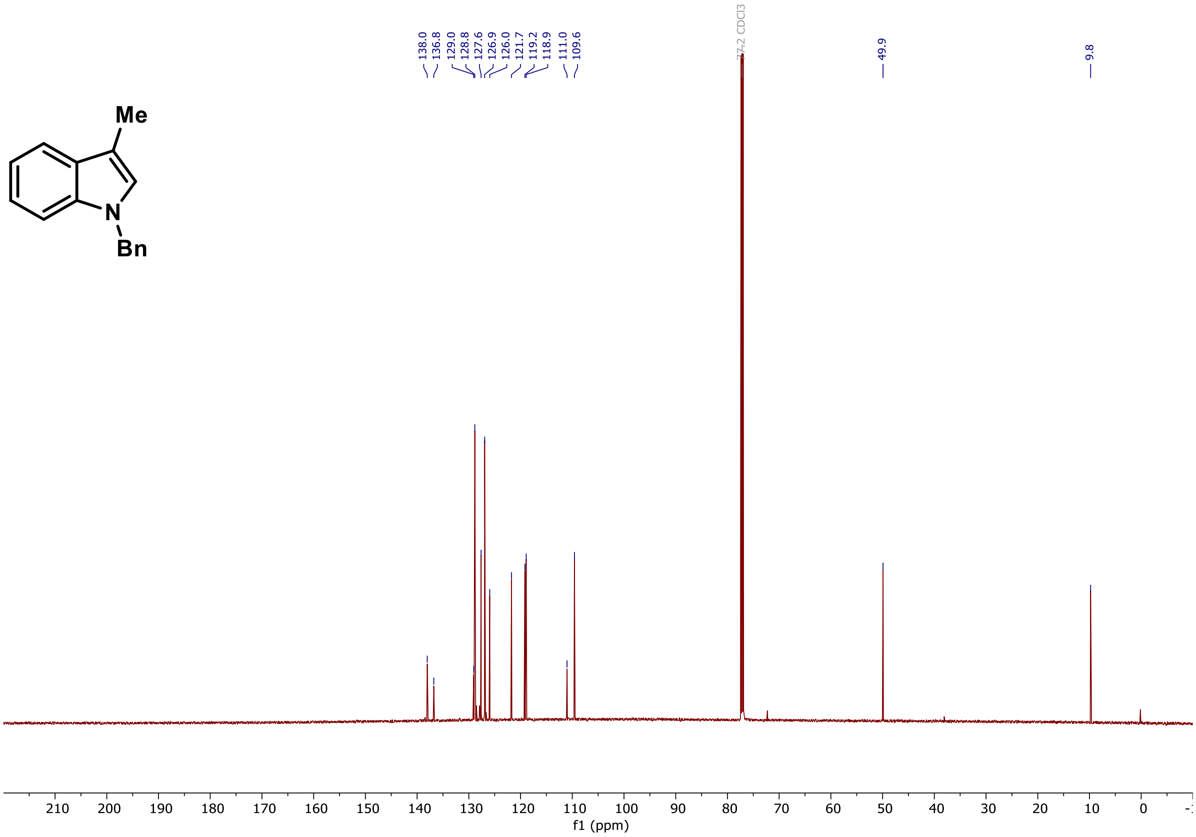

4,5,6,7-tetrachloro-1,3-dioxoisoindolin-2-yl 9,10-dihydroanthracene-9-carboxylate (7)

^{1}H NMR (600 MHz, $CDCl_3$)

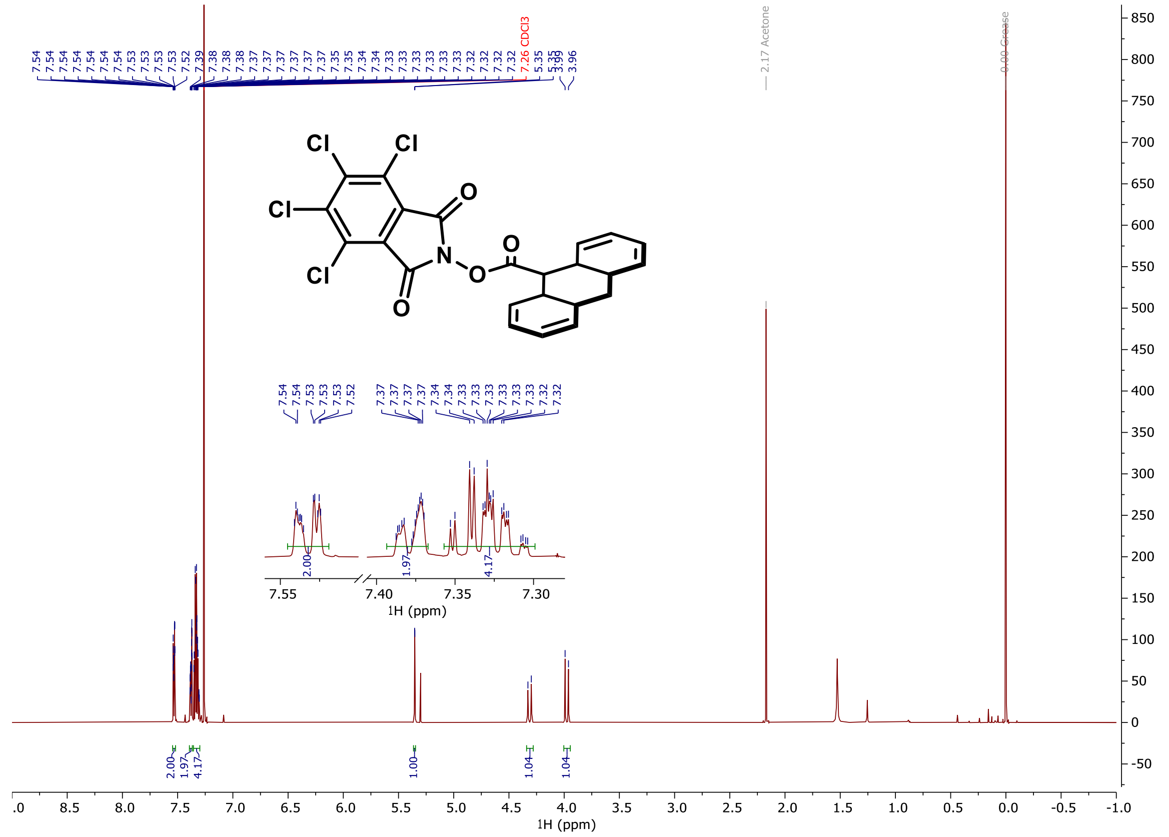

$^{13}C\{^1H\}$ NMR (151 MHz, $CDCl_3$)

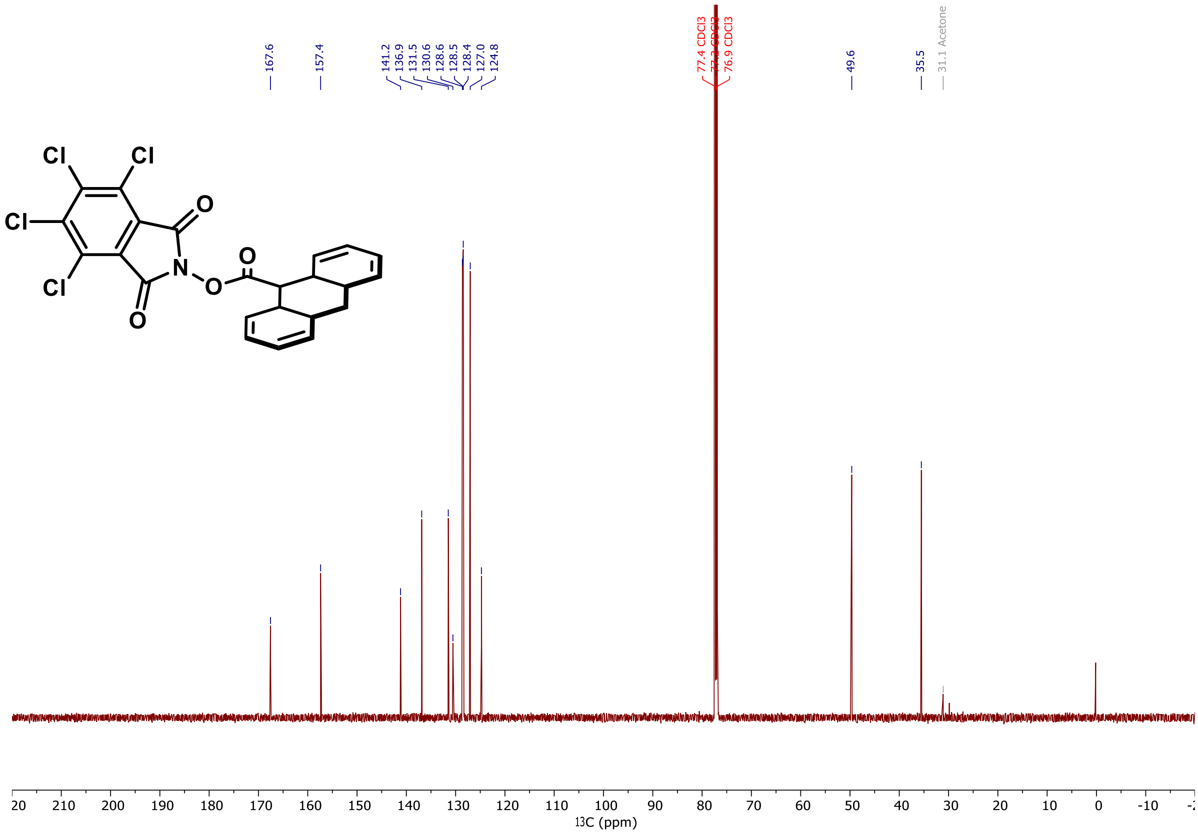

1,3,5-trimethoxy-2-(trifluoromethyl)benzene (4a)

^{1}H NMR (600 MHz, $CDCl_3$)

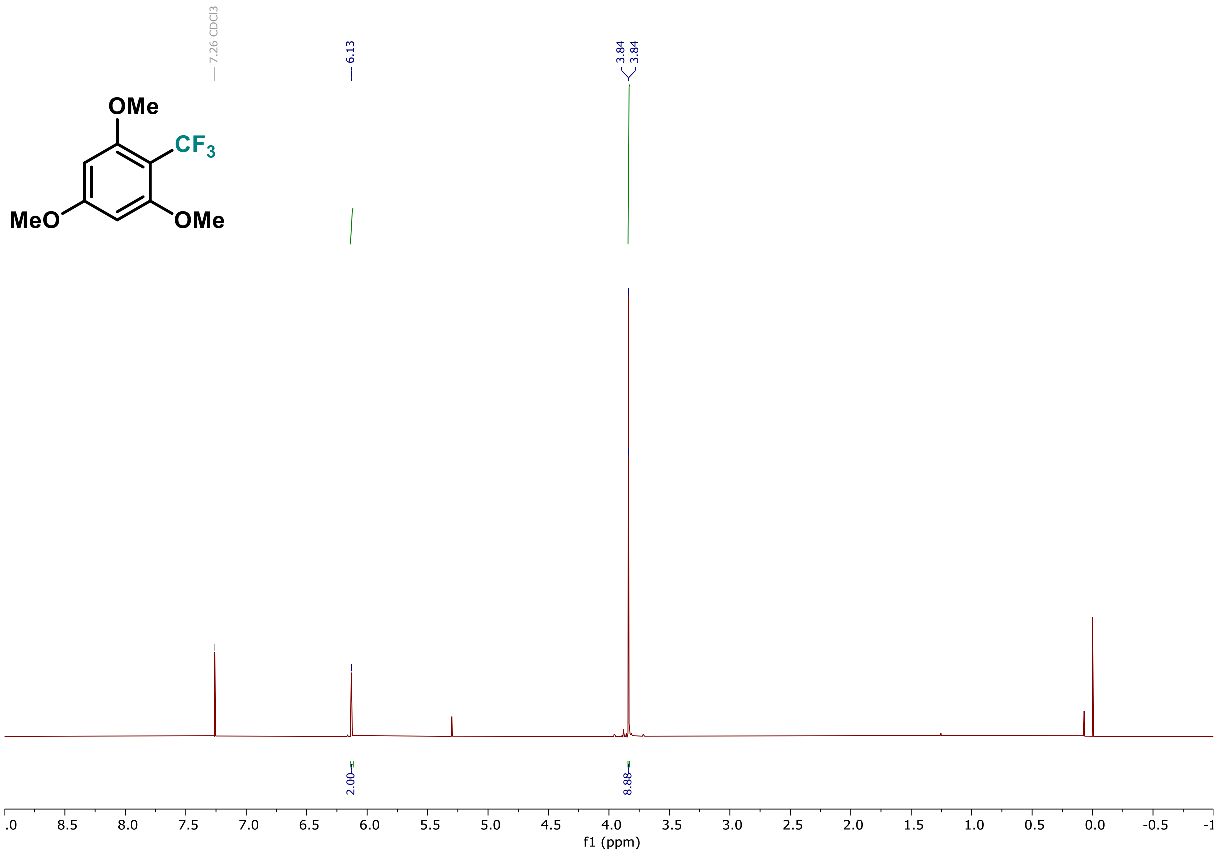

$^{13}C\{^{1}H\}$ NMR (151 MHz, $CDCl_3$)

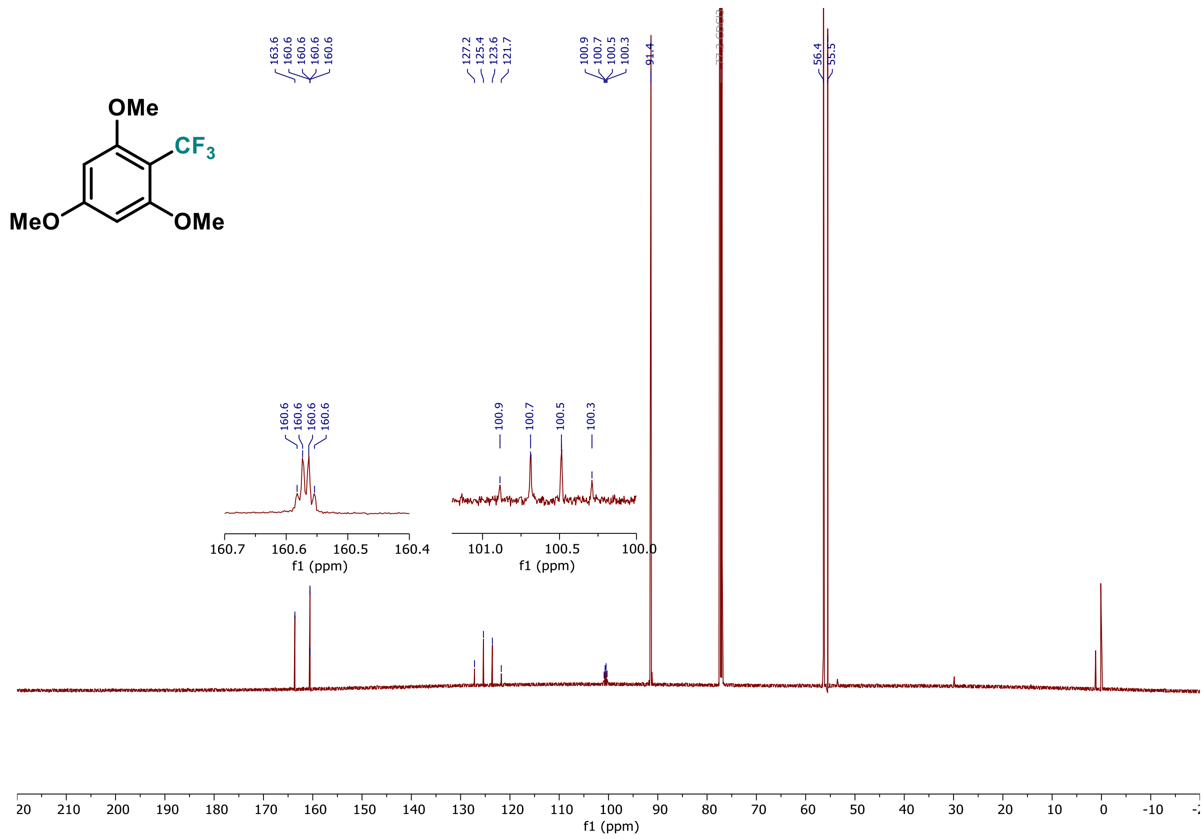

^{19}F NMR (282 MHz, $CDCl_3$)

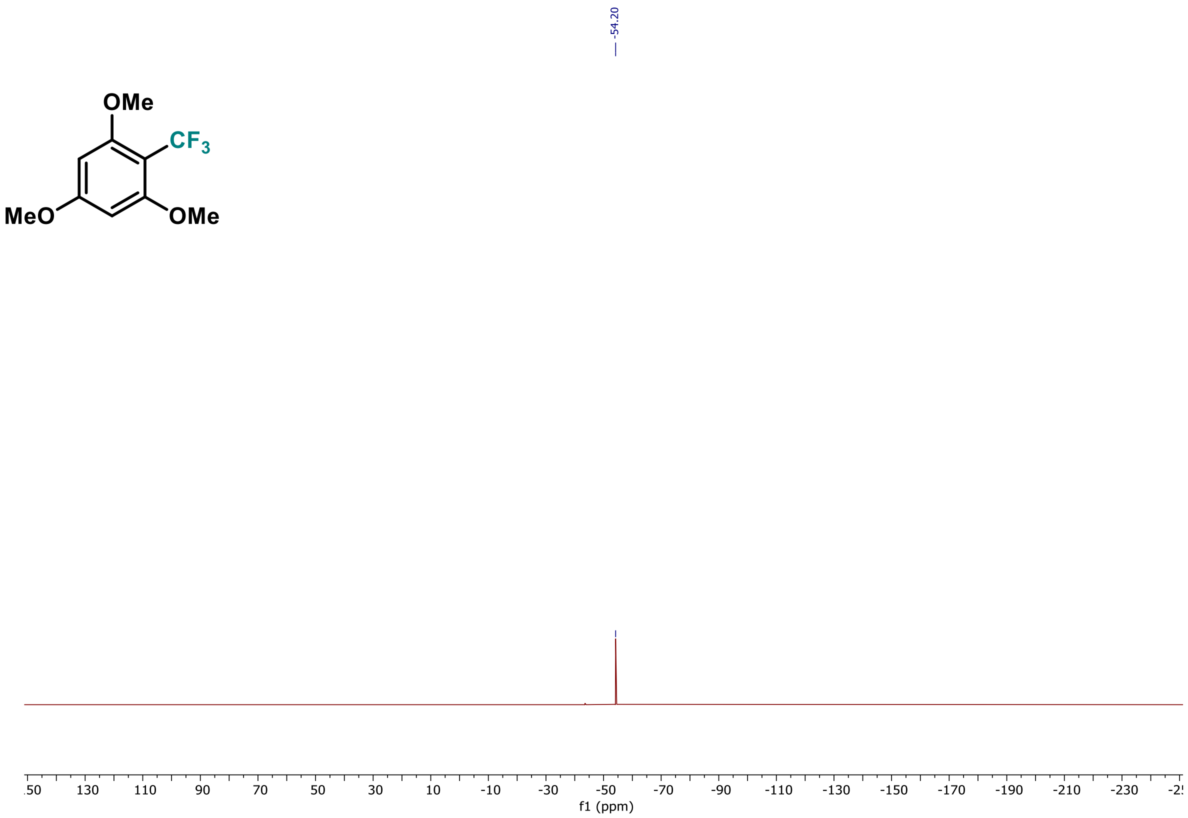

3,4,5-trimethoxy-2-(trifluoromethyl)benzaldehyde (4b)

^1H NMR (300 MHz, $CDCl_3$)

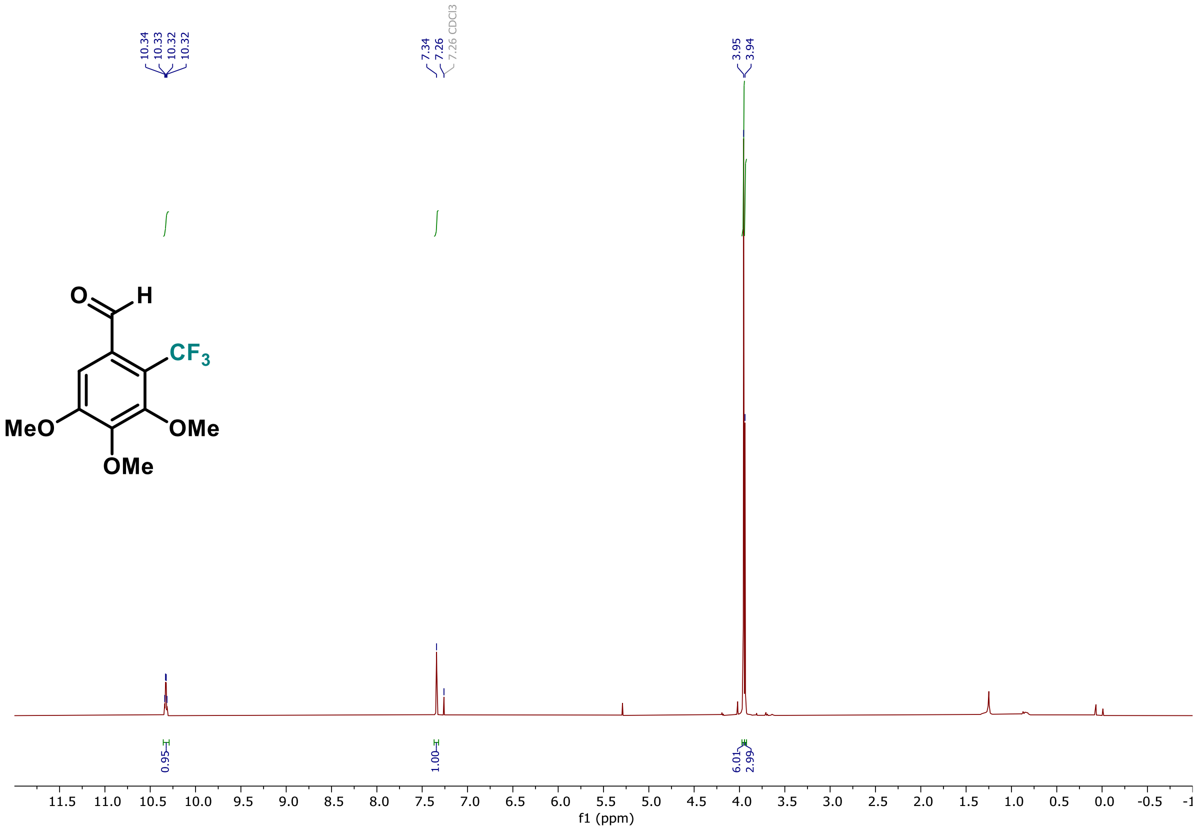

$^{13}C\{^{1}H\}$ NMR (151 MHz, $CDCl_3$)

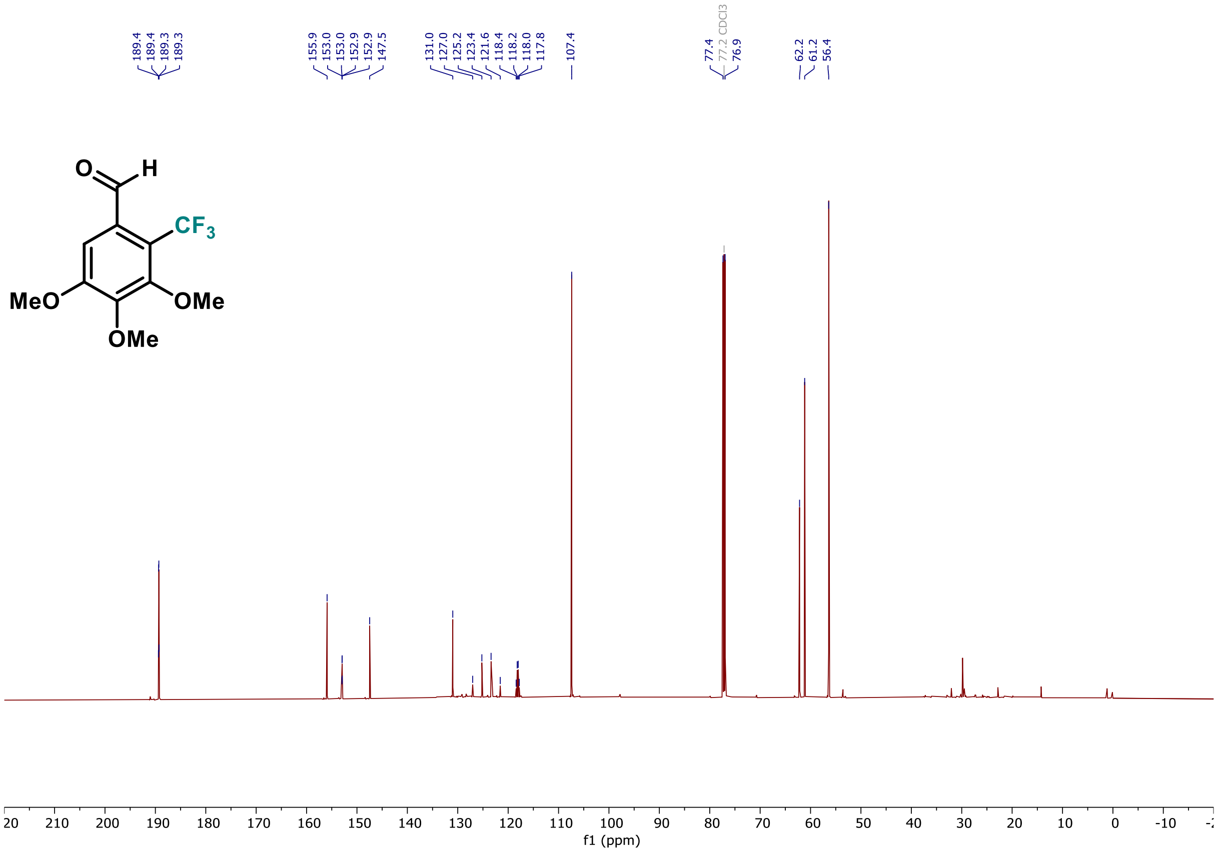

^{19}F NMR (282 MHz, $CDCl_3$)

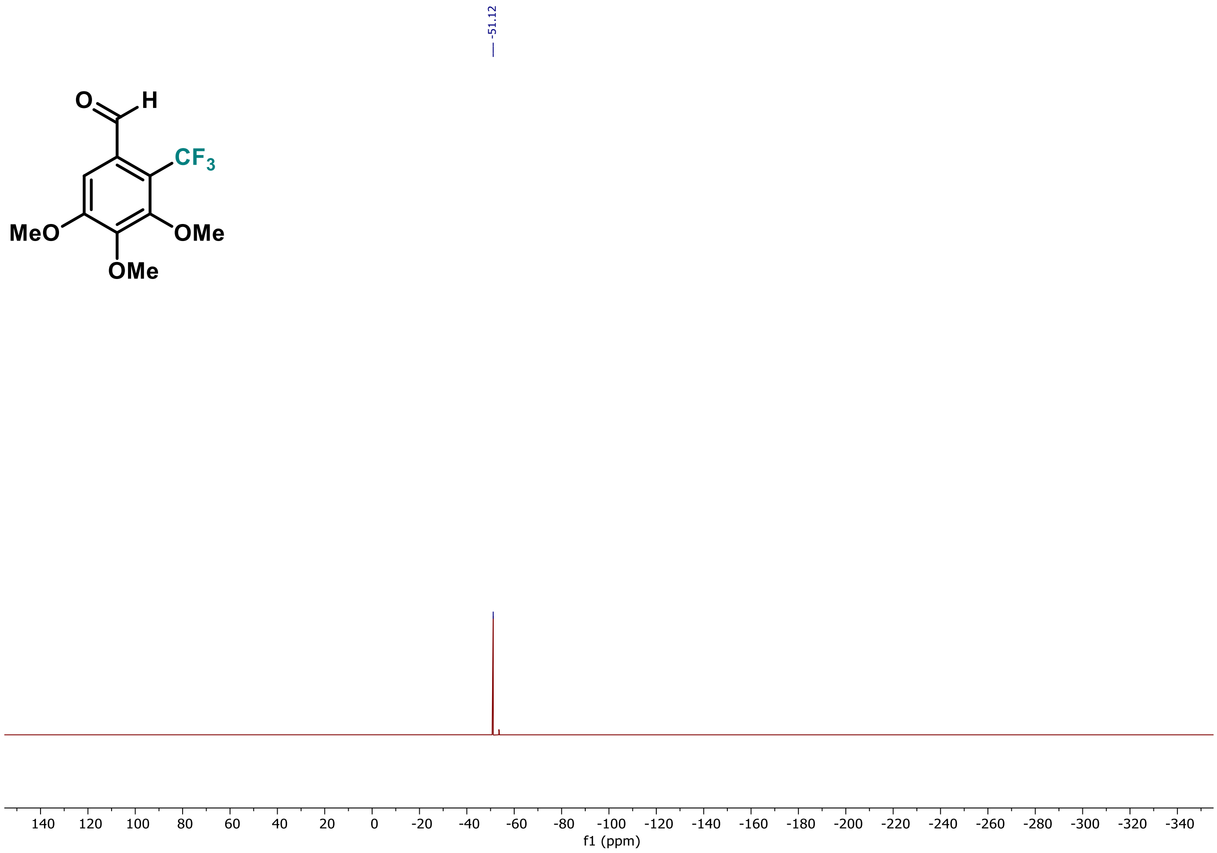

1-(3,4,5-trimethoxy-2-(trifluoromethyl)phenyl)ethan-1-one (4c)

^{1}H NMR (300 MHz, $CDCl_3$)

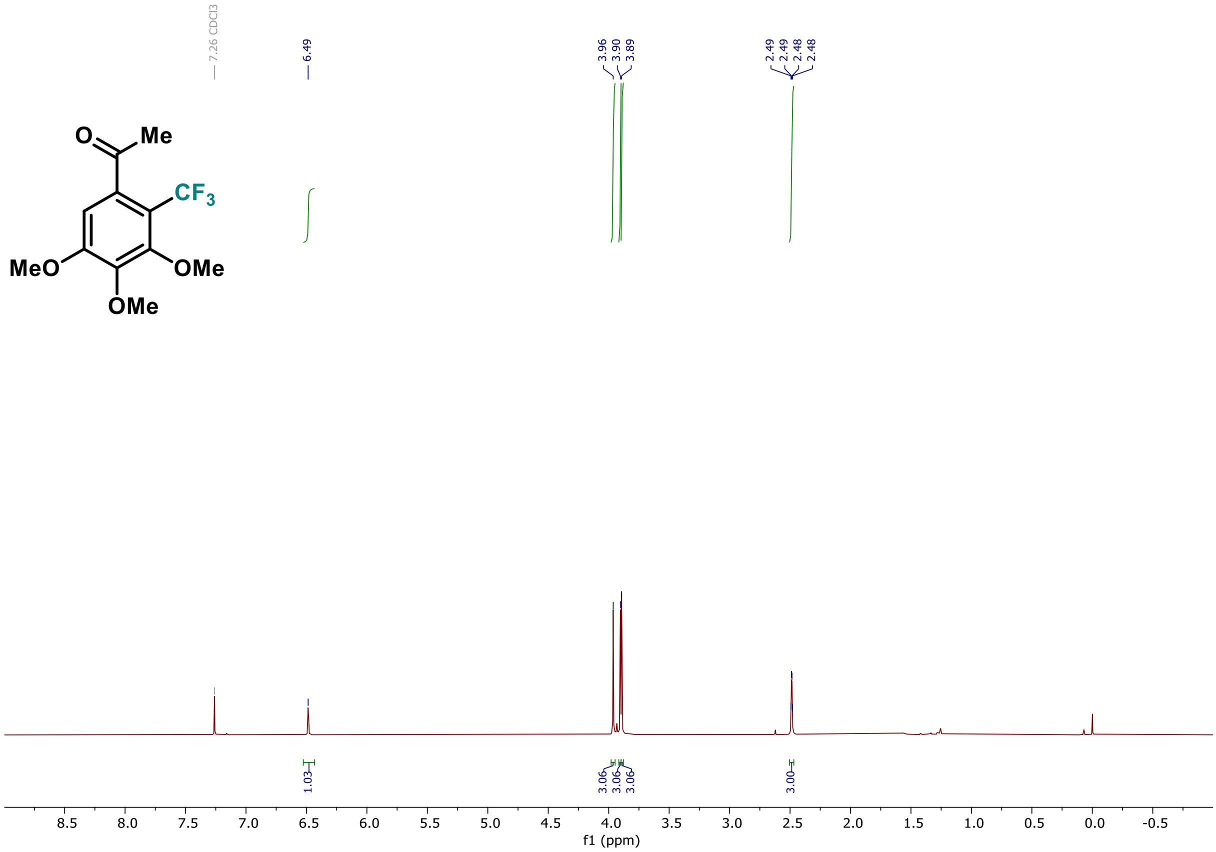

^{13}C{^{1}H} NMR (151 MHz, $CDCl_3$)

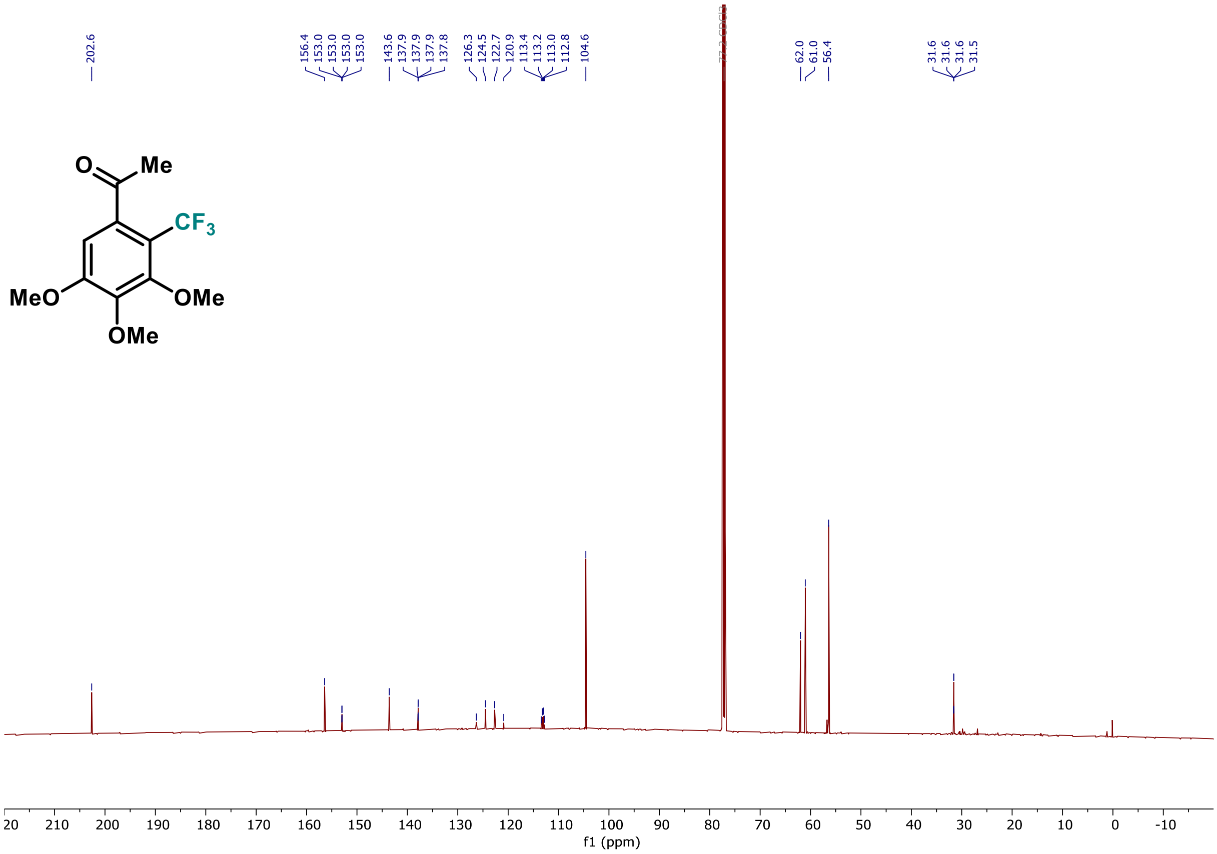

^{19}F NMR (282 MHz, $CDCl_3$)

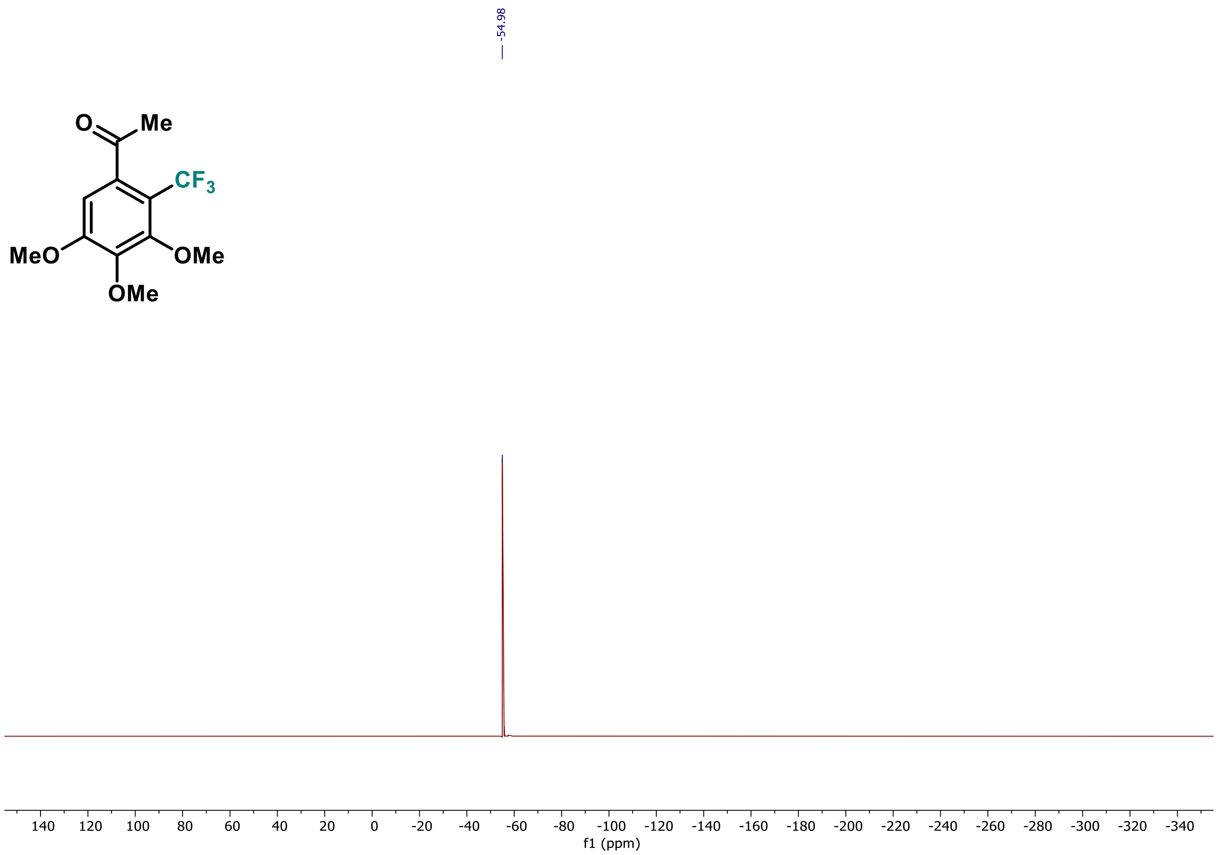

1,2,3-trimethoxy-5-methyl-4-(trifluoromethyl)benzene (4d)

^{1}H NMR (300 MHz, $CDCl_3$)

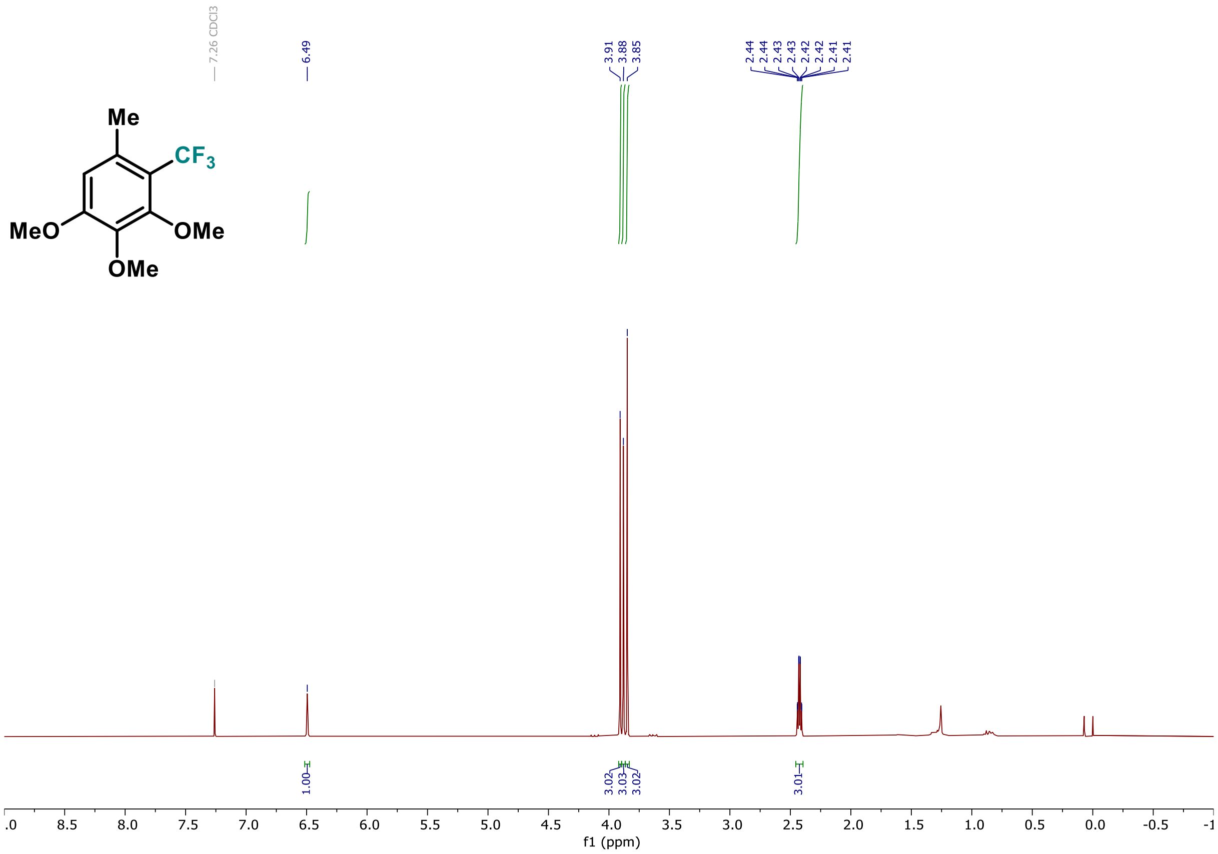

$^{13}C\{^1H\}$ NMR (151 MHz, $CDCl_3$)

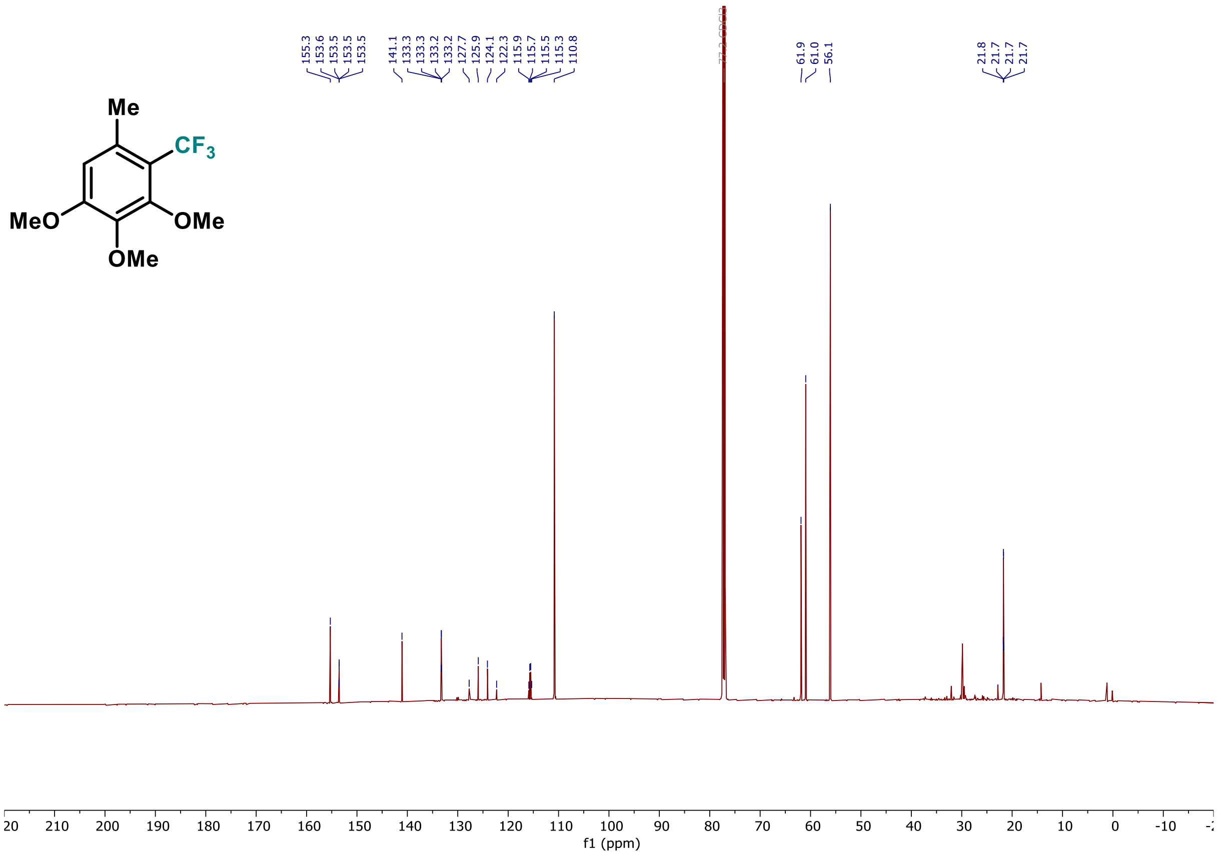

^{19}F NMR (282 MHz, $CDCl_3$)

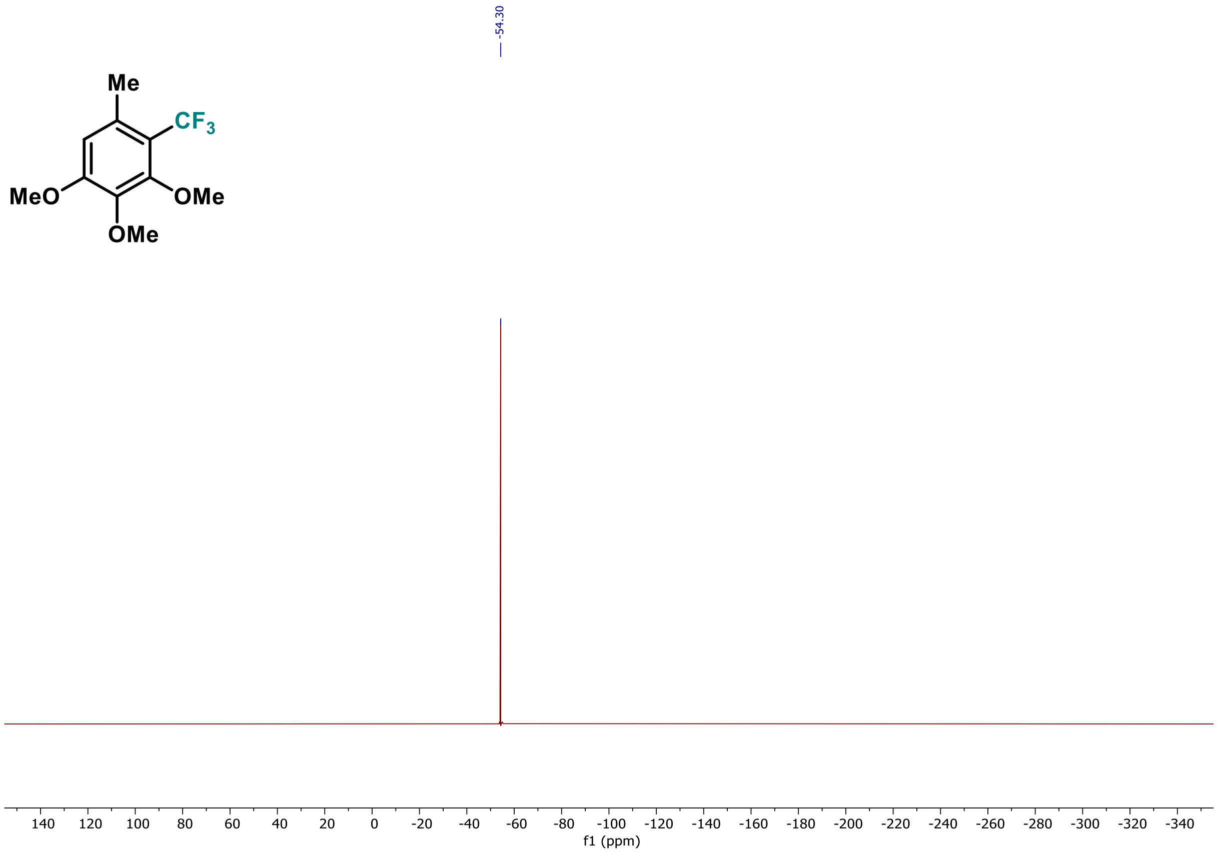

2-bromo-1,3,5-trimethoxy-4-(trifluoromethyl)benzene (4e)

^{1}H NMR (300 MHz, $CDCl_3$)

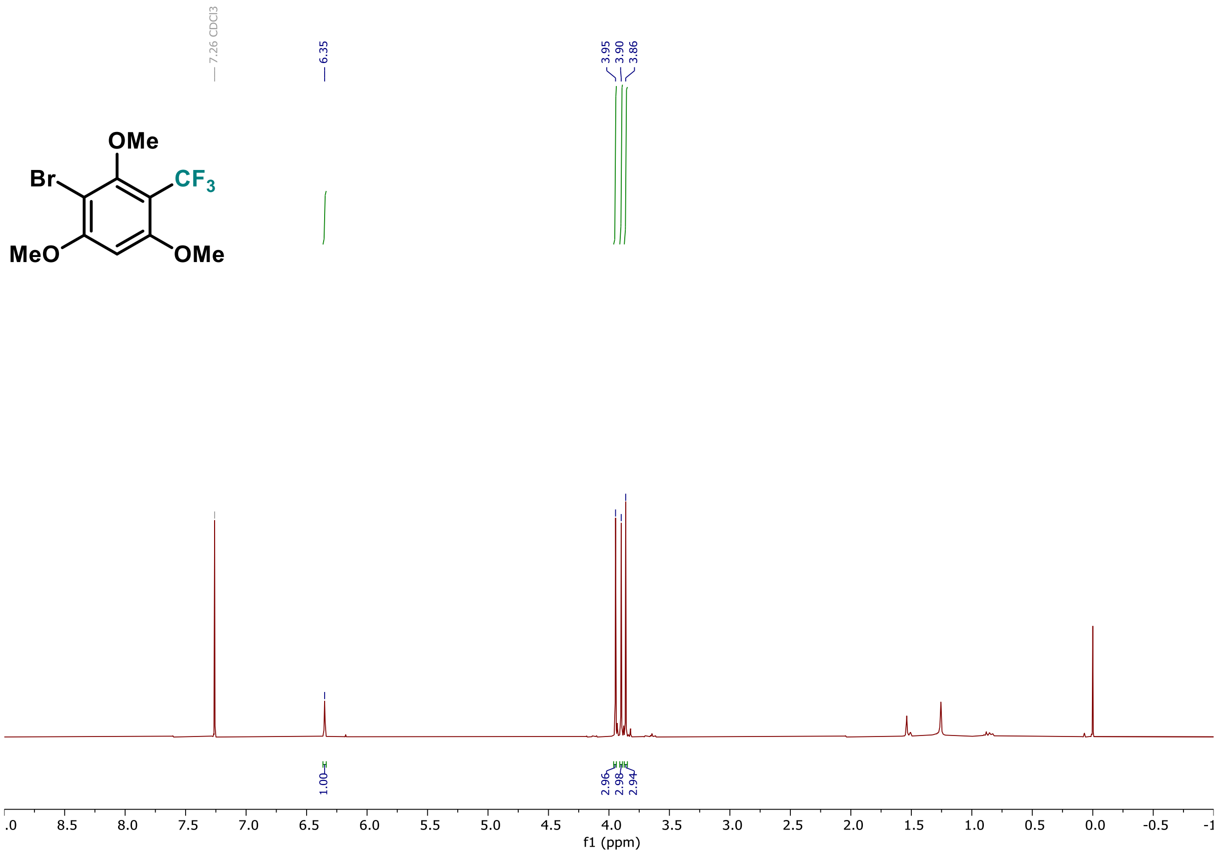

$^{13}C\{^{1}H\}$ NMR (151 MHz, $CDCl_3$)

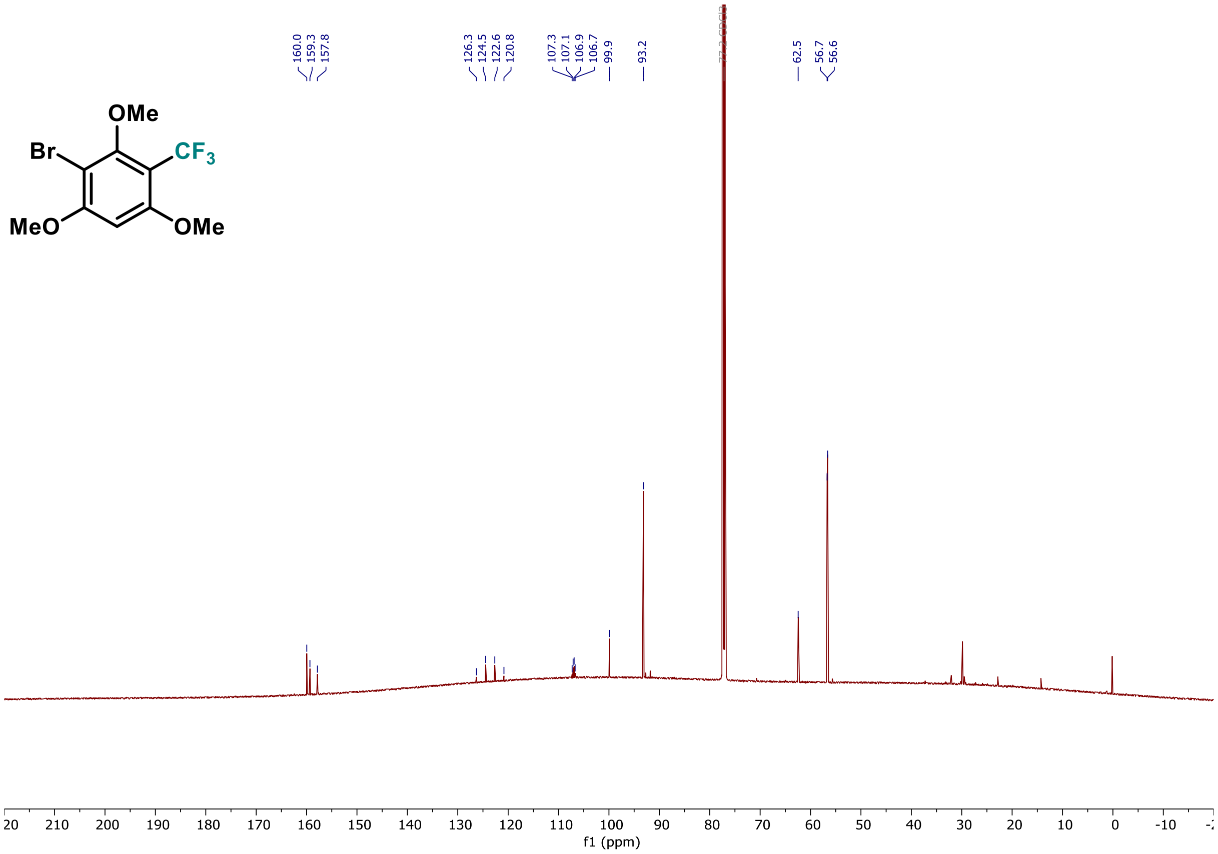

^{19}F NMR (282 MHz, $CDCl_3$)

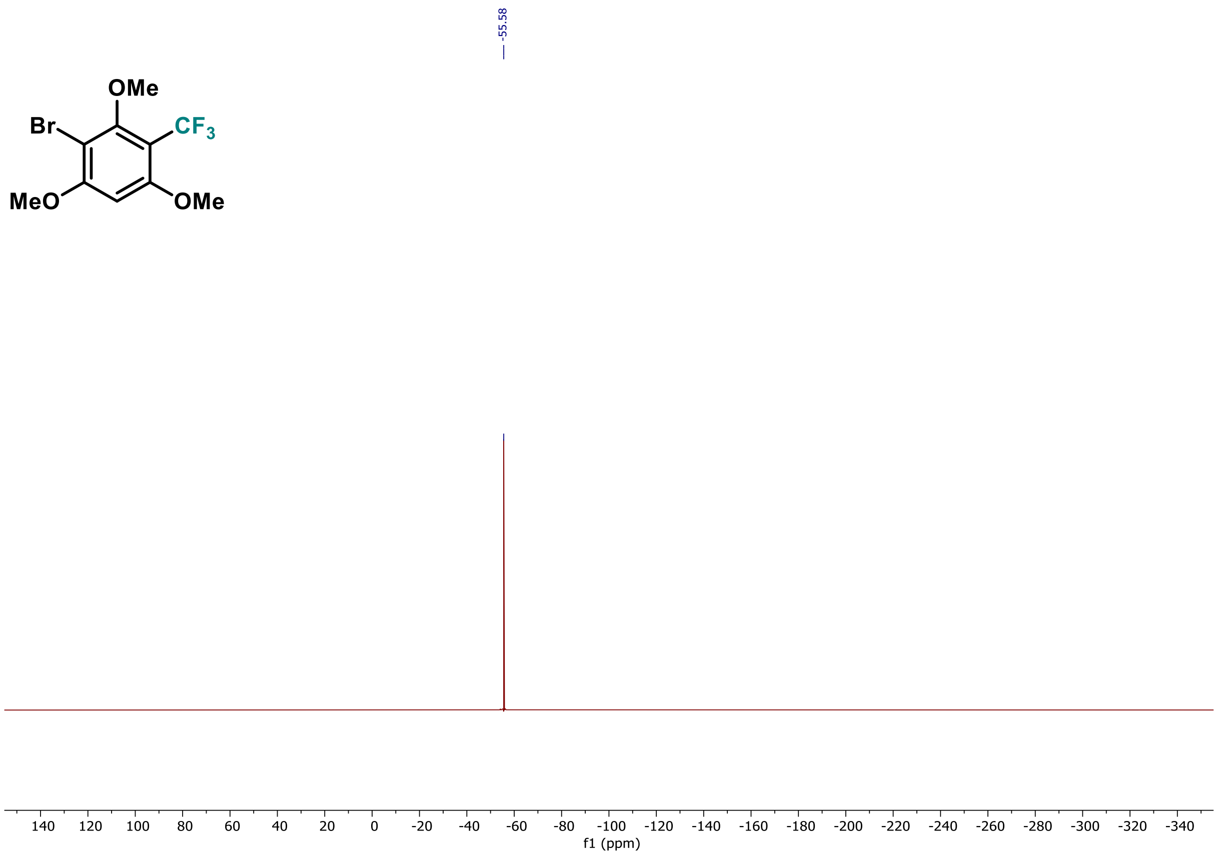

2,4,6-trimethoxy-5-(trifluoromethyl)pyrimidine (4g)

^{1}H NMR (300 MHz, $CDCl_3$)

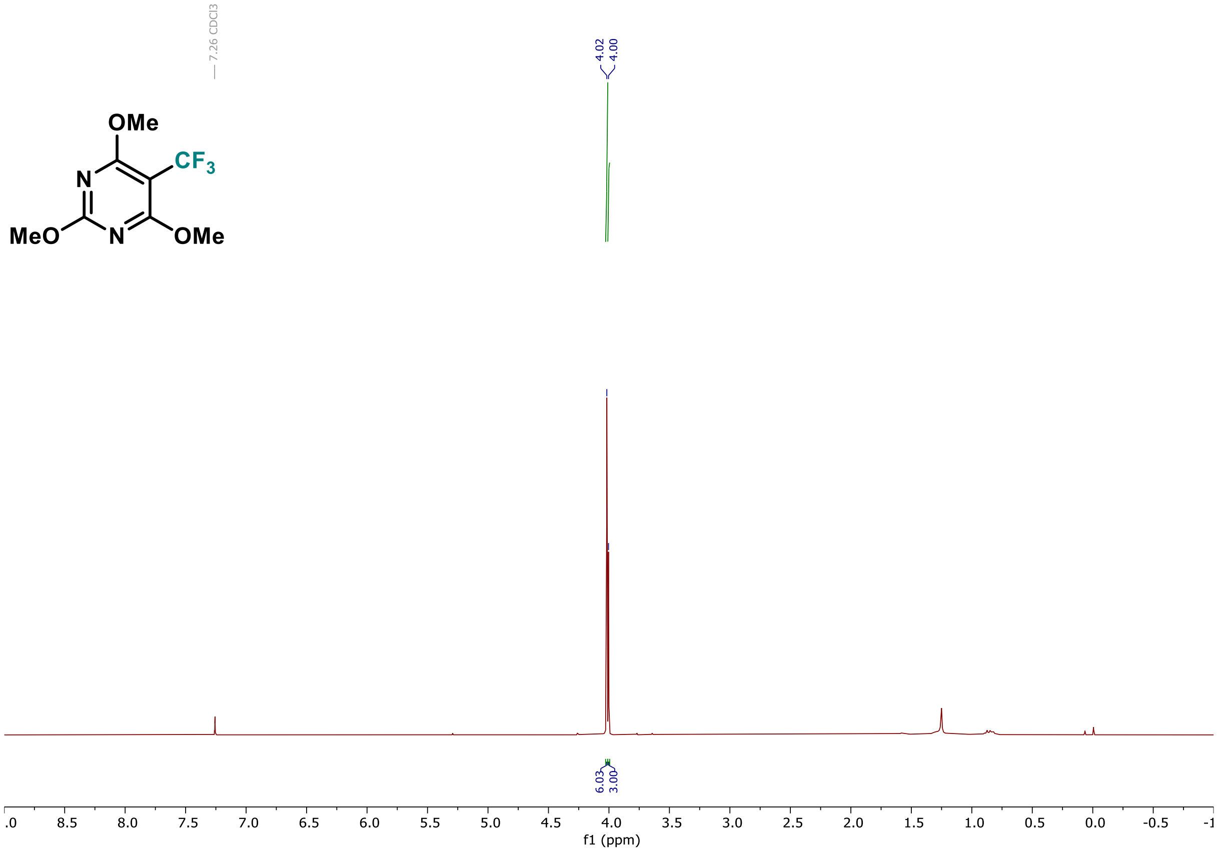

$^{13}C\{^{1}H\}$ NMR (151 MHz, $CDCl_3$)

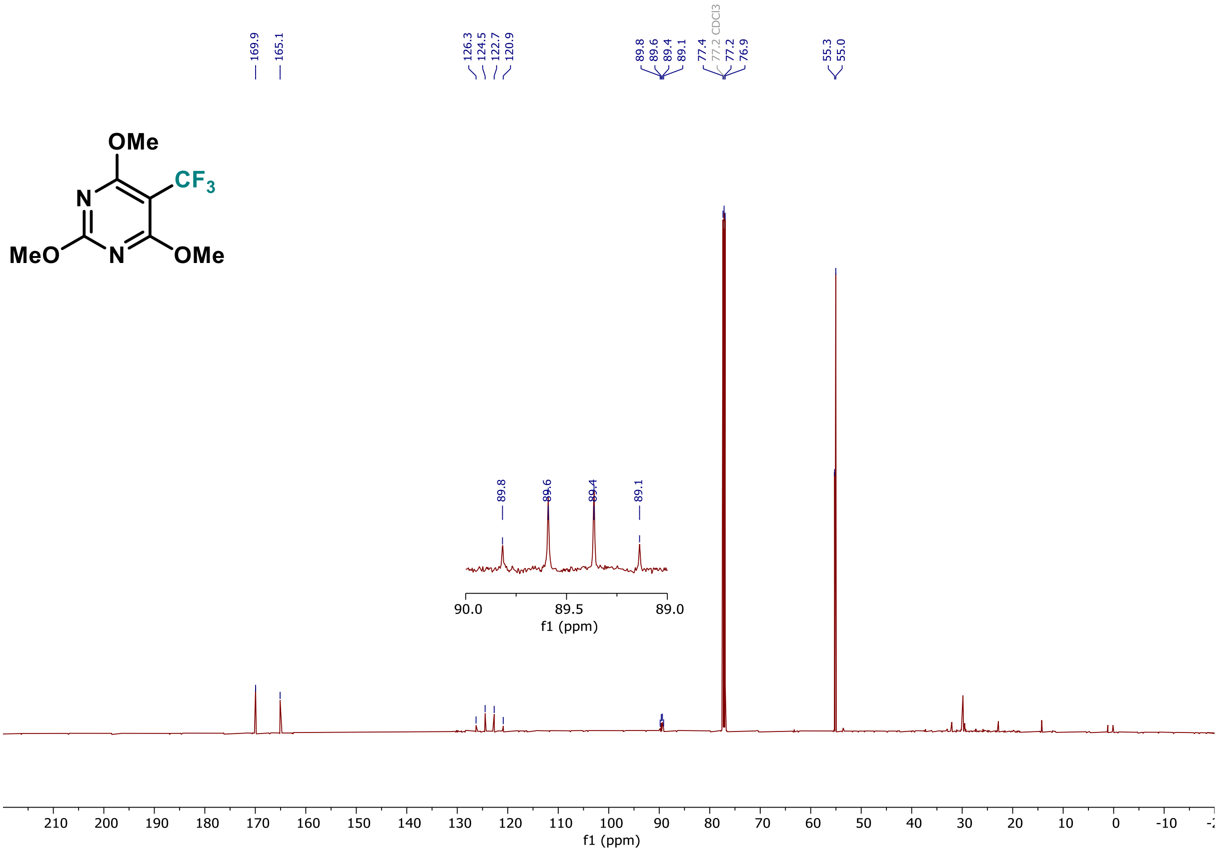

^{19}F NMR (282 MHz, $CDCl_3$)

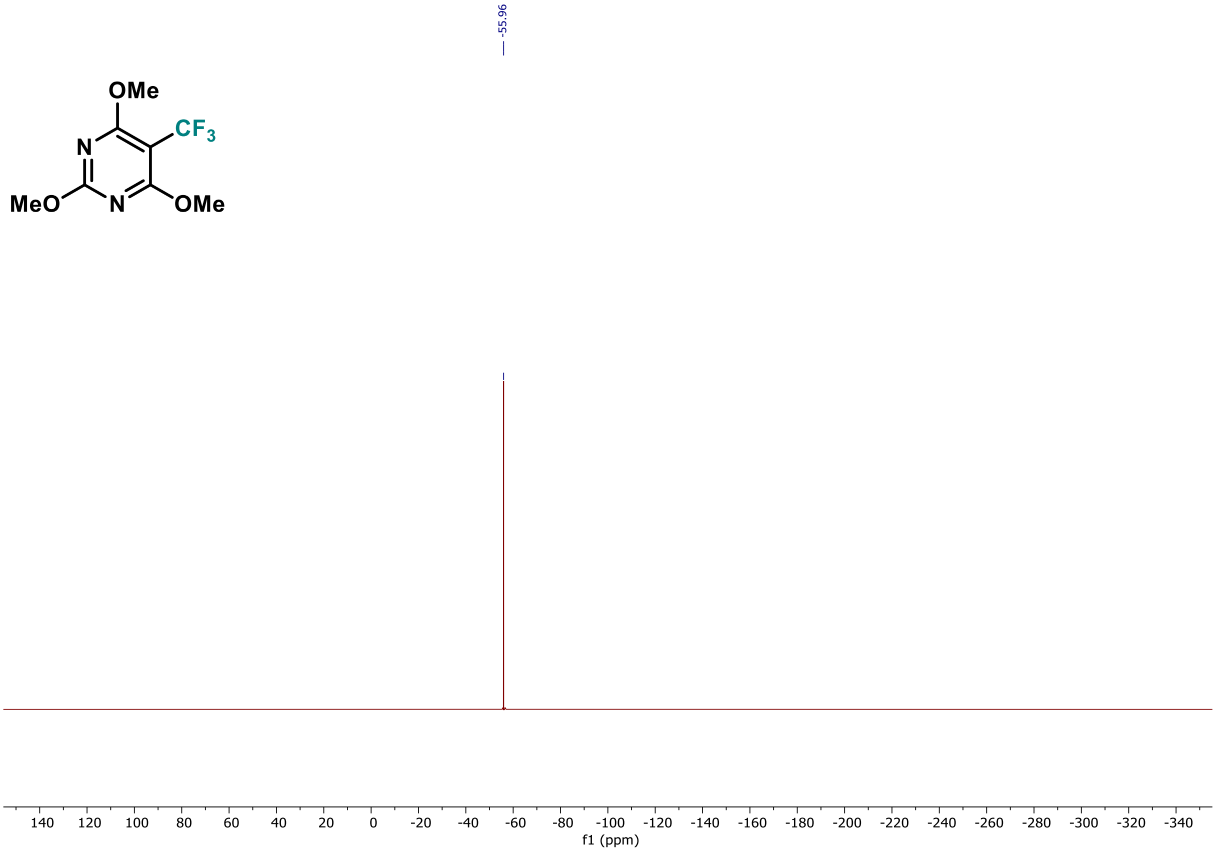

2,6-dimethoxy-3-(trifluoromethyl)pyridine (4h)

^{1}H NMR (300 MHz, $CDCl_3$)

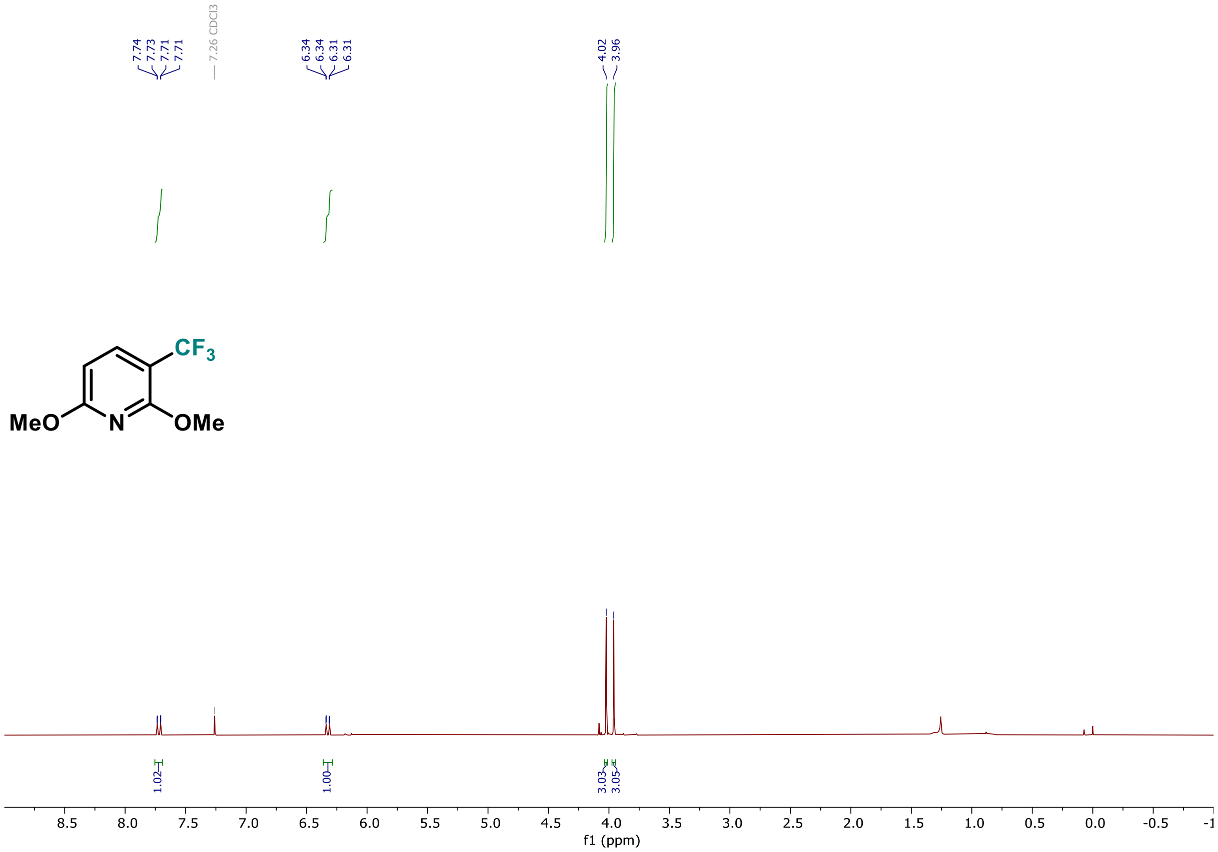

^{13}C{^{1}H} NMR (151 MHz, $CDCl_3$)

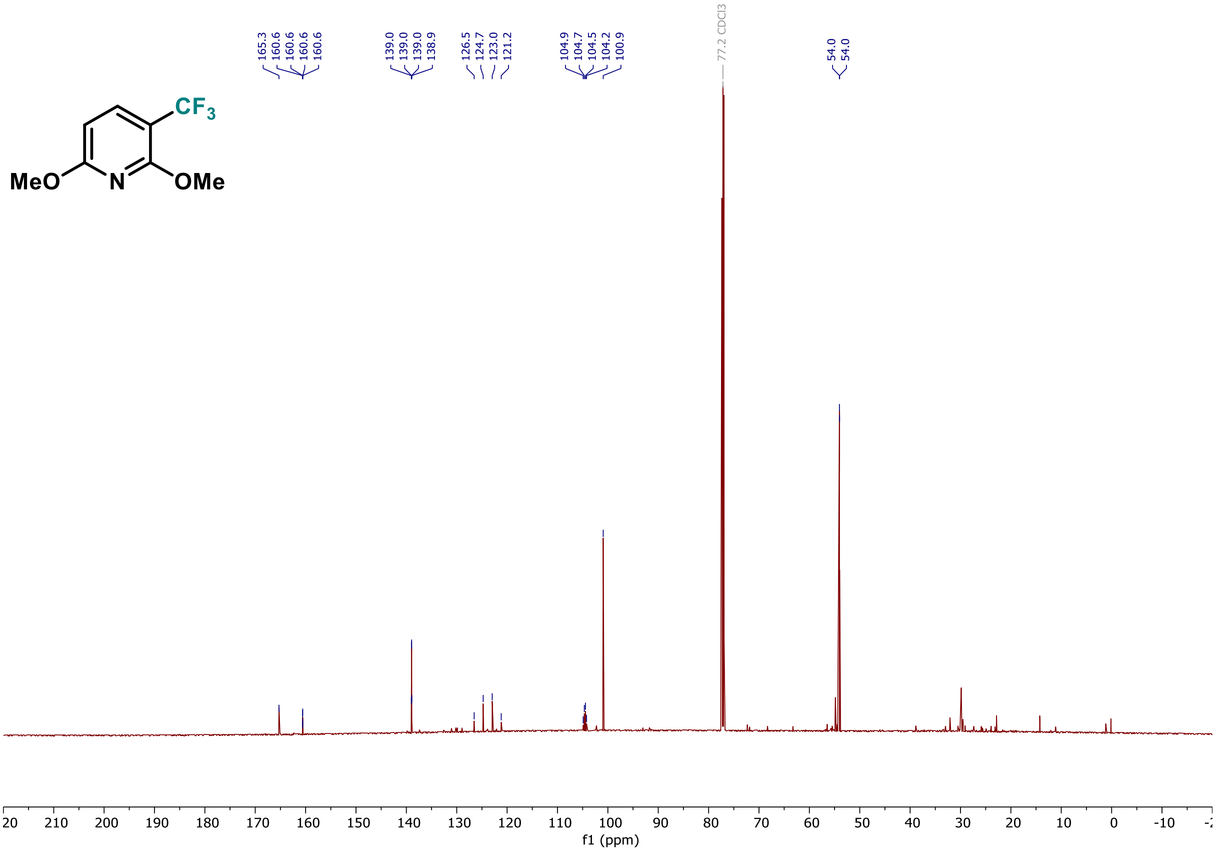

^{19}F NMR (282 MHz, $CDCl_3$)

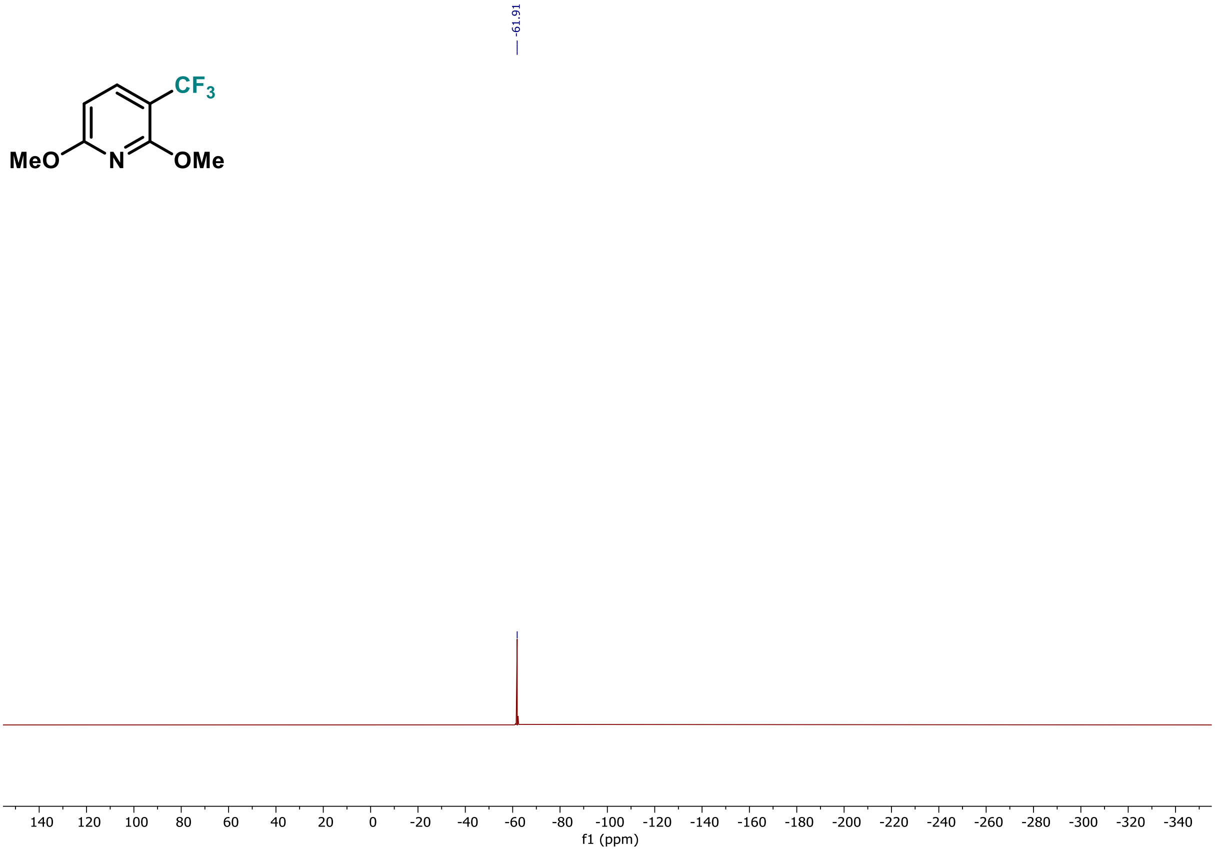

4,6-dimethyl-5-(trifluoromethyl)-2H-pyran-2-one (4j)

^{1}H NMR (300 MHz, $CDCl_3$)

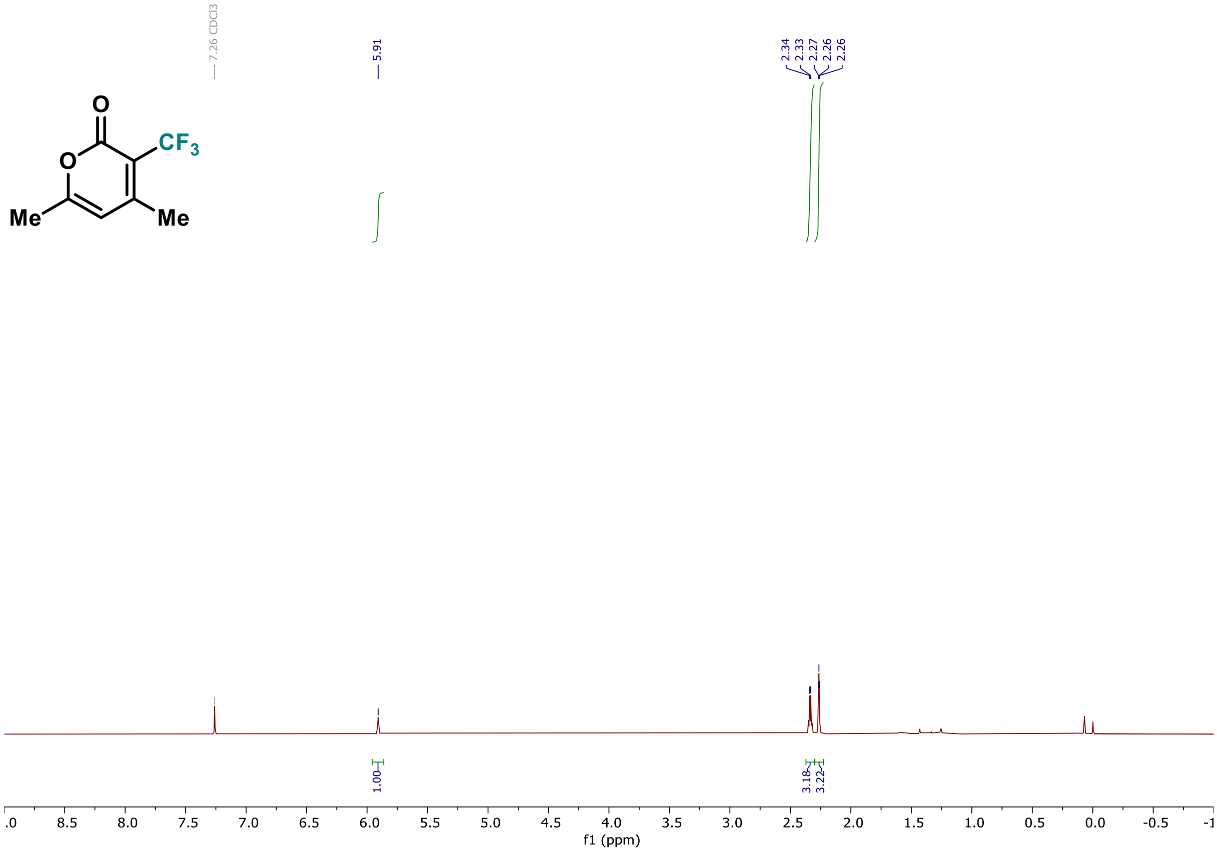

$^{13}C\{^{1}H\}$ NMR (151 MHz, $CDCl_3$)

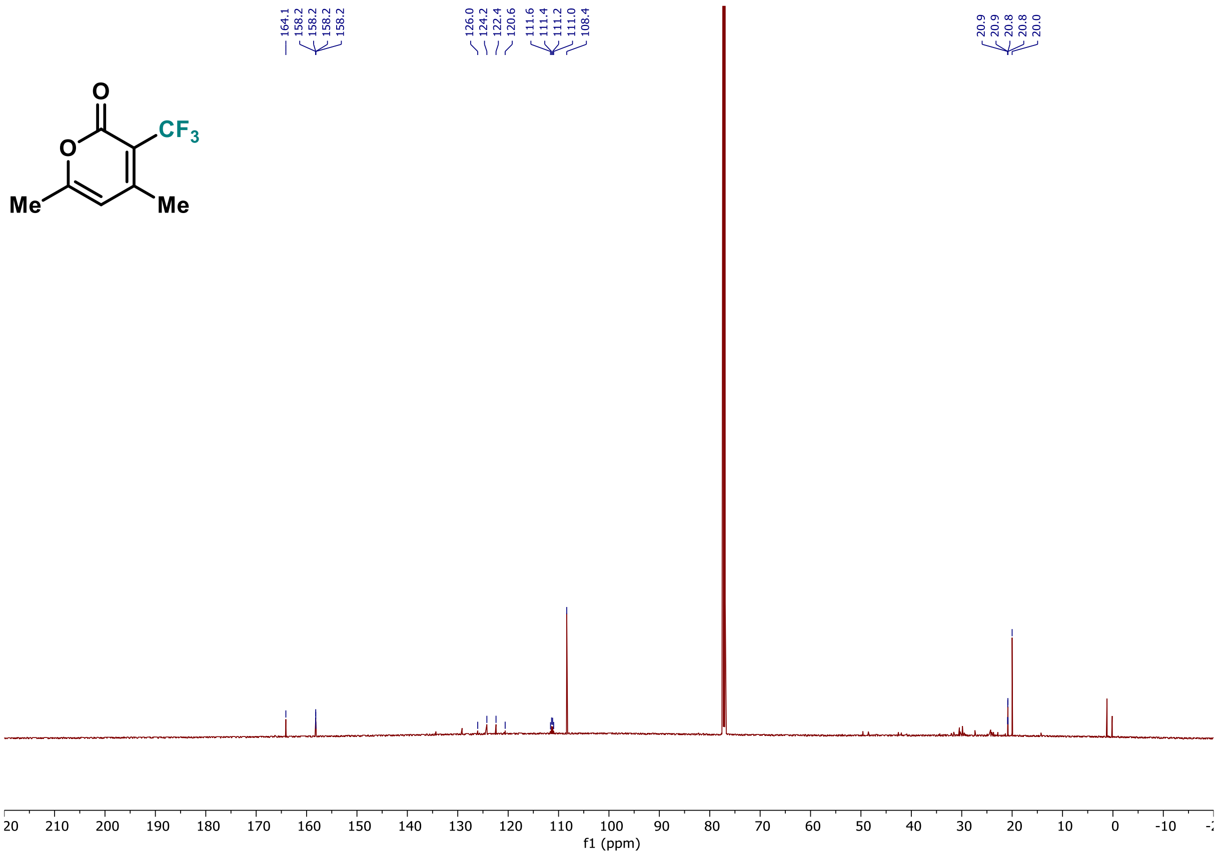

^{19}F NMR (282 MHz, $CDCl_3$)

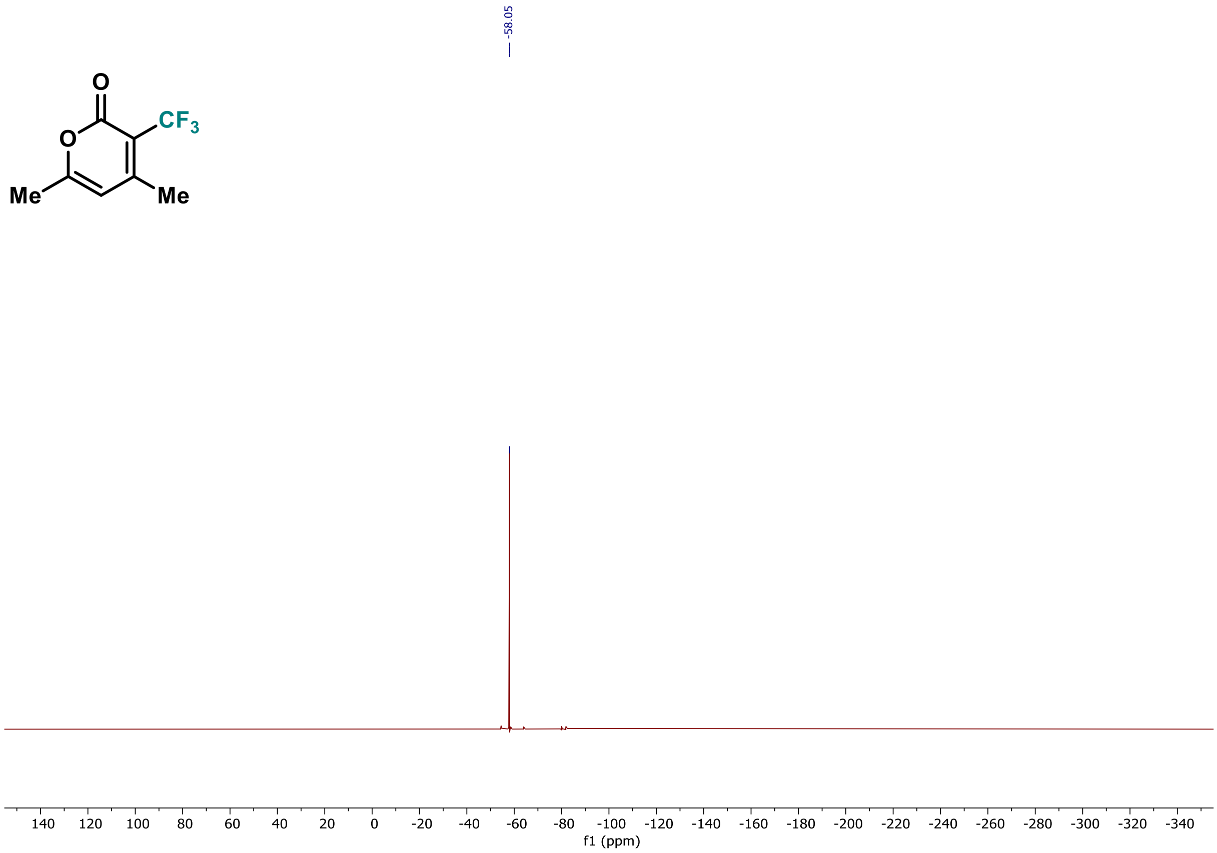

^{1}H-^{13}C HSQC

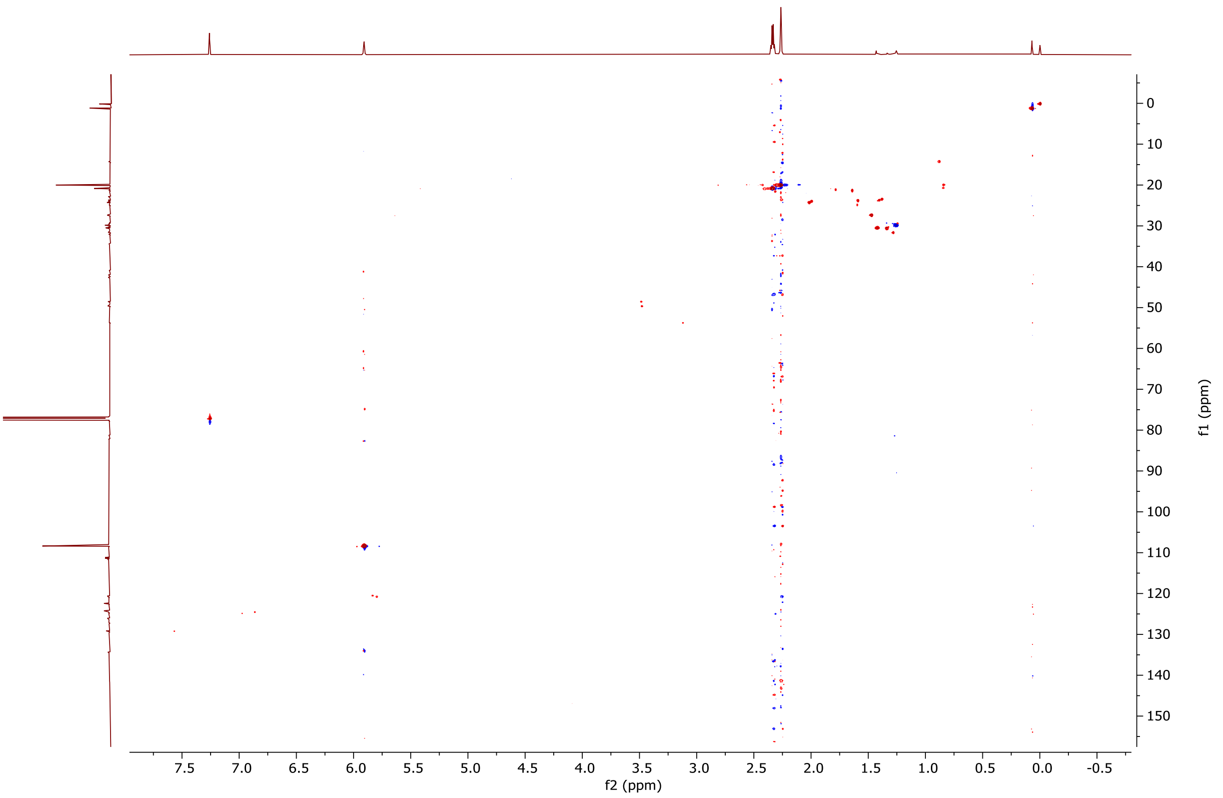

^{1}H-^{13}C HMBC

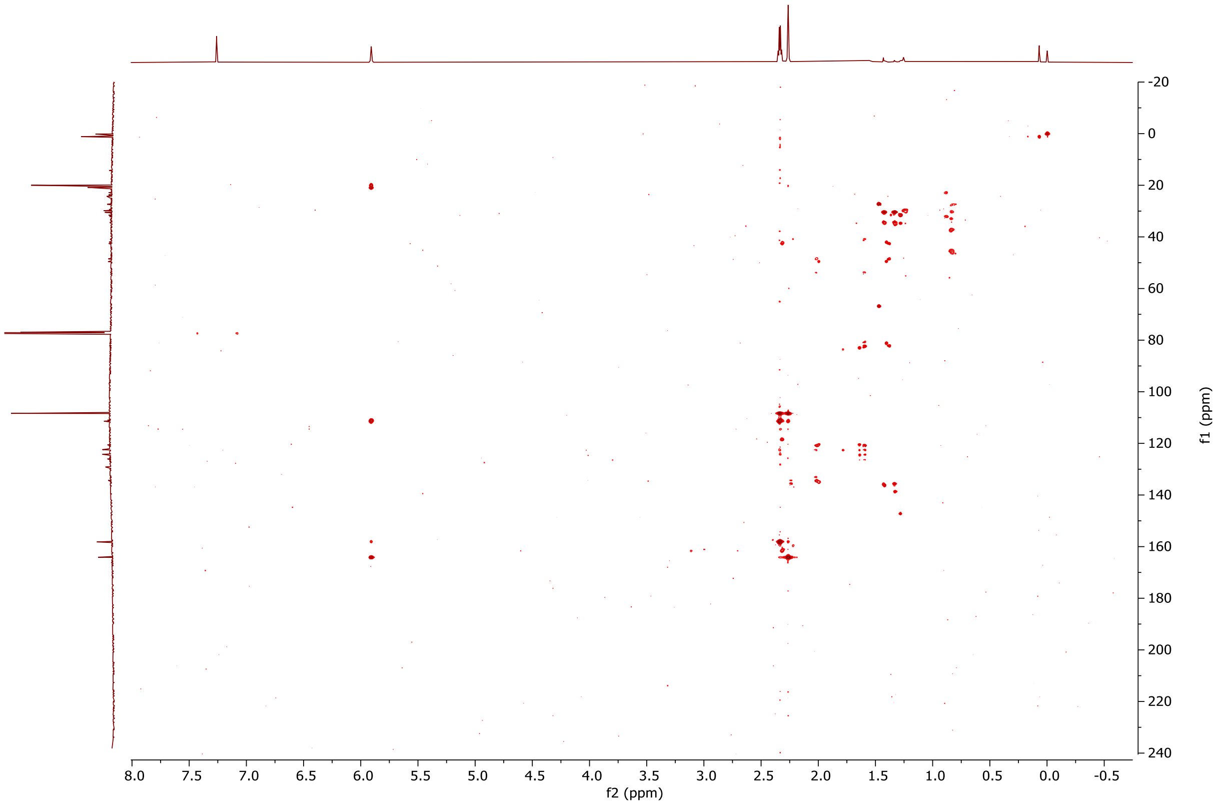

tert-butyl 2-(trifluoromethyl)-1_H_-pyrrole-1-carboxylate (4l)

^{1}H NMR (600 MHz, $CDCl_3$)

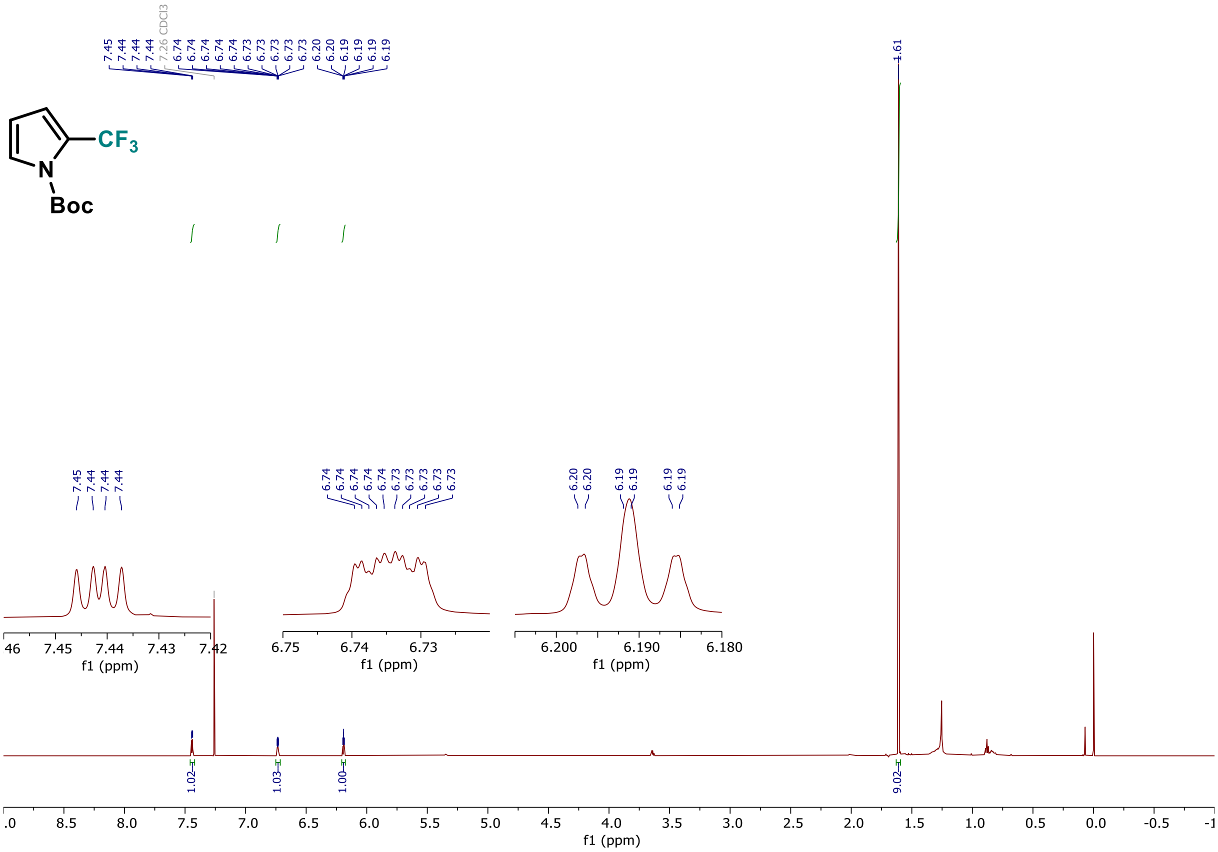

^{13}C{^{1}H} NMR (151 MHz, $CDCl_3$)

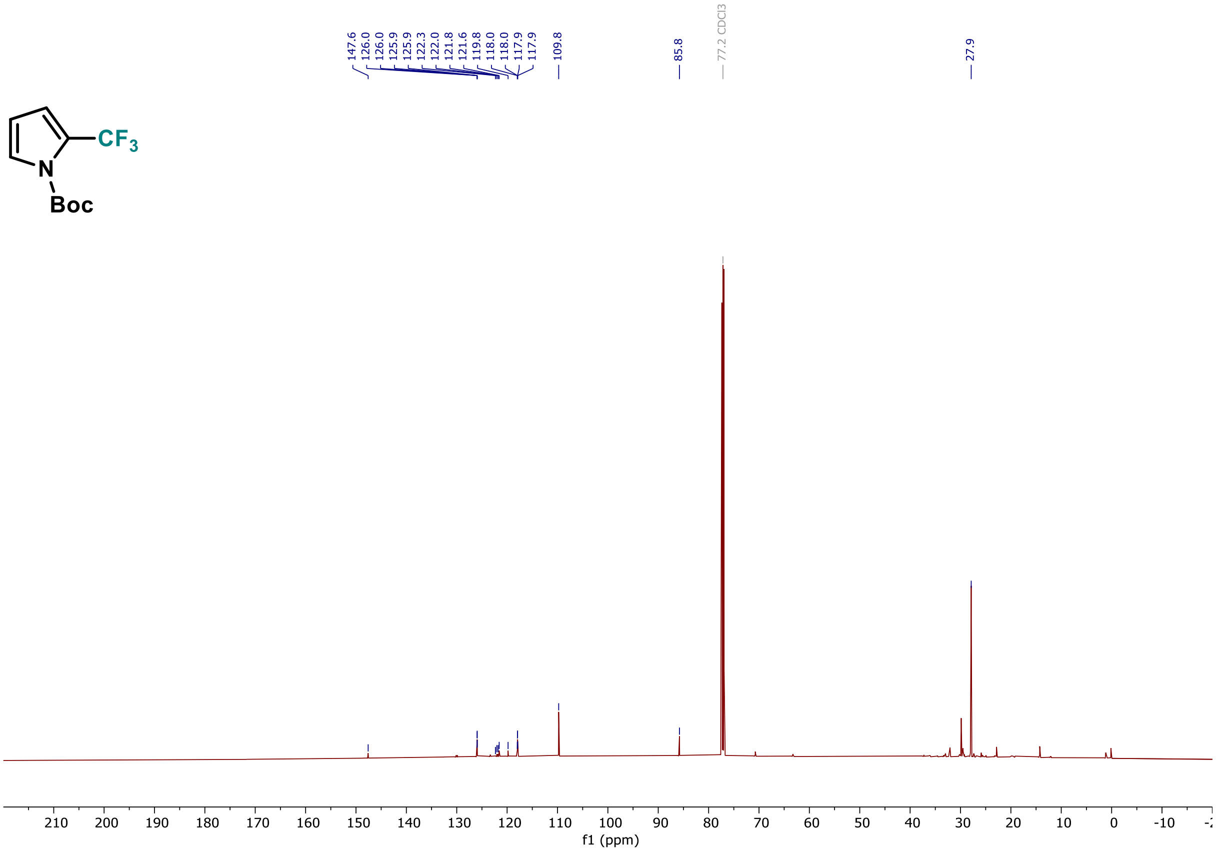

^{19}F NMR (565 MHz, $CDCl_3$)

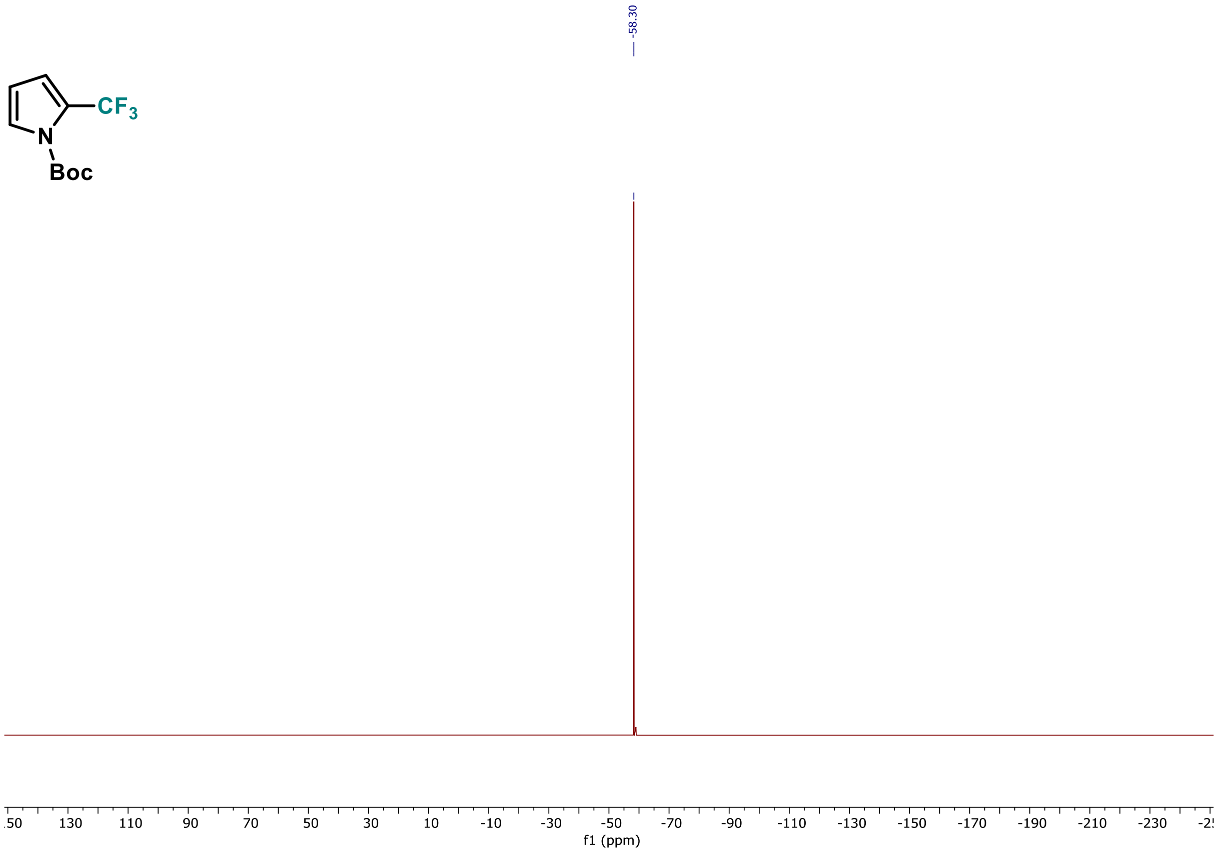

3,4-dimethoxy-2-(trifluoromethyl)thiophene (4m)

^{1}H NMR (600 MHz, $CDCl_3$)

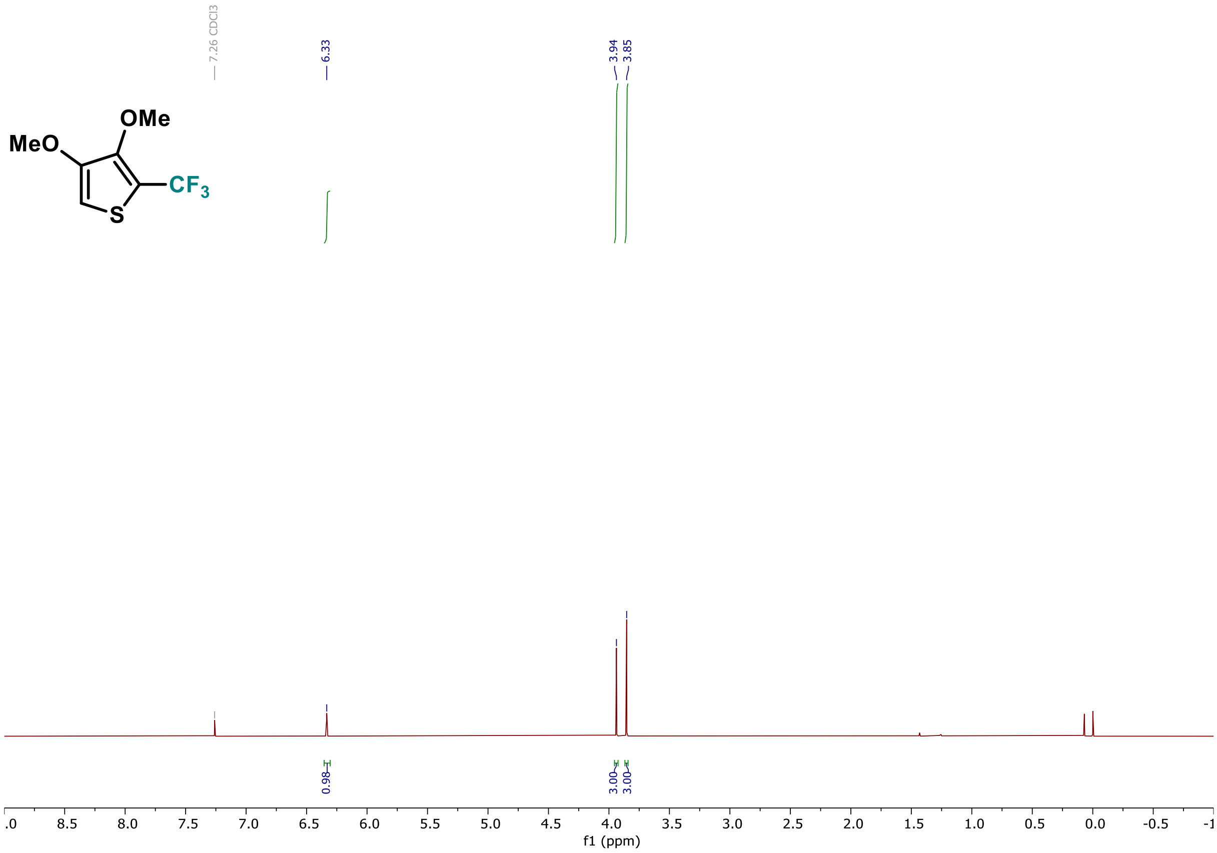

$^{13}C\{^{1}H\}$ NMR (151 MHz, $CDCl_3$)

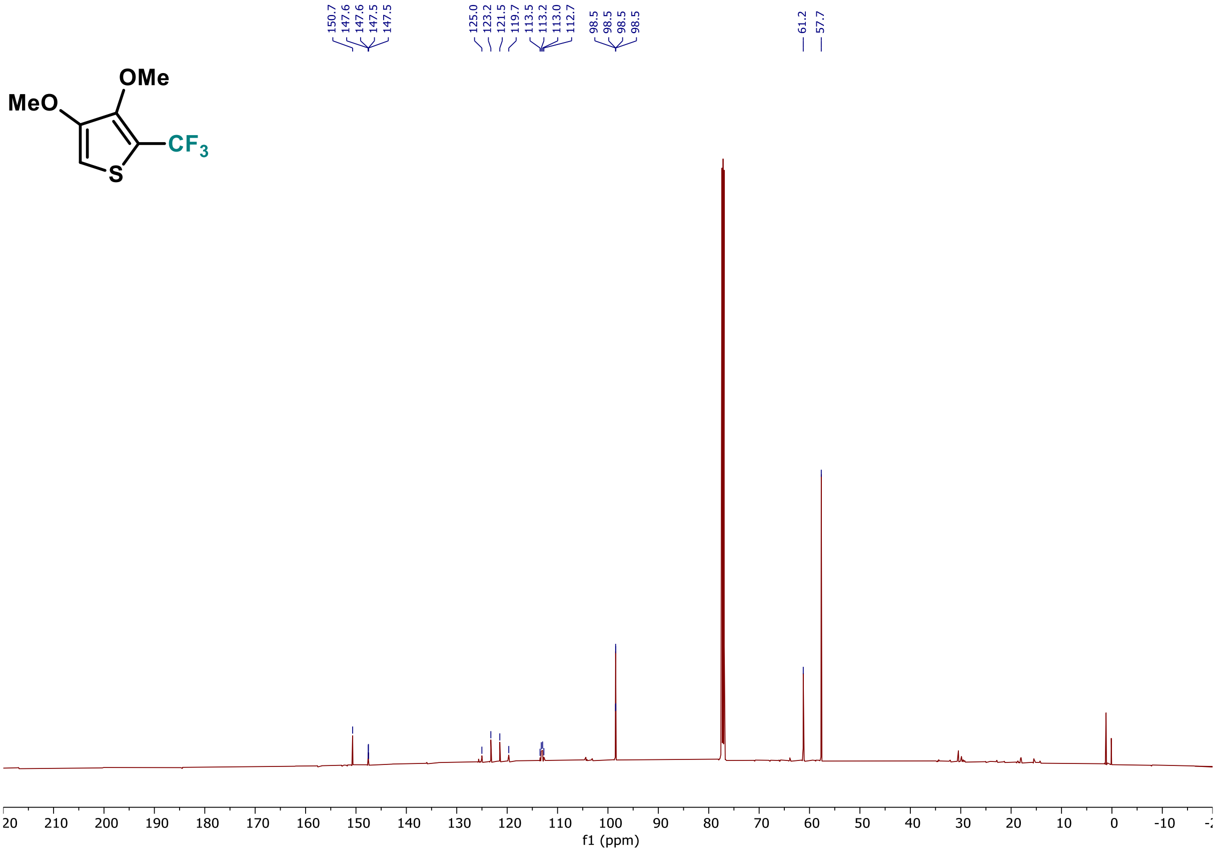

^{19}F NMR (565 MHz, $CDCl_3$)

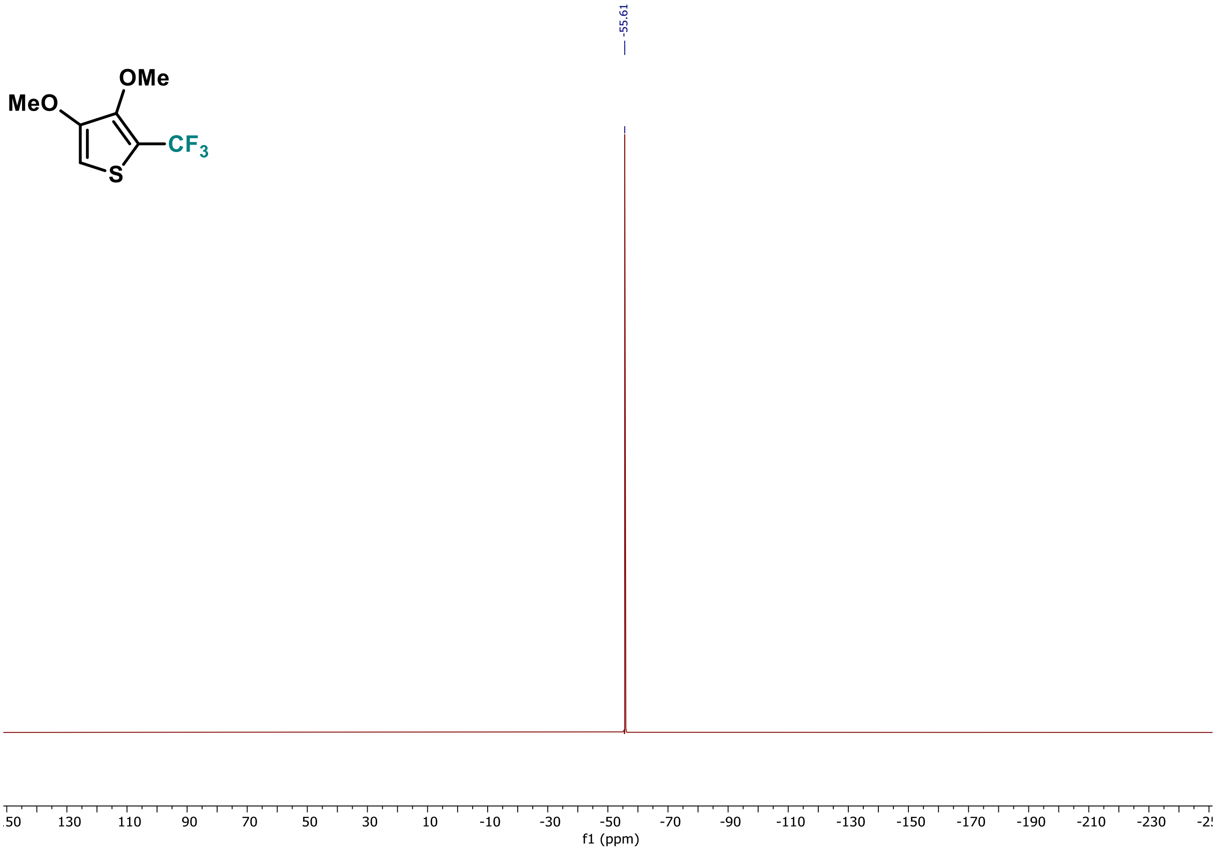

1-benzyl-3-methyl-2-(trifluoromethyl)-1*H*-indole (4n)

^{1}H NMR (300 MHz, $CDCl_3$)

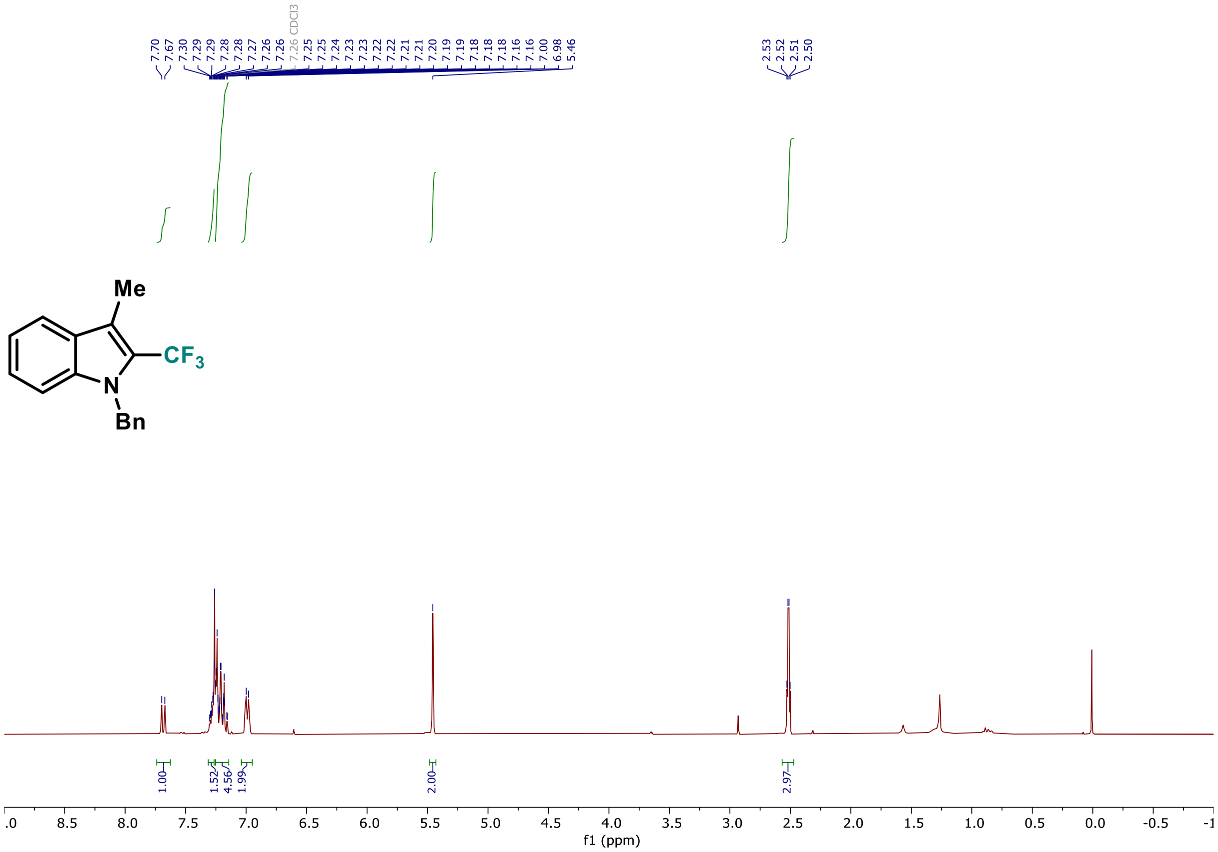

$^{13}C\{^1H\}$ NMR (151 MHz, $CDCl_3$)

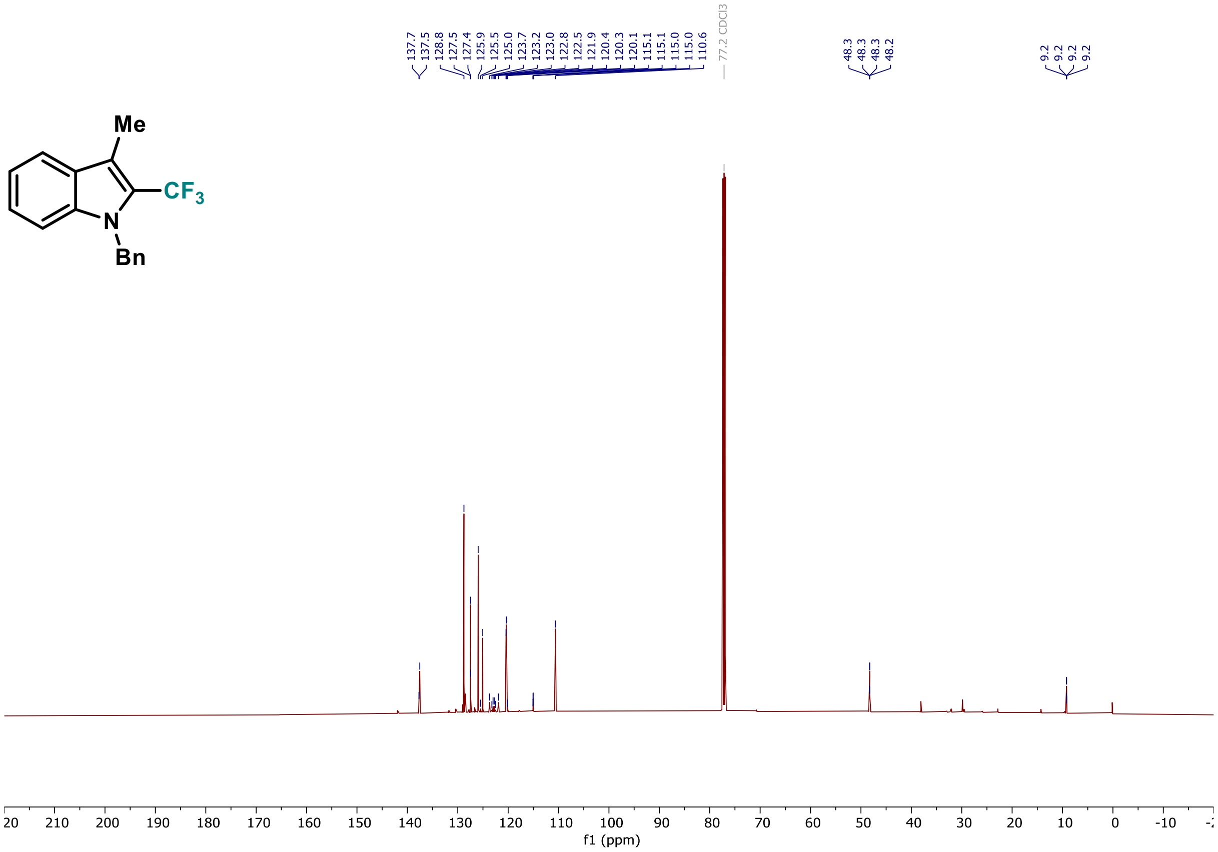

^{19}F NMR (282 MHz, $CDCl_3$)

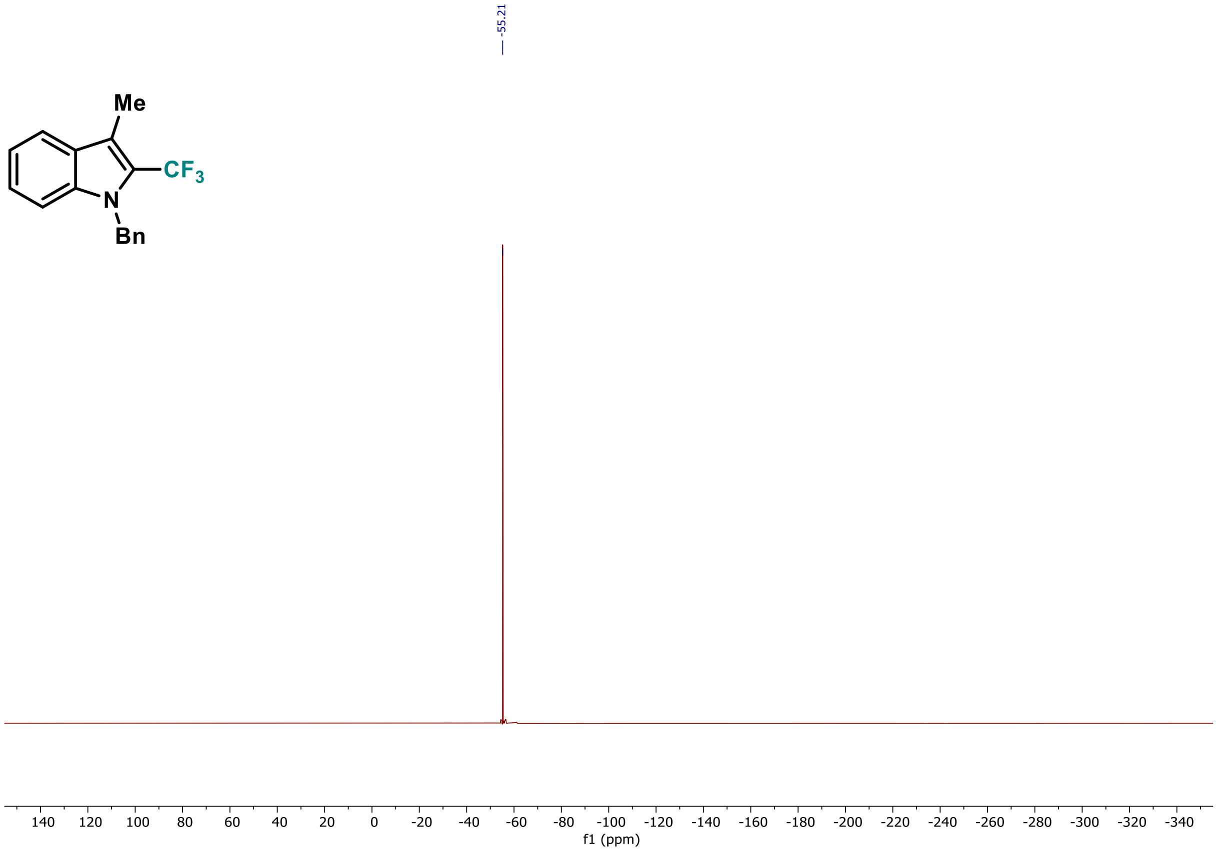

(*R*)-3,6-dimethyl-2-(trifluoromethyl)-4,5,6,7-tetrahydrobenzofuran (4o)

^{1}H NMR (600 MHz, $CDCl_3$)

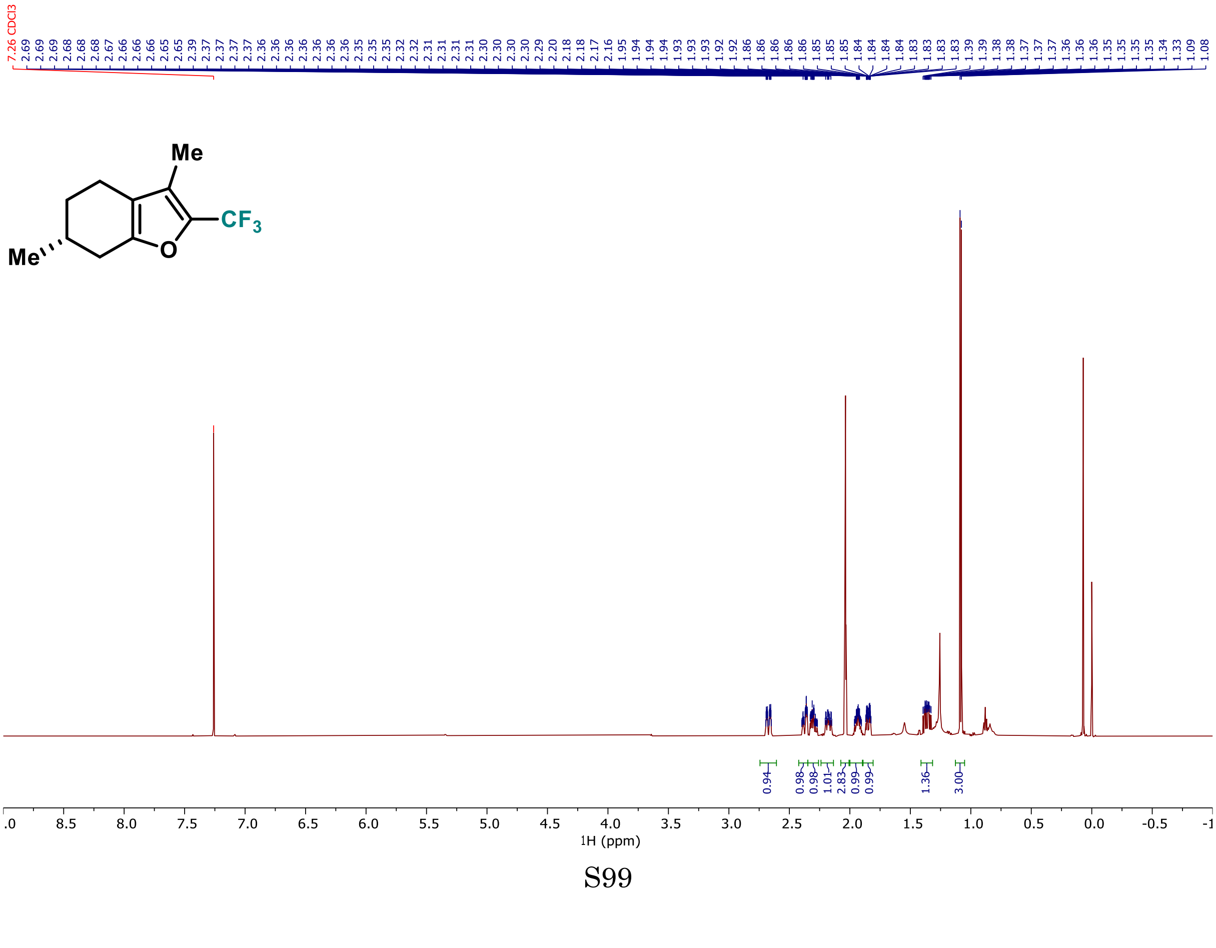

$^{13}C\{^{1}H\}$ NMR (151 MHz, $CDCl_3$)

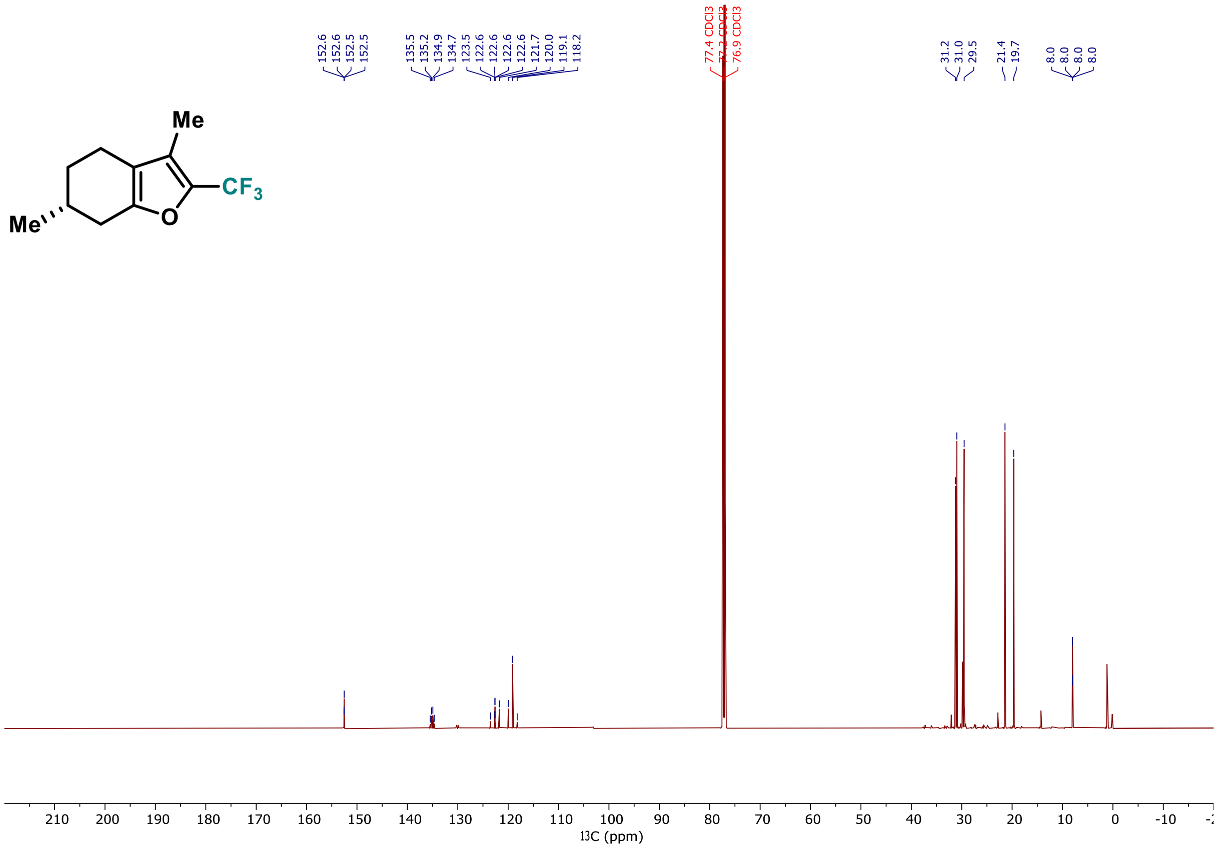

^{19}F NMR (565 MHz, $CDCl_3$)

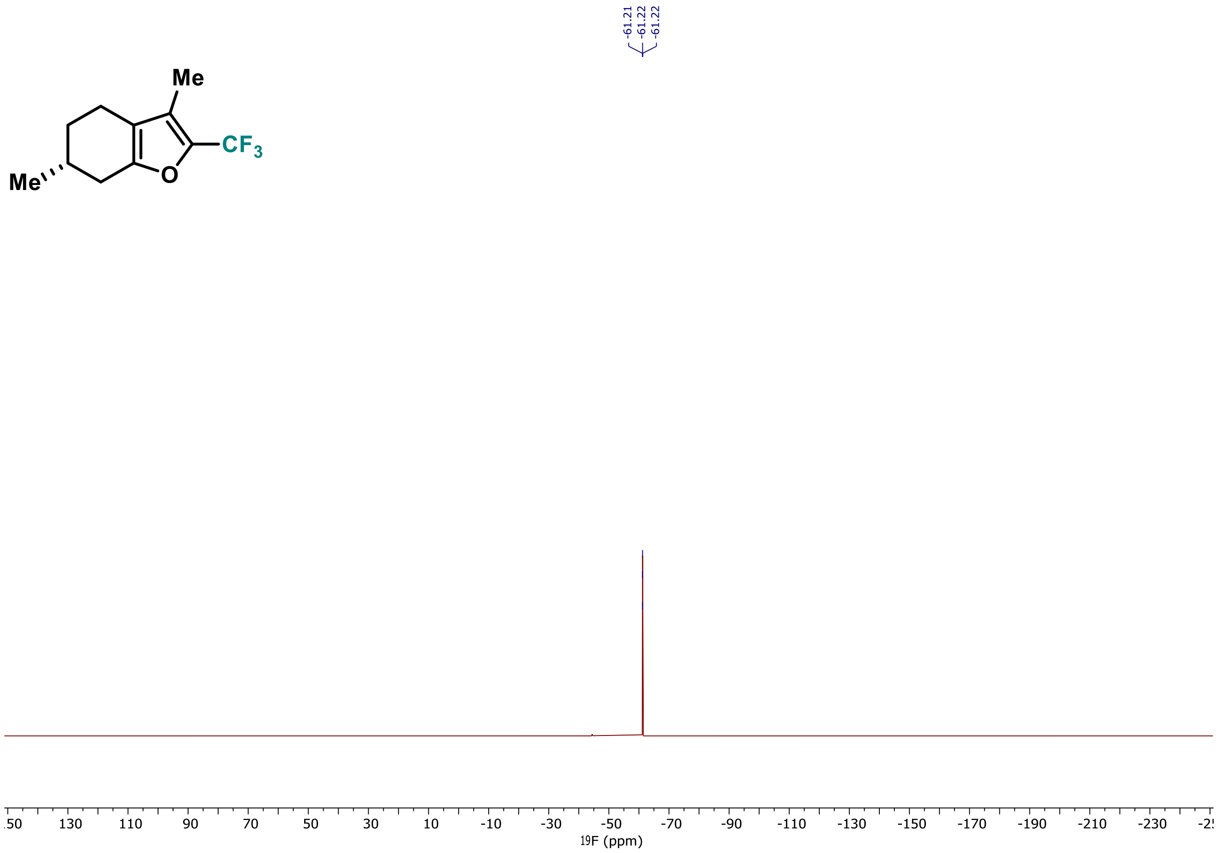

^{1}H-^{13}C HSQC

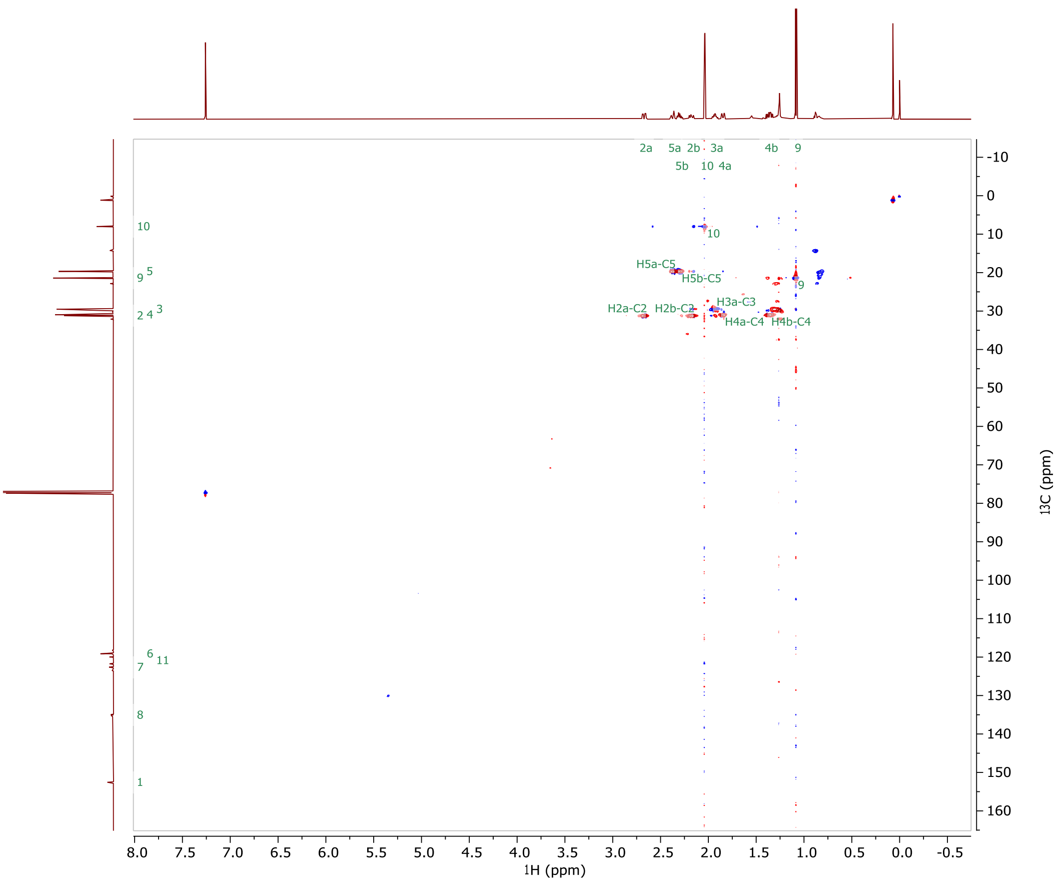

^{1}H-^{13}C HMBC

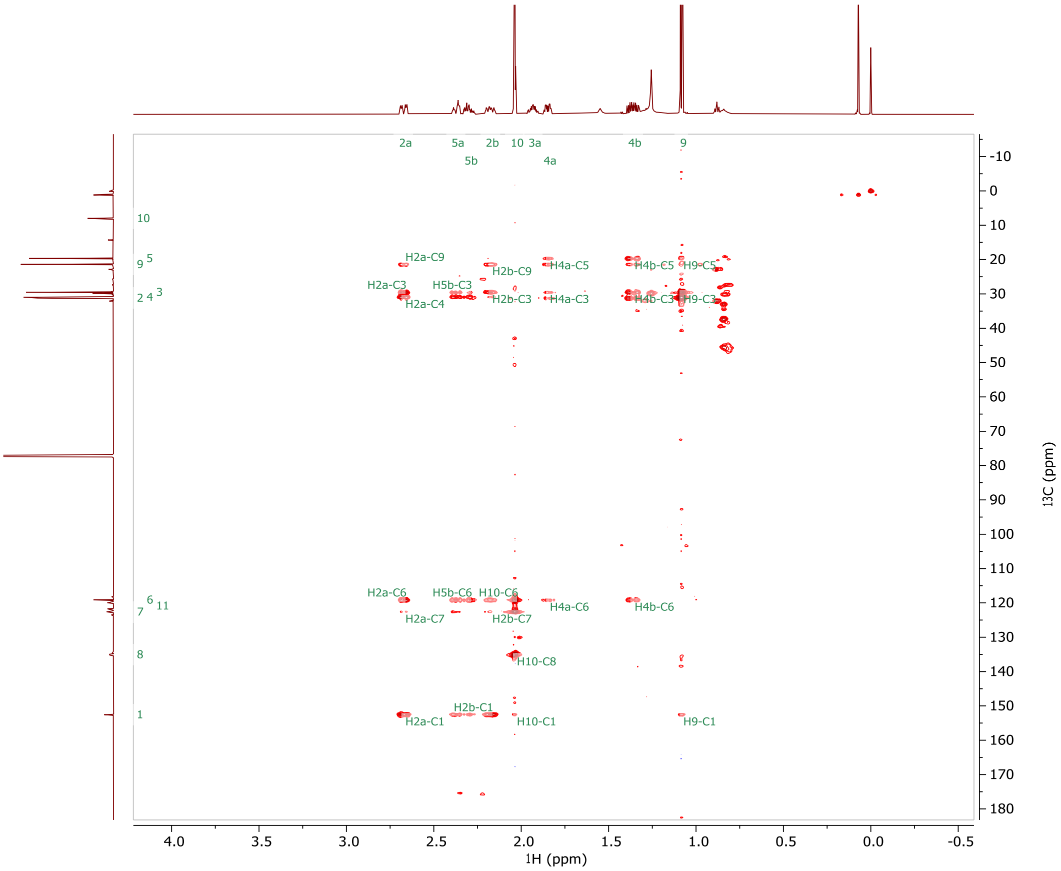

^{1}H-^{1}H COSY

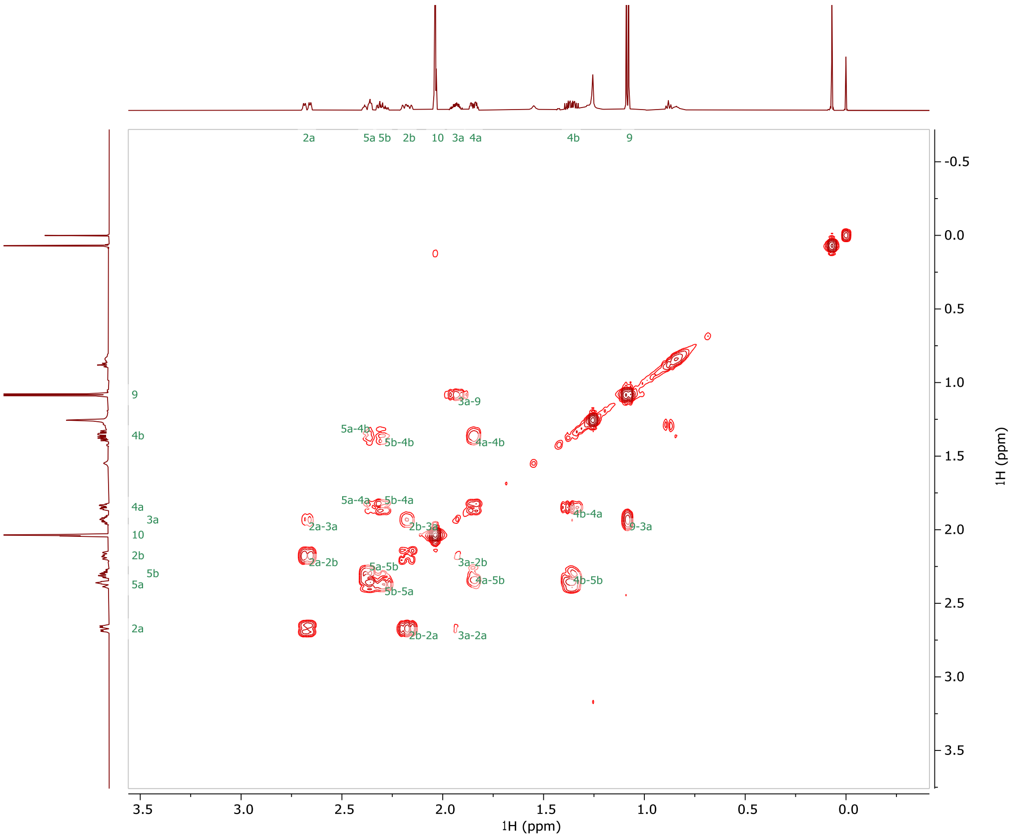

^{1}H-^{1}H NOESY

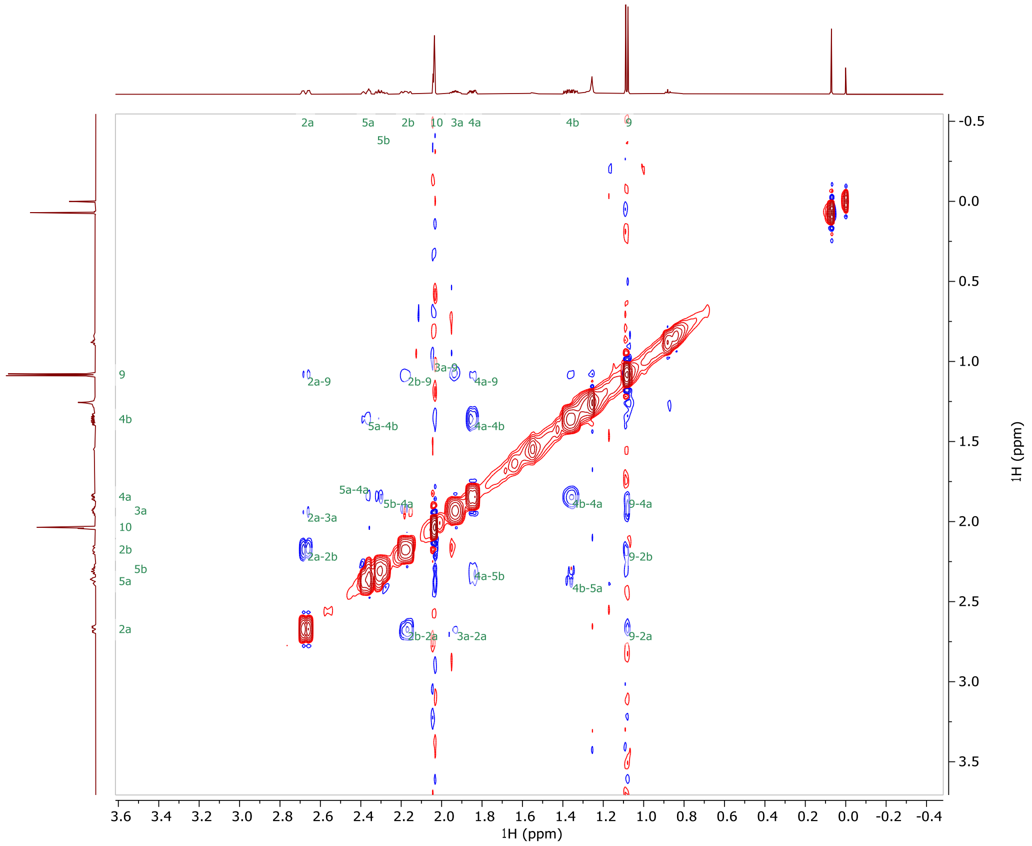

1,3,7-trimethyl-8-(trifluoromethyl)-3,7-dihydro-1H-purine-2,6-dione (4p)

^{1}H NMR (300 MHz, $CDCl_3$)

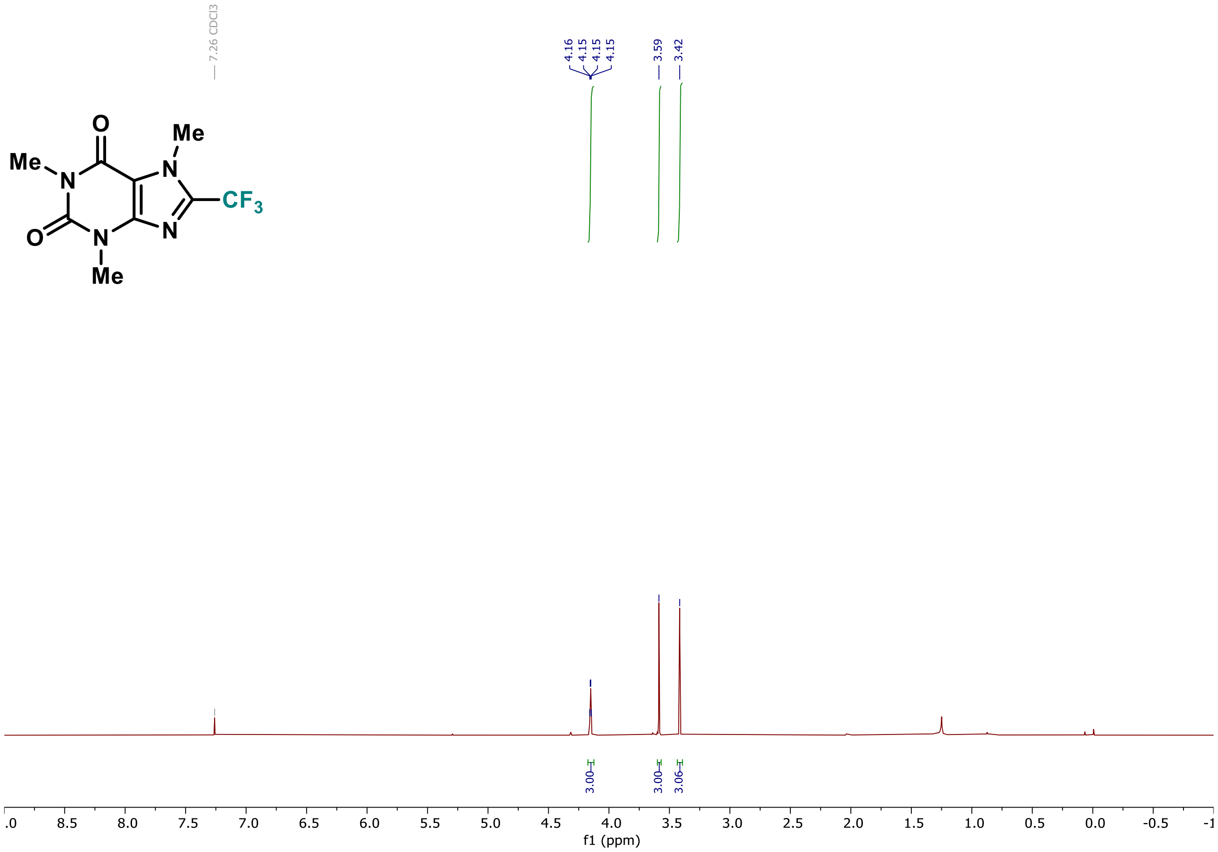

^{13}C{^{1}H} NMR (151 MHz, $CDCl_3$)

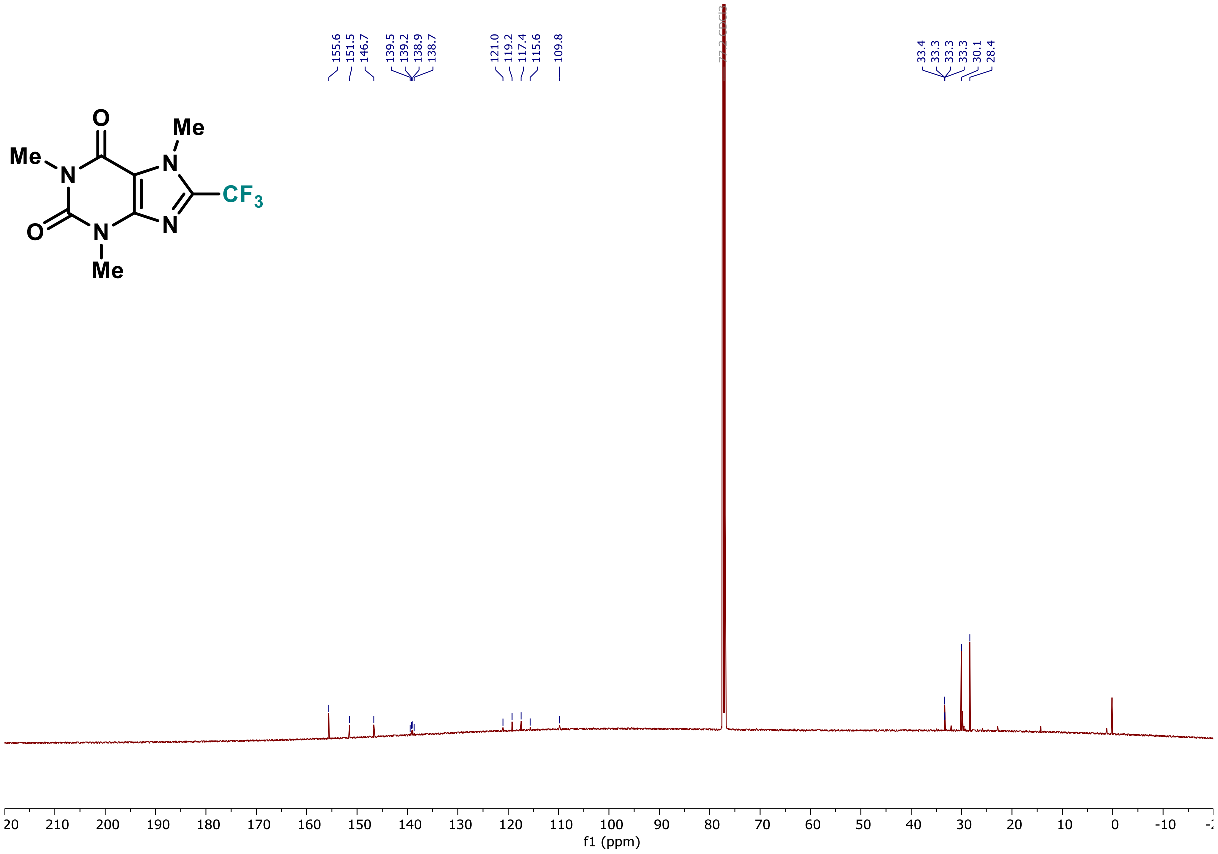

^{19}F NMR (282 MHz, $CDCl_3$)

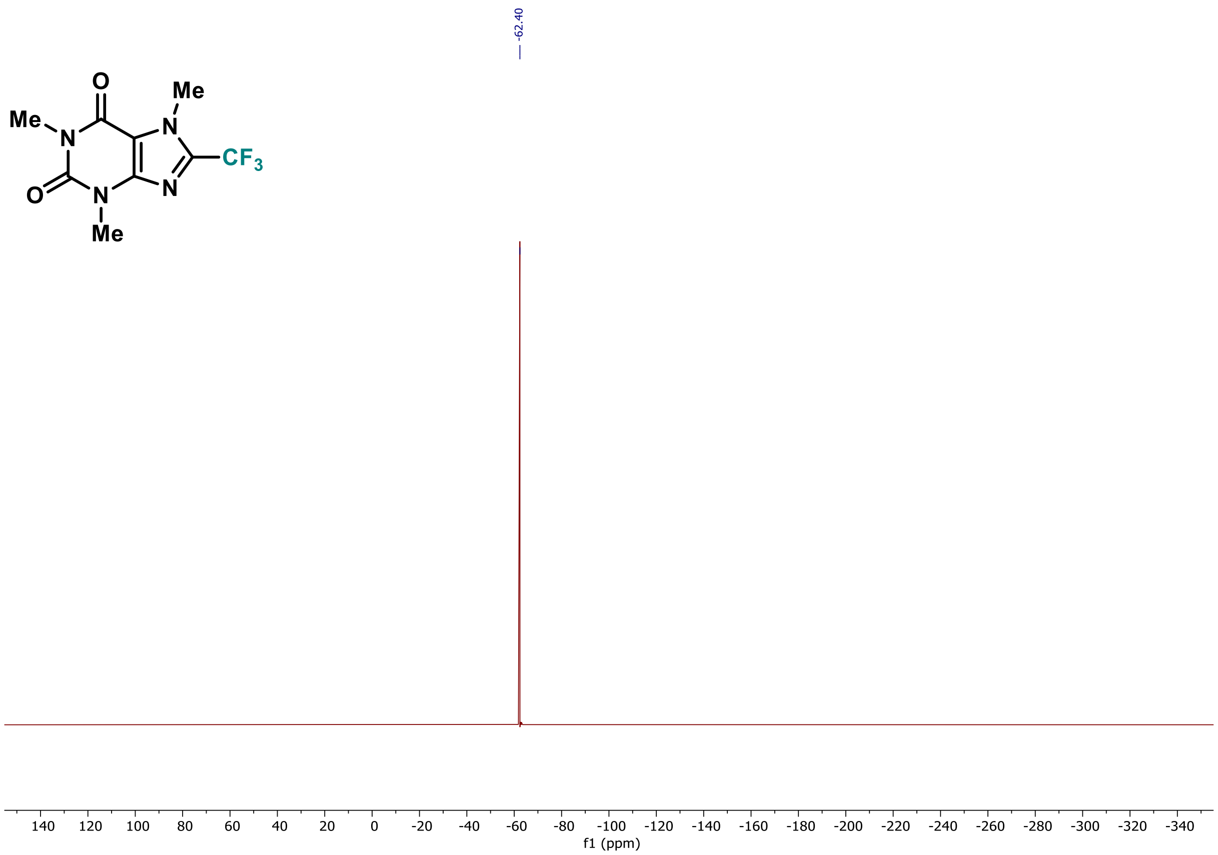

1,3-dimethyl-5-(trifluoromethyl)pyrimidine-2,4(1H,3H)-dione (4q)

^{1}H NMR (600 MHz, $CDCl_3$)

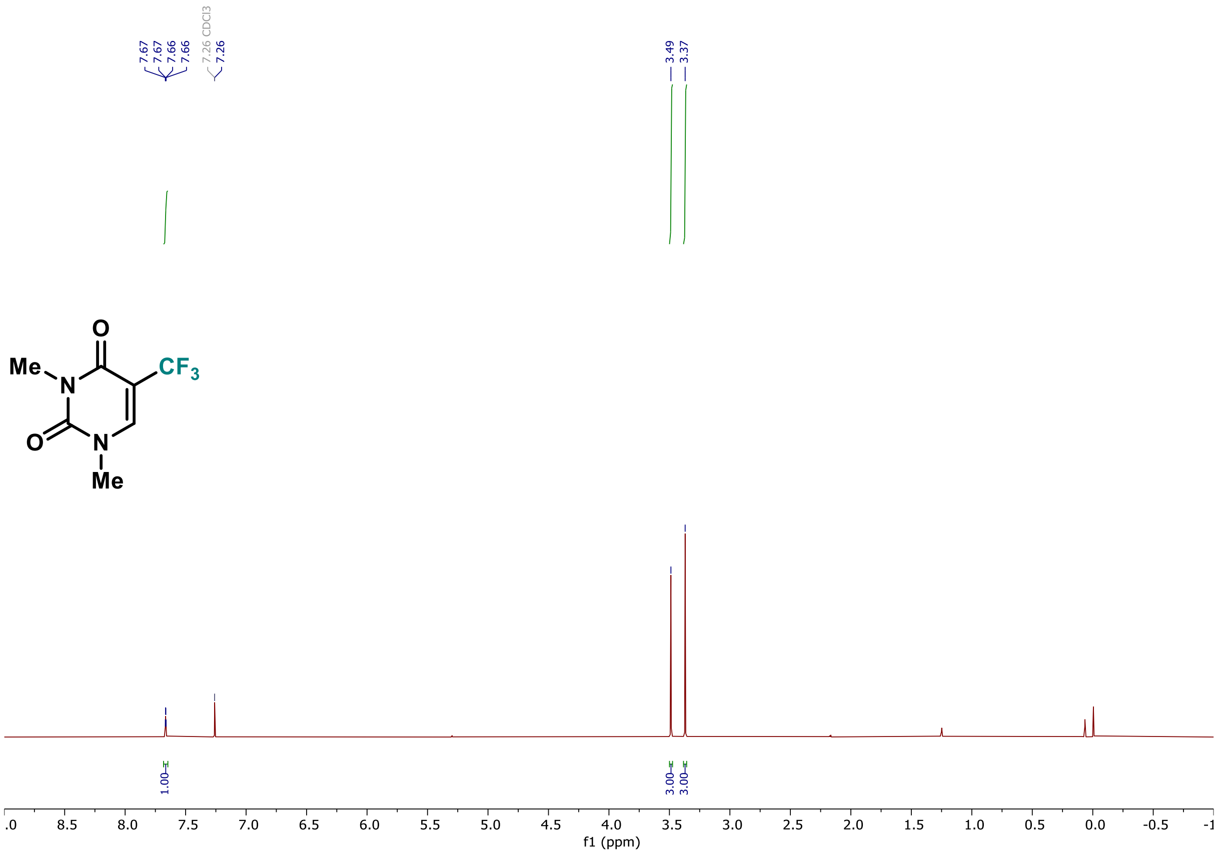

$^{13}C\{^{1}H\}$ NMR (151 MHz, $CDCl_3$)

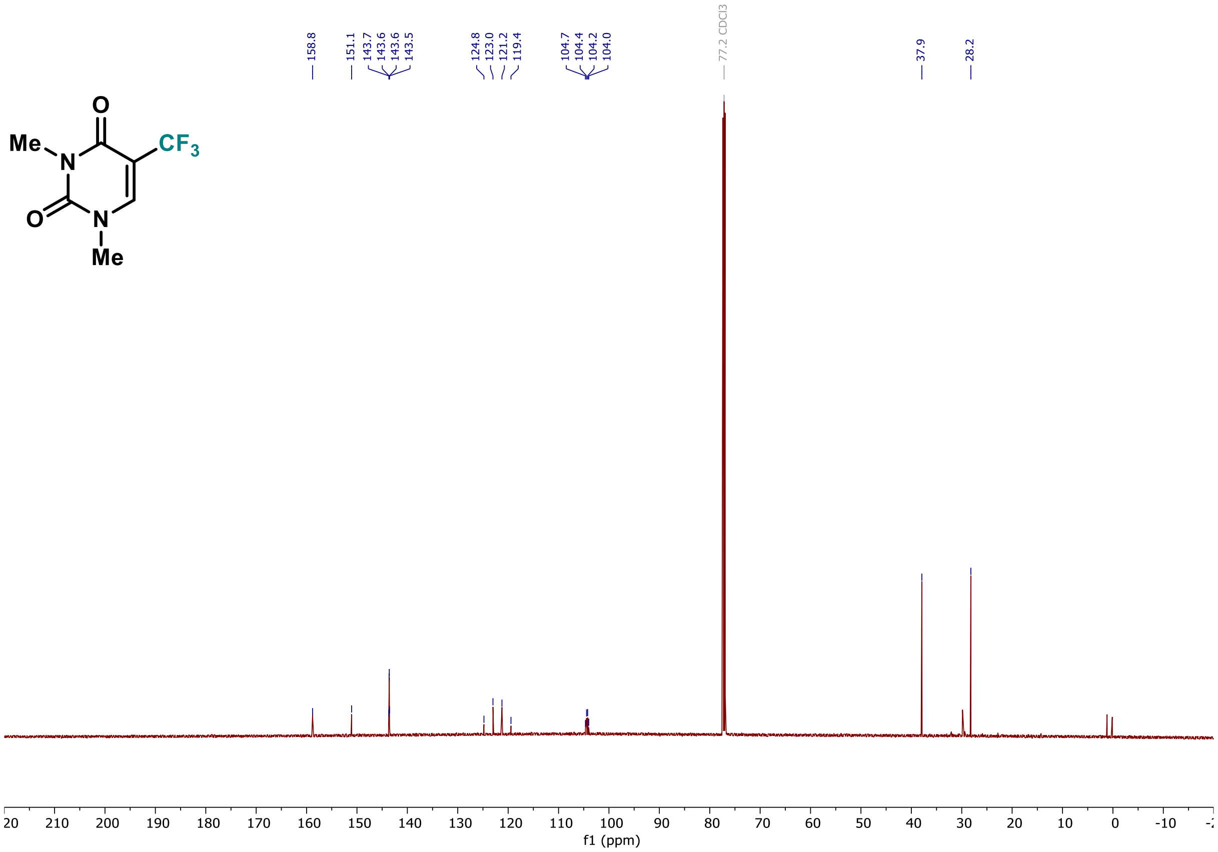

^{19}F NMR (565 MHz, $CDCl_3$)

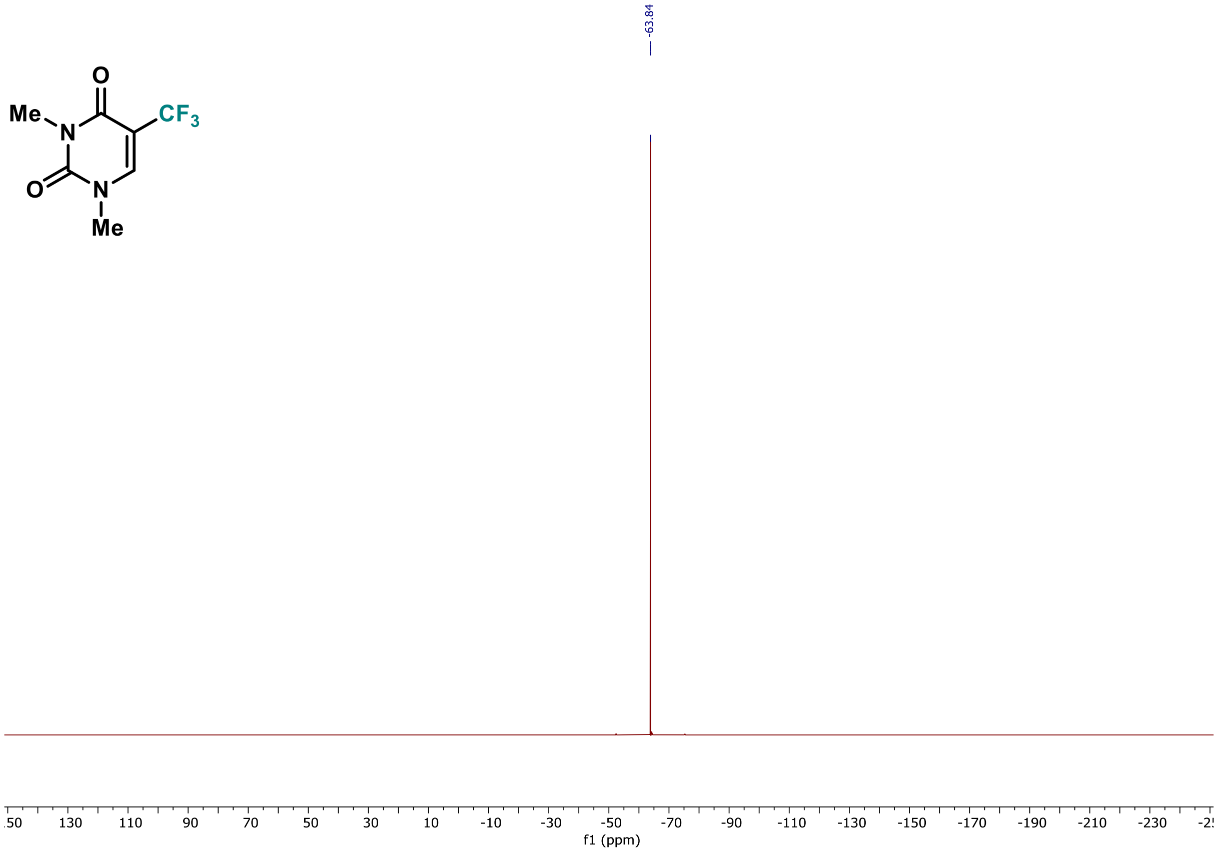

Brucin-CF$_3$ (4r)

^{1}H NMR (600 MHz, CDCl$_3$)

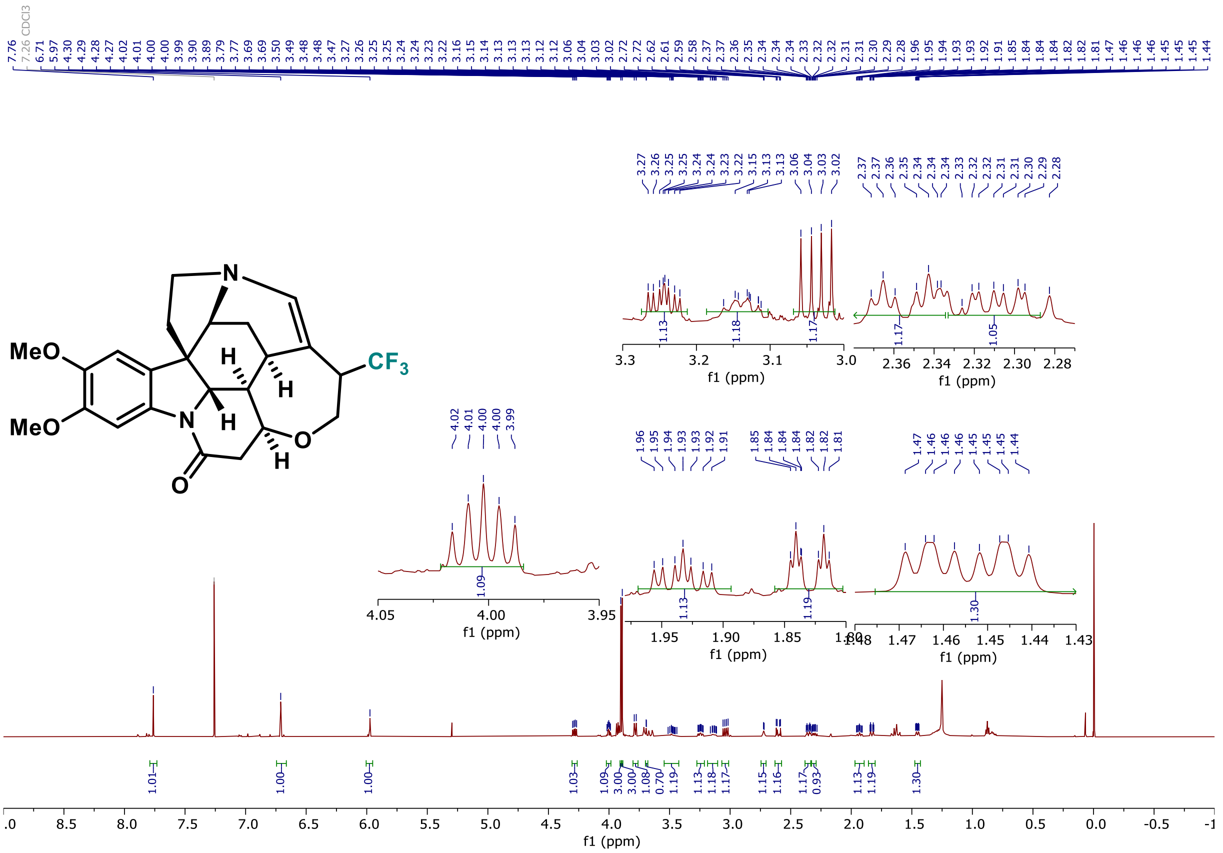

^{13}C{^{1}H} NMR (151 MHz, CDCl$_3$)

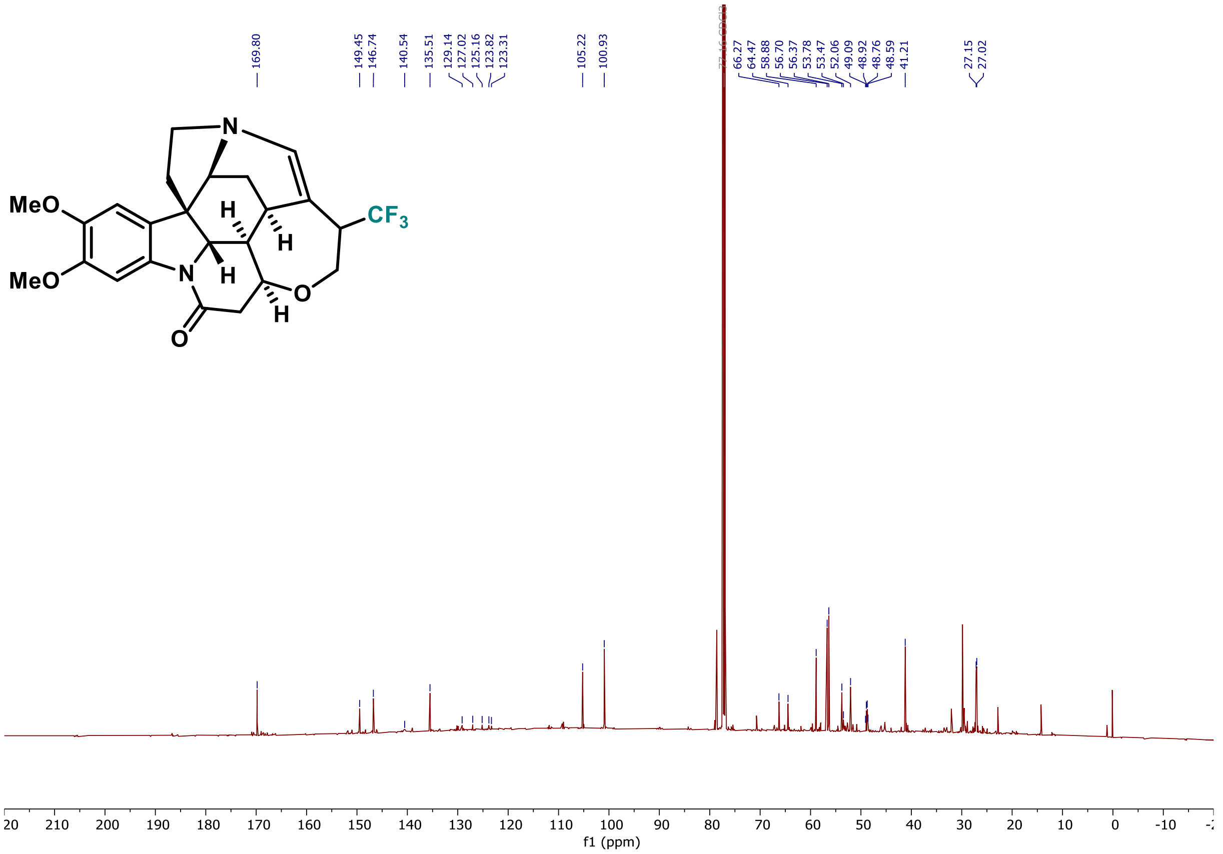

^{19}F NMR (282 MHz, CDCl$_3$)

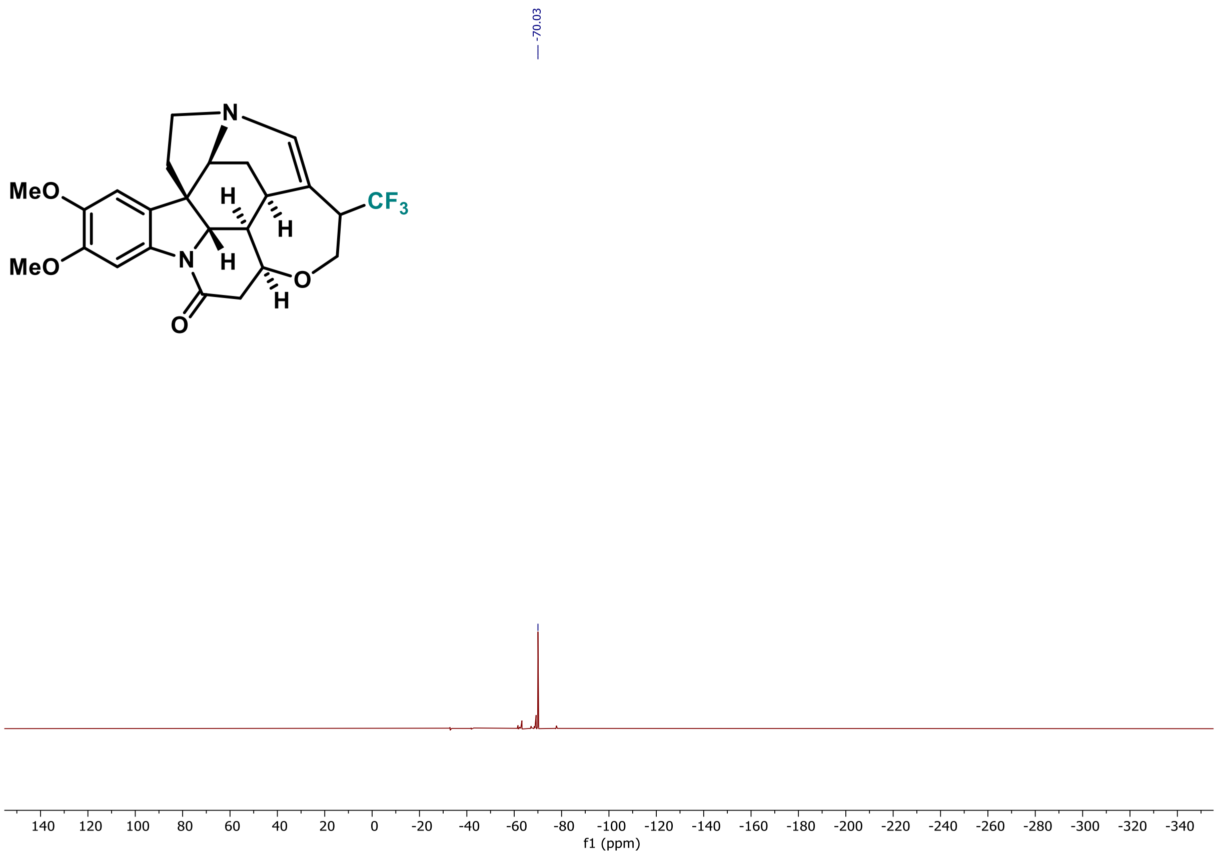

^{1}H-^{13}C HSQC

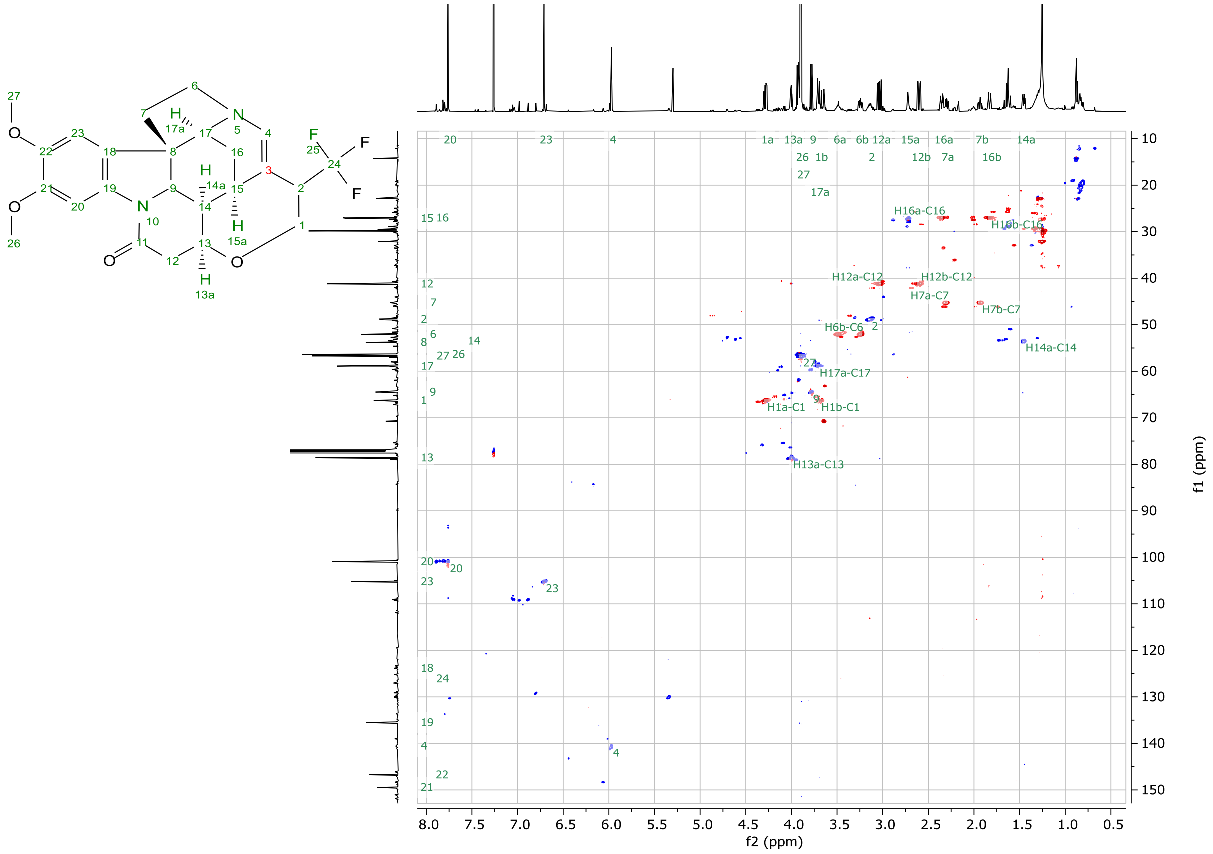

^{1}H-^{13}C HMBC

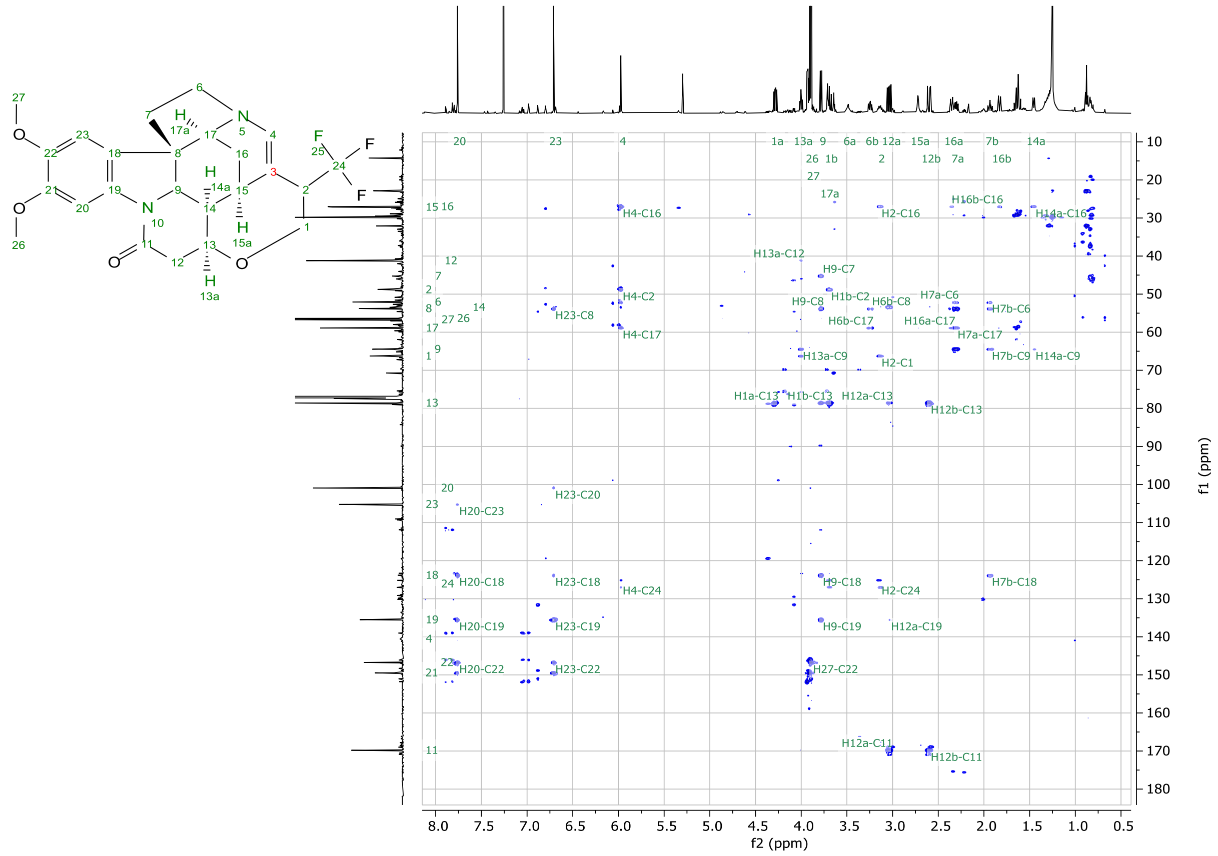

^{1}H-^{1}H COSY

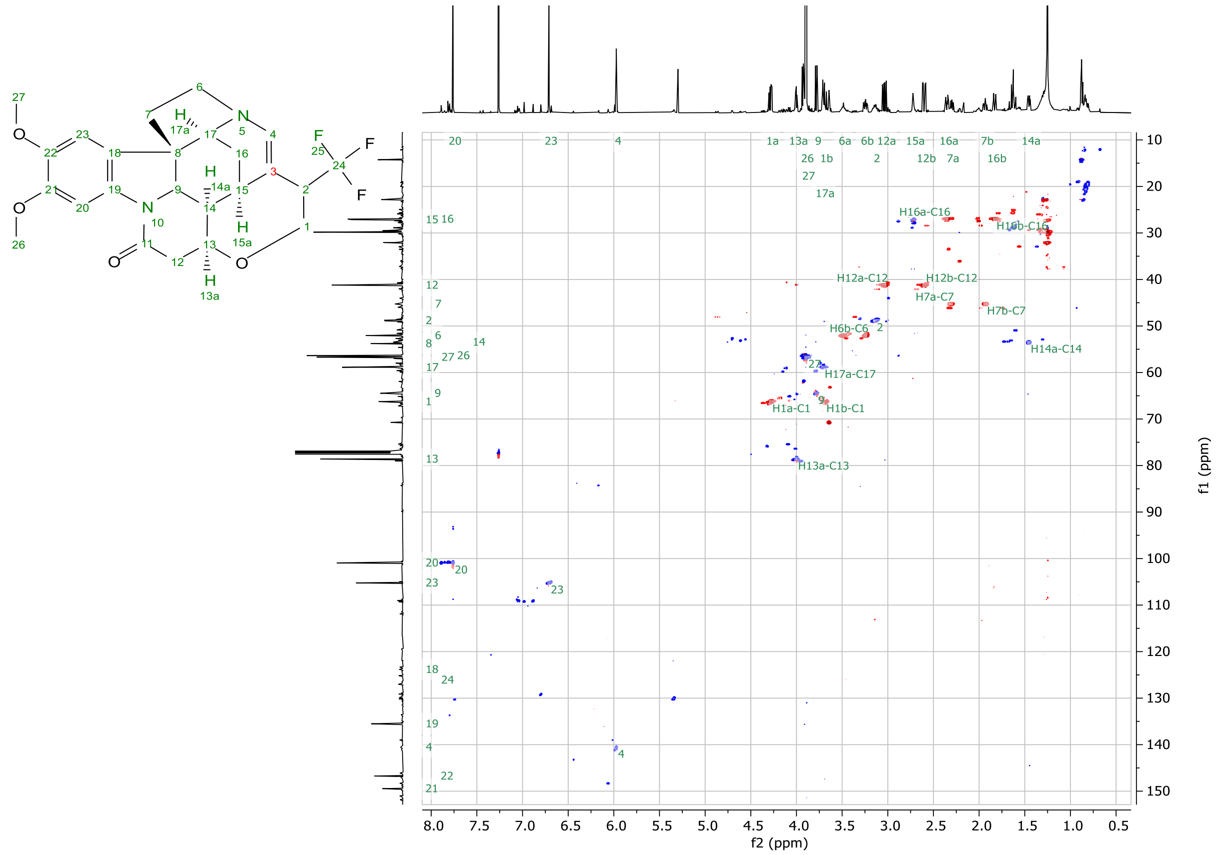

^{1}H-^{1}H COSY

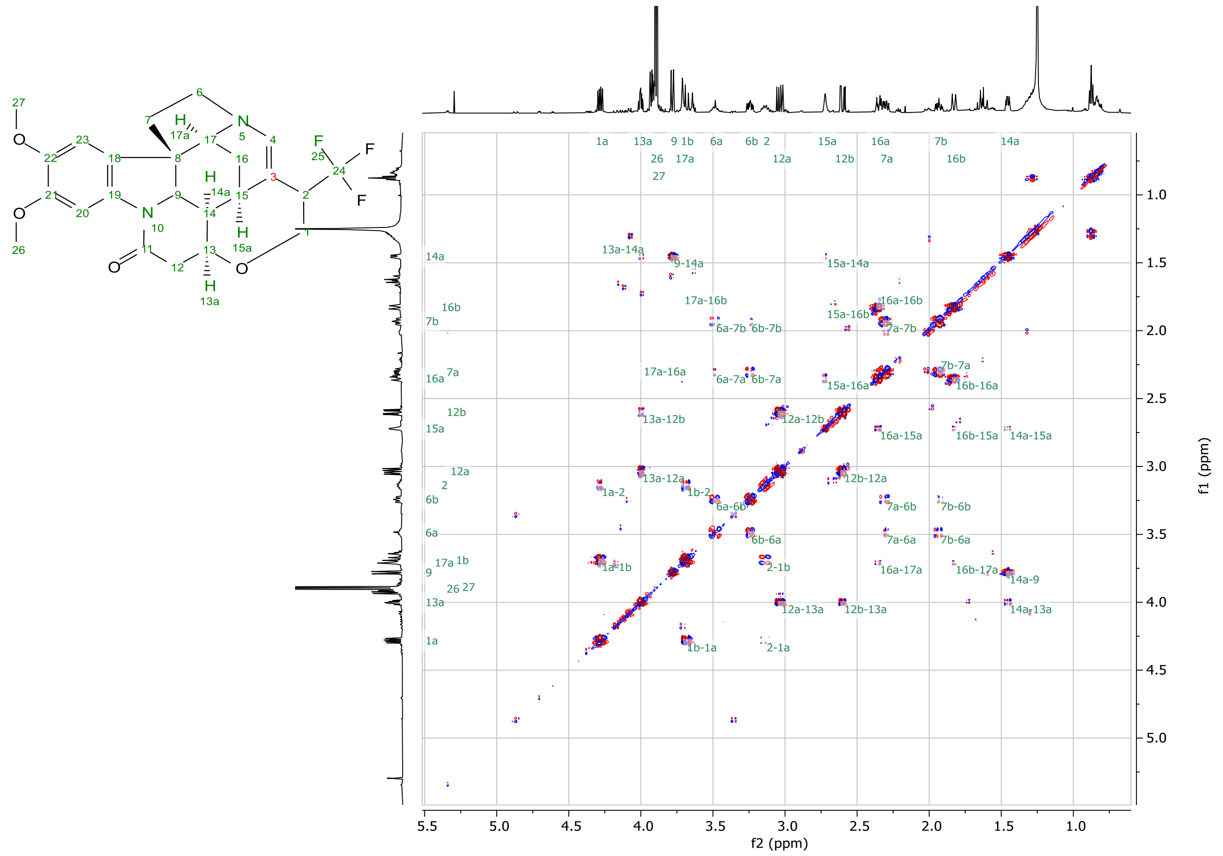

^{1}H-^{1}H NOESY

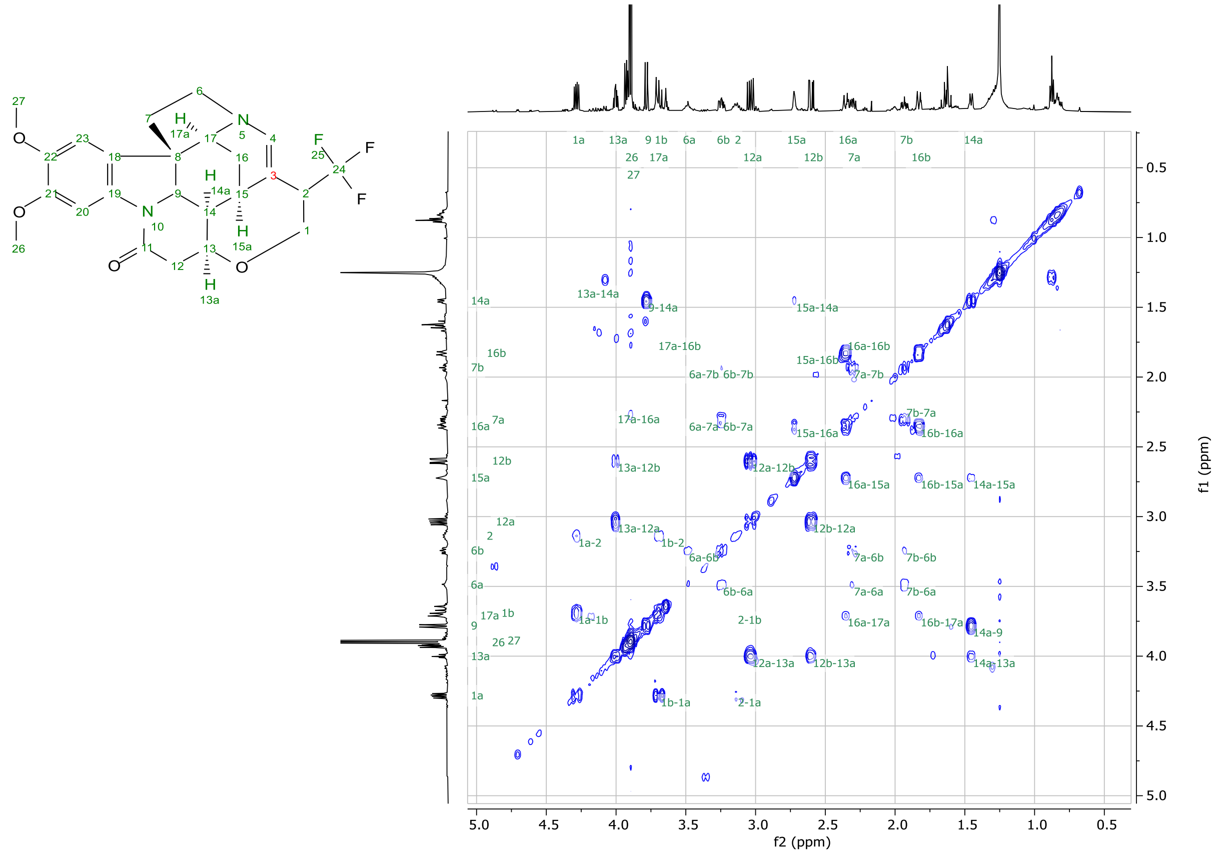

Griseofulvin-CF_3 (4s)

^{1}H NMR (600 MHz, $CDCl_3$)

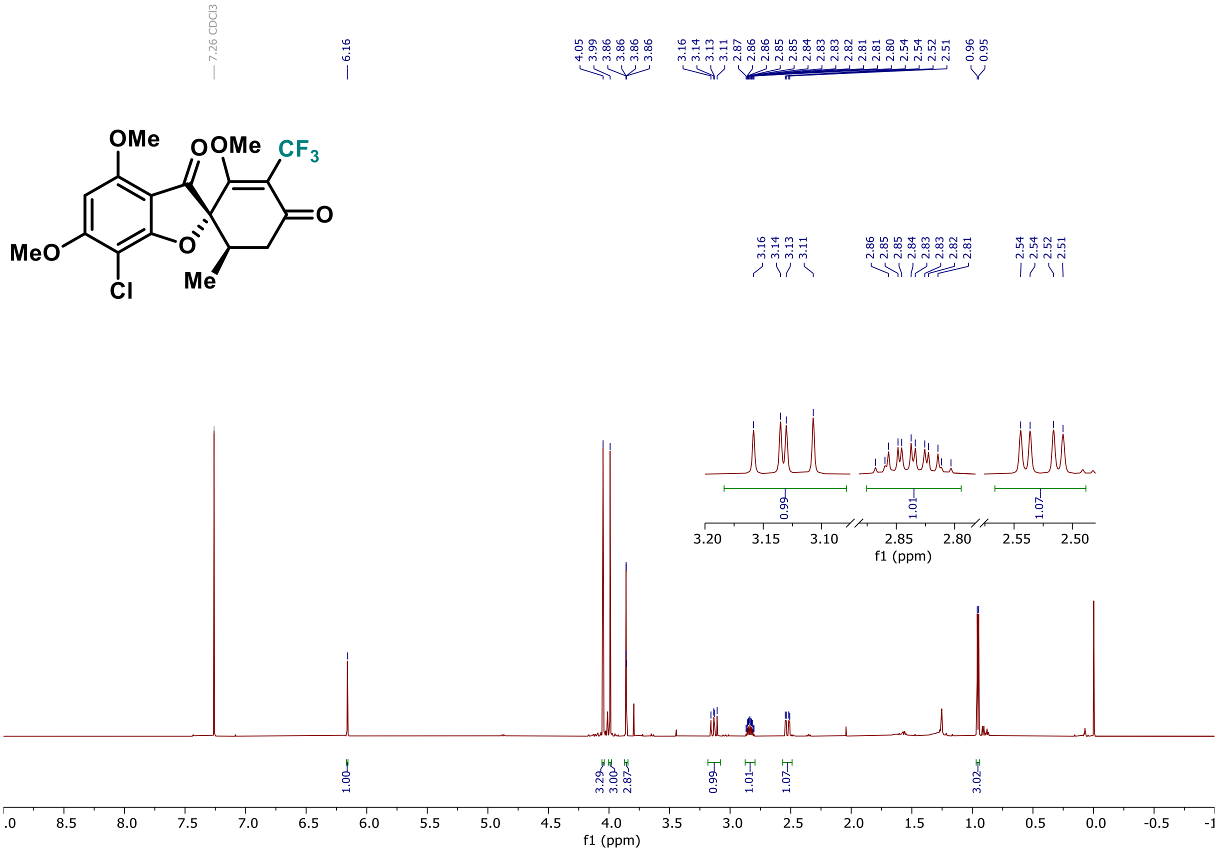

^{13}C{^{1}H} NMR (151 MHz, $CDCl_3$)

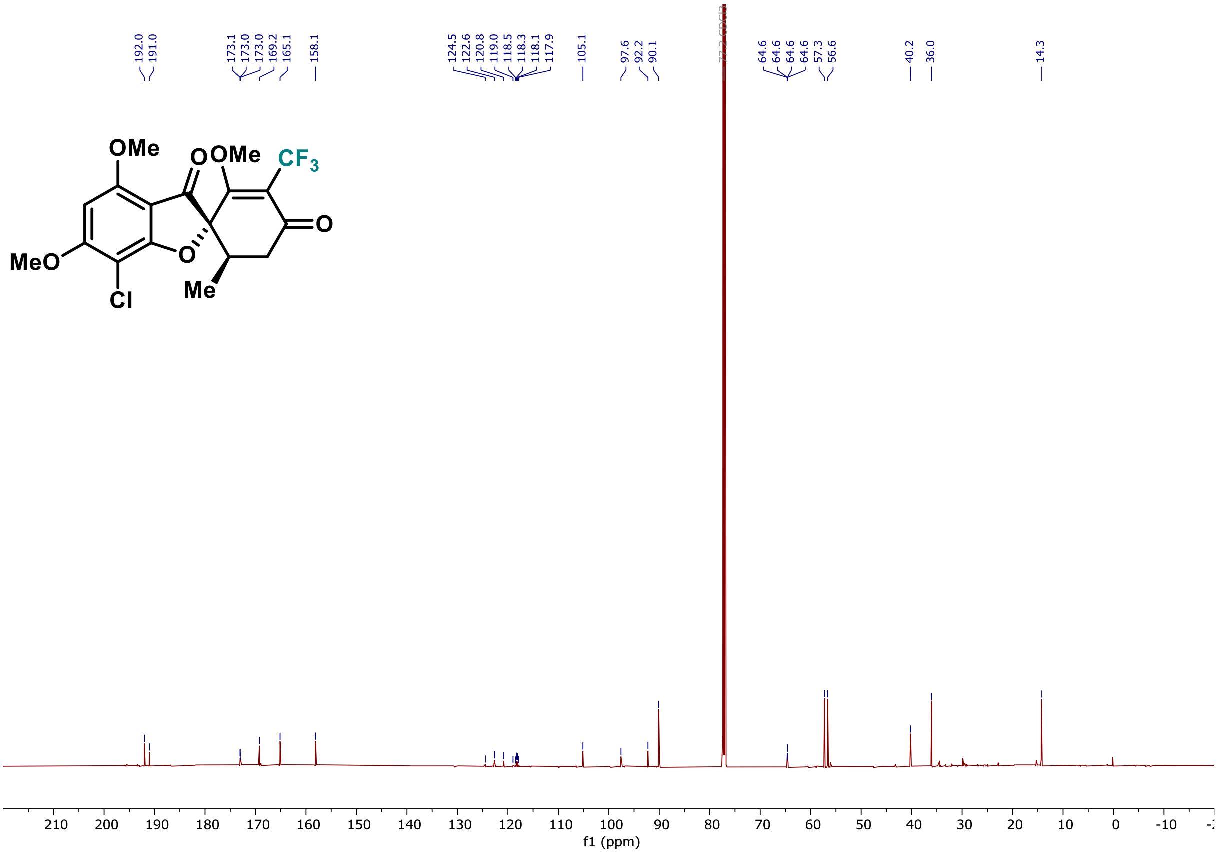

^{19}F NMR (565 MHz, $CDCl_3$)

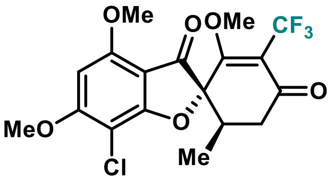

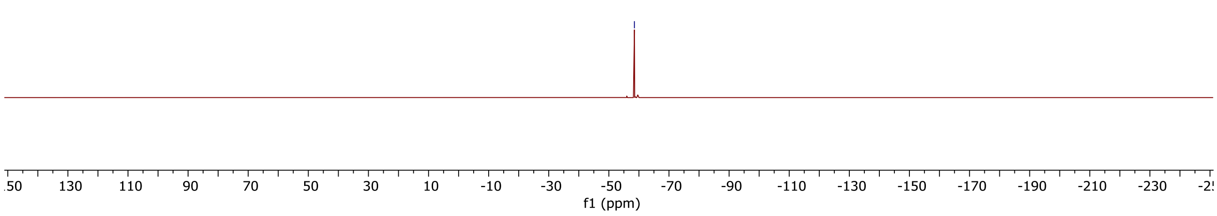

^{1}H-^{1}H COSY

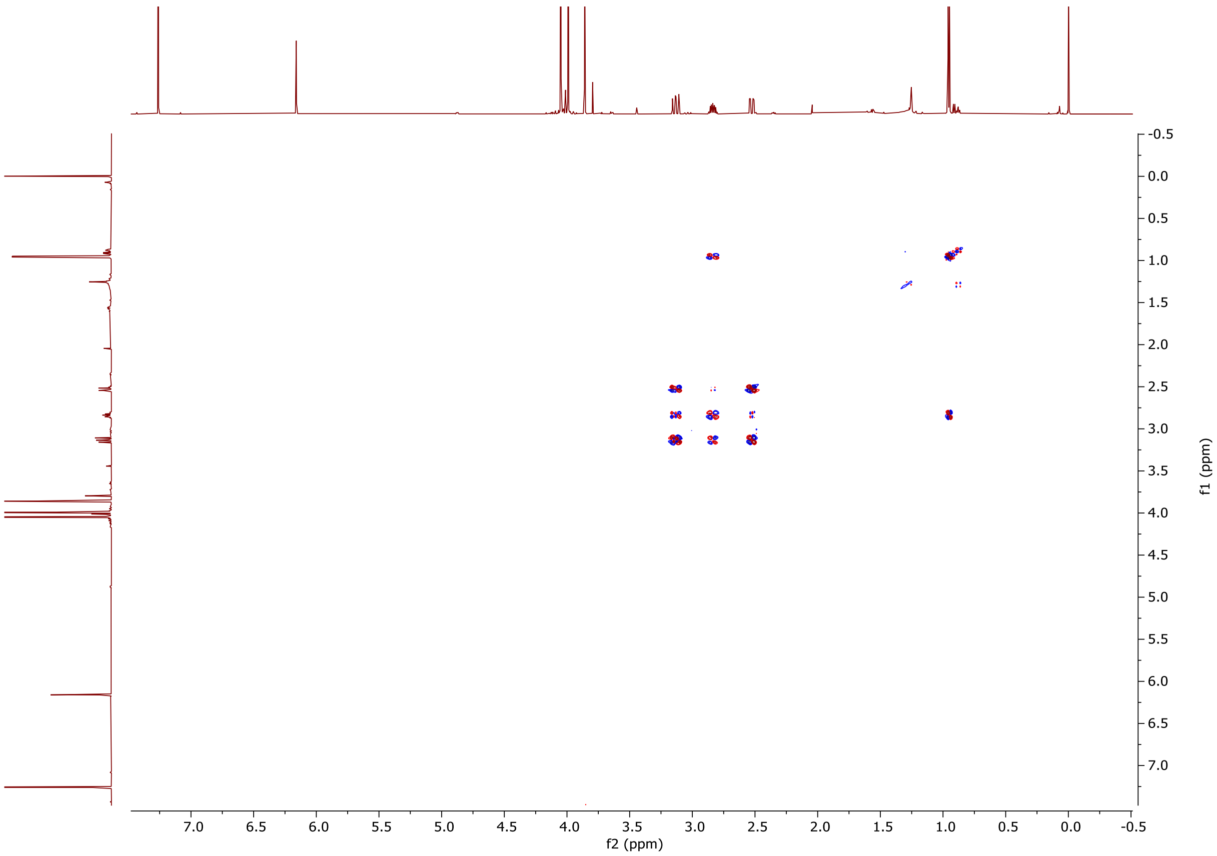

^1H-^{13}C HSQC

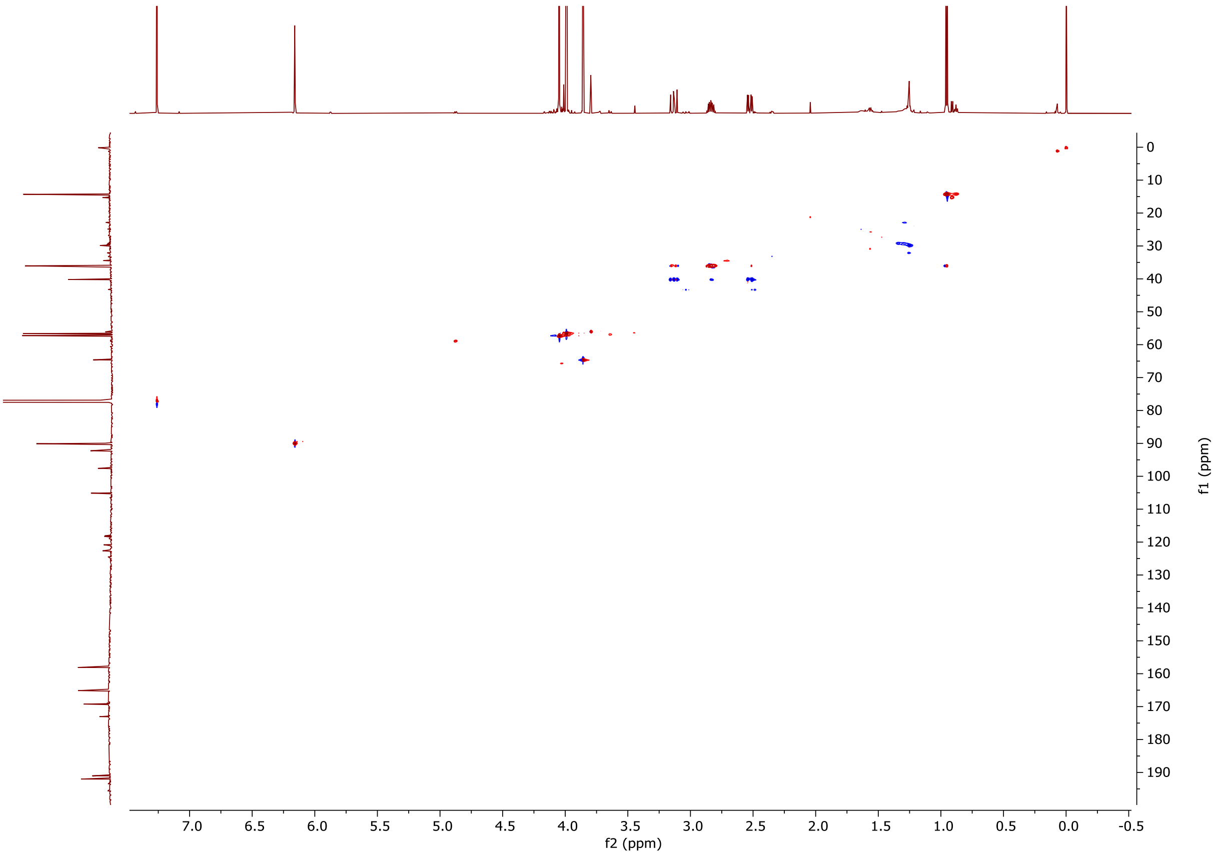

^1H-^{13}C HMBC

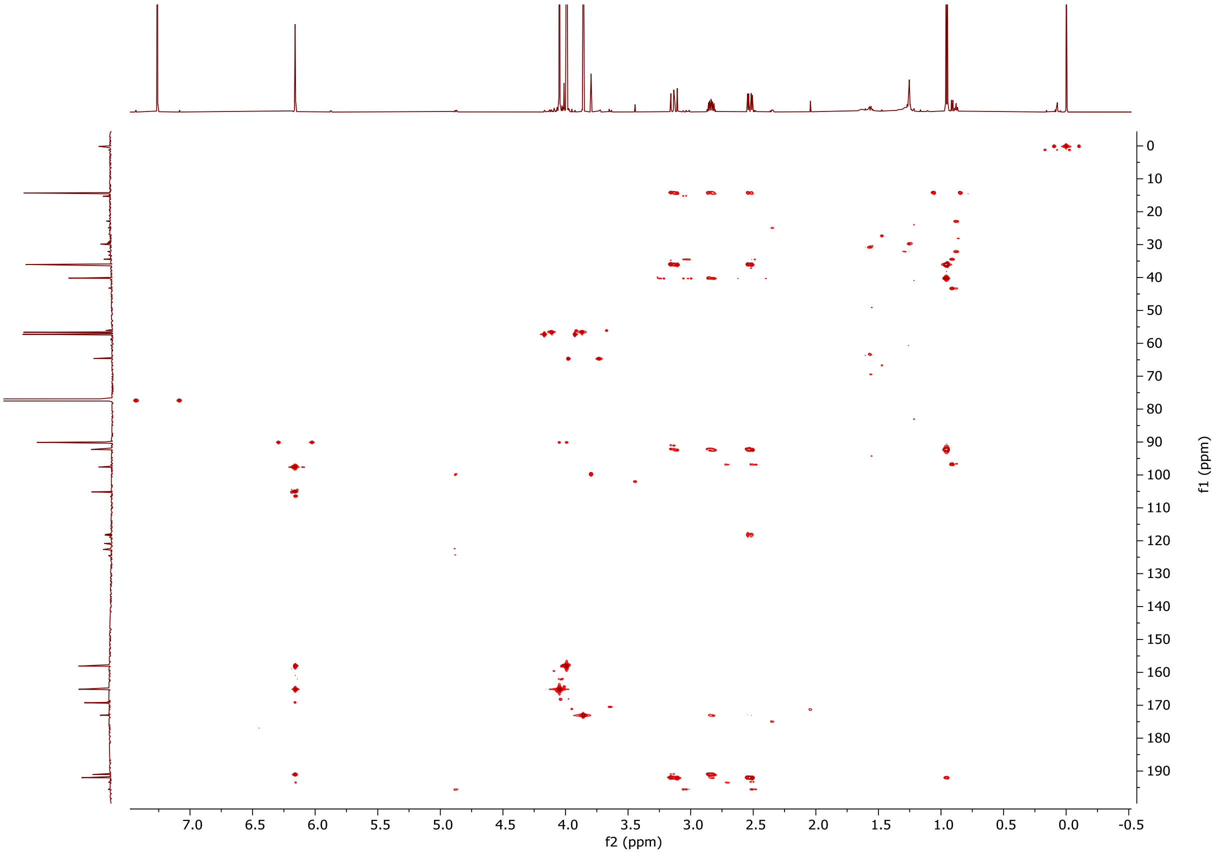

^{1}H-^{1}H NOESY

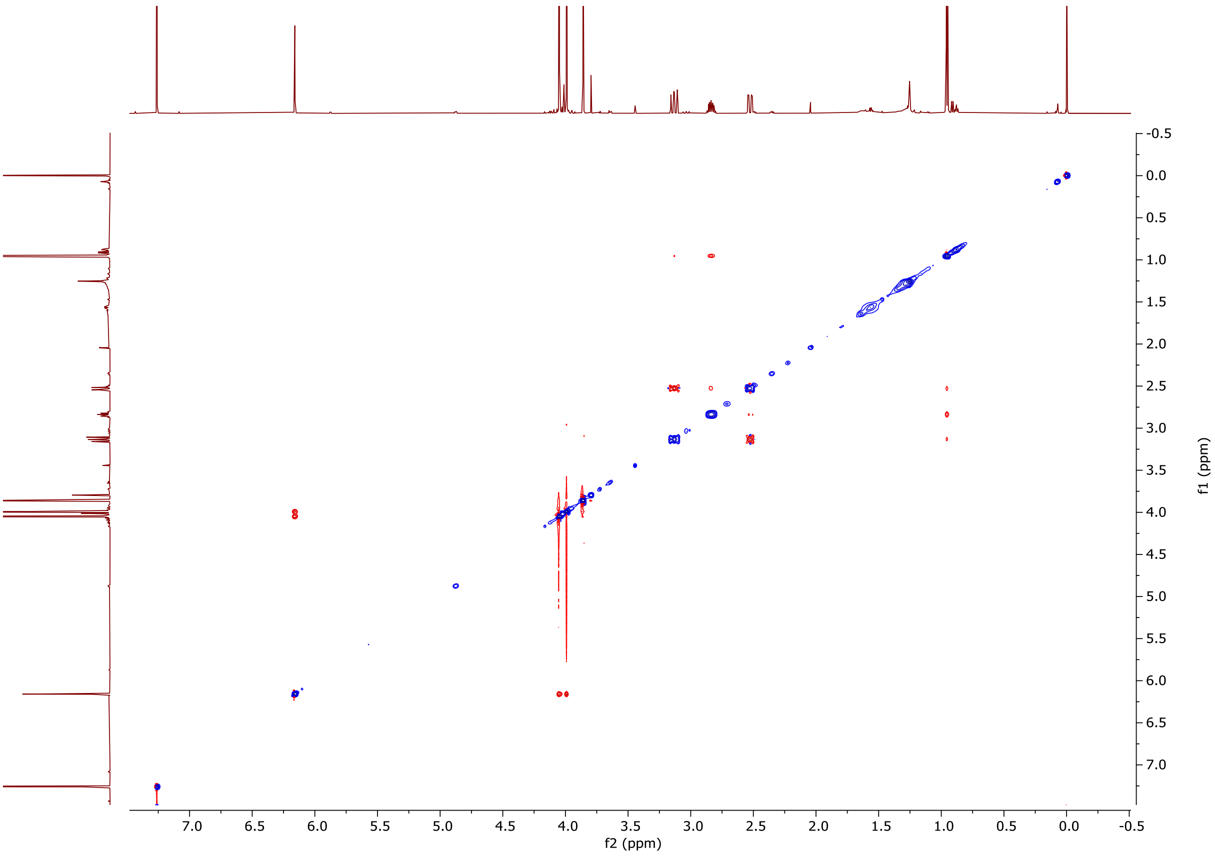

Sulfadoxine-CF_3 (4t)

^{1}H NMR (600 MHz, $CDCl_3$)

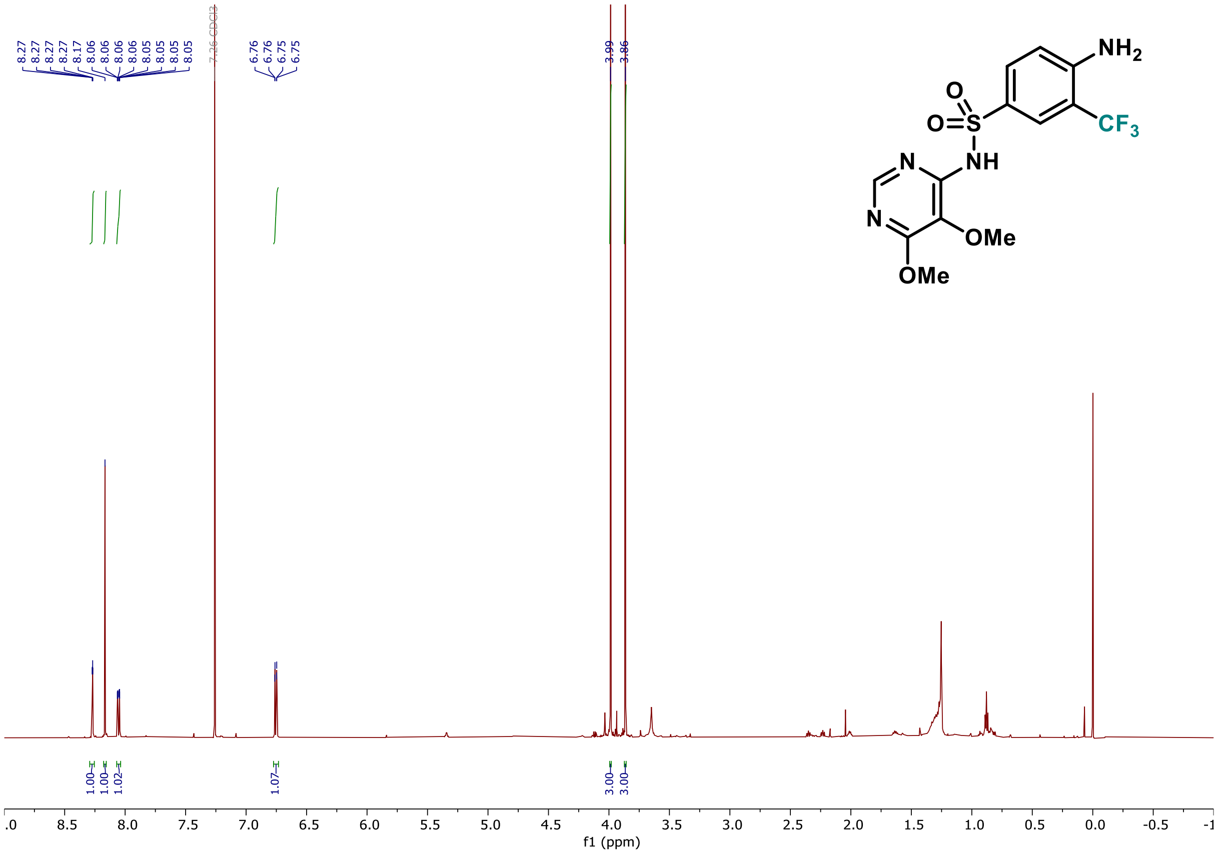

$^{13}C\{^{1}H\}$ NMR (151 MHz, $CDCl_3$)

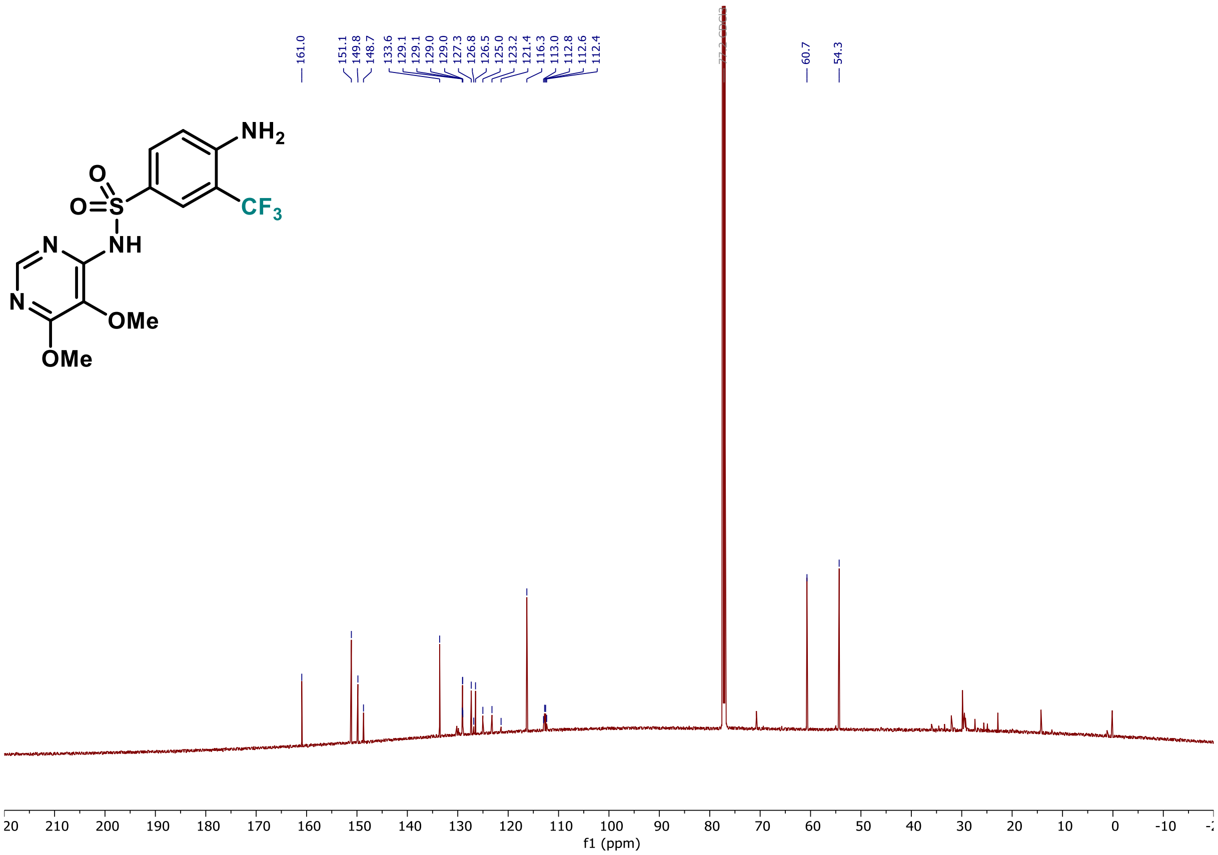

^{19}F NMR (565 MHz, $CDCl_3$)

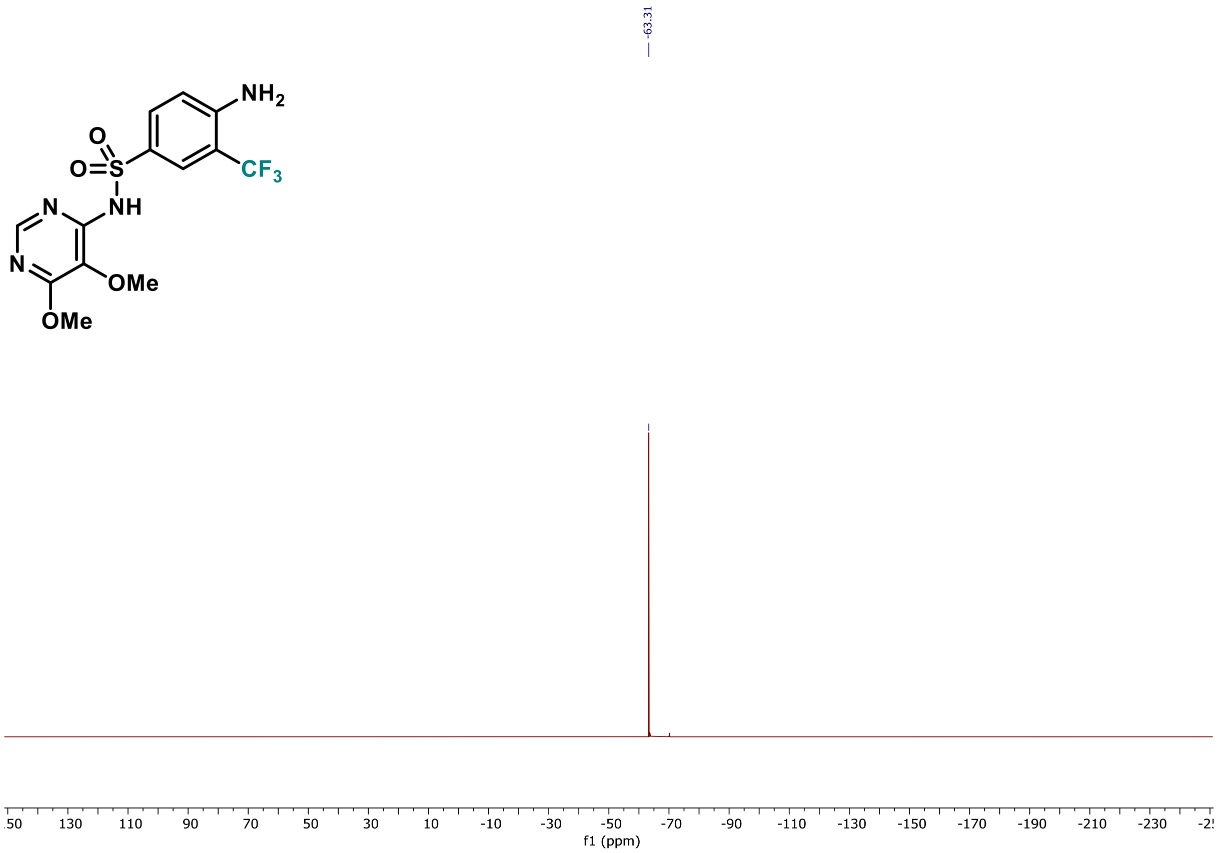

1,3,5-trimethoxy-2-(4,4,4,4,4,4,4,4,4-nonafluoro-4l12-buta-1,3-diyn-1-yl)benzene (4a’)

^{1}H NMR (300 MHz, $CDCl_3$)

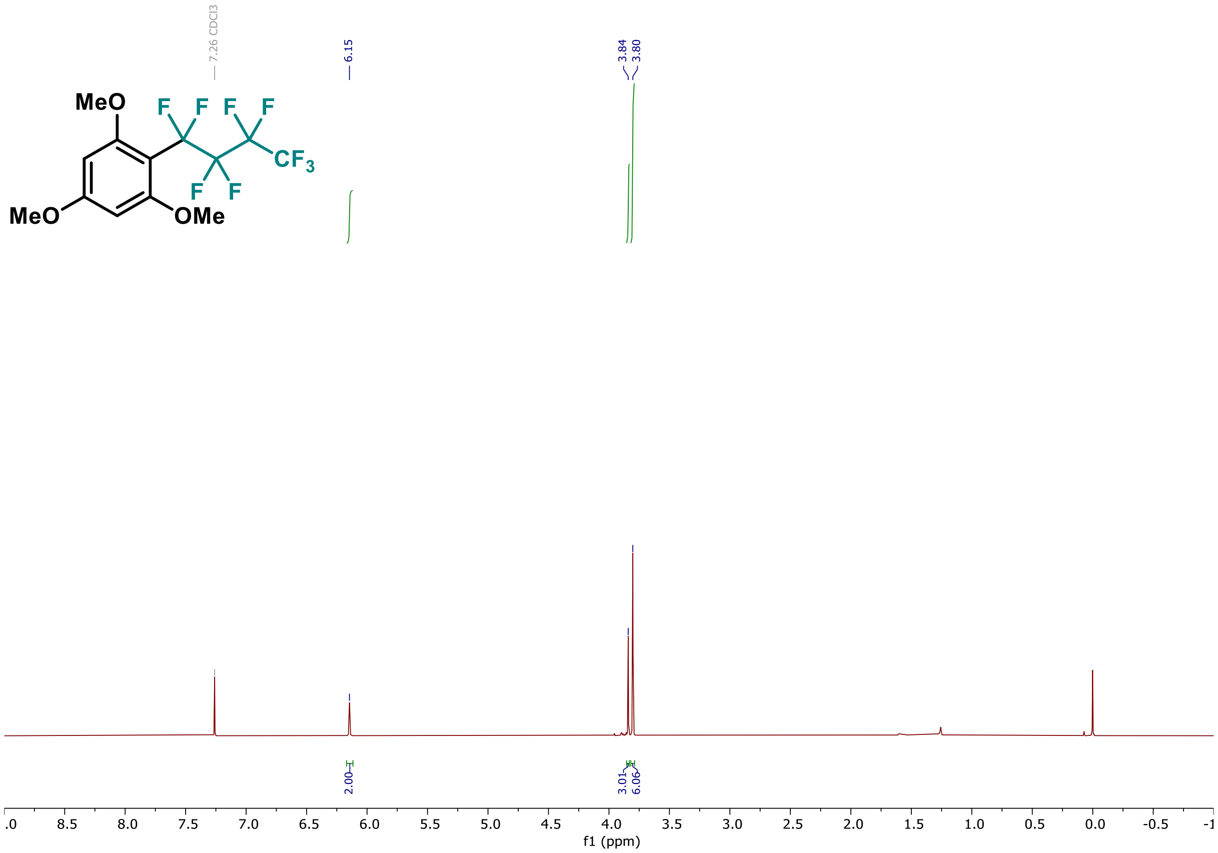

$^{13}C\{^{1}H\}$ NMR (151 MHz, $CDCl_3$)

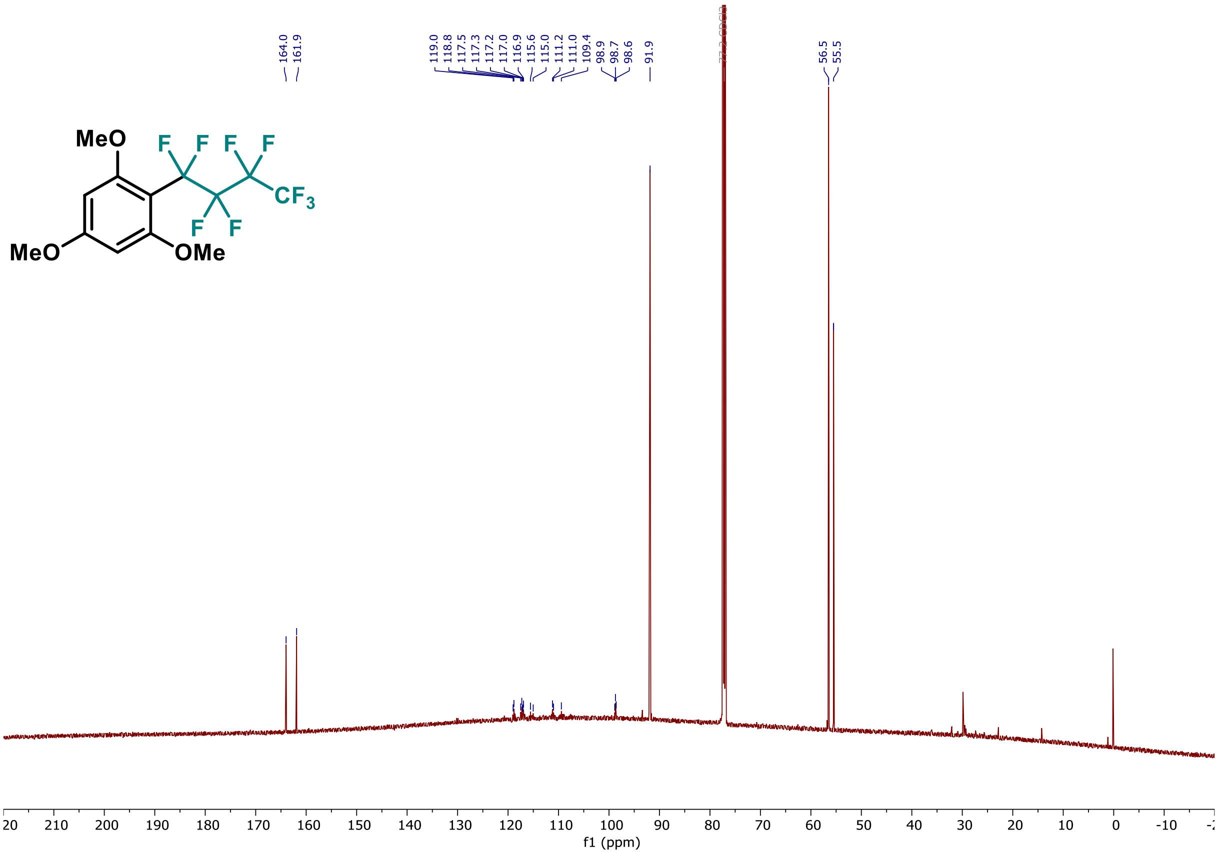

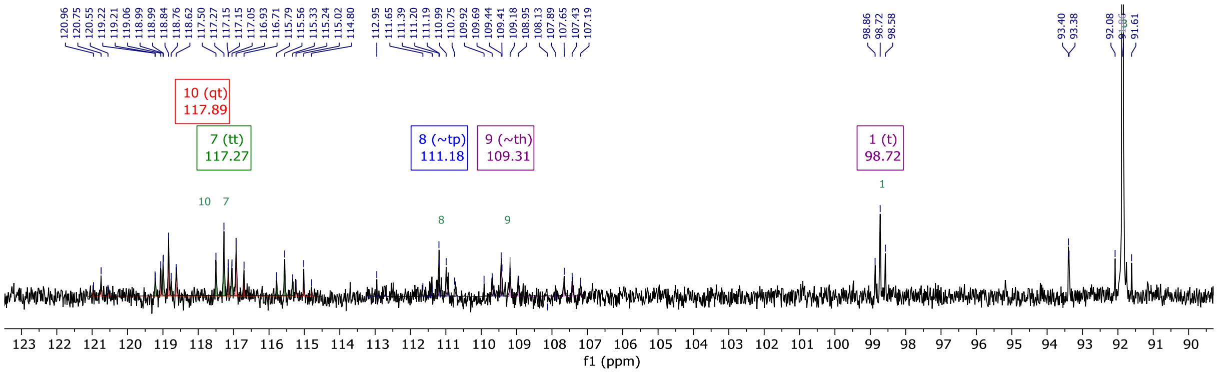

^{19}F NMR (282 MHz, $CDCl_3$)

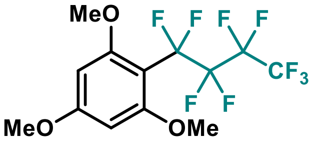

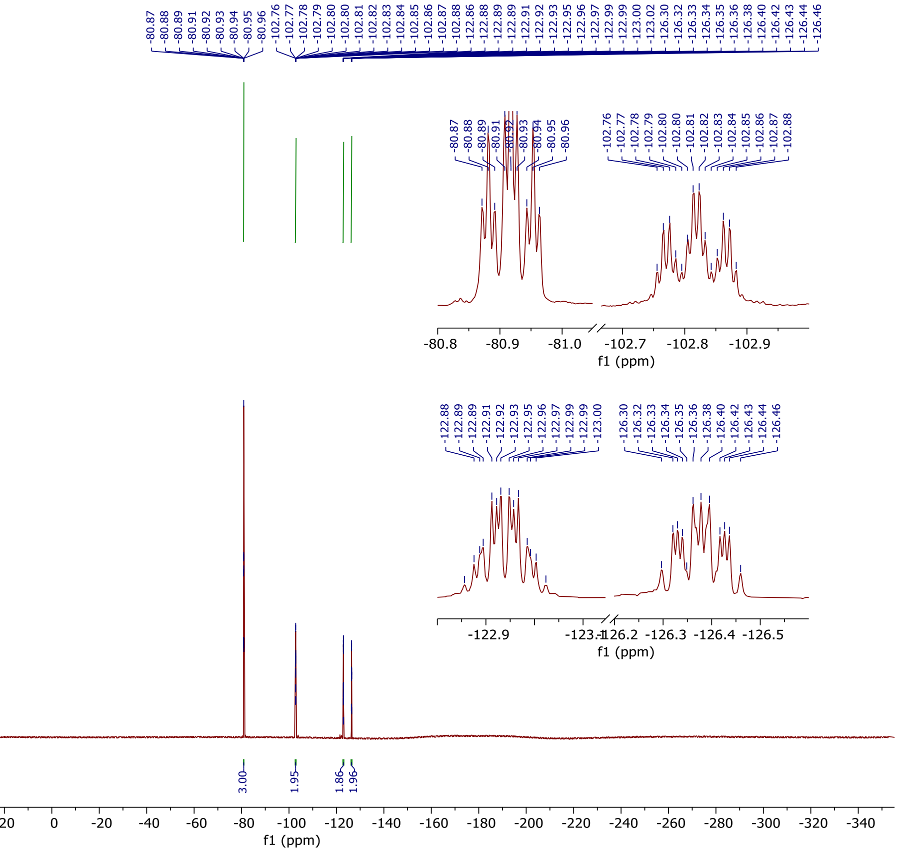

10a and **11a**

^{1}H NMR (600 MHz, $CDCl_3$)

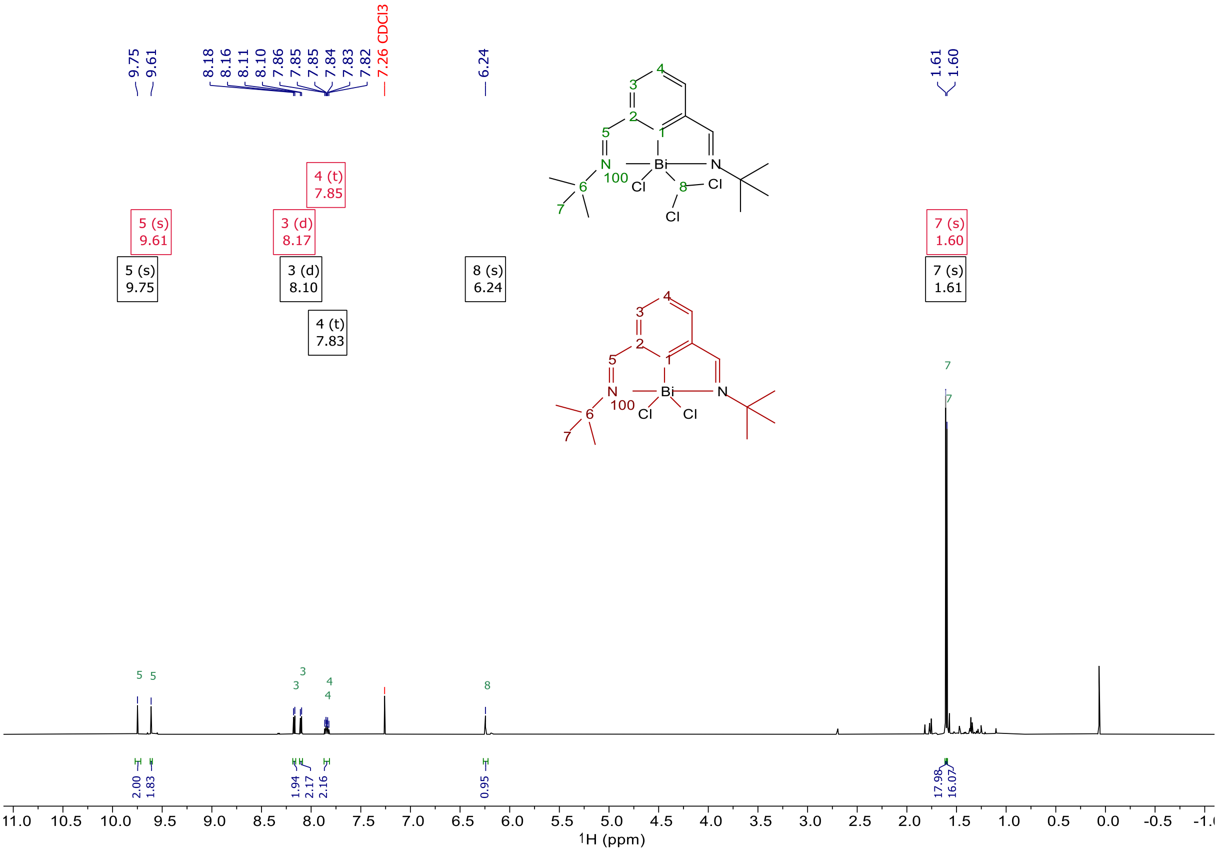

^{13}C{^{1}H} NMR (151 MHz, $CDCl_3$)

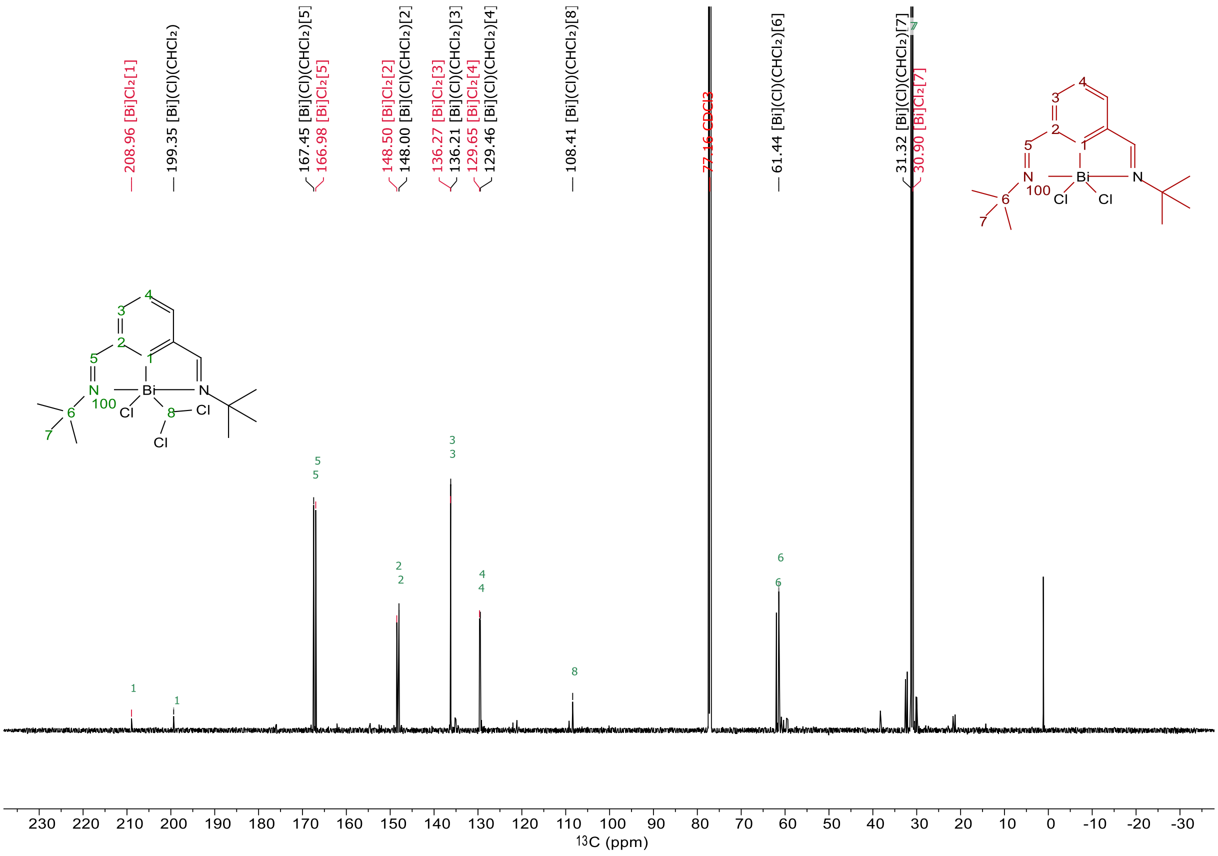

^{1}H-^{13}C HSQC

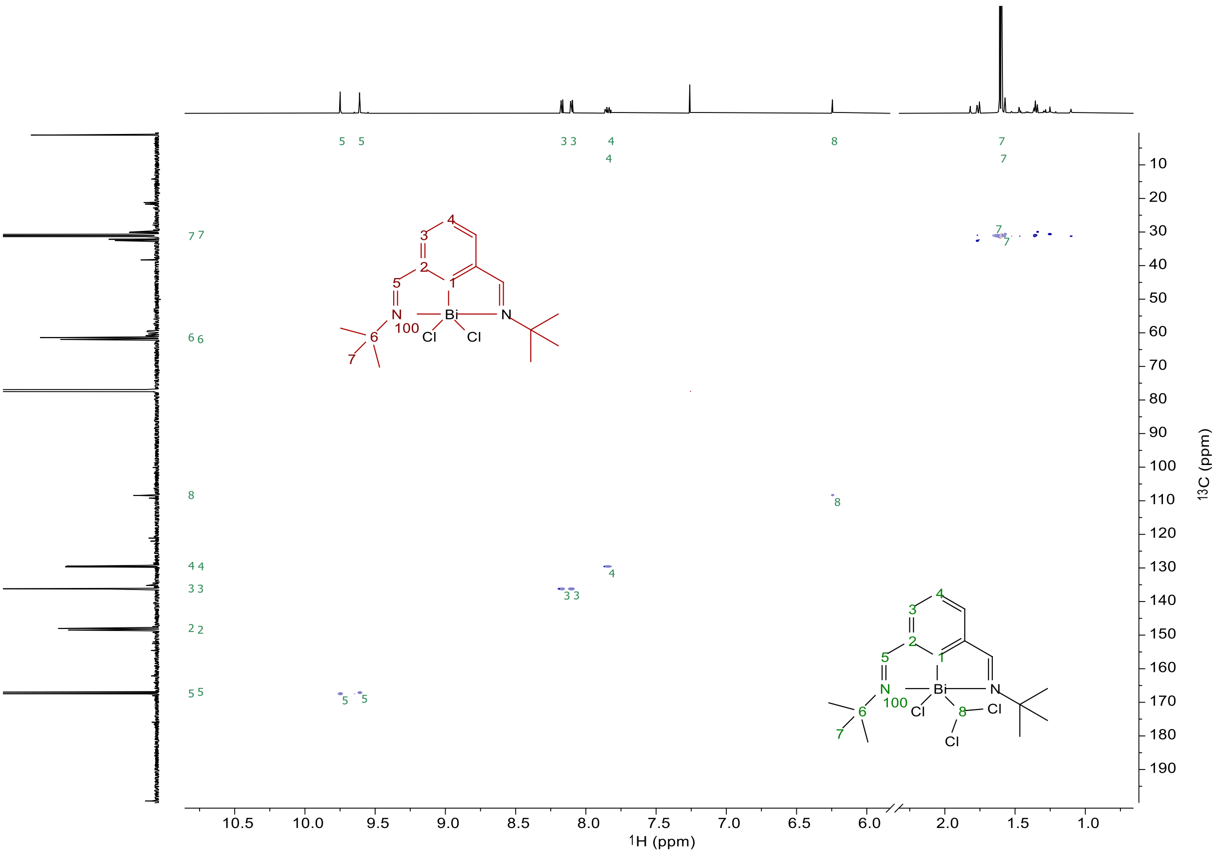

^{1}H-^{13}C HMBC

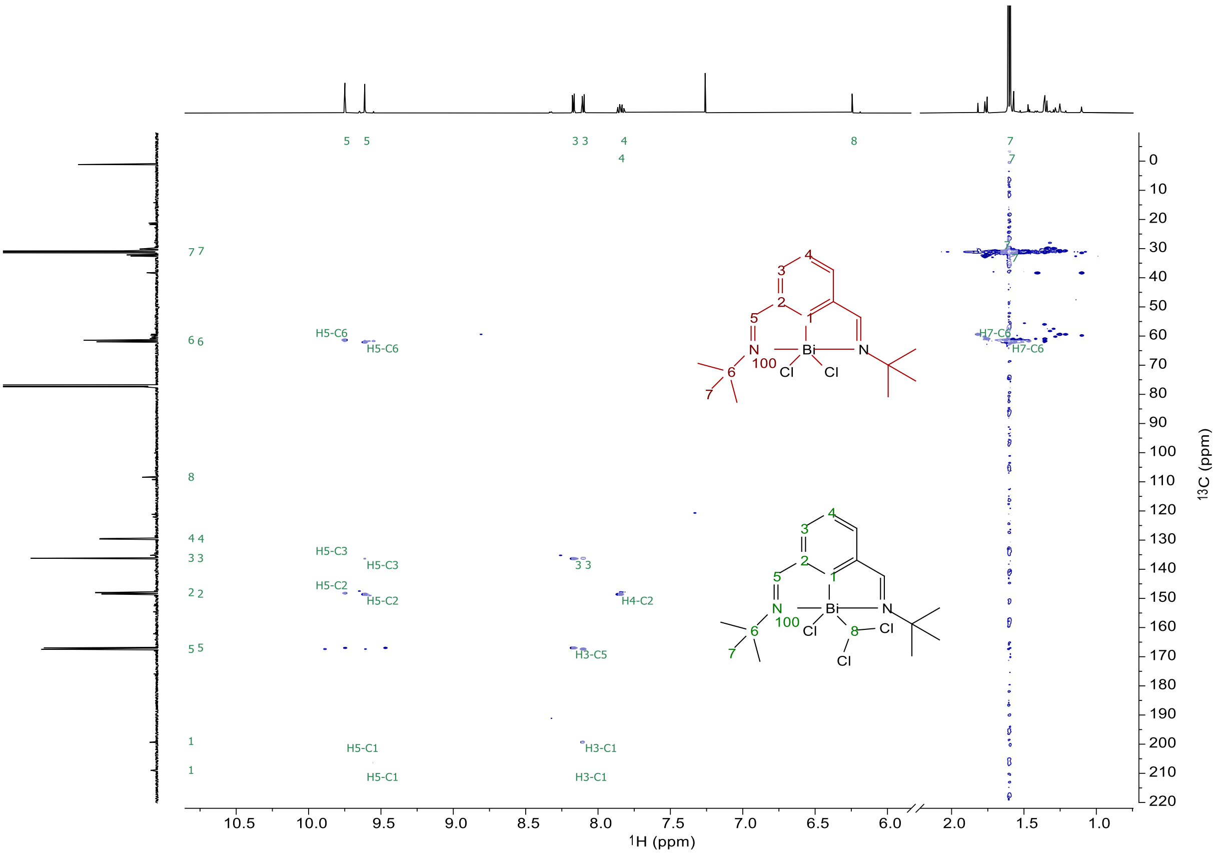

^1H-^1H COSY

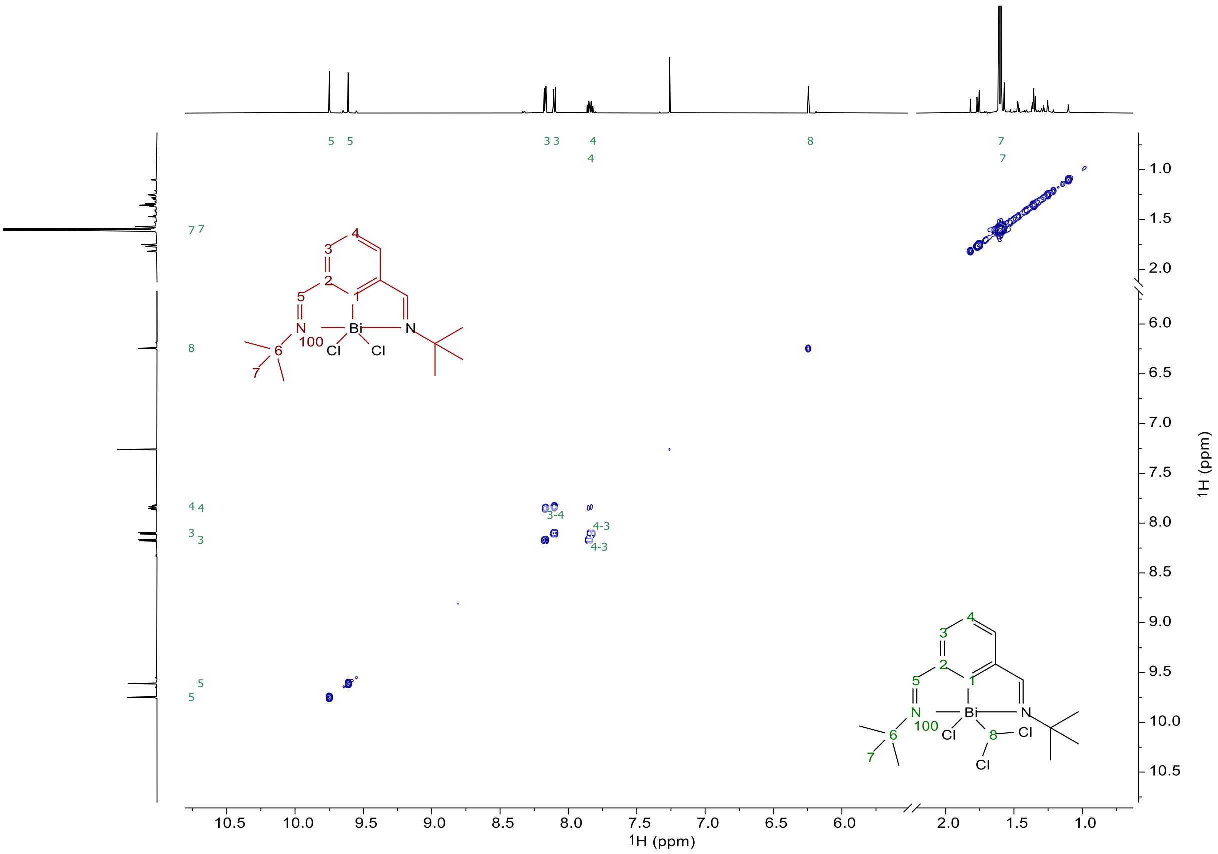

^1H-^{15}N HMBC

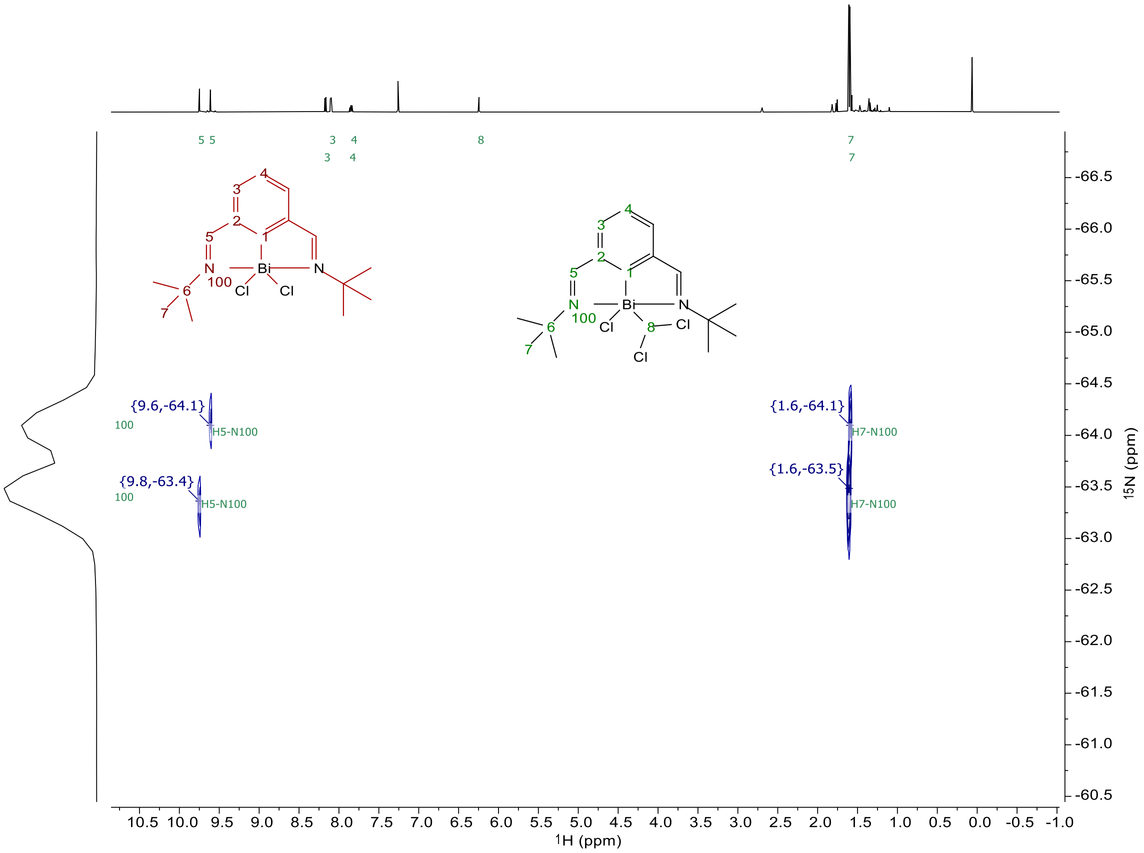

Supplement: Supplementary file 1 — ja3c10333_si_001.pdf [file ja3c10333_si_001.pdf]
